# Selective monodeuteration enabled by bisphosphonium catalyzed ring opening processes

**DOI:** 10.1038/s41467-024-53728-x

**Published:** 2024-10-30

**Authors:** Yuanli Xu, Wenlong Chen, Ruihua Pu, Jia Ding, Qing An, Yi Yang, Weimin Liu, Zhiwei Zuo

**Affiliations:** 1 Innovation Center for Chenguang High Performance Fluorine Material, Key Laboratory of Green Chemistry of Sichuan Institutes of Higher Education, Sichuan University of Science and Engineering, 643000 Zigong, China; 2 State Key Laboratory of Organometallic Chemistry, Shanghai Institute of Organic Chemistry, Chinese Academy of Sciences, 200032 Shanghai, China; 3 School of Physical Science and Technology, ShanghaiTech University, 201210 Shanghai, China

**Keywords:** Photocatalysis, Synthetic chemistry methodology

## Abstract

The selective incorporation of a deuterium atom into small molecules with high selectivity is highly valuable for medical and chemical research. Unfortunately, this remains challenging due to the complete deuteration caused by commonly used hydrogen isotope exchange strategies. We report the development of a photocatalytic selective monodeuteration protocol utilizing C–C bond as the unconventional functional handle. The synergistic combination of radical-mediated C–C bond scission and deuterium atom transfer processes enables the effective constructions of benzylic CDH moieties with high selectivity for monodeuteration. The combinational use of a bisphosphonium photocatalyst, thiol catalyst, and CH_3_OD deuteration agent provides operationally simple conditions for photocatalytic monodeuteration. Moreover, the photoinduced electron transfer process of the bisphosphonium photocatalyst is elucidated through a series of spectroscopy experiments, identifying a peculiar back electron transfer process that can be regulated by subsequent nucleophilic additions.

## Abstract (web summary)

Incorporation of deuterium into small molecules in the place of hydrogens can alter the properties of the molecule in ways that provide benefits without affecting the core function of the molecule. Here, the authors report a ring-opening deuteration of cyclic alkanes via biphosphonium photocatalysis.

## Introduction

The selective incorporation of deuterium atoms into small molecules is of great importance in medicinal and chemical research^1–4^. Compared to their nondeuterated analogues, deuterium-labeled compounds exhibit unique metabolism and pharmacokinetic properties, which have been frequently exploited to increase the bioavailability of pharmaceutical candidates^5,6^. Deuterated compounds have also been used as metabolic tracers and mass spectrometry analytic standards^7^. In organic chemistry, kinetic isotope effect (KIE) experiments with mono-deuterated or fully-deuterated compounds have been widely used in mechanistic investigations to elucidate reaction pathways^8,9^. Despite widespread uses, the preparation of deuterium-labeled molecules with high selectivity oftentimes are rather challenging tasks, as the separation of unlabeled or partially labeled materials from the desired deuterated product has been impeded by the trivial difference in physical properties.

**Fig. 1:**
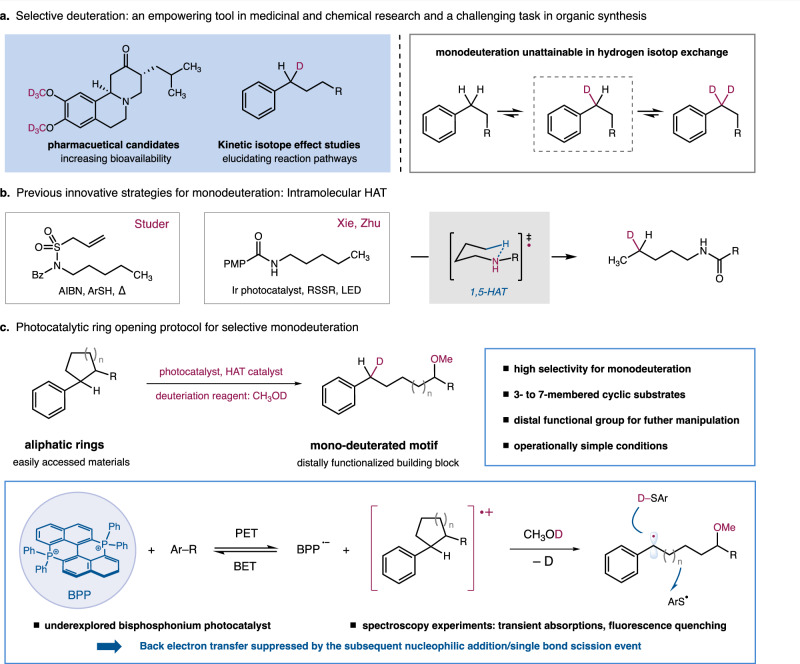
Selective monodeuteration via photoredox catalysis. **a** Selective deuteration: an empowering tool in medicinal and chemical research and a challenging task in organic synthesis. **b** Previous innovative strategies for monodeuteration: Intramolecular HAT. **c**. Photocatalytic ring opening protocol for selective monodeuteration.

With the advancement of transition metal catalysis, tremendous progress has been made in H/D exchange reactions, enabling various environmentally benign and operationally simple protocols^2,10–18^. In this manifold, the selectivity problem can be addressed through the complete deuteration of an aliphatic carbon which can be easily achieved by thermodynamic effect^19^, while the selective incorporation of a single deuterium into methylene and methyl groups is deemed impractical due to over-deuteration (Fig. 1)^20^. Thusly, the preparation of monodeuterated molecules heavily relies on the use of prefunctionalized substrates such as alkyl halides. The necessities of expensive deuteration reagents as well as multistep conversions has raised urgent needs to develop operationally simple and sustainable methods^21–30^. Recently, selective monodeuteration of C(sp^3^)–H bonds have been realized through elegantly designed intramolecular 1,5-HAT processes by Studer, Xie, and Zhu groups^31,32^. With the combinational uses of radical precursors with AIBN, or amide substrates with Ir photocatalyst, monodeuterated amides can be obtained with high selectivity. Considering the peculiar uses of mono-deuterated compounds in KIE studies and pharmaceutical investigations^5,9^, the development of innovative strategies and catalytic protocols remains in high demand.

Radical-mediated C–C bond cleavage and functionalizations, with emerging photoredox catalysis as the enabling platform to generate open-shell radical intermediates, have provided a synthetically valuable strategy to exploit ubiquitous C(sp^3^)–C(sp^3^) bonds as functional handles^33,34^. Under ambient temperature, a high-energy radical species such as alkoxy radical or benzene radical cation could promote the homolytic cleavage of the adjacent C–C single bond to generate a more stabilized carbon-centered radical^35,36^. Importantly, the resultant alkyl radical intermediate could undergo diversified conversions which have provided intriguing opportunities for functional group installations. As such, a series of ring-opening transformations have been developed over the last decade, demonstrating various intriguing applications, including oxidations, halogenation, alkylation, and arylation for the syntheses of distally functionalized carbonyl compounds^37–56^. We recently wondered whether a radical-mediated deuterium transfer event could be incorporated in the photocatalytic ring opening process for the selective mondeuteration, providing an intriguing strategy to utilize inert and ubiquitous C(sp^3^)–C(sp^3^) bonds as functional handles for deuterium incorporation. Compared to radical-mediated H/D exchange where conversion rates suffer from the small driving force (difference in zero-point energy, ~1 kcal/mol), ring-opening deuterations take advantage of the substantial energy gain in strain release (strain energies of 3- to 5- membered aliphatic rings, 6-27 kcal/mol) to enhance efficient deuteration.

Given the prevalence of benzylic C–H bonds in pharmaceutical candidates, we opt to first investigate the ring-opening deuteration protocol for the selective constructions of benzylic CDH moieties. In the deuteration of aromatic hydrocarbons, the relatively weakened benzylic C–H bonds can facilitate H/D exchange but have posed severe challenges for the selective monodeuteration^2^. We envisioned that the well-established β-bond cleavage of aromatic radical cations could render a facile approach to achieve benzylic monodeuteration, via selective ring openings of cyclic hydroaromatics and the intermediacy of a benzylic radical intermediate^35,57–62^. Through the use of efficient HAT catalysts, inexpensive and operationally handy deuteration agents such as CH_3_OD can be employed to enforce the desired deuterium atom transfer. Critically, the selective and irreversible C–C bond scission process would only allow the installation of one deuterium atom. Regarding the challenges raised by the high oxidation potential of aromatic hydrocarbons, we firstly looked into the development of highly oxidizing photocatalysts.

Organophosphorus compounds have been extensively investigated in organic electronics, optical sensing, and imaging applications^63–68^. Nevertheless, their unique photophysical and electrochemical properties remain largely underexploited in photoredox catalysis. Recently, we found that a bisphosphonium (BPP) compound easily prepared from the oxidative cyclization of 2,2′-*bis*(diphenylphosphino)-1,1′-binaphthyl (BINAP), can act as a strongly oxidizing organophotocatalyst to promote the intramolecular hydroetherification of alkenols. The remarkable capacity of this photoredox catalyst, including high oxidation potential (*E* ^*^_1/2_ = 2.17 V vs. SCE), strong absorption of visible light (λ_max_ = 413 nm), and long excited life time, has not been fully explored for synthetic transformations^69–72^.

Herein, we disclose a practical and selective monodeuteration protocol by bisphosphonium photocatalysis which can enable the facile constructions of benzylic CDH moieties from easily accessed materials. The photoexcitation and photoinduced electron transfer events of bisphosphonium catalyst are elu cidated in its premiere instance by spectroscopy experiments, paving the way for future applications.

## Results and discussion

### Reaction optimization

**Fig. 2:**
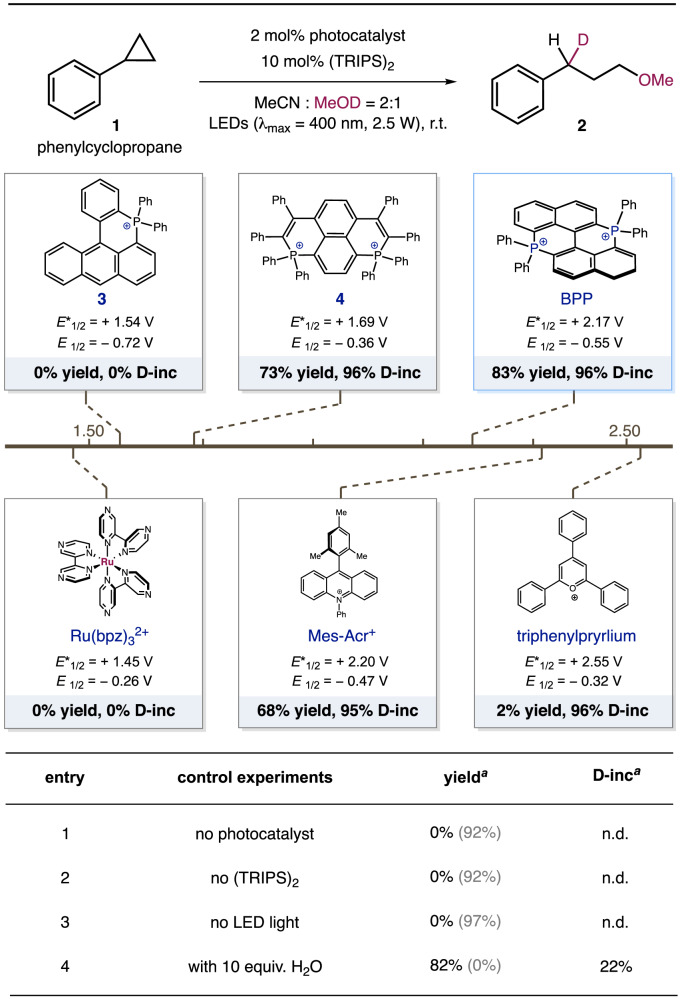
Reaction optimization and control experiments. Reactions were performed in a parallel reactor, with 0.2 mmol **1**, 2 mol% photocatalyst, 10 mol% (TRIPS)_2_, CH_3_CN (0.6 ml) and MeOD (0.3 ml). ^a^The yields were determined with GC-FID, the D-incorporation (D-inc) was determined by HR-MS. The recovery of **1** was presented in parentheses.

To validate our hypothesis, phenylcyclopropane (*E*_1/2_ = 1.7 V vs. SCE) was employed as the template substrate, readily available mono-deuteromethanol CH_3_OD was chosen as the deuteration reagent. In combination with commonly used atom transfer catalyst (TRIPS)_2_, a variety of oxidizing photocatalysts were evaluated under the irradiation of high intensive LED light (see supporting information for the detailed description of set-up for the parallel reactions). As depicted in Fig. 2, commonly utilized oxidizing photocatalysts Ru(bpz)_3_^2+^ and triphenylpryrlium were found ineffective, while mesitylacridinium photocatalyst could afford product **2** with 68% yield and 95% D-incorporation. Among the P-containing conjugated arenes evaluated, BPP photocatalyst rendered the optimal condition, delivering the monodeuterated product with high efficiency and selectivity (83% yield, 96% D-incorporation). A seemingly positive correlation between the oxidation capacity and catalytic efficiency can be found in this series of organophotocatalyst, as phosphonium salt **3** (*E* ^*^_1/2_ = 1.54 V vs. SCE) was found inactive and bisphosphapyrenium **4** (*E* ^*^_1/2_ = 1.69 V vs. SCE) enabled moderate efficiency (73% yield, 96% D-incorporation). Furthermore, control experiments have indicated the essential role of BPP photocatalyst, thiol catalyst and LED light. Notably, the control experiment with H_2_O in entry 4 has also revealed the critical importance of anhydrous conditions to achieve high deuteration selectivity.

### Selective monodeuteration of cyclic aromatic substrates

**Fig. 3:**
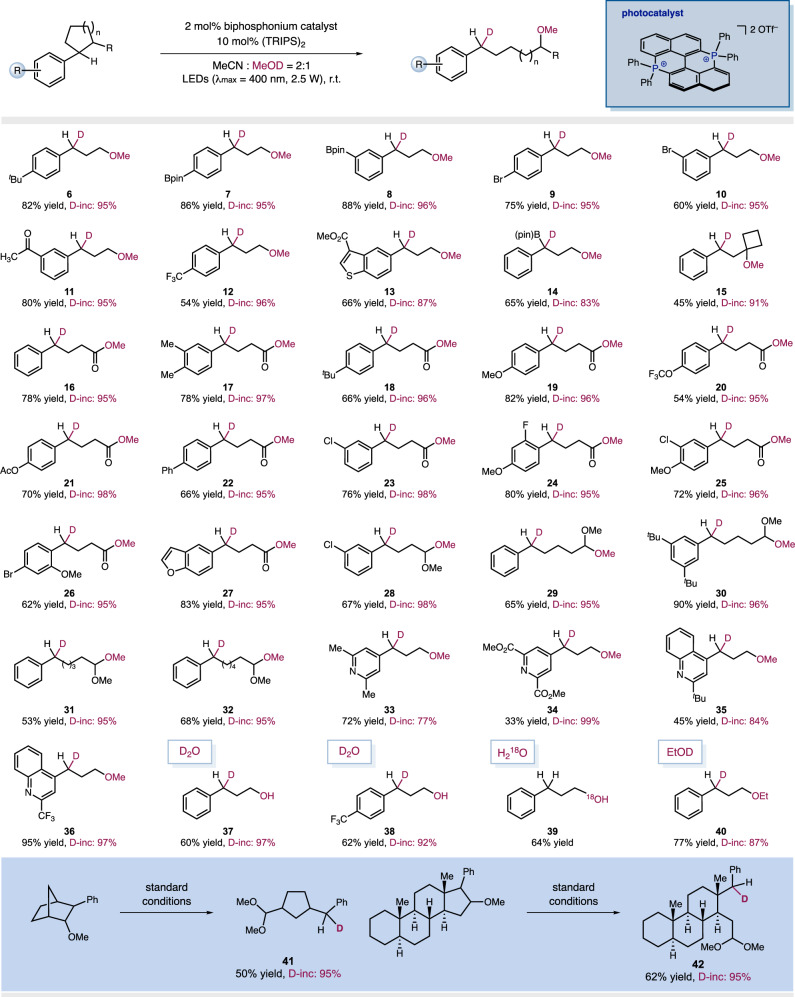
Reaction scope. Reactions were performed in a parallel reactor, with 0.2 mmol substrate, 2 mol% photocatalyst, 10 mol% (TRIPS)_2_, CH_3_CN (0.6 ml) and MeOD (0.3 ml). Deuterium incorporation was determined by HR-MS analysis.

With the optimal condition in hand, we next explored the scope of cyclic aromatic substrates and were delighted to find that this operationally simple and inexpensive catalytic system could be adapted to 3-membered to 7-membered cyclic starting materials. Importantly, a high degree of monodeuteration selectivity was achieved across the board, delivering the desired products with more than 95% D-incorporation. Even in the presence of weakened benzylic C–H bonds and acidic α-carbonyl C–H bonds which are prone to undergo H/D exchange to induce multiple deuterium incorporation, only the desired monodeuterated product was obtained. As shown in Fig. 3, this organophotocatalytic monodeuteration protocol can be successfully applied to a variety of substituted arylcyclopropanes, generating monodeuterated 3-methoxypropylbenzenes in high D-incorporation rate. Arylbornic ester (**7,** **8, 14**) and aryl bromide (**9,** **10,** **26**), commonly used functional handles in transition metal catalysis, could be well tolerated under the current photocatalytic condition. Owing to the high oxidation capacity of BPP catalyst, electron deficient arenes which are more challenging to activate in SET oxidations can be employed, although *p*-CF_3_ and *m*-Br substitutes rendered somewhat declined efficiencies. Regarding unsymmetrical arylcyclobutanones, the radical cation-mediated ring-opening process proved to be highly selective, as the C–C bond between the carbonyl and benzylic carbon terminus were selectively cleaved to generate monodeuterated 4-phenylbutanoates (**16**-**27**). For less strained 5-, 6-, 7-membered cyclic substrates, we noticed that this photocatalytic ring-opening deuteration can be effectively promoted by stabilizing the partially formed positive charge at the β-carbon terminus^73^. The introduction of a methoxy group at the β-carbon led to the facile cleavage of these less strained C–C bonds, rendering monodeuterated products with a distal hemiacetal functionality for further functionality manipulations (see Figs. S19–S23 for the conversions into amine, alcohol, alkene and tetrahydroquinoline moites). A variety of N-heterocyclic compounds, including pyridine and quinoline derivatives, have been effectively incorporated into monodeuterated products with yields ranging from excellent to moderate, without a significant impact on deuterium incorporation efficiency (**33**-**36**). Deuteration using D_2_O as the agent under standard conditions has yielded satisfactory outcomes (**37,** **38**). Furthermore, ^18^O labeling is readily accomplished with H_2_^18^O as the nucleophile under standard conditions (**39**). Importantly, the cyclic hydroaromatic moieties embedded in complex scaffolds such as steroid ring systems can be selectively cleaved, enabling the selective incorporation of benzylic CHD moieties. The implementation of a continuous-flow synthesis method has facilitated scaled-up production of **2**, achieving an 87% yield and a space-time yield of 17.4 g/L•h under optimized conditions, while significantly reducing the disulfide loading to 5 mol%.

### Spectroscopy experiments

**Fig. 4:**
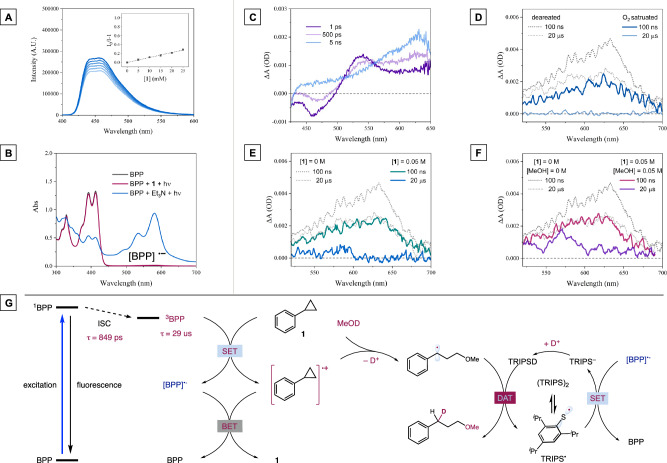
Mechanistic investigations and proposed reaction pathways. **A** Stern-Volmer quenching experiment. **B** Photolysis experiment. **C** Transient absorption spectra. **D** ESA decay signals in the presence of oxygen. **E** ESA decay signals in the presence of **1**. **F** ESA decay signals in the presence of **1** and MeOH. **G** proposed photoexcitation and monodeuteration pathways.

Spectroscopy experiments were then conducted to probe the critical photoinduced electron transfer (PET) event between phenylcyclopropane **1** and BPP catalyst. Unexpectedly, Stern–Volmer quenching studies revealed an insignificant fluorescence quenching effect of phenylcyclopropane even at relatively high concentrations (Fig. 4A), reminding us that the electron transfer between excited BPP and phenylcyclopropane might be sluggish. This was in striking contrast to the observation made in the same set of experiments with triethylamine as the reductant (see Fig. S4 for the quenching experiment with triethylamine). As demonstrated in Fig. 4B, the reduced BPP ([BPP]^**•–**^) generated from the PET process with triethylamine can be clearly identified in the 580 nm region in the UV-vis absorption spectrum, while the irradiation of the solution containing BPP and phenylcyclopropane did not cause any observable changes in the absorption spectrum. From the redox potential perspective, both PET processes are thermodynamically favorable, but only the PET process with triethylamine led to the net generation of [BPP]^**•–**^. Considering that the only difference lies in the oxidative nature of the resultant radical cations, we posited that highly oxidizing aromatic radical cations might result in an overwhelming back electron transfer, leading to declined efficiency^74,75^. These preliminary findings, contradicting the efficient ring-opening deuteration we obtained, promoted us to elucidate the PET events between BPP photocatalyst and the phenylcyclopropane substrate.

As the excitation pattern of bisphosphonium photocatalyst is currently unknown, we decided to first probe the photoexcitation process via ultrafast transient absorption (TA) spectroscopy. As depicted in Fig. 4C, the ultrafast TA spectra of BPP in acetonitrile solution (*c* = 0.5 mM) following 400 nm excitation (power intensity, 20 nJ per pulse) exhibit abundant excited state spectra features, including a ground state bleaching (GSB) at 420–430 nm, a simulated emission (SE) band at 430-500 nm, and two excited state absorption bands (ESA 1 and ESA 2) in the range of 500-650 nm (see Fig. S8 for details). The ESA 1 band at 540 nm shows single exponential decay of ~849 ps which is accompanied by recovery of the SE band at 466-nm region indicating the decay dynamics of the ESA 1 and SE bands originate from the same singlet excited state (S1 state). The SE negative signal coincides with the fluorescence emission spectrum of BPP measured by fluorescence spectroscopy, and we identify it as the fluorescence signal generated when the S1 state returns to the ground state after BPP excitation. The lifetime (τ_f_ = 819 ps measured by TCSPC) is consistent with the decay lifetime (τ_1_ = 849 ps) obtained by transient spectral dynamics analysis. The ESA 2 band at 625 nm showcases two exponential dynamics with a rise of 849 ps followed by an infinity-long lifetime decay. The rise component of 849 ps is reasonably ascribed to the intersystem crossing relaxation (ISC) time from the S1 state to the triplet state (T1) state. The latter component corresponds to the decay lifetime of T1 state.

To obtain a more complete dynamic process of the ESA 2 band, we carried out transient absorption spectroscopy in ns-μs timescale in the region of 525–700 nm. As shown in Fig. 4D, the ESA 2 band exhibits exponential decay with time constants of 29 μs. Monitoring whether the lifetime of the photoreaction system has a rapid reduction after the introduction of oxygen can be used to determine whether the triplet state exists. As demonstrated in Fig. 4D, oxygen can quickly quench 640 nm region signal, thus identifying the signal of ESA 2 band as the triplet signal generated by ISC after BPP excitation.

After the identification of the triplet state which is responsible for the desired electron transfer event, we next monitored the TA spectrum of BPP catalyst in the presence of phenylcyclopropane **1** (BPP: **1** = 1:100). With the addition of phenylcyclopropane, an accelerated decay of triplet BPP absorption can be observed (Fig. 4E). At 20 μs, the absorption centered of ESA 2 band at 625 nm returned to baseline, indicating a nearly complete consumption of triplet BPP by the SET with **1**. But in the 580 nm region, the characteristic absorption of [BPP]^**•–**^ was too weak to be distinguished. This peculiar observation could be explained by the fast and predominant back electron transfer between [BPP]^**•–**^ and radical cation of phenylcyclopropane [**1**^**•+**^] that has converted [BPP]^**•–**^ into ground state BPP. Thusly, no net electron transfer takes place and this gives rise to no [BPP]^**•–**^, in accordance with the observation made in the photolysis experiment. Nevertheless, in the presence of phenylcyclopropane and methanol, the characteristic absorption of [BPP]^**•–**^ can be clearly observed as the triple absorption returned to baseline (Fig. 4F). The nucleophilic attack of [**1**^**•+**^] with methanol generates of a more stable and less oxidizing benzylic radical through a ring-opening process. Thusly, the net formation of [BPP]^**•–**^ has indicated that the back-electron transfer event is suppressed by the nucleophilic attack of [**1**^**•+**^].

### Proposed reaction mechanism

Based on these findings, the photoinduced electron transfer processes in this ring-opening deuteration have been established and the proposed reaction pathway was depicted in Fig. 4G. Upon visible-light irradiation, a transient singlet BPP can be formed, most of which undergo a fast ISC process (τ_ISC_ = 849 ps) to generate a long-lived triplet state (τ_1_ = 29 us). The single electron oxidation of **1** by ^**3**^BPP is thermodynamically favorable and generates [**1**^**•+**^] and reduced BPP. Nevertheless, bimolecular back electron transfer from the resultant [**1**^**•+**^] to [BPP]^**•–**^ regenerates ground state catalyst, rendering a fully reversible PET process. The BET process could be suppressed by the well-established nucleophilic attack of [**1**^**•+**^] with methanol, forming a benzylic radical intermediate. A subsequent deuterium atom transfer mediated by TRIPSD would deliver the desired monodeuteration product. Lastly, a single electron transfer event would regenerate BPP and thiol catalysts.

In summary, we show a practical and selective monodeuteration protocol utilizing radical-mediated C–C bond scission and deuterium atom transfer processes. Under LED irradiation, in the presence of bisphosphonium photocatalyst, thiol catalyst, and CH_3_OD, easily accessible cyclic hydroaromatics can be efficiently converted into mono-deuterated products equipped with distal functionalities. The redox-neutral and operationally simple reaction conditions provides a synthetically appealing approach for the constructions of benzylic CDH moieties. Through spectroscopy experiments including transient absorptions, the photoexcitation and photoinduced electron transfer events of bisphosphonium catalyst are established, elucidating a peculiar back-electron transfer process which can be regulated by subsequent nucleophilic additions.

## Methods

### General procedure for batch reactions

An 8 mL vial was charged with substrates (1.0 equiv.), BPP (0.02 equiv.), (TRIPS)_2_ (0.1 equiv.) and 1 mL MeCN/MeOD (*v*/*v* = 2:1). The vial was sealed with a Teflon®-lined cap, the reaction mixture was degassed by argon sparging for 10 minutes. The mixture was then irradiated with LED (λ_max_ = 400 nm photon flux, 2.5 W) and stirred under irradiation at ambient temperature for 16 hours. After the reaction, the mixture was evaporated in vacuo and the residue was purified by flash chromatography.

## Supplementary information

Supplementary Information

Transparent Peer Review file

## Data Availability

Data are available in the manuscript and supplementary materials. Data supporting the findings of this manuscript are also available from the authors upon request.

## References

[1] Kop, S. et al. Recent developments for the deuterium and tritium labeling of organic molecules. *Chem. Rev.* **122**, 6634–6718 (2022). 35179363 10.1021/acs.chemrev.1c00795

[2] Prakash, G., Paul, N., Oliver, G. A., Werz, D. B. & Maiti, D. C–H deuteration of organic compounds and potential drug candidates. *Chem. Soc. Rev.* **51**, 3123–3163 (2022). 35320331 10.1039/d0cs01496f

[3] Li, N., Li, Y., Wu, X., Zhu, C. & Xie, J. Radical deuteration. *Chem. Soc. Rev.* **51**, 6291–6306 (2022). 35856093 10.1039/d1cs00907a

[4] Zhou, R., Ma, L., Yang, X. & Cao, J. Recent advances in visible-light photocatalytic deuteration reactions. *Org. Chem. Front.* **8**, 426–444 (2021).

[5] Atzrodt, J., Derdau, V., Kerr, W. J. & Reid, M. Deuterium- and tritium-labelled compounds: applications in the life sciences. *Angew. Chem. Int. Ed.* **57**, 1758–1784 (2018). 10.1002/anie.201704146 28815899

[6] Pirali, T., Serafini, M., Cargnin, S. & Genazzani, A. A. Applications of deuterium in medicinal chemistry. *J. Med. Chem.* **62**, 5276–5297 (2019). 30640460 10.1021/acs.jmedchem.8b01808

[7] Mutlib, A. E. Application of stable isotope-labeled compounds in metabolism and in metabolism-mediated toxicity studies. *Chem. Res. Toxicol.* **21**, 1672–1689 (2008). 18702535 10.1021/tx800139z

[8] Gómez-Gallego, M. & Sierra, M. A. Kinetic isotope effects in the study of organometallic reaction mechanisms. *Chem. Rev.* **111**, 4857–4963 (2011). 21545118 10.1021/cr100436k

[9] Simmons, E. M. & Hartwig, J. F. On the interpretation of deuterium kinetic isotope effects in C–H bond functionalizations by transition-metal complexes. *Angew. Chem. Int. Ed.* **51**, 3066–3072 (2012). 10.1002/anie.201107334 22392731

[10] Atzrodt, J., Derdau, V., Fey, T. & Zimmermann, J. The renaissance of H/D exchange. *Angew. Chem. Int. Ed.* **46**, 7744–7765 (2007). 10.1002/anie.200700039 17886815

[11] Kurita, T. et al. Efficient and convenient heterogeneous palladium-catalyzed regioselective deuteration at the benzylic position. *Chem. Eur. J.* **14**, 664–673 (2008). 17910018 10.1002/chem.200701147

[12] Khaskin, E. & Milstein, D. Simple and efficient catalytic reaction for the selective deuteration of alcohols. *ACS Catal.* **3**, 448–452 (2013).

[13] Kerr, W. J., Reid, M. & Tuttle, T. Iridium-catalyzed formyl-selective deuteration of aldehydes. *Angew. Chem. Int. Ed.* **56**, 7808–7812 (2017). 10.1002/anie.201702997 28510987

[14] Palmer, W. N. & Chirik, P. J. Cobalt-catalyzed stereoretentive hydrogen isotope exchange of C(sp3)–H Bonds. *ACS Catal.* **7**, 5674–5678 (2017). 29456876 10.1021/acscatal.7b02051 PMC5813831

[15] Pfeifer, V. et al. Palladium nanoparticles for the deuteration and tritiation of benzylic positions on complex molecules. *Angew. Chem. Int. Ed.* **60**, 26671–26676 (2021). 10.1002/anie.202109043 34424591

[16] Puleo, T. R., Strong, A. J. & Bandar, J. S. Catalytic α-selective deuteration of styrene derivatives. *J. Am. Chem. Soc.* **141**, 1467–1472 (2019). 30625273 10.1021/jacs.8b12874

[17] Li, W. et al. Scalable and selective deuteration of (hetero)arenes. *Nat. Chem.* **14**, 334–341 (2022). 35027706 10.1038/s41557-021-00846-4 PMC8898765

[18] Du, H.-Z. et al. Cesium amide-catalyzed selective deuteration of benzylic C-H bonds with D_2_ and application for tritiation of pharmaceuticals. *Angew. Chem. Int. Ed.* **62**, e202214461 (2023). 10.1002/anie.202214461 36289047

[19] Junk, T. & Catallo, W. J. Hydrogen isotope exchange reactions involving C–H (D, T) bonds. *Chem. Soc. Rev.* **26**, 401–406 (1997).

[20] Bhadra, P. K. et al. Enhancement of the properties of a drug by mono-deuteriation: reduction of acid-catalysed formation of a gut-motilide enol ether from 8-deuterio-erythromycin B. *Org. Biomol. Chem.* **14**, 6289–6296 (2016). 27273525 10.1039/c6ob00785f

[21] Loh, Y. Y. et al. Photoredox-catalyzed deuteration and tritiation of pharmaceutical compounds. *Science* **358**, 1182–1187 (2017). 29123019 10.1126/science.aap9674 PMC5907472

[22] Soulard, V., Villa, G., Vollmar, D. P. & Renaud, P. Radical Deuteration with D_2_O: catalysis and mechanistic insights. *J. Am. Chem. Soc.* **140**, 155–158 (2018). 29240406 10.1021/jacs.7b12105

[23] Zhang, M., Yuan, X.-A., Zhu, C. & Xie, J. Deoxygenative deuteration of carboxylic acids with D_2_O. *Angew. Chem. Int. Ed.* **58**, 312–316 (2019). 10.1002/anie.201811522 30352142

[24] Li, Y. et al. Organophotocatalytic selective deuterodehalogenation of aryl or alkyl chlorides. *Nat. Commun.* **12**, 2894 (2021). 34001911 10.1038/s41467-021-23255-0 PMC8129137

[25] Li, N. et al. A highly selective decarboxylative deuteration of carboxylic acids. *Chem. Sci.* **12**, 5505–5510 (2021). 34163771 10.1039/d1sc00528f PMC8179560

[26] Zhou, X., Yu, T. & Dong, G. Site-specific and degree-controlled alkyl deuteration via Cu-catalyzed redox-neutral deacylation. *J. Am. Chem. Soc.* **144**, 9570–9575 (2022). 35613457 10.1021/jacs.2c04382 PMC9486252

[27] Zhao, G., Yao, W., Mauro, J. N. & Ngai, M.-Y. Excited-state palladium-catalyzed 1,2-spin-center shift enables selective C-2 reduction, deuteration, and iodination of carbohydrates. *J. Am. Chem. Soc.* **143**, 1728–1734 (2021). 33465308 10.1021/jacs.0c11209 PMC7988686

[28] Zhang, Y., Ji, P., Dong, Y., Wei, Y. & Wang, W. Deuteration of formyl groups via a catalytic radical H/D exchange approach. *ACS Catal.* **10**, 2226–2230 (2020). 33623725 10.1021/acscatal.9b05300 PMC7899177

[29] Kuang, Y. et al. Visible light driven deuteration of formyl C–H and hydridic C(sp3)–H bonds in feedstock chemicals and pharmaceutical molecules. *Chem. Sci.* **11**, 8912–8918 (2020). 34123145 10.1039/d0sc02661a PMC8163369

[30] Shi, Q. et al. Visible-light mediated catalytic asymmetric radical deuteration at non-benzylic positions. *Nat. Commun*. **13** (2022). 10.1038/s41467-022-32238-8 PMC9343372 35915119

[31] Wang, L., Xia, Y., Derdau, V. & Studer, A. Remote site-selective radical C(sp3)−H monodeuteration of amides using D_2_O. *Angew. Chem. Int. Ed.* **60**, 18645–18650 (2021). 10.1002/anie.202104254 PMC8456965 34114304

[32] Li, N. et al. Highly selective single and multiple deuteration of unactivated C(sp3)-H bonds. *Nat. Commun.* **13**, 4224 (2022). 35869077 10.1038/s41467-022-31956-3 PMC9307835

[33] Yu, X.-Y., Chen, J.-R. & Xiao, W.-J. Visible light-driven radical-mediated C–C bond cleavage/functionalization in organic synthesis. *Chem. Rev.* **121**, 506–561 (2021). 32469528 10.1021/acs.chemrev.0c00030

[34] Fumagalli, G., Stanton, S. & Bower, J. F. Recent methodologies that exploit C–C single-bond cleavage of strained ring systems by transition metal complexes. *Chem. Rev.* **117**, 9404–9432 (2017). 28075115 10.1021/acs.chemrev.6b00599

[35] Baciocchi, E., Bietti, M. & Lanzalunga, O. Mechanistic aspects of β-bond-cleavage reactions of aromatic radical cations. *Acc. Chem. Res.* **33**, 243–251 (2000). 10775317 10.1021/ar980014y

[36] Chang, L., An, Q., Duan, L., Feng, K. & Zuo, Z. Alkoxy radicals see the light: new paradigms of photochemical synthesis. *Chem. Rev.* **122**, 2429–2486 (2022). 34613698 10.1021/acs.chemrev.1c00256

[37] Pitts, C. R., Ling, B., Snyder, J. A., Bragg, A. E. & Lectka, T. Aminofluorination of cyclopropanes: a multifold approach through a common, catalytically generated intermediate. *J. Am. Chem. Soc.* **138**, 6598–6609 (2016). 27136383 10.1021/jacs.6b02838

[38] Yayla, H. G., Wang, H., Tarantino, K. T., Orbe, H. S. & Knowles, R. R. Catalytic ring-opening of cyclic alcohols enabled by PCET activation of strong O–H bonds. *J. Am. Chem. Soc.* **138**, 10794–10797 (2016). 27515494 10.1021/jacs.6b06517 PMC5110324

[39] Guo, J.-J. et al. Photocatalytic C−C bond cleavage and amination of cycloalkanols by cerium(III) chloride complex. *Angew. Chem. Int. Ed.* **55**, 15319–15322 (2016). 10.1002/anie.201609035 27862775

[40] Wang, D., Mao, J. & Zhu, C. Visible light-promoted ring-opening functionalization of unstrained cycloalkanols via inert C–C bond scission. *Chem. Sci.* **9**, 5805–5809 (2018). 30079191 10.1039/c8sc01763h PMC6050598

[41] Hu, A. et al. Cerium-catalyzed formal cycloaddition of cycloalkanols with alkenes through dual photoexcitation. *J. Am. Chem. Soc.* **140**, 13580–13585 (2018). 30289250 10.1021/jacs.8b08781

[42] Petzold, D., Singh, P., Almqvist, F. & König, B. Visible-light-mediated synthesis of β-chloro ketones from aryl cyclopropanes. *Angew. Chem. Int. Ed.* **58**, 8577–8580 (2019). 10.1002/anie.201902473 30901148

[43] Zuo, Z., Daniliuc, C. G. & Studer, A. Cooperative NHC/photoredox catalyzed ring-opening of aryl cyclopropanes to 1-aroyloxylated-3-acylated alkanes. *Angew. Chem. Int. Ed.* **60**, 25252–25257 (2021). 10.1002/anie.202110304 PMC9298441 34580972

[44] Zuo, Z. & Studer, A. 1,3-Oxyalkynylation of aryl cyclopropanes with ethylnylbenziodoxolones using photoredox catalysis. *Org. Lett.* **24**, 949–954 (2022). 35023750 10.1021/acs.orglett.1c04319

[45] Xin, H., Duan, X.-H., Yang, M., Zhang, Y. & Guo, L.-N. Visible light-driven, copper-catalyzed aerobic oxidative cleavage of cycloalkanones. *J. Org. Chem.* **86**, 8263–8273 (2021). 34107678 10.1021/acs.joc.1c00708

[46] Chen, Y. G., Wang, X., He, X., An, Q. & Zuo, Z. W. Photocatalytic dehydroxymethylative arylation by synergisti cerium and nickel catalysis. *J. Am. Chem. Soc.* **143**, 4896–4902 (2021). 33756079 10.1021/jacs.1c00618

[47] Huang, L. et al. Bioinspired desaturation of alcohols enabled by photoredox proton-coupled electron transfer and cobalt dual catalysis. *Nat. Commun.* **13**, 809 (2022). 35145083 10.1038/s41467-022-28441-2 PMC8831637

[48] Wang, X., Li, Y. & Wu, X. Photoredox/cobalt dual catalysis dnabled regiospecific synthesis of distally unsaturated ketones with hydrogen evolution. *ACS Catal.* **12**, 3710–3718 (2022).

[49] Ge, L. et al. Photoredox-catalyzed C–C bond cleavage of cyclopropanes for the formation of C(sp3)–heteroatom bonds. *Nat. Commun.* **13**, 5938 (2022). 36209214 10.1038/s41467-022-33602-4 PMC9547854

[50] Ge, L. et al. Photoredox-catalyzed oxo-amination of aryl cyclopropanes. *Nat. Commun.* **10**, 4367 (2019). 31554813 10.1038/s41467-019-12403-2 PMC6761154

[51] Liu, H., Li, Y., Wang, D. X., Sun, M. M. & Feng, C. Visible-light-promoted regioselective 1,3-fluoroallylation of gem-difluorocyclopropanes. *Org. Lett.* **22**, 8681–8686 (2020). 33112624 10.1021/acs.orglett.0c03268

[52] Wang, Y. et al. Visible-light-promoted site-specific and diverse functionalization of a C(sp3)–C(sp3) bond adjacent to an arene. *ACS Catal.* **10**, 6603–6612 (2020).

[53] Xin, H., Duan, X. H., Liu, L. & Guo, L. N. Metal-free, visible-light-induced selective C-C bond cleavage of cycloalkanones with molecular oxygen. *Chem. Eur. J.* **26**, 11690–11694 (2020). 32557942 10.1002/chem.202001032

[54] Nguyen, T. V. T., Wodrich, M. D. & Waser, J. Substrate-controlled C-H or C-C alkynylation of cyclopropanes: generation of aryl radical cations by direct light activation of hypervalent iodine reagents. *Chem. Sci.* **13**, 12831–12839 (2022). 36519037 10.1039/d2sc04344k PMC9645386

[55] Xu, Y. et al. Stereoselective photoredox catalyzed (3+3) dipolar cycloaddition of nitrone with aryl cyclopropane. *Angew. Chem. Int. Ed. Engl.* **62**, e202310671 (2023). 37700683 10.1002/anie.202310671

[56] Zhao, Y. R. et al. Synthesis of alpha-difluoromethylene ethers via photoredox-induced hyperconjugative ring opening of gem-difluorocyclopropanes. *J. Org. Chem.* **88**, 3787–3793 (2023). 36827360 10.1021/acs.joc.2c03062

[57] Rao, V. R. & Hixson, S. S. Arylcyclopropane photochemistry. electron-transfer-mediated photochemical addition of methanol to arylcyclopropanes. *J. Am. Chem. Soc.* **101**, 6458–6459 (1979).

[58] Mizuno, K., Ichinose, N. & Otsuji, Y. Photochemistry of 9, 10-dicyanoanthracene-1, 2-diarylcyclopropane systems. Photocycloaddition and photoisomerization. *J. Org. Chem.* **57**, 1855–1860 (1992).

[59] Taily, I. M., Saha, D. & Banerjee, P. Arylcyclopropane yet in its infancy: the challenges and recent advances in its functionalization. *Org. Biomol. Chem.* **19**, 8627–8645 (2021). 34549770 10.1039/d1ob01432c

[60] Peng, P. et al. Electrochemical C−C bond cleavage of cyclopropanes towards the synthesis of 1,3-difunctionalized molecules. *Nat. Commun.* **12**, 3075 (2021). 34031421 10.1038/s41467-021-23401-8 PMC8144616

[61] Kolb, S. et al. Electrocatalytic activation of donor–acceptor cyclopropanes and cyclobutanes: an alternative C(sp3)−C(sp3) cleavage mode. *Angew. Chem. Int. Ed.* **60**, 15928–15934 (2021). 10.1002/anie.202101477 PMC8362004 33890714

[62] Liao, K. et al. Photoredox cleavage of a Csp3–Csp3 bond in aromatic hydrocarbons. *J. Am. Chem. Soc.* **145**, 12284–12292 (2023). 37216226 10.1021/jacs.3c02745

[63] Duffy, M. P., Delaunay, W., Bouit, P. A. & Hissler, M. π-Conjugated phospholes and their incorporation into devices: components with a great deal of potential. *Chem. Soc. Rev.* **45**, 5296–5310 (2016). 27220681 10.1039/c6cs00257a

[64] Jeon, S. O. & Lee, J. Y. Phosphine oxide derivatives for organic light emitting diodes. *J. Mater. Chem.* **22**, 4233–4243 (2012).

[65] Federmann, P. et al. P-protected diphosphadibenzo[a, e]pentalenes and their mono- and dicationic P-bridged ladder stilbenes. *Org. Lett.* **21**, 2033–2038 (2019). 30896181 10.1021/acs.orglett.9b00161

[66] Belyaev, A., Chou, P. T. & Koshevoy, I. O. Cationic organophosphorus chromophores: a diamond in the rough among ionic dyes. *Chem. Eur. J.* **27**, 537–552 (2020). 32492231 10.1002/chem.202001853 PMC7821147

[67] Delouche, T. et al. Luminescent molecular switches based on dicationic P-doped polycyclic aromatic hydrocarbons. *Mater. Adv.* **1**, 3369–3377 (2020).

[68] Regulska, E. & Romero-Nieto, C. Design of organophosphorus materials for organic electronics and bio-applications. *Mater. Today Chem*. **22** (2021).

[69] Cheng, H. et al. Bisphosphonium salt: an effective photocatalyst for the intramolecular hydroalkoxylation of olefins. *Sci. Bull.* **64**, 1896–1901 (2019). 10.1016/j.scib.2019.08.014 36659585

[70] Yang, Z., Chen, J. & Liao, S. Monophosphoniums as effective photoredox oganocatalysts for visible light-regulated cationic RAFT polymerization. *ACS Macro. Lett.* **11**, 1073–1078 (2022). 35984378 10.1021/acsmacrolett.2c00418

[71] Zhang, X., Jiang, Y., Ma, Q., Hu, S. & Liao, S. Metal-free cationic polymerization of vinyl ethers with strict temporal control by employing an organophotocatalyst. *J. Am. Chem. Soc.* **143**, 6357–6362 (2021). 33900068 10.1021/jacs.1c02500

[72] Ding, J. et al. Selective oxidation of benzylic alcohols via synergistic bisphosphonium and cobalt catalysis. *Chem. Commun.* **59**, 4055–4058 (2023). 10.1039/d3cc00532a 36929170

[73] Dinnocenzo, J. P., Zuilhof, H., Lieberman, D. R., Simpson, T. R. & McKechney, M. W. Three-electron SN_2_ reactions of arylcyclopropane cation radicals. 2. steric and electronic effects of substitution1. *J. Am. Chem. Soc.* **119**, 994–1004 (1997).

[74] Ohkubo, K., Kobayashi, T. & Fukuzumi, S. Direct oxygenation of benzene to phenol using quinolinium ions as homogeneous photocatalysts. *Angew. Chem. Int. Ed.* **50**, 8652–8655 (2011). 10.1002/anie.201102931 21805547

[75] Ohkubo, K., Fujimoto, A. & Fukuzumi, S. Photocatalytic monofluorination of benzene by fluoride via photoinduced electron transfer with 3-cyano-1-methylquinolinium. *J. Phys. Chem. A* **117**, 10719–10725 (2013). 24050618 10.1021/jp408315a

