# Supplementary material for: Selective monodeuteration enabled by bisphosphonium catalyzed ring opening processes
Source: Nat Commun. 2024 Oct 30;15:9366. doi: 10.1038/s41467-024-53728-x (PMC11526102; doi:10.1038/s41467-024-53728-x)
Supplement: Supplementary file 1 — Supplementary Information [file 41467_2024_53728_MOESM1_ESM.pdf]

Supplementary Information for Selective monodeuteration enabled by bisphosphonium catalyzed ring opening processes

Yuanli Xu [1†], Wenlong Chen [2†], Ruihua Pu [3], Jia Ding [2], Qing An [2], Yi Yang *[1], Weimin Liu *[3], Zhiwei Zuo *[2]

[1] *Innovation Center for Chenguang High Performance Fluorine Material, Key Laboratory of Green Chemistry of Sichuan Institutes of Higher Education, Sichuan University of Science and Engineering, Zigong, 643000, China*

[2] *State Key Laboratory of Organometallic Chemistry, Shanghai Institute of Organic Chemistry, Chinese Academy of Sciences, Shanghai 200032, China*

[3] *School of Physical Science and Technology, ShanghaiTech University, Shanghai 201210, China*

Contents

1. General information S3

2. Mechanistic experiments S5

3. Reaction optimization S21

4. Derivatization studies S23

5. General procedure S24

6. Experimental procedures and spectral characterization of the products S31

7. Experimental data for derivatives S52

8. Experimental data for starting materials. S56

9. Continuous-flow synthesis S60

10. Experimental data using various deuterated nucleophiles S62

11. Spectral data S65

12. Reference S131

1. General information.

1.1 Materials and Methods

Reagents were purchased from Aldrich, TCI, Energy Chemical and J&K. All reactions were carried out in an oven-dried glassware under an argon atmosphere unless otherwise noted. Chromatographic purification of products was accomplished by flash chromatography using silica gel. Thin-layer chromatography (TLC) was performed on Silicycle 250 mm silica gel F-254 plates. ^{1}HNMR and ^{13}C NMR spectra were recorded on a Bruker 500 (500 MHz and 126 MHz), Agilent 400 (400 MHz and 100 MHz) or Varian 400 (400 MHz and 100 MHz), and are internally referenced to the signal of the residual protonated solvent: $CDCl_3$, δ 7.26 and 77.16 ppm. Data for ^{1}H NMR are reported as follows: chemical shift (δ ppm), multiplicity (s = singlet, d = doublet, t = triplet, q = quartet, m = multiplet, br = broad), integration, coupling constant (Hz). ^{13}C spectra were reported as chemical shifts in ppm and multiplicity where appropriate. GC analyses were carried out on Agilent 7890B Infinity system. High Resolution Mass spectra were obtained from Thermo Fisher Q-Exactive High-resolution MS.

1.2 The batch reaction set-up

A customized photoreactor was used for parallel photoredox reactions to ensure the reaction efficiency and data reproducibility, in which up to 24 reactions can be performed simultaneously under identical conditions. The reaction vials placed in the water-cooled aluminum heat block were irradiated by the LED chips underneath. Through the effective cooling of the LED chips and reaction vails by the aluminum plate with continuous water flow, the heating effect of the high-power LEDs can be offset and the reaction temperature can be maintained at ambient temperature. Through this design, identical irradiation intensity and ambient temperature can be guaranteed for each reaction to ensure data consistency.

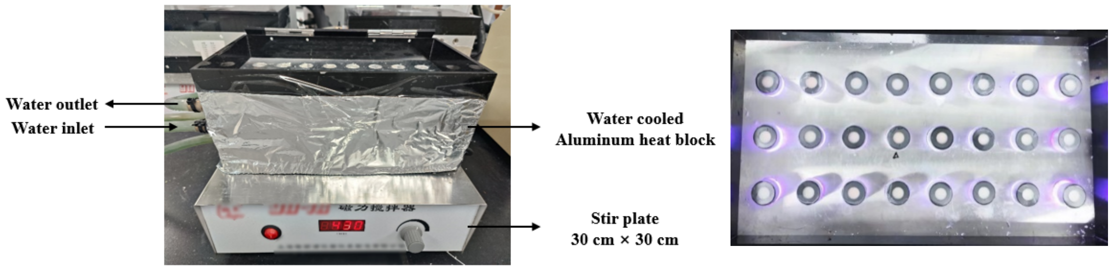

Figure S1. The batch reaction set-up. Left: main view of the photoreactor; right: top view of the photoreactor

2. Mechanistic experiments.

2.1 UV-vis absorption experiments

UV-vis absorption spectra were recorded using an Agilent Carry 5000 UV-vis spectrophotometer. Solutions of bisphosphonium salt (BPP) were prepared and introduced to a 1 cm path length quartz cuvette equipped with a Teflon® septum in the glovebox (all solutions were prepared in the dark). In a typical experiment, the appropriate amount of Et_3N or phenylcyclopropane (**1**) was added to a 0.1 mM solution of BPP in CH_3CN in a screw-top quartz cuvette in the glovebox and the absorption spectra were recorded on a UV-vis spectrophotometer in the dark or under LED (λ_{max} = 400 nm) irradiation for 10 seconds.

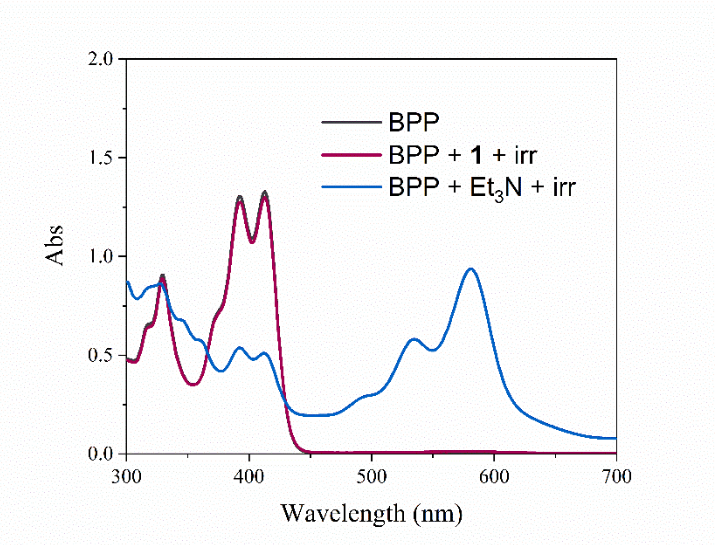

Figure S2. **Absorption spectra of a solution of BPP in CH_3CN.** [BPP] = 0.1 mM, [Et_3N] = 10 mM, [**1**] = 10 mM, Light source: LEDs (λ_{max} = 400 nm).

2.2 Luminescence quenching experiments (Stern-Volmer studies)

Emission intensities were recorded using a Fluorolog-3 luminescence spectrometer. Solutions of BPP were prepared and introduced to a 1 cm path length quartz cuvette equipped with a Teflon® septum in the glovebox (all solutions were prepared in the dark). In a typical experiment, the appropriate amount of phenylcyclopropane (**1**) was added to a 0.1 mM solution of BPP in CH_3CN in a screw-top quartz cuvette in the

glovebox. Then the sample was excited at a wavelength of 380 nm and the resulting emission was collected at 435 nm.

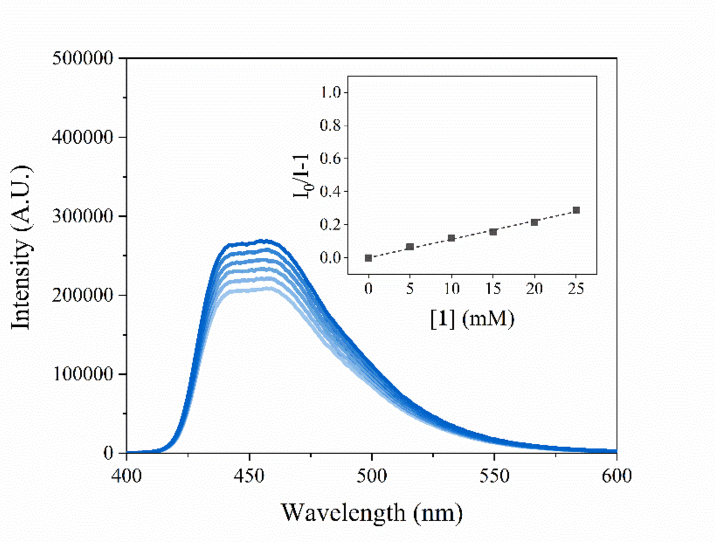

Figure S3. Stern-Volmer quenching experiments using phenylcyclopropane (1) as the quencher. [BPP] = 0.1 mM, [**1**] = 0–25 mM, excited wavelength: 380 nm, emission collected: 435 nm.

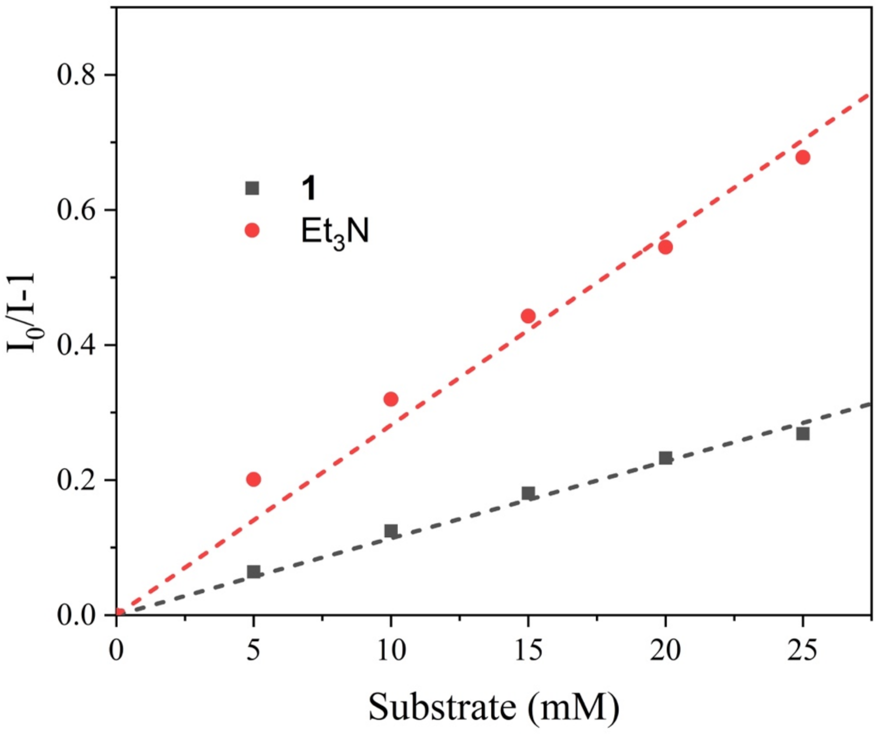

Figure S4. Stern-Volmer quenching experiments using triethylamine or phenylcyclopropane (1) as the quencher. [BPP] = 0.1 mM, [**1**] = 0–25 mM, [Et_3N] = 0–25 mM, excited wavelength:

380 nm, emission collected: 435 nm.

2.3 Fluorescence lifetime experiments

The fluorescence lifetimes were recorded using a DeltaFlex-011x time-resolved fluorescence spectrometer. A 0.1 mM solution of BPP was prepared and introduced to a 1 cm path length quartz cuvette equipped with a Teflon® septum in the glovebox (all solutions were prepared in the dark). In a typical experiment, the sample was excited at a wavelength of 367 nm and the fluorescence lifetime of the sample was recorded at 450 nm.

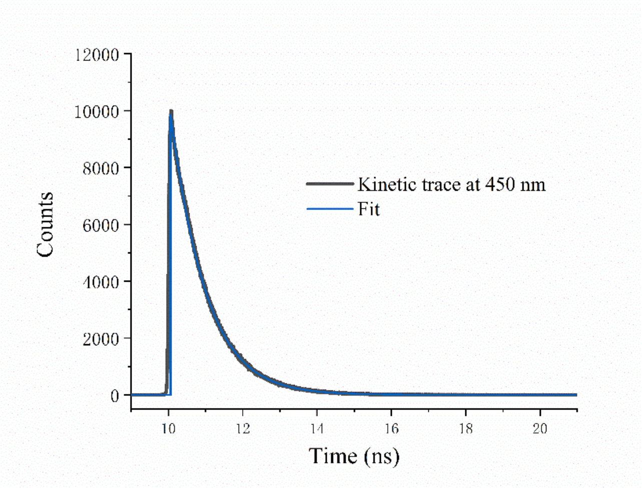

Figure S5. Time-resolved emission intensity decay of BPP in acetonitrile shown in black trace. The decay data was collected at 450 nm upon 367 nm excitation. A double exponential fit from 9 ns to 21 ns is given as blue lines, affording τ = 819 ps.

2.4 Transient absorption experiments

For measurements, the 800 nm fundamental pulses were generated from a Ti: Sapphire laser system (Coherent, Astrella, 35 fs, 7 mJ/pulse, 1 kHz repetition rate). A 400 nm pulse was used as the actinic pump, which was generated by 800 nm fundamental pulses via optical parametric amplifier (OPerA Solo, Coherent Inc.).

Femtosecond transient absorption (TA) spectra were collected using a commercial femtosecond transient absorption spectrometer (Helios fire, Ultrafast System). A broadband super continuum white light with a wavelength range from 320–650 nm

serves as the probe pulse by focusing the fundamental beam into a CaF_2 crystal plate and the instrument response function (IRF) of this whole system is about 120 fs. Femtosecond-resolved TA spectra were recorded under 400 nm excitation with power intensity of 10 μW (20 nJ per pulse). Pump power was measured with an optical power sensor (Thorlabs).

EOS-TA spectra in ns-μs region were collected using a commercial transient absorption spectrometer (EOS, Ultrafast System). A broadband super-continuum white light with wavelength ranges from ~320 nm to ~900 nm serves as the probe pulse, which was obtained through a sub-ns white light laser. IRF of the EOS system is determined to be ~5100 ps. EOS TA spectra were recorded under 400 nm excitation with power intensity of 50 W (100 nJ per pulse). Pump power was measured with an optical power sensor (Thorlabs). Pump power was measured with an optical power sensor (Thorlabs).

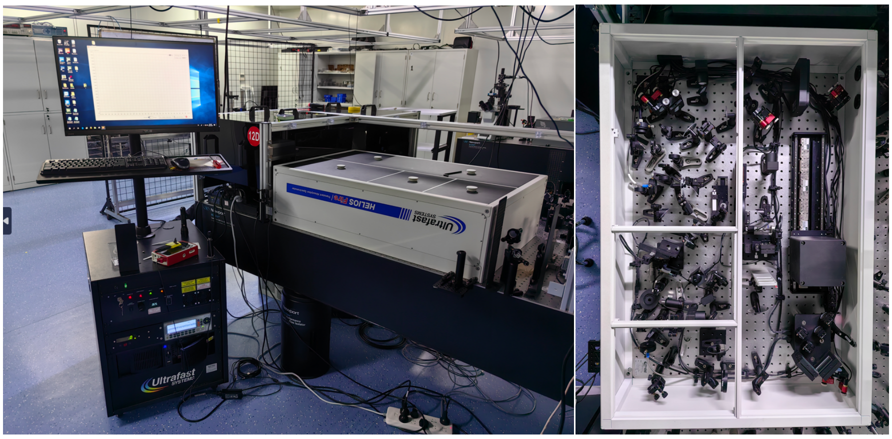

Figure S6. **Set-up for transient absorption investigations.** Left: main view of EOS-TA spectrometer; right: top view of EOS-TA spectrometer.

Kinetic fitting at single wavelengths was performed using Surface Explorer. All spectra were corrected for chirp and an average of 18 background spectra were subtracted in Surface Explorer. Background and chirp corrected spectra were imported to Surface Explorer.

Ultrafast transient absorption experiments were carried out using the solution of BPP ([BPP] = 0.5 mM) in anhydrous CH_3CN (through deoxygenation before use) in

0.2 cm path length quartz cuvettes with a Teflon®-coated magnetic stirrer. Before each measurement, the solution was filtered by a 0.22 μm syringe filter to avoid the influence of scattering on signal.

In order to gain insights into the reaction mechanism of BPP under photoexcitation, we first studied the photoexcitation process via ultrafast transient absorption (TA) spectroscopy.

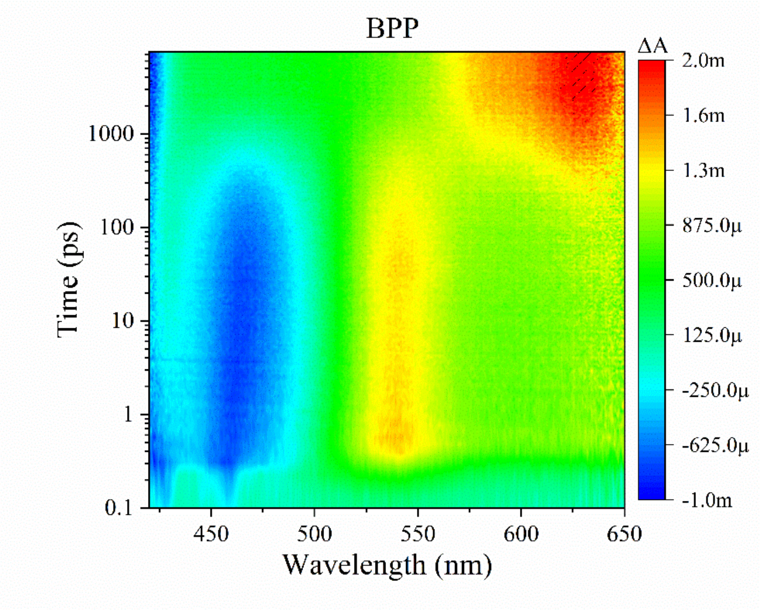

Figure S7. 2D-TA spectra of BPP in MeCN under 400 nm excitation in sub-picosecond to picosecond region. [BPP] = 0.5 mM. λ = 400 nm; light intensity: 20 nJ per pulse.

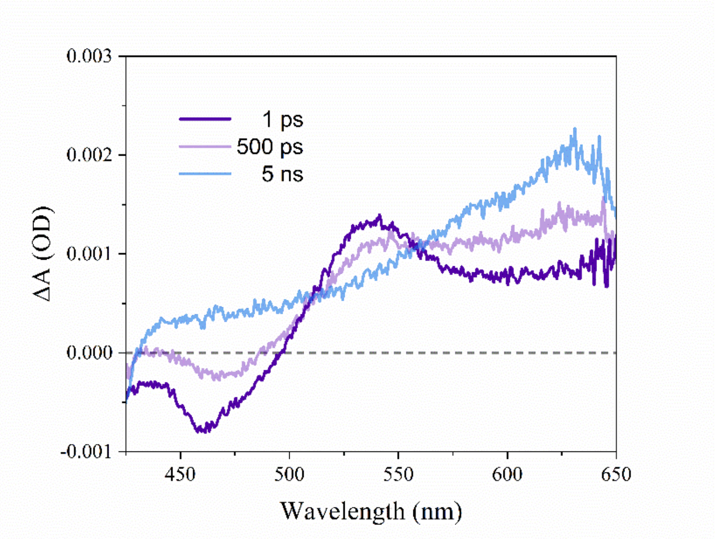

Figure S8. Transient absorption spectra of BPP in CH_3CN at different delay times following 400 nm excitation. [BPP] = 0.5 mM. λ = 400 nm; light intensity: 20 nJ per pulse.

We first measured the femtosecond transient spectrum of BPP, which mainly consists of a ground state bleaching (**GSB**) at 420–430 nm, a simulated emission (**SE**) at 430–500 nm and two excited state absorption (**ESA 1** and **ESA 2**) bands at 500–650 nm. Notably, as **ESA 1** (500–580nm) decays, **ESA 2** centered at 625 nm begins to be generated.

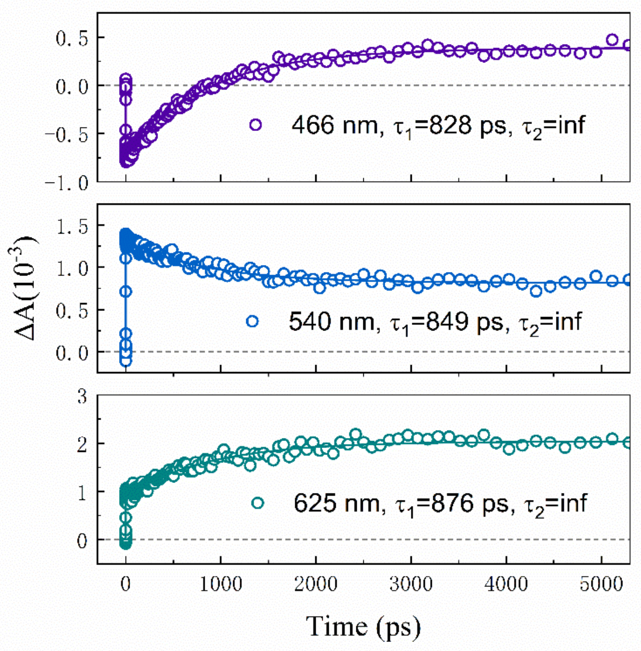

Figure S9. Transient absorption kinetic traces of BPP in CH_3CN at probe wavelength of 466 nm, 540 nm and 625 nm following 400 nm excitation (20 nJ per pulse). The fitting results are shown in solid lines.

The **ESA 1** band at 540 nm shows single exponential decay of ~849 ps which is accompanied by recovery of the **SE** band at 466 nm region indicating the decay dynamics of the **ESA 1** and **SE** bands originate from the same singlet excited state (**S1** state). The **SE** negative signal coincides with the fluorescence emission spectrum of BPP measured by fluorescence spectroscopy, and we identify it as the fluorescence signal generated when the **S1** state returns to the ground state after BPP excitation. The

lifetime (τ =819 ps measured by TCSPC) is consistent with the decay life-time (τ = 828 ps) obtained by transient spectral dynamics analysis.

The **ESA 2** band centered at 625 nm showcases two exponential dynamics with a rise of 876 ps followed by an infinity-long lifetime decay. The rise component of 876 ps is reasonably ascribed to the intersystem crossing relaxation (ISC) time from the S1 state to the triplet state (**T1**).

In order to obtain a more complete dynamic process of **ESA 2** band, we conducted nanosecond transient absorption experiments and focused on observing the changes in the the region of 525–700 nm (centered at 625 nm). Notably, oxygen can quickly quench 640 nm region signal, indicating that the signal in this band is a triplet signal generated by ISC after BPP excitation. The rising process of this signal observed in the femtosecond experiment corresponds to the inter system transition process (τ =876 ps).

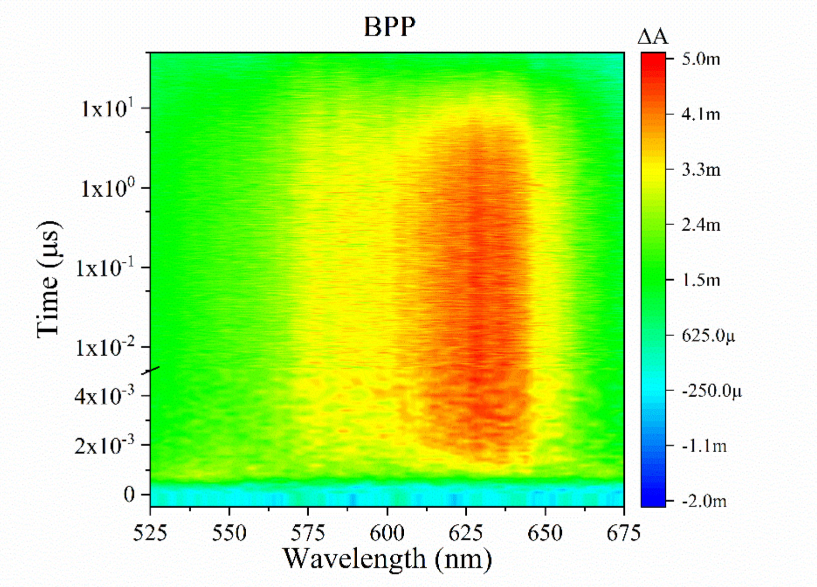

Figure S10. **2D-TA spectra of BPP in MeCN under 400 nm excitation in nanosecond to microsecond region.** [BPP] = 0.5 mM. λ = 400 nm, light intensity: 100 nJ per pulse.

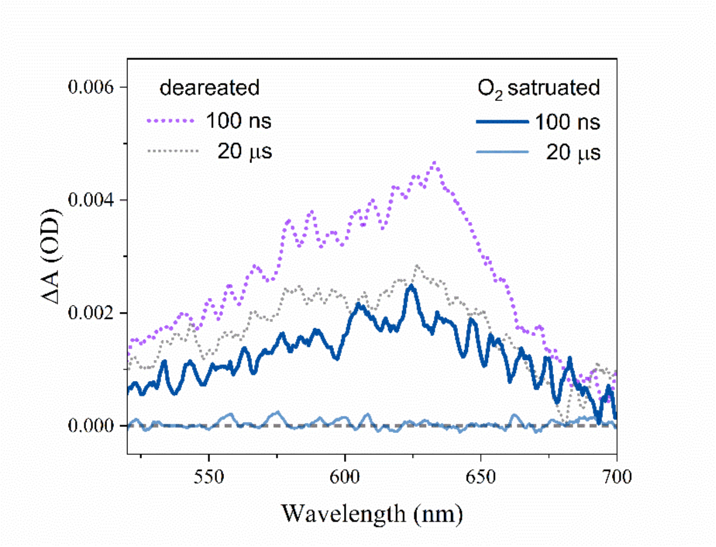

Figure S11. Transient absorption spectra of BPP in the absence or presence of O_2 in CH_3CN at different delay times following 400 nm excitation. [BPP] = 0.5 mM. λ = 400 nm; light intensity: 20 nJ per pulse.

The consistent dynamics of the decay of fluorescence signal, the rise of the triplet signal, and the decay of **ESA 1** centered at 540 nm allowed us to ultimately identify **ESA 1** as a singlet state signal after BPP excitation (τ = 849 ps).

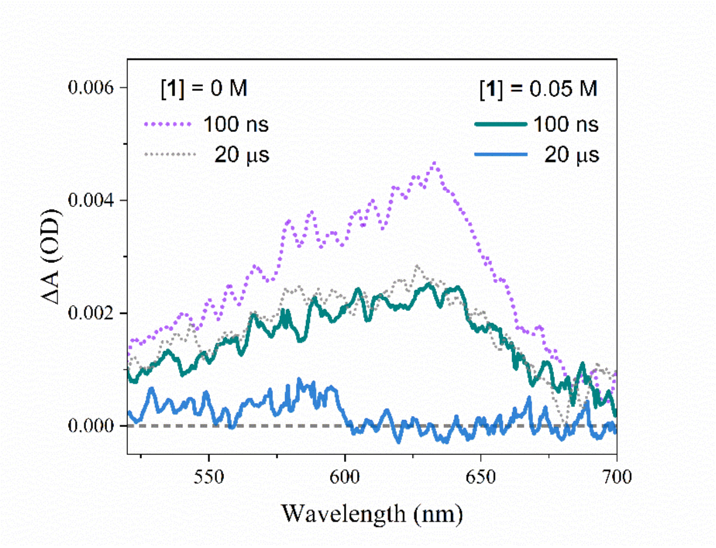

Figure S12. Transient absorption spectra of BPP in the absence or presence of phenylcyclopropane (1) in CH_3CN at different delay times following 400 nm excitation. [BPP] = 0.5 mM. λ = 400 nm; light intensity: 20 nJ per pulse.

After the identification of the triplet state which is responsible for the desired electron transfer event, we next monitored the TA spectrum of BPP catalyst in the presence of phenylcyclopropane **1** (BPP : **1** = 1:100). We found that the **ESA 2** centered at 625 nm decayed rapidly after the addition of phenylcyclopropane, indicating a nearly complete consumption of triplet BPP species by the SET with **1**, but there was a weak signal at about 580 nm that did not return to zero. This peculiar observation could be explained by the predominant back electron transfer that has converted $[BPP]^{\bullet-}$ into its ground state. Thusly, no net electron transfer takes place and this gives rise to no $[BPP]^{\bullet-}$, in accordance with the observation made in the photolysis experiment.

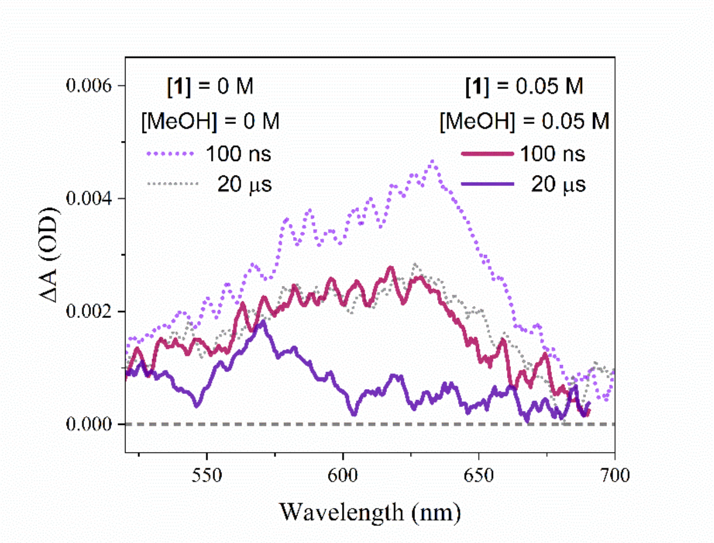

Figure S13. Transient absorption spectra of BPP in the absence or presence of phenylcyclopropane (1) and MeOH in CH_3CN at different delay times following 400 nm excitation. [BPP] = 0.5 mM. λ = 400 nm; light intensity: 20 nJ per pulse.

Nevertheless, in the presence of phenylcyclopropane and methanol, the characteristic absorption of $[BPP]^{\bullet-}$ can be clearly observed as the triple absorption returned to base-line. The net formation of $[BPP]^{\bullet-}$ indicates that the back-electron transfer has been suppressed by the nucleophilic attack of $[\mathbf{1}^{\bullet+}]$ which was converted into a more stable and less oxidizing benzylic radical through a ring-opening process.

2.3 Kinetic experiment

2.3.1 Parallel kinetic isotope effect (KIE) experiments using alcohol as the nucleophiles

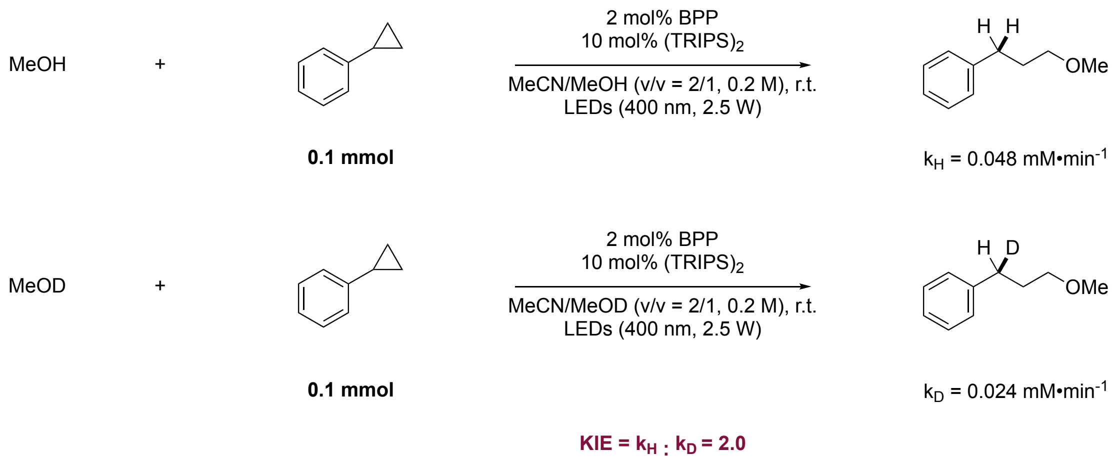

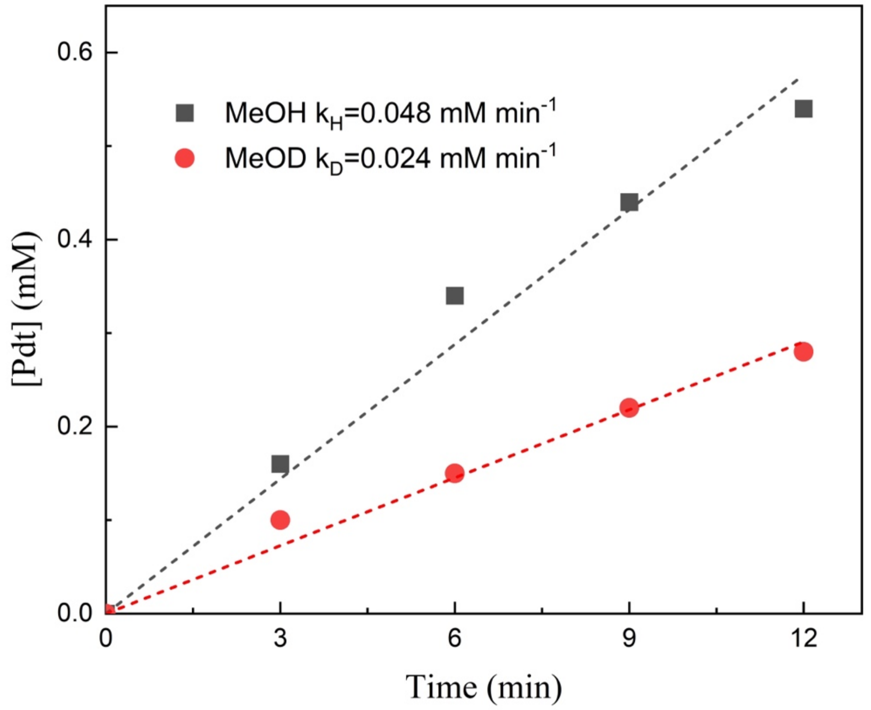

Figure S14. The parallel KIE experiment with MeOH and MeOD. k_H = 0.048 mM min^{-1}, k_D = 0.024 mM min^{-1}.

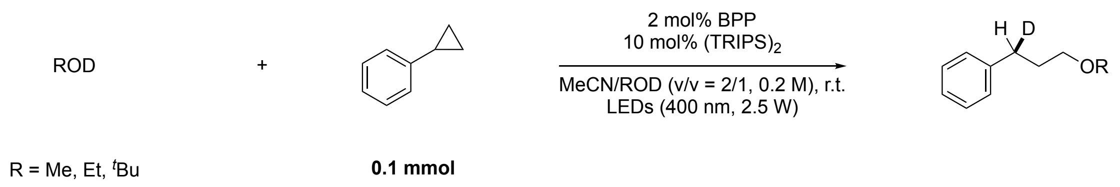

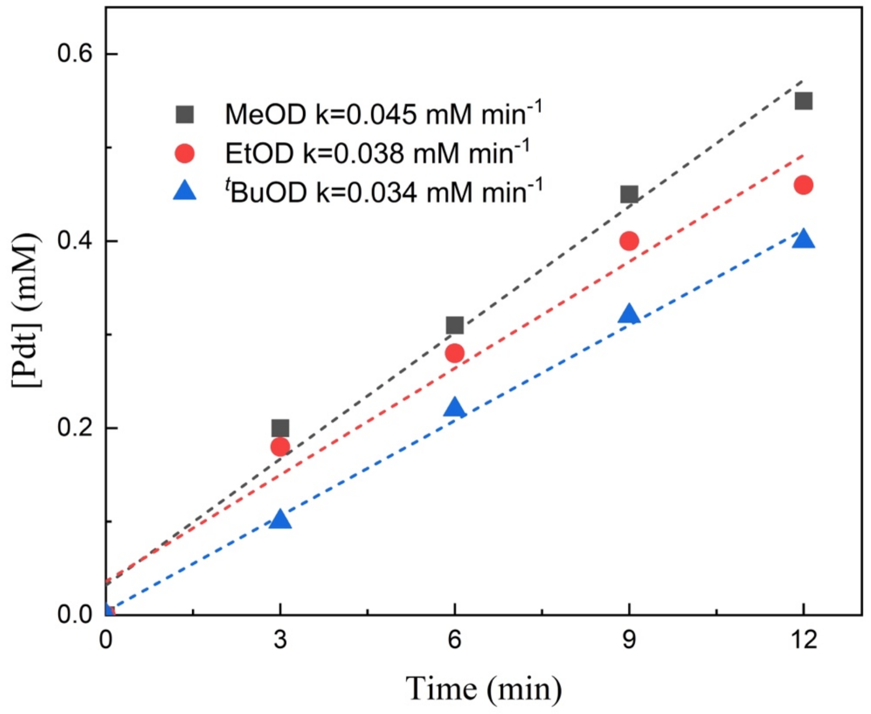

Figure S15. Initial rate for selective monodeuteration of phenylcyclopropane with ROD (R = Me, Et or *t*Bu). K_{MeOD} = 0.045 mM min^{-1}, k_{EtOD} = 0.038 mM min^{-1}, $k_{t\text{-}BuOD}$ = 0.034 mM min^{-1}.

An 8 mL vial was charged with phenylcyclopropane (0.1 mmol, 1.0 equiv.), BPP (0.002 mmol, 0.02 equiv.) and bis(2,4,6-triisopropylphenyl) disulfide ((TRIPS)$_2$, 0.01 mmol, 0.1 equiv.) in 0.5 mL MeCN/MeOH (v/v = 2/1) under N_2 atmosphere, and PhOMe was added to the reaction mixture as an internal standard. At each designated time interval, the solution was taken from the vial with a micro syringe (25 μL). The quantification of the products was determined by gas chromatography.

An 8 mL vial was charged with phenylcyclopropane (0.1 mmol, 1.0 equiv.), BPP (0.002 mmol, 0.02 equiv.) and (TRIPS)$_2$ (0.01 mmol, 0.1 equiv.) in 0.5 mL MeCN/ROD (v/v = 2/1) (R=Me, Et, or *t*Bu) under N_2 atmosphere, and PhOMe was added to the reaction mixture as an internal standard. At each designated time interval, the solution

was taken from the vial with a micro syringe (25 μL). The quantification of the products was determined by gas chromatography.

2.3.2 Competitive KIE experiments using alcohol as nucleophile

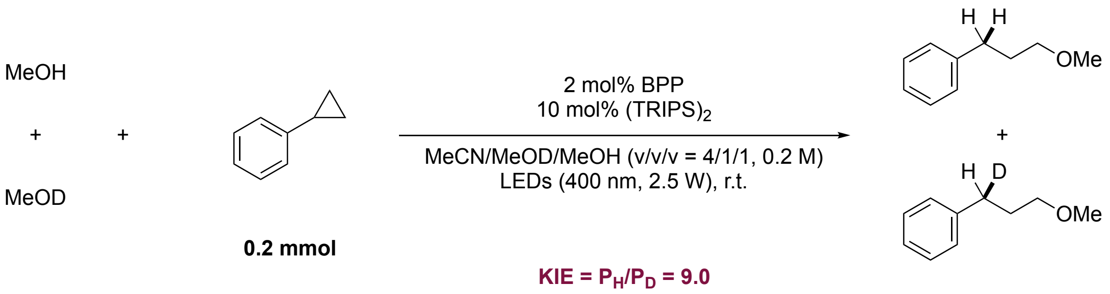

An 8 mL vial was charged with MeOH (167 μL), MeOD (167 μL), phenylcyclopropane (0.2 mmol, 1.0 equiv.), BPP (0.004 mmol, 0.02 equiv.) and $(TRIPS)_2$ (0.02 mmol, 0.1 equiv.) in MeCN (665 μL) under N_2 atmosphere. The solution then stirred at ambient temperature under the irradiation of LEDs (λ_{max} = 400 nm photon flux, 2.5 W) for 9 min. Then the pure product was obtained by flash column chromatography on silica gel (5% ethyl acetate in petroleum) to afford corresponding products. ^{1}H NMR spectra for the isolated product indicated that the ratio is P_H/P_D = 9.0.

2.3.3 Parallel KIE experiments using water as nucleophile

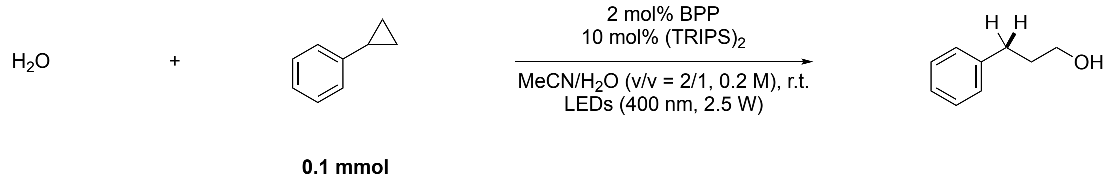

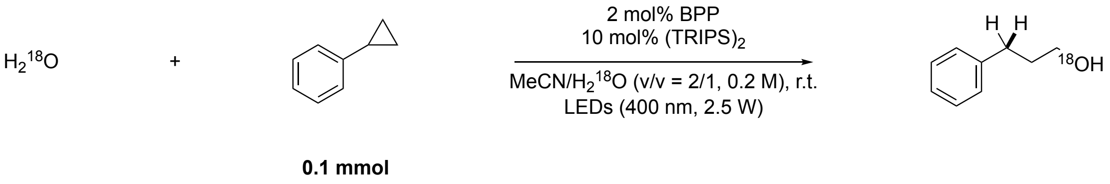

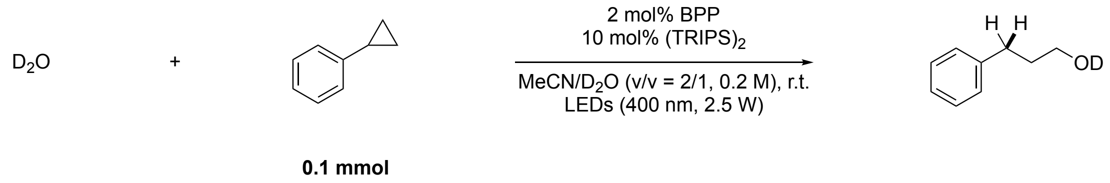

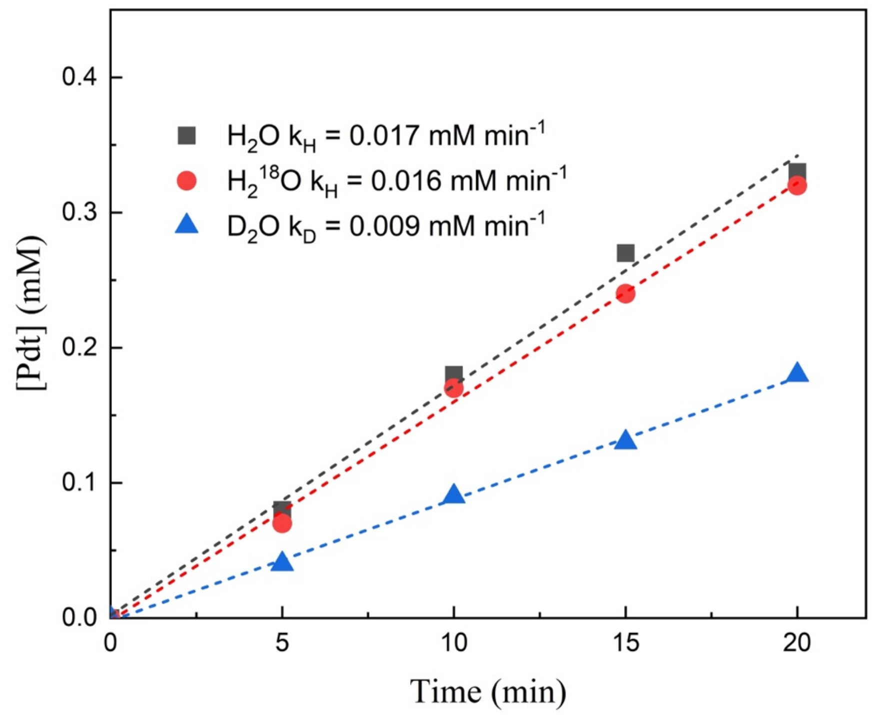

Figure S16. Initial rate for ring opening of phenylcyclopropane with H_2O, $H_2{}^{18}O$ and D_2O. $k_H(H_2O)$ = 0.017 mM min^{-1}, $k_H(H_2{}^{18}O)$ = 0.016 mM min^{-1}, $k_D(D_2O)$ = 0.009 mM min^{-1}.

An 8 mL vial was charged with phenylcyclopropane (0.1 mmol, 1.0 equiv.), BPP (0.002 mmol, 0.02 equiv.) and $(TRIPS)_2$ (0.01 mmol, 0.1 equiv.) in 0.5 mL MeCN/H_2O (v/v = 2/1) under N_2 atmosphere, and PhOMe was added to the reaction mixture as an internal standard. At each designated time interval, the solution was taken from the vial with a micro syringe (25 μL). The quantification of the products was determined by gas chromatography.

An 8 mL vial was charged with phenylcyclopropane (0.1 mmol, 1.0 equiv.), BPP (0.002 mmol, 0.02 equiv.) and $(TRIPS)_2$ (0.01 mmol, 0.1 equiv.) in 0.5 mL MeCN/$H_2{}^{18}O$ (v/v = 2/1) under N_2 atmosphere, and PhOMe was added to the reaction mixture as an internal standard. At each designated time interval, the solution was taken from the vial with a micro syringe (25 μL). The quantification of the products was determined by gas chromatography.

An 8 mL vial was charged with phenylcyclopropane (0.1 mmol, 1.0 equiv.), BPP (0.002 mmol, 0.02 equiv.) and $(TRIPS)_2$ (0.01 mmol, 0.1 equiv.) in 0.5 mL $MeCN/D_2O$ (v/v = 2/1) under N_2 atmosphere, and PhOMe was added to the reaction mixture as an internal standard. At each designated time interval, the solution was taken from the vial with a micro syringe (25 μL). The quantification of the products was determined by gas chromatography.

2.3.4 Competitive KIE experiments using water as nucleophile

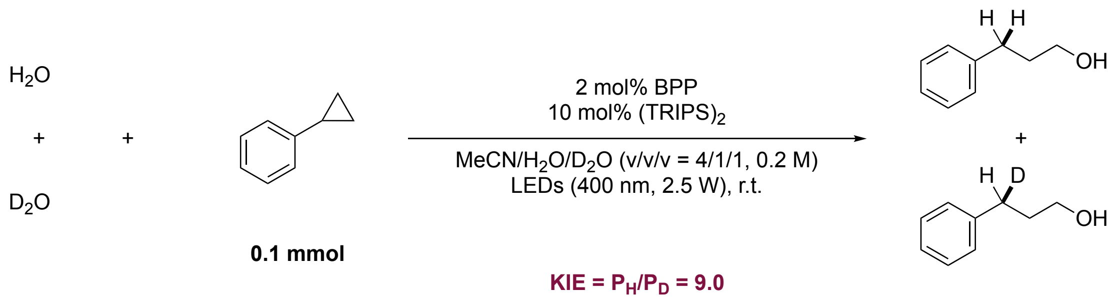

An 8 mL vial was charged with H_2O (84 μL), $H_2{}^{18}O$ (84 μL), phenylcyclopropane (0.1 mmol, 1.0 equiv.), BPP (0.002 mmol, 0.02 equiv.) and $(TRIPS)_2$ (0.01 mmol, 0.1 equiv.) in MeCN (332 μL) under N_2 atmosphere. The solution then stirred at ambient temperature under the irradiation of LEDS (λ_{max} = 400 nm photon flux, 2.5 W) for 20 min. Then the pure product was obtained by flash column chromatography on silica gel (5% ethyl acetate in petroleum) to afford corresponding products. ^1H NMR spectra for the isolated product indicated that the ratio is P_H/P_D = 9.0.

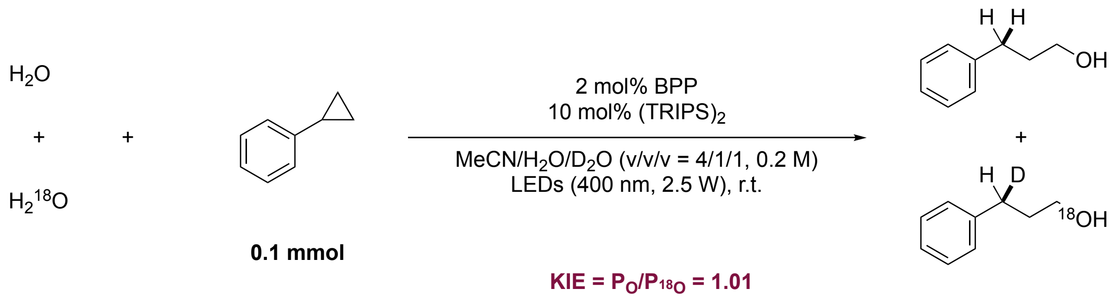

An 8 mL vial was charged with H_2O (84 μL), $H_2{}^{18}O$ (84 μL), phenylcyclopropane (0.1 mmol, 1.0 equiv.), BPP (0.002 mmol, 0.02 equiv.) and $(TRIPS)_2$ (0.01 mmol, 0.1 equiv.) in MeCN (332 μL) under N_2 atmosphere. The solution then stirred at ambient

temperature under the irradiation of LEDS (λ_{max} = 400 nm photon flux, 2.5 W) for 20 min. GCMS indicated that the ratio of product is $P_O/P_{^{18}O}$ = 1.01.

2.3.5 Kinetic experiments using staring materials with different ring size

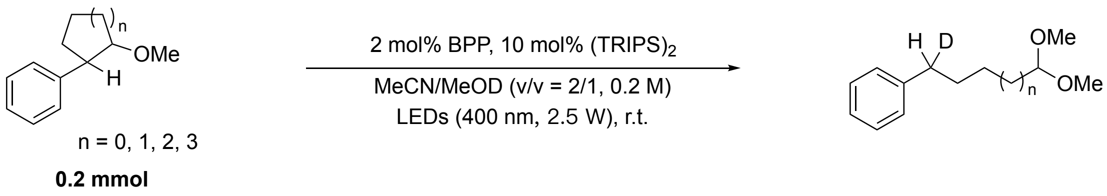

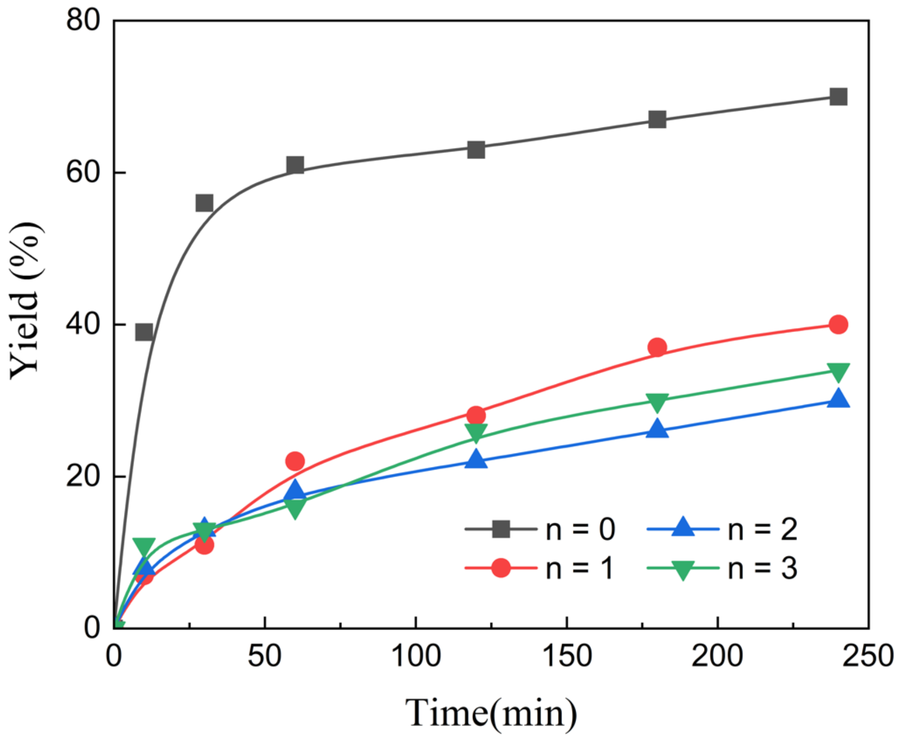

Figure S17. Reaction profile of (2-methoxycyclobutyl)benzene, (2-methoxycyclopentyl)benzene, (2-methoxycyclohexyl)benzene, and 1-methoxy-2-phenylcycloheptane with MeOD. Reactions were performed in a parallel reactor, with 0.2 mmol **1**, 2 mol% photocatalyst, 10 mol% $(TRIPS)_2$, CH_3CN (0.6 ml) and MeOD (0.3 ml).

An 8 mL vial was charged with (2-methoxycyclobutyl)benzene (0.2 mmol, 1.0 equiv.), BPP (0.004 mmol, 0.02 equiv.) and $(TRIPS)_2$ (0.02 mmol, 0.1 equiv.) in 1.0 mL MeCN/MeOD (v/v = 2/1) under N_2 atmosphere, and PhOMe was added to the

reaction mixture as an internal standard. At each designated time interval, the solution was taken from the vial with a micro syringe (25 μL). The quantification of the products was determined by gas chromatography.

An 8 mL vial was charged with (2-methoxycyclopentyl)benzene (0.2 mmol, 1.0 equiv.), BPP (0.004 mmol, 0.02 equiv.) and $(TRIPS)_2$ (0.02 mmol, 0.1 equiv.) in 1.0 mL MeCN/MeOD (v/v = 2/1) under N_2 atmosphere, and PhOMe was added to the reaction mixture as an internal standard. At each designated time interval, the solution was taken from the vial with a micro syringe (25 μL). The quantification of the products was determined by gas chromatography.

An 8 mL vial was charged with (2-methoxycyclohexyl)benzene (0.2 mmol, 1.0 equiv.), BPP (0.004 mmol, 0.02 equiv.) and $(TRIPS)_2$ (0.02 mmol, 0.1 equiv.) in 1.0 mL MeCN/MeOD (v/v = 2/1) under N_2 atmosphere, and PhOMe was added to the reaction mixture as an internal standard. At each designated time interval, the solution was taken from the vial with a micro syringe (25 μL). The quantification of the products was determined by gas chromatography.

An 8 mL vial was charged with 1-methoxy-2-phenylcycloheptane (0.2 mmol, 1.0 equiv.), BPP (0.004 mmol, 0.02 equiv.) and $(TRIPS)_2$ (0.02 mmol, 0.1 equiv.) in 1.0 mL MeCN/MeOD (v/v = 2/1) under N_2 atmosphere, and PhOMe was added to the reaction mixture as an internal standard. At each designated time interval, the solution was taken from the vial with a micro syringe (25 μL). The quantification of the products was determined by gas chromatography.

3. Reaction optimization

Table S1. Evaluation of MeOD loading

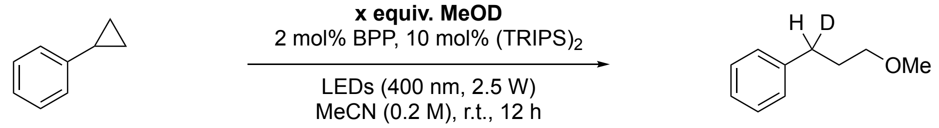

1 2

Entry	Amount of MeOD	Yield[b]	D-inc[c]
1	5 equiv.	16%	95%
2	10 equiv.	39%	95%
3	20 equiv.	61%	96%
**4**	**MeCN/MeOD (v/v = 2:1)**	**80%**	**96%**
5	MeCN/MeOD (v/v = 1:1)	76%	95%

[a]Reaction conditions: A solution of phenylcyclopropane **1** (0.2 mmol), BPP (2 mol%), $(TRIPS)_2$ (10 mol%) and MeOD in MeCN was irradiated with 2.5 W 400 nm LEDs for 12 hours at room temperature under argon atmosphere. [b]Yield was determined by GC-FID analysis of the crude mixture using PhOMe as an internal standard. [c]D-inc was determined by GC-MS.

Table S2. Evaluation of solvent

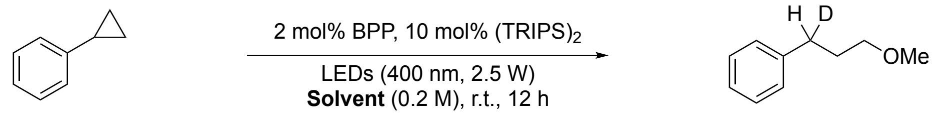

1 2

Entry	Solvent	Yield[b]	D-inc[c]
**1**	**MeCN/MeOD (v/v= 2:1)**	**80%**	**95%**
2	DCM/MeOD (v/v= 2:1)	49%	95%
3	$CHCl_3$/MeOD (v/v= 2:1)	35%	96%
4	TCEE/MeOD (v/v= 2:1)	24%	96%
5	EA/MeOD (v/v= 2:1)	44%	95%
6	Toluene/MeOD (v/v= 2:1)	52%	95%
7	$PhCF_3$/MeOD (v/v= 2:1)	63%	96%
8	^{n}Hexane/MeOD (v/v= 2:1)	63%	95%

[a]Reaction conditions: A solution of phenylcyclopropane **1** (0.2 mmol), BPP (2 mol%), and $(TRIPS)_2$ (10 mol%) in 1 mL solvent was irradiated with 2.5 W 400 nm LEDs for 12 hours at room temperature under argon atmosphere. [b]Yield was determined by GC-FID analysis of the crude mixture using PhOMe as an internal standard. [c]D-inc was determined by GC-MS. DCM = dichloromethane, TCEE = trichloroethylene.

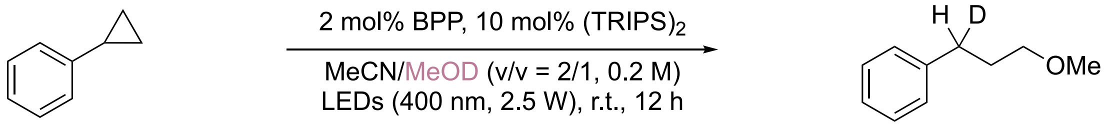

1 2

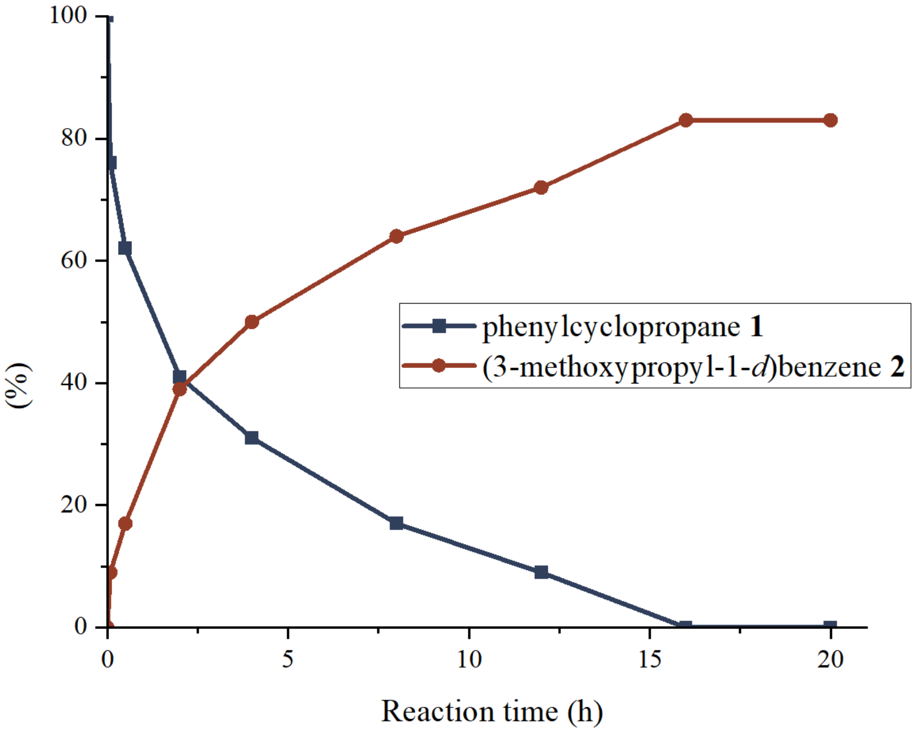

Figure S18. Reaction time profiles of phenylcyclopropane 1 and (3-methoxypropyl-1-*d*)benzene 2. Reactions were performed in a parallel reactor, with 0.2 mmol **1**, 2 mol% photocatalyst, 10 mol% $(TRIPS)_2$, CH_3CN (0.6 ml) and MeOD (0.3 ml).

4. Derivatization study

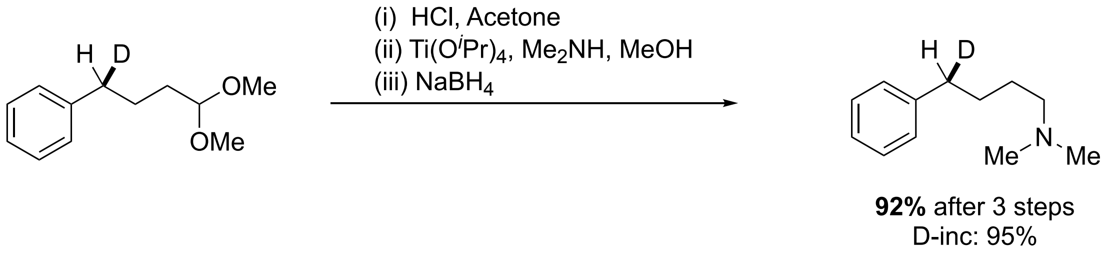

Figure S19. Conversion to amine. Conditions: (i) HCl, acetone; (ii) Ti(O^{i}Pr)$_4$, Me$_2$NH, MeOH; (iii) NaBH$_4$.

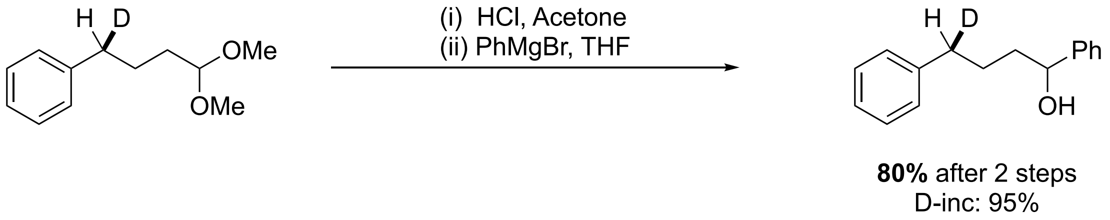

Figure S20. Conversion to alcohol. Conditions: (i) HCl, acetone; (ii) PhMgBr, THF.

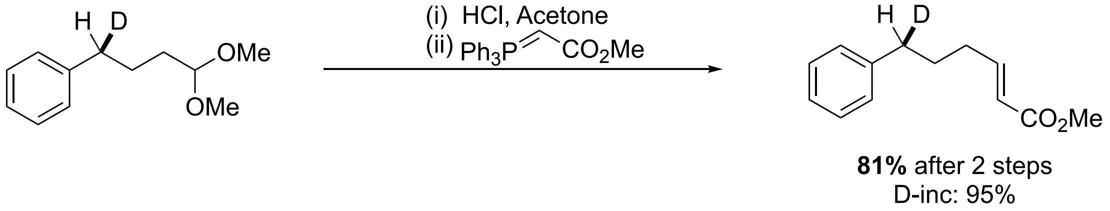

Figure S21. Conversion to alkene. Conditions: (i) HCl, acetone; (ii) Wittig salt.

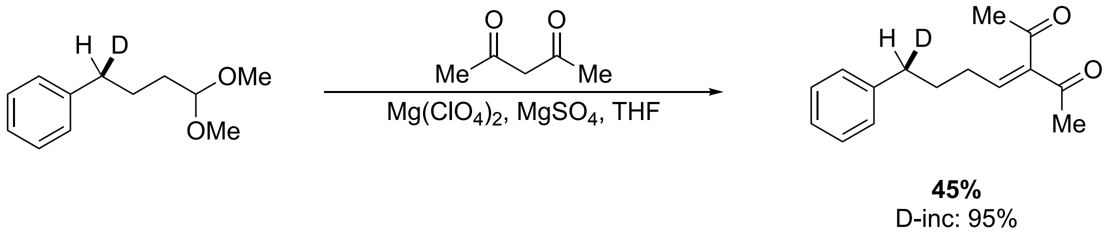

Figure S22. Conversion to unsaturated ketone. Conditions: acetylacetone, Mg(ClO$_4$)$_2$, MgSO$_4$, THF.

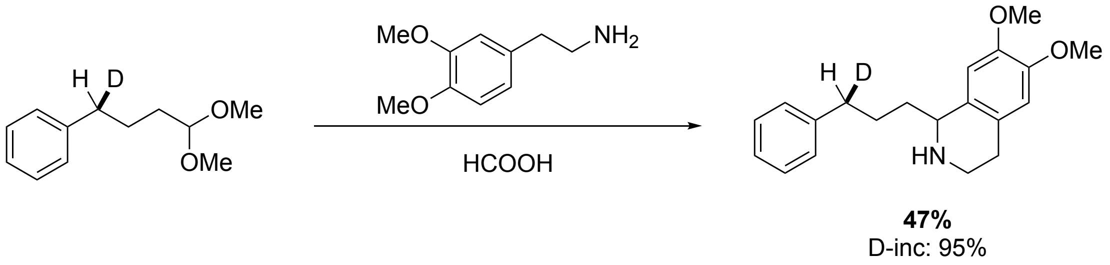

Figure S23. Conversion to tetrahydroquinoline derivative. Conditions: 2-(3,4-dimethoxyphenyl)ethan-1-amine, formic acid.

5. General procedure

5.1 General procedure for batch reactions

An 8 mL vial was charged with substrates (1.0 equiv.), BPP (0.02 equiv.), $(TRIPS)_2$ (0.1 equiv.) and 1 mL MeCN/MeOD (v/v = 2/1). The vial was sealed with a Teflon®-lined cap, the reaction mixture was degassed by argon sparging for 10 minutes. The mixture was then irradiated with LEDS (λ_{max} = 400 nm photon flux, 2.5 W) and stirred under irradiation at ambient temperature for 16 hours. After the reaction, the mixture was evaporated *in vacuo* and the residue was purified by flash chromatography.

5.2 General procedure for preparation of the bisphosphonium salts

BPP: A solution of $Cu(OTf)_2$ (3.62 g, 10.0 mmol, 1.0 equiv.) and 1.1'-Binaphthyl-2.2'-diphemyl phosphine (6.23 g, 10.0 mmol, 1.0 equiv.) in acetonitrile (200 mL) was stirred at room temperature for 15 minutes. The solvent was then evaporated, and the crude mixture was purified by silica gel chromatography (20% acetone in dichloromethane). The yellow band eluted was collected. The resulting solution was concentrated under *vacuo* to a volume of 10 mL. Diethyl ether diffused slowly into the concentrated solution, resulting in the formation of a microcrystalline solid. The solid was washed with Et_2O (2 × 20 mL), and afforded a yellow crystal (1.96 g, 20% yield). **^{1}H NMR (400 MHz, CD_3COCD_3):** δ 8.93–8.90 (m, 2H), 8.80–8.74 (m, 4H), 8.36–8.29 (m, 4H), 8.00–7.94 (m, 12H), 7.82–7.77 (m, 8H). **^{13}C NMR (100 MHz, CD_3COCD_3):** δ 140.5 (d, *J* = 8.0 Hz), 139.7, 137.8 (d, *J* = 9.0 Hz), 136.9 (d, *J* = 2.0 Hz), 136.0 (d, *J* = 13.0 Hz), 135.5 (d, *J* = 13.0 Hz), 133.7 (dd, *J* = 12.0, 5.0 Hz), 131.7 (d, *J* = 14.0 Hz), 131.4–130.9 (m), 130.1 (dd, *J* = 11.0, 6.0 Hz), 120.3, 119.7, 117.6, 117.1, 113.6, 113.0. **^{19}F NMR (376 MHz, CD_3COCD_3):** δ −78.91. **^{31}P NMR (162 MHz, CD_3COCD_3):** δ 2.82.

Bisphosphonium catalyst 3[1]: A solution of $Cu(OTf)_2$ (434 mg, 1.2 mmol, 0.6 equiv.) and triphenylphosphine (877 mg, 2.0 mmol, 1.0 equiv.) in acetonitrile (20 mL) was

stirred at room temperature for 3 h. Afterward, the solvent was evaporated, and the crude mixture was purified by silica gel chromatography (20% acetone in ldichloromethane). The solvents were removed, and a yellow powder (400 mg, 34% yield) was obtained. (The substituted triphenylphosphines were synthesized according to literature procedures[2].) **^{1}H NMR (400 MHz, $CDCl_3$):** δ 8.77 (s, 1H), 8.62–8.56 (m, 2H), 8.33–8.30 (m, 1H), 8.18–8.16 (m, 1H), 8.11–8.05 (m, 1H), 7.95–7.88 (m, 3H), 7.75–7.61 (m, 13H). **^{13}C NMR (100 MHz, CD_2Cl_2):** δ 140.2 (d, J = 5.0 Hz), 138.0 (d, J = 3.0 Hz), 137.9 (d, J = 8.0 Hz), 136.3, 138.0 (d, J = 3.0 Hz), 137.9 (d, J = 8.0 Hz), 136.3, 135.1 (d, J = 2.0 Hz), 134.7 (d, J = 9.0 Hz), 134.5, 134.3 (d, J = 10.0 Hz), 132.9, 131.6 (d, J = 2.0 Hz), 131.1 (d, J = 13.0 Hz), 130.5 (d, J = 8.0 Hz), 129.8 (d, J = 13.0 Hz), 129.7 (d, J = 53.0 Hz), 127.8, 127.4 (d, J = 11.0 Hz), 126.0 (d, J = 6.0 Hz), 125.8 (d, J = 15.0 Hz), 115.1 (d, J = 89.0 Hz), 111.1 (d, J = 93.0 Hz),. **^{19}F NMR (376 MHz, $CDCl_3$):** δ −78.03. **^{31}P NMR (162 MHz, $CDCl_3$):** δ 4.36.

Bisphosphonium catalyst 4[3]: A solution of 1,4-bis(diphenylphosphino)naphtalene (160 mg, 0.32 mmol,1 equiv.), diphenylacetylene (140 mg, 0.78 mmol, 2.4 equiv.) and $Cu(OTf)_2$ (468 mg, 1.30 mmol, 4 equiv.) in degassed acetonitrile (32 mL) was heated at 100 °C overnight. The solvent was then evaporated and the crude mixture was purified on silica gel chromatography (20% acetone in dichloromethane) to yield a yellow powder (100 mg, 54% yield). **^{1}H NMR (400 MHz, $CDCl_3$):** δ 8.42–8.38 (m, 2H), 7.89–7.83 (m, 8H), 7.77–7.74 (m, 4H), 7.69–7.65 (m, 8H), 7.63 (s, 2H), 7.32–7.31 (m, 4H), 7.25–7.19 (m, 6H), 7.05–7.02 (m, 2H), 6.95 (t, J = 7.6 Hz, 4H), 6.73–6.71 (m, 4H). **^{13}C NMR (100 MHz, CD_2Cl_2):** δ 160.2–160.1 (m), 136.8 (s), 136.7 (d, $J_{C\text{-}P}$ = 14 Hz), 136.4–136.1 (m), 135.2 (d, J = 11 Hz), 133.2 (d, J = 15 Hz), 132.5 (s), 131.3 (d, J = 4 Hz), 131.2 (d, J = 14 Hz), 130.5 (s), 129.9–129.8 (m), 129.4 (s), 129.4 (s), 129.0 (s), 129.0 (s), 122.3 (d, J = 79 Hz), 118.9 (d, J = 74 Hz), 118.4 (d, J = 92 Hz). **^{19}F NMR (376 MHz, $CDCl_3$):** δ −78.24. **^{31}P NMR (162 MHz, $CDCl_3$):** δ 1.13.

5.3 General procedure for starting materials

General procedure for the preparation of arylcyclopropane[4]

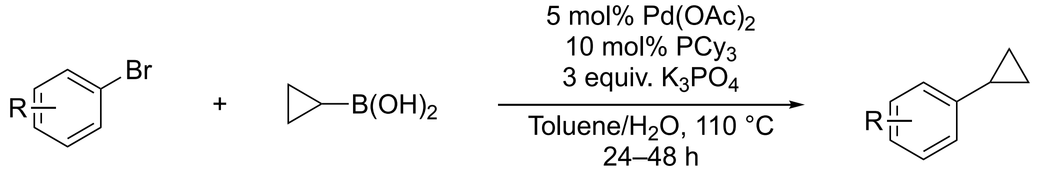

A flame-dried three-neck round bottom flask was equipped with a stir bar and a reflux condenser. Substituted bromobenzene (9.0 mmol, 1 equiv.), cyclopropylboronic acid (1.00 g, 11.6 mmol, 1.29 equiv.), K_3PO_4 (5.72 g, 27.0 mmol, 3 equiv.), toluene (40 mL), and H_2O (2 mL) were added to the flask under N_2 atmosphere. The mixture was stirred, and a 10 wt.% solution of $P(Cy)_3$ in toluene (1.3 mL, 0.9 mmol, 0.1 equiv.) was added via a syringe, followed by the addition of $Pd(OAc)_2$ (0.1 g, 0.45 mmol, 0.05 equiv.). The mixture was heated at 110 °C overnight. After cooling to room temperature, the reaction mixture was diluted with water and extracted with ethyl acetate. The organic phases were combined, dried over anhydrous Na_2SO_4, filtered, and concentrated in *vacuo* to obtain the crude product, which was then purified using flash column chromatography.

General procedure for the preparation of arylcyclobutanone[5]

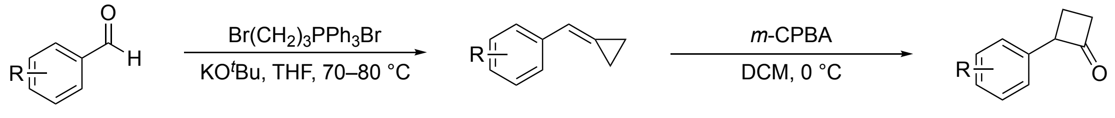

S1 **S2** **S3**

Under an argon atmosphere, the solution of ^{t}BuOK (3 equiv.) in THF (1 M) was added via a cannula to a suspension of 1-bromopropyl-3-triphenylphoshonium bromide (1.5 equiv.) in THF. Then the reaction mixture was heated at 70 °C for 1 hour, followed by the addition of aldehyde (**S1**). After heating the reaction mixture at 80 °C for an additional 8 hours, it was then cooled down to room temperature. The reaction mixture was then poured into a large Erlenmeyer containing pentane (10 mL/mmol), and the precipitates were filtered. The filtrate was concentrated and the residue was purified using a silica gel chromatography to obtain the **S2**.

m-CPBA (1.1 equiv.) was added in portions to a solution of cyclopropylidenes (**S2**) (1 equiv.) in CH_2Cl_2 (0.5 M) at 0 °C. After the consumption of **S2** (monitored by TLC,

around 30 minutes), camphor sulfonic acid (0.05 equiv.) was added to ensure complete conversion of epoxides to cyclobutanones. The reaction mixture was stirred for an additional 30 minutes before being quenched with a solution of 10% Na_2SO_3 and 2% KI. After vigorous stirring for 20 minutes, the mixture was extracted three times with Et_2O. The combined organic layers were washed three times with a saturated $NaHCO_3$ solution, dried over anhydrous Na_2SO_4, filtered and concentrated on a rotavapor. The residue was then purified using a silica gel chromatography to give the corresponding cyclobutanones **S3**.

General Procedure for the Preparation of Arylcyclobutylether[5, 6]

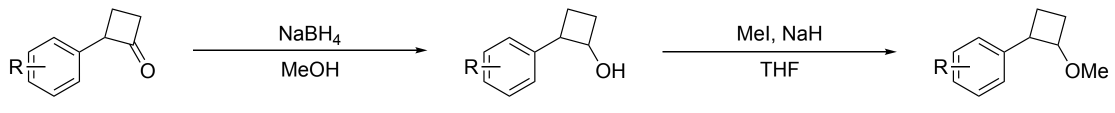

Cyclobutanone **S3** (1 equiv.) was dissolved in MeOH (0.33 M) and $NaBH_4$ (1 equiv.) was added gradually at 0 °C over a period of 10 minutes. After stirring the reaction mixture at room temperature for 1 hour, it was then quenched with saturated NH_4Cl water solution (3 mL per mmol of **S3**). The mixture was extracted with ethyl acetate for three times and the combined organic layers were washed with brine. The organic phase was dried over anhydrous Na_2SO_4, filtered, concentrated under *vacuo*. And the residue was purified on a silica gel column chromatography to give cyclobutanol **S4**.

To the solution of **S4** (1.0 equiv.) in anhydrous THF (10 mL), sodium hydride (3.0 equiv.) was added at 0 °C, followed by the dropwise addition of iodomethane (2.0 equiv.). The reaction mixture was allowed to warm to room temperature and stir for 30 minutes. Afterward, the reaction was quenched with saturated aqueous ammonium chloride and diluted with EtOAc. The organic layer was separated, washed with brine, and dried over anhydrous Na_2SO_4. After removal of solvents under reduced pressure, the residue was purified through column chromatography on silica gel to give **S5**.

General procedure for the preparation of arylcycloether[6, 7]

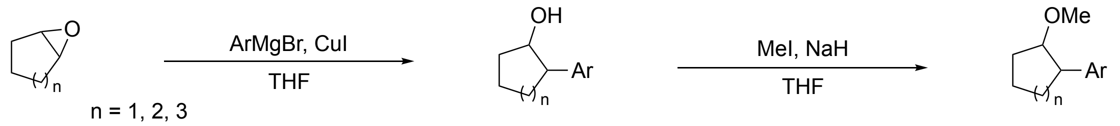

S6 **S7** **S8**

Copper iodide (0.05 equiv.) was added to a solution of arylmagnesium bromide (1 M in THF, 1.2 equiv.), and the reaction mixture was stirred at room temperature for 30 minutes. Then a solution of **S6** (1.0 equiv.) in THF was added dropwise at 0 °C for over 30 minutes. The reaction mixture was stirred for an additional 8 hours at room temperature. Then, the excess Grignard reagent was quenched by aqueous HCl (1 M) in an ice bath. The resulting mixture was poured into a separatory funnel containing water and extracted with Et_2O. The organic extracts were combined, dried over anhydrous Na_2SO_4, filtered, and concentrated in *vacuo*. The resulting residue was purified by flash column chromatography on silica gel to afford the corresponding **S7**.

The **S7** (1.0 equiv.) obtained above was dissolved in anhydrous THF (10 mL) and cooled to 0 °C. Sodium hydride (3.0 equiv.) was added in portions, followed by the dropwise addition of iodomethane (2.0 equiv.). The reaction mixture was stirred for another 30 minutes at room temperature. Once completed, the reaction was quenched with saturated aqueous ammonium chloride and diluted with EtOAc. The organic layer was separated, and the aqueous layer was extracted with EtOAc for three times. The combined organic layer was washed with brine, and dried over anhydrous Na_2SO_4. The solvents were removed under reduced pressure, and the residue was purified through column chromatography on silica gel to yield **S8**.

Procedure for the Preparation of 2-methoxy-3-phenylbicyclo[2.2.1]heptane[6, 7]

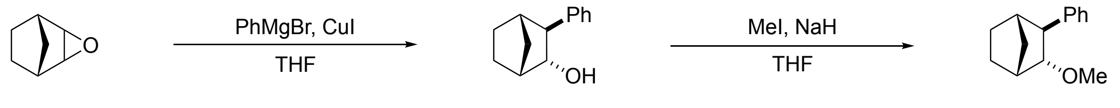

S9 **S10** **S11**

Copper iodide (0.05 equiv.) was added to a solution of phenylmagnesium bromide (1 M in THF, 1.2 equiv.), and the reaction mixture was stirred at room temperature for 30 minutes. Then a solution of **S9** (1.0 equiv.) in THF was added dropwise at 0 °C for over 30 minutes. The reaction mixture was stirred for an additional 8 h at room

temperature. Then, the excess Grignard reagent was quenched by aqueous HCl (1 M) in an ice bath. The resulting mixture was poured into a separatory funnel containing water and extracted with Et_2O. The organic extracts were combined, dried over anhydrous Na_2SO_4, filtered, and concentrated *in vacuo*. The resulting residue was purified by flash column chromatography on silica gel to afford the corresponding **S10**.

The **S10** (1.0 equiv.) obtained above was dissolved in anhydrous THF (10 mL) and cooled to 0 °C. Sodium hydride (3.0 equiv.) was added in portions, followed by the dropwise addition of iodomethane (2.0 equiv.). The reaction mixture was stirred for another 1 hours at 50 °C. Once completed, the reaction was quenched with saturated aqueous ammonium chloride and diluted with EtOAc. The organic layer was separated, and the aqueous layer was extracted with EtOAc for three times. The combined organic layer was washed with brine, and dried over anhydrous Na_2SO_4. The solvents were removed under reduced pressure, and the residue was purified through column chromatography on silica gel to yield **S11**.

Procedure for the Preparation of 1-phenylspiro[2.3]hexan[8, 9]

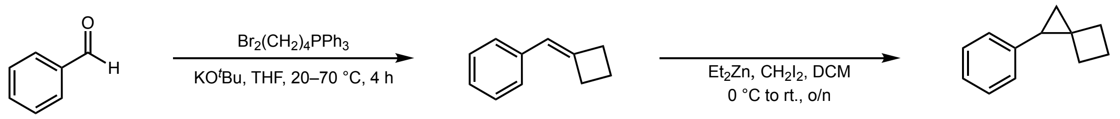

S12 **S13**

Under an argon atmosphere, the solution of ^{t}BuOK (3 equiv.) in THF (1 M) was added via a cannula to a suspension of 4-bromobutylphosphonium bromide (1.5 equiv.) in THF. Then the reaction mixture was heated at 70 °C for 1 hours, followed by the addition of benzaldehyde. After heating the reaction mixture at 80 °C for an additional 4 hours, it was then cooled down to room temperature. The reaction mixture was then poured into a large Erlenmeyer containing pentane (10 mL/mmol), and the precipitates were filtered. The filtrate was concentrated and the residue was purified using a silica gel chromatography to obtain the **S12**.

Under an argon atmosphere, the solution of dethylzinc (2.5 equiv.) in hexane (2 M) was added via a cannula to a solution of diiodomethane (4.0 equiv.) in DCM at -40 °C.

Then the reaction mixture was heated at this temperature for 15 minuts, followed by the addition of **S12** solution in DCM (3.5 M). After stirring the reaction mixture at room temberature for an additional 16 hours, it was then quenched by saturated NH_4Cl solution. The mixture was extracted with DCM and washed by sat. NaOH solution, water, and brine. Dried over anhydrous sodium sulfate and concentrated under *vacuo*. The residue was purified using a silica gel chromatography to obtain the **S13**.

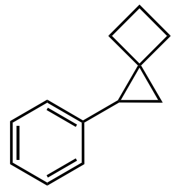

1-phenylspiro[2.3]hexane (S13): According to the general procedure for the preparation of arylcycloether**,** the residue was purified by flash chromatography (100% petroleum), a colorless oil was obtained (0.30 g, 94% yield). **^{1}H NMR (400 MHz, $CDCl_3$):** δ 7.27–7.23 (m, 2H), 7.15–7.11 (m, 1H), 6.96 (d, *J* = 8.0 Hz, 1H), 2.24–2.20 (m, 1H), 2.18–2.11 (m, 1H), 2.10–2.00 (m, 2H), 1.98–1.90 (m, 2H), 1.85–1.81 (m, 1H), 1.07–1.04 (m, 1H), 0.85 (t, *J* = 5.6 Hz, 1H); **^{13}C NMR (126 MHz, $CDCl_3$):** δ 141.6, 128.1, 126.8, 125.1, 31.6, 28.8, 28.3, 26.6, 20.6, 16.7.

6. Experimental procedures and spectral characterization of the products

6. 1 Substrate Scope

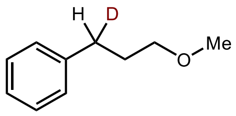

(3-Methoxypropyl-1-*d*)benzene (2): According to the general procedure, phenylcyclopropane (23.6 mg, 0.2 mmol, 1.0 equiv.), BPP (3.7 mg, 4 µmol, 0.02 equiv.), $(TRIPS)_2$ (9.4 mg, 0.02 mmol, 0.1 equiv.), and 1 mL MeCN/MeOD (v/v = 2/1) was used. Under the irradiation of LEDs (λ_{max} = 400 nm) for 16 h, colorless oil was obtained by flash chromatography (5% ethyl acetate in petroleum). 83% yield determined by GC-FID analysis with PhOMe as internal standard. D-inc.: 96% (determined by HR-MS). **^{1}H NMR (400 MHz, $CDCl_3$):** δ 7.33–7.29 (m, 2H), 7.23–7.19 (m, 3H), 3.41(t, *J* = 6.4 Hz, 2H), 3.37 (s, 3H), 2.73–2.68 (**m, 1H**), 1.91 (q, *J* = 6.8 Hz, 2H); **^{13}C NMR (100 MHz, $CDCl_3$):** δ 142.0, 128.6, 128.4, 125.9, 72.0, 58.7, 31.9 (t, $J_{C\text{-}D}$ = 19 Hz, benzylic carbon), 31.3. **HRMS (ESI+)**: calcd. for $C_{10}H_{14}DO^+$ ($[M+H]^+$) 152.1180, found 152.1178.

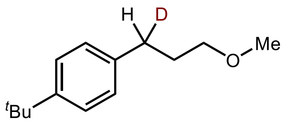

1-(*tert*-Butyl)-4-(3-methoxypropyl-1-*d*)benzene (6): According to the general procedure, 1-(*tert*-butyl)-4-cyclopropylbenzene (34.8 mg, 0.2 mmol, 1.0 equiv.), BPP (3.7 mg, 4 µmol, 0.02 equiv.), $(TRIPS)_2$ (9.4 mg, 0.02 mmol, 0.1 equiv.), and 1 mL MeCN/MeOD (v/v = 2/1) was used. Under the irradiation of LEDs (λ_{max} = 400 nm) for 16 h, colorless oil was obtained by flash chromatography (5% ethyl acetate in petroleum), 82% yield determined by ^{1}H NMR analysis with PhOMe as internal standard. D-inc.: 95% (determined by HR-MS). **^{1}H NMR (400 MHz, $CDCl_3$):** δ 7.34–7.32 (m, 2H), 7.16–7.14 (m, 2H), 3.42 (t, *J* = 6.4 Hz, 2H), 3.37 (s, 3H), 2.70–2.64 (**m, 1H**), 1.90 (q, *J* = 6.8 Hz, 2H), 1.34 (s, 9H); **^{13}C NMR (100 MHz, $CDCl_3$):** δ 148.7,

139.0, 128.2, 125.3, 72.2, 58.7, 34.5, 31.8–31.3 (m, deuterated benzylic carbon, overlaps with the methyl carbon of *tert*-butyl group), 31.6. **HRMS (ESI+):** calcd. for $C_{14}H_{22}DO^+$ ([M+H]$^+$) 208.1806, found 208.1808.

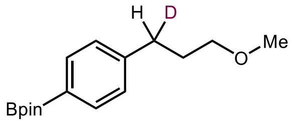

2-(4-(3-Methoxypropyl-1-*d*)phenyl)-4,4,5,5-tetramethyl-1,3,2-dioxaborolane (7): According to the general procedure, 2-(4-cyclopropylphenyl)-4,4,5,5-tetramethyl-1,3,2-dioxaborolane (48.8 mg, 0.2 mmol, 1.0 equiv.), BPP (3.7 mg, 4 μmol, 0.02 equiv.), $(TRIPS)_2$ (9.4 mg, 0.02 mmol, 0.1 equiv.), and 1 mL MeCN/MeOD (v/v = 2/1) was used. Under the irradiation of LEDs (λ_{max} = 400 nm) for 16 h, colorless oil was obtained by flash chromatography (5% ethyl acetate in petroleum), 86% yield determined by ^{1}H NMR analysis with PhOMe as internal standard, D-inc.: 95% (determined by HR-MS). **^{1}H NMR (400 MHz, $CDCl_3$):** δ 7.74 (d, J = 8.0 Hz, 2H), 7.21 (d, J = 8.0 Hz, 2H), 3.37 (t, J = 6.4 Hz, 2H), 3.33 (s, 3H), 2.72–2.66 **(m, 1H)**, 1.88 (q, J = 6.8 Hz, 2H), 1.34 (s, 12H); **^{13}C NMR (100 MHz, $CDCl_3$):** δ 145.5, 135.0, 128.1, 83.8, 72.0 58.7, 32.3 (t, $J_{C\text{-}D}$ = 19 Hz, benzylic carbon), 31.2, 25.0. A signal for the carbon directly attached to the boron atom was not observed. [10] **HRMS (ESI+):** calcd. for $C_{16}H_{24}DO_3BNa^+$ ([M+Na]$^+$) 299.1888, found 299.1889.

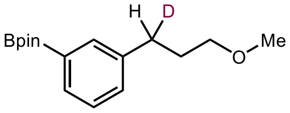

2-(3-(3-Methoxypropyl-1-*d*)phenyl)-4,4,5,5-tetramethyl-1,3,2-dioxaborolane (8): According to the general procedure, 2-(3-cyclopropylphenyl)-4,4,5,5-tetramethyl-1,3,2-dioxaborolane (48.8 mg, 0.2 mmol, 1.0 equiv.), BPP (3.7 mg, 4 μmol, 0.02 equiv.), $(TRIPS)_2$ (9.4 mg, 0.02 mmol, 0.1 equiv.), and 1 mL MeCN/MeOD (v/v = 2/1) was used. Under the irradiation of LEDs (λ_{max} = 400 nm) for 16 h, colorless oil was obtained by flash chromatography (5% ethyl acetate in petroleum), 88% yield determined by ^{1}H

NMR analysis with PhOMe as internal standard, D-inc.: 96% (determined by HR-MS). **^{1}H NMR (400 MHz, CDCl$_3$):** δ 7.67–7.64 (m, 2H), 7.31–7.29 (m, 2H), 3.38 (t, *J* = 6.4 Hz, 2H), 3.34 (s, 3H), 2.71–2.66 (**m, 1H**), 1.90 (q, *J* = 6.8 Hz, 2H), 1.35 (s, 12H); **^{13}C NMR (100 MHz, CDCl$_3$):** δ 141.3, 134.9, 132.4, 131.6, 127.9, 83.8, 72.1, 58.6, 32.3–31.8 (m, benzylic carbon), 31.4, 24.8. A signal for the carbon directly attached to the boron atom was not observed. [10] **HRMS (ESI+):** calcd. for $C_{16}H_{25}DO_3B^+$ ($[M+H]^+$) 277.2069, found 277.2071.

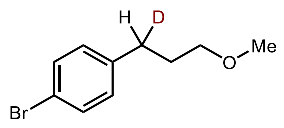

1-Bromo-4-(3-methoxypropyl-1-*d*)benzene (9): According to the general procedure, 1-Bromo-4-cyclopropylbenzene (39.2 mg, 0.2 mmol, 1.0 equiv.), BPP (3.7 mg, 4 μmol, 0.02 equiv.), (TRIPS)$_2$ (9.4 mg, 0.02 mmol, 0.1 equiv.), and 1 mL MeCN/MeOD (v/v = 2/1) was used. Under the irradiation of LEDs (λ_{max} = 400 nm) for 16 h, colorless oil was obtained by flash chromatography (5% ethyl acetate in petroleum), 75% yield determined by ^{1}H NMR analysis with PhOMe as internal standard, D-inc.: 95% (determined by HR-MS). **^{1}H NMR (400 MHz, CDCl$_3$):** δ 7.41–7.38 (m, 2H), 7.08–7.05 (m, 2H), 3.36 (t, *J* = 6.4 Hz, 2H), 3.34 (s, 3H), 2.66–2.60 (**m, 1H**), 1.85 (q, *J* = 6.8 Hz, 2H); **^{13}C NMR (100 MHz, CDCl$_3$):** δ 140.8, 131.3, 130.2, 119.5, 71.6, 58.6, 31.7–31.3 (m, benzylic carbon), 31.2. **HRMS (ESI+):** calcd. for $C_{10}H_{13}DOBr^+$ ($[M+H]^+$) 230.0285, found 230.0282.

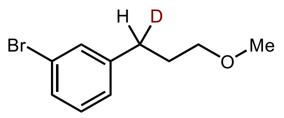

1-Bromo-3-(3-methoxypropyl-1-*d*)benzene (10): According to the general procedure, 1-Bromo-3-cyclopropylbenzene (39.2 mg, 0.2 mmol, 1.0 equiv.), BPP (3.7 mg, 4 μmol, 0.02 equiv.), (TRIPS)$_2$ (9.4 mg, 0.02 mmol, 0.1 equiv.), and 1 mL MeCN/MeOD (v/v = 2/1) was used. Under the irradiation of LEDs (λ_{max} = 400 nm) for 16 h, colorless oil

was obtained by flash chromatography (5% ethyl acetate in petroleum), 60% yield determined by ^{1}H NMR analysis with PhOMe as internal standard, D-inc.: 95% (determined by HR-MS). **^{1}H NMR (400 MHz, CDCl$_3$):** δ 7.35–7.30 (m, 2H), 7.17–7.10 (m, 2H), 3.37 (t, J = 6.4 Hz, 2H), 3.34 (s, 3H), 2.68–2.62 (**m, 1H**), 1.86 (q, J = 6.8 Hz, 2H); **^{13}C NMR (100 MHz, CDCl$_3$):** δ 144.4, 131.7, 130.0, 129.0, 127.3, 122.5, 71.7, 58.7, 31.8 (t, $J_{C\text{-}D}$ = 20 Hz, benzylic carbon), 31.1. **HRMS (EI+):** calcd. for $C_{10}H_{12}DOBr^+$ ([M]$^+$) 229.0207, found 229.0205.

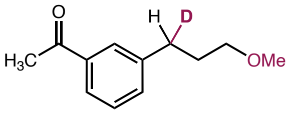

1-(3-(3-Methoxypropyl-1-*d*)phenyl)ethan-1-one (11): According to the general procedure, 1-(3-cyclopropylphenyl)ethanone (32.0 mg, 0.2 mmol, 1.0 equiv.), BPP (3.7 mg, 4 μmol, 0.02 equiv.), (TRIPS)$_2$ (9.4 mg, 0.02 mmol, 0.1 equiv.), and 1 mL MeCN/MeOD (v/v = 2/1) was used. Under the irradiation of LEDs (λ_{max} = 400 nm) for 16 h, colorless oil was obtained by flash chromatography (5% ethyl acetate in petroleum), 80% yield determined by ^{1}H NMR analysis with PhOMe as internal standard, D-inc.: 95% (determined by HR-MS). **^{1}H NMR (400 MHz, CDCl$_3$):** δ 7.79–7.76 (m, 2H), 7.40–7.35 (m, 2H), 3.38 (t, J = 6.4 Hz, 2H), 3.34 (s, 3H), 2.77–2.71 (**m, 1 H**), 2.59 (s, 3H), 1.89 (q, J = 6.8 Hz, 2H). **^{13}C NMR (100 MHz, CDCl$_3$):** δ 198.5, 142.6, 137.4, 133.4, 128.7, 128.3, 126.2, 71.8, 58.7, 32.3–31.8 (m, benzylic carbon), 31.2, 26.8. HRMS (ESI+): calcd. for $C_{12}H_{16}DO_2^+$ ([M+H]$^+$) 194.1286, found 194.1283.

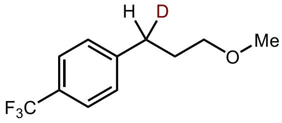

1-(3-Methoxypropyl-1-*d*)-4-(trifluoromethyl)benzene (12): According to the general procedure, 1-cyclopropyl-4-(trifluoromethyl)benzene (37.2 mg, 0.2 mmol, 1.0 equiv.), BPP (3.7 mg, 4 μmol, 0.02 equiv.), (TRIPS)$_2$ (9.4 mg, 0.02 mmol, 0.1 equiv.), and 1 mL MeCN/MeOD (v/v = 2/1) was used. Under the irradiation of LEDs (λ_{max} = 400 nm) for

16 h, colorless oil was obtained by flash chromatography (5% ethyl acetate in petroleum), 54% yield determined by ^{1}H NMR analysis with PhOMe as internal standard, D-inc.: 96% (determined by HR-MS). **^{1}H NMR (400 MHz, $CDCl_3$):** δ 7.54–4.52 (m, 2H), 7.31–7.29 (m, 2H), 3.38 (t, *J* = 6.4Hz, 2H), 3.35 (s, 3H), 2.77–2.71 (**m, 1 H**), 1.89 (q, *J* = 6.8 Hz, 2H); **^{13}C NMR (100 MHz, $CDCl_3$):** δ 146.2, 128.9, 128.3 (q, $J_{CF3\text{-}C}$ = 33 Hz), 124.5 (q, $J_{C\text{-}F}$ = 270 Hz), 125.4 (q, $J_{CF3\text{-}C}$ = 4.0 Hz), 71.6, 58.7, 32.0 (t, $J_{C\text{-}D}$ = 19 Hz, benzylic carbon), 30.1. **^{19}F NMR (376 MHz, $CDCl_3$):** δ -62.34. **HRMS (EI+):** calcd. for $C_{10}H_8F_3^+$ ([M-CH_3OH]$^+$) 187.0714, found 187.0709.

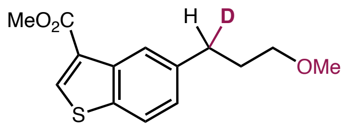

Methyl 5-(3-methoxypropyl-1-*d*)benzo[*b*]thiophene-3-carboxylate (13): According to the general procedure, methyl 5-cyclopropylbenzo[*b*]thiophene-3-carboxylate (46.4 mg, 0.2 mmol, 1.0 equiv.), BPP (3.7 mg, 4 μmol, 0.1 equiv.), $(TRIPS)_2$ (9.4 mg, 0.02 mmol, 0.1 equiv.), and 1 mL MeCN/MeOD (v/v = 2/1) was used. Under the irradiation of LEDs (λ_{max} = 400 nm) for 16 h, pale yellow waxy solid was obtained by flash chromatography (9% ethyl acetate in petroleum). 66% yield determined by isolation. D-inc.: 87% (determined by HR-MS). **^{1}H NMR (400 MHz, $CDCl_3$):** δ 8.00 (s, 1H), 7.77 (d, *J* = 8.0 Hz, 1H), 7.68 (m, 1H), 7.32–7.30 (m, 1H), 3.94 (s, 3H), 3.40 (t, *J* = 6.4 Hz, 2H), 3.35 (s, 3H), 2.83–2.78 (**m, 1H**), 1.93 (q, *J* = 7.2 Hz, 2H); **^{13}C NMR (100 MHz, $CDCl_3$):** δ 163.5, 140.1, 139.2, 139.1, 133.6, 130.6, 128.4, 124.9, 122.7, 71.9, 58.7, 52.6, 32.0 (t, $J_{C\text{-}D}$ = 19 Hz, benzylic carbon, Peak at 32.3 ppm is the benzylic carbon of no-deuterated product), 31.5. **HRMS (FI+)**: calcd. for $C_{14}H_{15}DO_3S^+$ ([M]$^+$) 265.0877, found 265.0875.

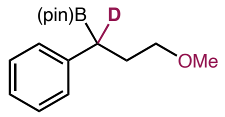

2-(3-Methoxy-1-phenylpropyl-1-*d*)-4,4,5,5-tetramethyl-1,3,2-dioxaborolane (14):

According to the general procedure, 4,4,5,5-tetramethyl-2-(1-phenylcyclopropyl)-1,3,2-dioxaborolane (48.8 mg, 0.2 mmol, 1.0 equiv.), BPP (3.7 mg, 4 μmol, 0.1 equiv.), $(TRIPS)_2$ (9.4 mg, 0.02 mmol, 0.1 equiv.), and 1 mL MeCN/MeOD (v/v = 2/1) was used. Under the irradiation of LEDs (λ_{max} = 400 nm) for 20 h, colorless oil was obtained by flash chromatography (9% ethyl acetate in petroleum). 65% yield determined by isolation. D-inc.: 83% (determined by HR-MS). **^{1}H NMR (400 MHz, $CDCl_3$):** δ 7.27–7.20 (m, 4H), 7.16–7.10 (m, 1H), 3.41–3.31 (m, 2H), 3.30 (s, 3H), 2.41 (t, *J* = 8.0 Hz, 0.13H, from no-deuterated product), 2.19–2.12 (m, 1H), 1.89–1.83 (m, 1H), 1.19 (s, 6H), 1.18 (s, 6H); **^{13}C NMR (100 MHz, $CDCl_3$):** δ 143.04, 128.51, 128.42, 125.36, 83.40, 72.15, 58.60, (32.49, from no-deuterated product), 32.42, 24.72, 24.70. **HRMS (ESI+)**: calcd. for $C_{16}H_{25}DBO_3^+$ ([M+H]$^+$) 278.2032, found 278.2029.

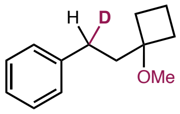

(2-(1-Methoxycyclobutyl)ethyl-1-*d*)benzene (15): According to the general procedure, 1-phenylspiro[2.3]hexane (31.6 mg, 0.2 mmol, 1.0 equiv.), BPP (3.7 mg, 4 μmol, 0.02 equiv.), $(TRIPS)_2$ (9.4 mg, 0.02 mmol, 0.1 equiv.), and 1 mL MeCN/MeOD (v/v = 2/1) was used. Under the irradiation of LEDs (λ_{max} = 400 nm) for 16 h, colorless oil was obtained by flash chromatography (100% petroleum). 45% yield determined by isolation. D-inc.: 91% (determined by HR-MS). **^{1}H NMR (400 MHz, $CDCl_3$):** δ 7.32–7.28 (m, 2H), 7.24–7.17 (m, 3H), 3.18 (s, 3H), 2.64–2.58 (**m, 1H**), 2.14–2.09 (m, 2H), 1.95–1.88 (m, 4H), 1.83–1.74 (m, 1H), 1.64–1.57 (m, 1H); **^{13}C NMR (100 MHz, $CDCl_3$):** δ 142.92, 128.51, 128.48, 125.81, 79.38, 49.35, 36.91, 36.84, 31.60, 29.25 (t, $J_{C\text{-}D}$ = 20 Hz, benzylic carbon), 12.67. **HRMS (FI+)**: calcd. for $C_{13}H_{17}DO^+$ ([M]$^+$) 191.1415, found 191.1419.

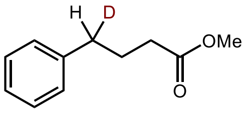

Methyl 4-phenylbutanoate-4-*d* (16): According to the general procedure, 2-phenylcyclobutanone (29.2 mg, 0.2 mmol, 1.0 equiv.), BPP (3.7 mg, 4 μmol, 0.02 equiv.), $(TRIPS)_2$ (9.4 mg, 0.02 mmol, 0.1 equiv.), and 1 mL MeCN/MeOD (v/v = 2/1) was used. Under the irradiation of LEDs (λ_{max} = 400 nm) for 16 h, colorless oil was obtained by flash chromatography (5% ethyl acetate in petroleum), 78% yield determined by ^{1}H NMR analysis with PhOMe as internal standard, D-inc.: 95% (determined by HR-MS). **^{1}H NMR (400 MHz, $CDCl_3$):** δ 7.30–7.27 (m, 2H), 7.21–7.17 (m, 3H), 3.66 (s, 3H), 2.67–2.62 (**m, 1 H**), 2.33 (t, J = 7.2 Hz, 2H), 1.96 (q, J = 7.6 Hz, 2H); **^{13}C NMR (100 MHz, $CDCl_3$):** δ 174.0, 141.4, 128.6, 128.5, 126.1, 51.6, 34.9 (t, $J_{C\text{-}D}$ = 19 Hz, benzylic carbon), 33.4, 26.5. **HRMS (ESI+):** calcd. for $C_{11}H_{13}DO_2Na^+$ ($[M+Na]^+$) 202.0949, found 202.0947.

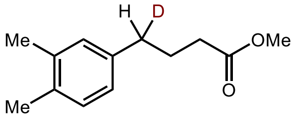

Methyl 4-(3,4-dimethylphenyl)butanoate-4-*d* (17): According to the general procedure, 2-(3,4-dimethylphenyl)cyclobutan-1-one (34.8 mg, 0.2 mmol, 1.0 equiv.), BPP (3.7 mg, 4 μmol, 0.02 equiv.), $(TRIPS)_2$ (9.4 mg, 0.02 mmol, 0.1 equiv.), and 1 mL MeCN/MeOD (v/v = 2/1) was used. Under the irradiation of LEDs (λ_{max} = 400 nm) for 16 h, colorless oil was obtained by flash chromatography (5% ethyl acetate in petroleum), 78% yield determined by ^{1}H NMR analysis with PhOMe as internal standard, D-inc.: 97% (determined by HR-MS). **^{1}H NMR (400 MHz, $CDCl_3$):** δ 7.06 (d, J = 7.6 Hz, 1H), 6.97 (s, 1H), 6.92 (d, J = 7.6 Hz, 1H), 3.68 (s, 3H), 2.61–2.56 (**m, 1 H**), 2.34 (t, J = 7.6 Hz, 2H), 2.25 (s, 3H), 2.24 (s, 3H), 1.94 (q, J = 7.6 Hz, 2H); **^{13}C NMR (100 MHz, $CDCl_3$):** δ 174.2, 138.9, 136.6, 134.2, 130.0, 129.7, 125.9, 51.6, 34.4 (t, $J_{C\text{-}D}$ = 20 Hz, benzylic carbon), 33.6, 26.7, 19.0, 19.4. **HRMS (ESI+):** calcd. for $C_{13}H_{17}DO_2Na^+$ ($[M+Na]^+$) 230.1262, found 230.1259.

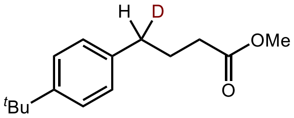

Methyl 4-(4-(*tert*-butyl)phenyl)butanoate-4-*d* (18): According to the general procedure, 2-(*p*-*tert*-butylphenyl)cyclobutanone (40.4 mg, 0.2 mmol, 1.0 equiv.), BPP (3.7 mg, 4 μmol, 0.02 equiv.), $(TRIPS)_2$ (9.4 mg, 0.02 mmol, 0.1 equiv.), and 1 mL MeCN/MeOD (v/v = 2/1) was used. Under the irradiation of LEDs (λ_{max} = 400 nm) for 16 h, colorless oil was obtained by flash chromatography (5% ethyl acetate in petroleum), 66% yield determined by ^{1}H NMR analysis with PhOMe as internal standard, D-inc.: 96% (determined by HR-MS). **^{1}H NMR (400 MHz, $CDCl_3$):** δ 7.31 (d, J = 8.0 Hz, 2H), 7.12 (d, J = 8.4 Hz, 2H), 3.67 (s, 3H), 2.64–2.59 (**m, 1 H**), 2.34 (t, J = 7.6 Hz, 2H), 1.95 (q, J = 7.6 Hz, 2H), 1.31 (s, 9H); **^{13}C NMR (100 MHz, $CDCl_3$):** δ 174.2, 148.9, 138.4, 128.3, 125.4, 51.6, 34.5–34.2 (m, deuterated benzylic carbon, overlaps with the benzylic carbon of *tert*-butyl group), 33.6, 31.5, 26.5. **HRMS (ESI+):** calcd. for $C_{15}H_{21}DO_2Na^+$ ($[M+Na]^+$) 258.1575, found 258.1571.

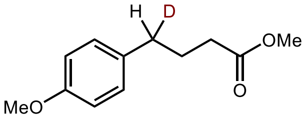

Methyl 4-(4-methoxyphenyl)butanoate-4-*d* (19): According to the general procedure, 2-(4-methoxyphenyl)cyclobutanone (35.2 mg, 0.2 mmol, 1.0 equiv.), BPP (3.7 mg, 4 μmol, 0.02 equiv.), $(TRIPS)_2$ (9.4 mg, 0.02 mmol, 0.1 equiv.), and 1 mL MeCN/MeOD (v/v = 2/1) was used. Under the irradiation of LEDs (λ_{max} = 400 nm) for 16 h, colorless oil was obtained by flash chromatography (5% ethyl acetate in petroleum), 82% yield determined by ^{1}H NMR analysis with PhOMe as internal standard, D-inc.: 96% (determined by HR-MS). **^{1}H NMR (400 MHz, $CDCl_3$):** δ 7.09 (d, J = 8.4 Hz, 2H), 6.83 (d, J = 8.4 Hz, 2H), 3.78 (s, 3H), 3.66 (s, 3H), 2.59–2.55 (**m, 1 H**), 2.32 (t, J = 7.6 Hz, 2H), 1.91 (q, J = 7.6 Hz, 2H); **^{13}C NMR (100 MHz, $CDCl_3$):** δ 174.1, 158.0, 133.5, 129.5, 113.9, 55.3, 51.6, 33.9 (t, $J_{C\text{-}D}$ = 20 Hz, benzylic carbon), 33.4, 26.8. **HRMS (ESI+):** calcd. for $C_{12}H_{15}DO_3Na^+$ ($[M+Na]^+$) 232.1054, found 232.1051.

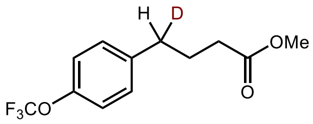

Methyl 4-(4-(trifluoromethoxy)phenyl)butanoate-4-*d* (20): According to the general procedure, 2-[4-(trifluoromethoxy)phenyl]cyclobutan-1-one (46.0 mg, 0.2 mmol, 1.0 equiv.), BPP (3.7 mg, 4 μmol, 0.02 equiv.), $(TRIPS)_2$ (9.4 mg, 0.02 mmol, 0.1 equiv.), and 1 mL MeCN/MeOD (v/v = 2/1) was used. Under the irradiation of LEDs (λ_{max} = 400 nm) for 16 h, colorless oil was obtained by flash chromatography (5% ethyl acetate in petroleum), 54% yield determined by ^{1}H NMR analysis with PhOMe as internal standard, D-inc.: 95% (determined by HR-MS). **^{1}H NMR (400 MHz, $CDCl_3$):** δ 7.19 (d, J = 8.4 Hz, 2H), 7.12 (d, J = 8.4 Hz, 2H), 3.66 (s, 3H), 2.67–2.62 (**m, 1 H**), 2.33 (t, J = 7.2 Hz, 2H), 1.94 (q, J = 7.6 Hz, 2H); **^{13}C NMR (100 MHz, $CDCl_3$):** 173.9, 147.6, 140.2, 129.8z, 121.1, 120.6 (q, $J_{C\text{-}F}$ = 255 Hz), 51.7, 34.2 (t, $J_{C\text{-}D}$ = 20 Hz, benzylic carbon), 33.4, 26.4. **^{19}F NMR (376 MHz, $CDCl_3$):** δ -58.00. **HRMS (ESI+):** calcd. for $C_{12}H_{13}DO_3F_3^+$ ($[M+H]^+$) 264.0952, found 264.0954.

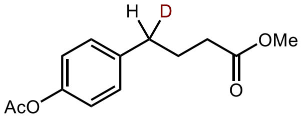

Methyl 4-(4-acetoxyphenyl)butanoate-4-*d* (21): According to the general procedure, 4-(2-oxocyclobutyl)phenyl acetate (40.8 mg, 0.2 mmol, 1.0 equiv.), BPP (3.7 mg, 4 μmol, 0.02 equiv.), $(TRIPS)_2$ (9.4 mg, 0.02 mmol, 0.1 equiv.), and 1 mL MeCN/MeOD (v/v = 2/1) was used. Under the irradiation of LEDs (λ_{max} = 400 nm) for 16 h, colorless oil was obtained by flash chromatography (5% ethyl acetate in petroleum), 70% yield determined by ^{1}H NMR analysis with PhOMe as internal standard, D-inc.: 98% (determined by HR-MS). **^{1}H NMR (400 MHz, $CDCl_3$):** δ 7.17 (d, J = 8.4 Hz, 2H), 6.99 (d, J = 8.4 Hz, 2H), 3.66 (s, 3H), 2.66–2.60 (**m, 1H**), 2.33 (t, J = 7.6 Hz, 2H), 2.28 (s, 3H), 1.93 (q, J = 7.6 Hz, 2H); **^{13}C NMR (100 MHz, $CDCl_3$):** 174.0, 169.8, 149.0, 139.0,

129.5, 121.5, 51.7, 34.2 (t, J_{C-D} = 20 Hz, benzylic carbon), 33.4, 26.4, 21.2. **HRMS (EI+):** calcd. for $C_{13}H_{15}DO_4^+$ ([M]$^+$) 237.1106, found 237.1109.

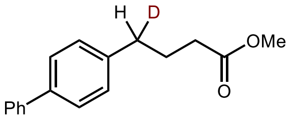

Methyl 4-([1,1'-biphenyl]-4-yl)butanoate-4-*d* (22): According to the general procedure, 2-([1,1'-biphenyl]-4-yl)cyclobutan-1-one (44.5 mg, 0.2 mmol, 1.0 equiv.), BPP (3.7 mg, 4 μmol, 0.02 equiv.), $(TRIPS)_2$ (9.4 mg, 0.02 mmol, 0.1 equiv.), and 1 mL MeCN/MeOD (v/v = 2/1) was used. Under the irradiation of LEDs (λ_{max} = 400 nm) for 16 h, colorless oil was obtained by flash chromatography (5% ethyl acetate in petroleum), 66% yield determined by ^{1}H NMR analysis with PhOMe as internal standard, D-inc.: 95% (determined by HR-MS). **^{1}H NMR (400 MHz, $CDCl_3$):** δ 7.41–7.33 (m, 4H), 7.25 (t, J = 7.6 Hz, 2H), 7.17–7.12 (m, 1H), 7.07 (d, J = 8.0 Hz, 2H), 3.50 (s, 3H), 2.52–2.48 (**m, 1 H**), 2.19 (t, J = 7.6 Hz, 2H), 1.81 (q, J = 7.6 Hz, 2H); **^{13}C NMR (100 MHz, $CDCl_3$):** δ 174.0, 141.1, 140.6, 139.1, 129.0, 128.8, 127.2, 127.2, 127.1, 51.6, 34.5 (t, J_{C-D} = 20 Hz, benzylic carbon), 33.5, 26.5. **HRMS (ESI+):** calcd. for $C_{17}H_{17}DO_2Na^+$ ([M+Na]$^+$) 278.1262, found 278.1263.

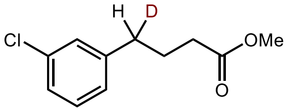

Methyl 4-(3-chlorophenyl)butanoate-4-*d* (23): According to the general procedure, 2-(3-chlorophenyl)cyclobutan-1-one (36.0 mg, 0.2 mmol, 1.0 equiv.), BPP (3.7 mg, 4 μmol, 0.02 equiv.), $(TRIPS)_2$ (9.4 mg, 0.02 mmol, 0.1 equiv.), and 1 mL MeCN/MeOD (v/v = 2/1) was used. Under the irradiation of LEDs (λ_{max} = 400 nm) for 16 h, colorless oil was obtained by flash chromatography (5% ethyl acetate in petroleum), 76% yield determined by ^{1}H NMR analysis with PhOMe as internal standard, D-inc.: 98% (determined by HR-MS). **^{1}H NMR (400 MHz, $CDCl_3$):** δ 7.23–7.17 (m, 3H), 7.07–7.04 (m, 1H), 3.67 (s, 3H), 2.65–2.59 (**m, 1 H**), 2.33 (t, J = 7.6 Hz,

2H), 1.94 (q, J = 7.6 Hz, 2H); **^{13}C NMR (100 MHz, $CDCl_3$):** δ 173.9, 143.5, 134.3, 129.8, 128.7, 126.8, 126.3, 51.7, 34.5 (t, $J_{C\text{-}D}$ = 20 Hz, benzylic carbon), 33.3, 26.3. **HRMS (ESI+):** calcd. for $C_{11}H_{13}DO_2Cl^+$ ([M+H]$^+$) 214.0740, found 214.0743.

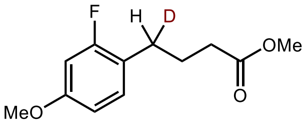

Methyl 4-(2-fluoro-4-methoxyphenyl)butanoate-4-*d* (24): According to the general procedure, 2-(2-fluoro-4-methoxyphenyl)cyclobutan-1-one (38.8 mg, 0.2 mmol, 1.0 equiv.), BPP (3.7 mg, 4 μmol, 0.02 equiv.), $(TRIPS)_2$ (9.4 mg, 0.02 mmol, 0.1 equiv.), and 1 mL MeCN/MeOD (v/v = 2/1) was used. Under the irradiation of LEDs (λ_{max} = 400 nm) for 16 h, colorless oil was obtained by flash chromatography (5% ethyl acetate in petroleum), 80% yield determined by ^{1}H NMR analysis with PhOMe as internal standard, D-inc.: 95% (determined by HR-MS). **^{1}H NMR (400 MHz, $CDCl_3$):** δ 7.07–7.03 (m, 1H), 6.63–6.56 (m, 2H), 3.76 (s, 3H), 3.65 (s, 3H), 2.60–2.57 (**m, 1H**), 2.32 (t, J = 7.6 Hz, 2H), 1.90 (q, J = 7.6 Hz, 2H); **^{13}C NMR (100 MHz, $CDCl_3$):** δ 174.0, 161.6 (d, $J_{C\text{-}F}$ = 243 Hz), 159.3 (d, $J_{C\text{-}F}$ = 11 Hz), 131.0 (d, $J_{C\text{-}F}$ = 7 Hz), 119.9 (d, $J_{C\text{-}F}$ = 17 Hz), 109.7 (d, $J_{C\text{-}F}$ = 3 Hz), 101.6 (d, $J_{C\text{-}F}$ = 26 Hz), 55.6, 51.6, 33.4, 27.4 (t, $J_{C\text{-}D}$ = 20 Hz, benzylic carbon), 25.5. **^{19}F NMR (376 MHz, $CDCl_3$):** δ -116.72–(-116.67) (m). **HRMS (ESI+):** calcd. for $C_{12}H_{14}DO_3FNa^+$ ([M+Na]$^+$) 250.0960, found 250.0962.

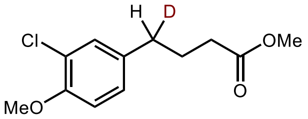

Methyl 4-(3-chloro-4-methoxyphenyl)butanoate-4-*d* (25): According to the general procedure, 2-(3-chloro-4-methoxyphenyl)cyclobutan-1-one (42.0 mg, 0.2 mmol, 1.0 equiv.), BPP (3.7 mg, 4 μmol, 0.02 equiv.), $(TRIPS)_2$ (9.4 mg, 0.02 mmol 0.1 equiv.), and 1 mL MeCN/MeOD (v/v = 2/1) was used. Under the irradiation of LEDs (λ_{max} =

400 nm) for 16 h, colorless oil was obtained by flash chromatography (5% ethyl acetate in petroleum), 72% yield determined by ^{1}H NMR analysis with PhOMe as internal standard, D-inc.: 96% (determined by HR-MS). **^{1}H NMR (400 MHz, CDCl$_3$):** δ 7.18 (d, J = 2.4 Hz, 1H), 7.01 (dd, J = 8.4, 2.4 Hz, 1H), 6.83 (d, J = 8.4 Hz, 1H), 3.86 (s, 3H), 3.65 (s, 3H), 2.58–2.52 (**m, 1 H**), 2.30 (t, J = 7.6 Hz, 2H), 1.89 (q, J = 7.6 Hz, 2H); **^{13}C NMR (100 MHz, CDCl$_3$):** δ 173.9, 153.4, 134.6, 130.2, 127.0, 122.2, 112.2, 56.3, 51.6, 33.6 (t, $J_{C\text{-}D}$ = 20 Hz, benzylic carbon), 33.3, 26.5. **HRMS (ESI+):** calcd. for $C_{12}H_{14}DO_3ClNa^+$ ([M+Na]$^+$) 266.0665, found 266.0666.

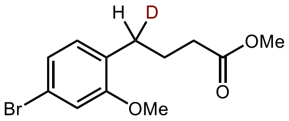

Methyl 4-(4-bromo-2-methoxyphenyl)butanoate-4-*d* (26): According to the general procedure, 2-(4-bromo-2-methoxyphenyl)cyclobutan-1-one (50.8 mg, 0.2 mmol, 1.0 equiv.), BPP (3.7 mg, 4 µmol, 0.02 equiv.), (TRIPS)$_2$ (9.4 mg, 0.02 mmol, 0.1 equiv.), and 1 mL MeCN/MeOD (v/v = 2/1) was used. Under the irradiation of LEDs (λ_{max} = 400 nm) for 16 h, colorless oil was obtained by flash chromatography (5% ethyl acetate in petroleum), 62% yield determined by ^{1}H NMR analysis with PhOMe as internal standard, D-inc.: 95% (determined by HR-MS). **^{1}H NMR (400 MHz, CDCl$_3$):** δ 7.00–6.94 (m, 3H), 3.79 (s, 3H), 3.65 (s, 3H), 2.60–2.55 (**m, 1 H**), 2.30 (t, J = 7.6 Hz, 2H), 1.87 (q, J = 7.6 Hz, 2H); **^{13}C NMR (100 MHz, CDCl$_3$):** δ 174.1, 158.2, 131.2, 128.9, 123.4, 120.3, 113.9, 55.6, 51.6, 33.5, 28.9 (t, $J_{C\text{-}D}$ = 20 Hz, benzylic carbon), 24.7. **HRMS (ESI+):** calcd. for $C_{12}H_{15}DO_3Br^+$ ([M+H]$^+$) 288.0340, found 288.0336.

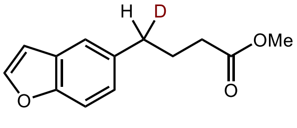

Methyl 4-(benzofuran-5-yl)butanoate-4-*d* (27): According to the general procedure, 2-(benzofuran-5-yl)cyclobutan-1-one (37.2 mg, 0.2 mmol, 1.0 equiv.), BPP (3.7 mg, 4 µmol, 0.02 equiv.), (TRIPS)$_2$ (9.4 mg, 0.02 mmol, 0.1 equiv.), and 1 mL MeCN/MeOD (v/v = 2/1) was used. Under the irradiation of LEDs (λ_{max} = 400 nm) for 16 h, colorless

oil was obtained by flash chromatography (5% ethyl acetate in petroleum), 83% yield determined by ^{1}H NMR analysis with PhOMe as internal standard, D-inc.: 95% (determined by HR-MS). **^{1}H NMR (400 MHz, CDCl$_3$):** δ 7.60 (d, *J* = 2.4 Hz, 1H), 7.43–7.39 (m, 2H), 7.11 (dd, *J* = 8.4, 1.6 Hz, 1H), 6.71 (dd, *J* = 2.0, 0.8 Hz, 1H), 3.67 (s, 3H), 2.76–2.70 (**m, 1 H**), 2.35 (t, *J* = 7.6 Hz, 2H), 1.99 (q, *J* = 7.6 Hz, 2H); **^{13}C NMR (100 MHz, CDCl$_3$):** δ 174.1, 153.8, 145.2, 135.9, 127.6, 125.0, 120.7, 111.2, 106.5, 51.6, 34.7 (t, $J_{C\text{-}D}$ = 20 Hz, benzylic carbon), 33.4, 27.1. **HRMS (ESI+):** calcd. for $C_{13}H_{13}DO_3Na^+$ ([M+Na]$^+$) 242.0898, found 242.0896.

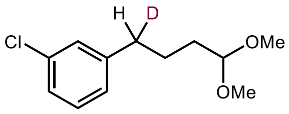

1-Chloro-3-(4,4-dimethoxybutyl-1-*d*)benzene (28): According to the general procedure, 1-chloro-3-(2-methoxycyclobutyl)benzene (39.2 mg, 0.2 mmol, 1.0 equiv.), BPP (3.7 mg, 4 μmol, 0.02 equiv.), (TRIPS)$_2$ (9.4 mg, 0.02 mmol, 0.1 equiv.), and 1 mL MeCN/MeOD (v/v = 2/1) was used. Under the irradiation of LEDs (λ_{max} = 400 nm) for 16 h, colorless oil was obtained by flash chromatography (5% ethyl acetate in petroleum), 67% yield determined by ^{1}H NMR analysis with PhOMe as internal standard, D-inc.: 98% (determined by HR-MS). **^{1}H NMR (400 MHz, CDCl$_3$):** δ 7.22–7.14 (m, 3H), 7.07–7.04 (m, 1H), 4.36 (t, *J* = 5.6 Hz, 1H), 3.30 (s, 6H), 2.63–2.57 (**m, 1 H**), 1.68–1.61 (m, 4H); **^{13}C NMR (100 MHz, CDCl$_3$):** δ 144.3, 134.2, 129.7, 128.6, 126.7, 126.1, 104.4, 52.9, 35.3–34.9 (m, benzylic carbon), 32.1, 26.2. **HRMS (EI+):** calcd. for $C_{11}H_{12}DClO^+$ ([M-CH$_3$OH]$^+$) 197.0712, found 197.0719.

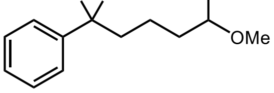

(5,5-Dimethoxypentyl-1-*d*)benzene (29): According to the general procedure, (2-methoxycyclopentyl)benzene (35.2 mg, 0.2 mmol, 1.0 equiv.), BPP (3.7 mg, 4 μmol, 0.02 equiv.), (TRIPS)$_2$ (9.4 mg, 0.02 mmol, 0.1 equiv.), and 1 mL MeCN/MeOD (v/v =

2/1) was used. Under the irradiation of LEDs (λ_{max} = 400 nm) for 16 h, colorless oil was obtained by flash chromatography (5% ethyl acetate in petroleum), 65% yield determined by ^{1}H NMR analysis with PhOMe as internal standard, D-inc.: 95% (determined by HR-MS). **^{1}H NMR (400 MHz, CDCl$_3$):** δ 7.29–7.25 (m, 2H), 7.19–7.15 (m, 3H), 4.35 (t, J = 5.6 Hz, 1H), 3.31 (s, 6H), 2.63–2.58 (**m, 1 H**), 1.66–1.60 (m, 4H), 1.43–1.35 (m, 2H); **^{13}C NMR (100 MHz, CDCl$_3$):** δ 142.6, 128.5, 128.4, 125.8, 104.6, 52.8, 35.6 (t, $J_{C\text{-}D}$ = 19 Hz, benzylic carbon), 32.5, 31.4, 24.4. **HRMS (ESI+):** calcd. for $C_{13}H_{19}DO_2Na^+$ ([M+Na]$^+$) 232.1418, found 232.1421.

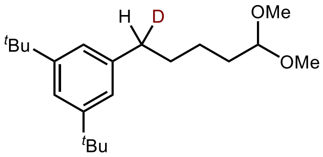

1,3-*di-tert*-Butyl-5-(5,5-dimethoxypentyl-1-*d*)benzene (30): According to the general procedure, 1,3-*di-tert*-butyl-5-(2-methoxycyclopentyl)benzene (57.6 mg, 0.2 mmol, 1.0 equiv.), BPP (3.7 mg, 4 μmol, 0.02 equiv.), (TRIPS)$_2$ (9.4 mg, 0.02 mmol, 0.1 equiv.), and 1 mL MeCN/MeOD (v/v = 2/1) was used. Under the irradiation of LEDs (λ_{max} = 400 nm) for 16 h, colorless oil was obtained by flash chromatography (5% ethyl acetate in petroleum), 90% yield determined by ^{1}H NMR analysis with PhOMe as internal standard, D-inc.: 96% (determined by HR-MS). **^{1}H NMR (400 MHz, CDCl$_3$):** δ 7.29–7.28 (m, 1H), 7.07–7.06 (m, 2H), 4.42 (t, J = 5.6 Hz, 1H), 3.35 (s, 6H), 2.65–2.61 (**m, 1 H**), 1.72–1.60 (m, 4H), 1.52-1.45 (m, 2H), 1.36 (s, 18H); **^{13}C NMR (100 MHz, CDCl$_3$):** δ 150.61, 141.67, 122.67, 119.80, 104.60, 52.73, 36.23 (t, $J_{C\text{-}D}$ = 19 Hz, benzylic carbon), 34.87, 32.50, 31.69, 31.65, 24.66. **HRMS (ESI+):** calcd. for $C_{21}H_{35}DO_2Na^+$ ([M+Na]$^+$) 344.2670, found 344.2665.

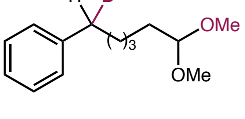

(6,6-Dimethoxyhexyl-1-*d*)benzene (31): According to the general procedure, (2-

methoxycyclohexyl)benzene (38.0 mg, 0.2 mmol, 1.0 equiv.), BPP (3.7 mg, 4 µmol, 0.02 equiv.), $(TRIPS)_2$ (9.4 mg, 0.02 mmol, 0.1 equiv.), and 1 mL MeCN/MeOD (v/v = 2/1) was used. Under the irradiation of LEDs (λ_{max} = 400 nm) for 16 h, colorless oil was obtained by flash chromatography (5% ethyl acetate in petroleum), 53% yield determined by ^{1}H NMR analysis with PhOMe as internal standard, D-inc.: 95% (determined by HR-MS). **^{1}H NMR (400 MHz, $CDCl_3$):** δ 7.29–7.25 (m, 2H), 7.18-7.16 (m, 3H), 4.35 (t, *J* = 5.6 Hz, 1H), 3.30 (s, 6H), 2.62–2.57 (m, 1H), 1.64–1.57 (m, 4H), 1.40–1.33 (m, 4H); **^{13}C NMR (100 MHz, $CDCl_3$):** δ142.8, 128.5, 128.4, 125.7, 104.6, 52.7, 35.6 (t, $J_{C\text{-}D}$ = 20 Hz, benzylic carbon), 32.5, 31.4, 29.2, 24.6. **HRMS (EI+):** calcd. for $C_{13}H_{17}DO^{+}$ ($[M\text{-}CH_3OH]^{+}$) 191.1415, found 191.1414.

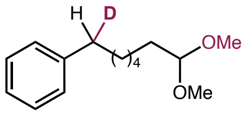

(7,7-Dimethoxyheptyl-1-*d*)benzene (32): According to the general procedure, 1-methoxy-2-phenylcycloheptane (40.8 mg, 0.2 mmol, 1.0 equiv.), BPP (3.7 mg, 4 µmol, 0.02 equiv.), $(TRIPS)_2$ (9.4 mg, 0.02 mmol, 0.1 equiv.), and 1 mL MeCN/MeOD (v/v = 2/1) was used. Under the irradiation of LEDs (λ_{max} = 400 nm) for 16 h, colorless oil was obtained by flash chromatography (5% ethyl acetate in petroleum), 68% yield determined by ^{1}H NMR analysis with PhOMe as internal standard, D-inc.: 95% (determined by HR-MS). **^{1}H NMR (400 MHz, $CDCl_3$):** δ 7.29–7.26 (m, 2H), 7.18–7.16 (m, 3H), 4.37–4.34 (m, 1H), 3.31 (m, 6H), 2.62–2.56 (**m, 1 H**), 1.62–1.56 (m, 5(4)H, water peak contaminated), 1.35–1.34 (m, 6H); **^{13}C NMR (100 MHz, $CDCl_3$):** δ 142.9, 128.5, 128.4, 125.7, 104.7, 53.6, 35.7 (t, $J_{C\text{-}D}$ = 19 Hz, benzylic carbon), 32.6, 31.5, 29.5, 29.3, 24.7. **HRMS (ESI+):** calcd. for $C_{15}H_{23}DO_2Na^{+}$ ($[M+Na]^{+}$) 260.1731, found 260.1728.

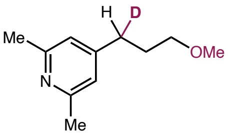

4-(3-methoxypropyl-1-*d*)-2,6-dimethylpyridine (33): According to the general procedure, 4-cyclopropyl-2,6-dimethylpyridine (29.4 mg, 0.2 mmol, 1.0 equiv.), BPP (18.4 mg, 20 μmol, 0.1 equiv.), $(TRIPS)_2$ (9.4 mg, 0.02 mmol, 0.1 equiv.), and 1 mL MeCN/MeOD (v/v = 2/1) was used. Under the irradiation of LEDs (λ_{max} = 400 nm) for 72 h, yellow oil was obtained by flash chromatography (100% petroleum). 72% yield determined by isolation. D-inc.: 77% (determined by HR-MS). **^{1}H NMR (500 MHz, $CDCl_3$):** δ 6.77 (s, 2H), 3.34 (t, *J* =6.5 Hz, 2H), 3.31 (s, 3H), 2.59–2.54 (**m, 1H**), 2.46 (s, 6H), 1.85–1.81 (m, 2H); **^{13}C NMR (126 MHz, $CDCl_3$):** δ 157.5, 151.5 (peak at 151.6 ppm is the aromatic carbon of no-deuterated product), 120.6, 71.6, 58.6, 31.2 (t, $J_{C\text{-}D}$ = 19 Hz, benzylic carbon. Peak at 31.6 ppm is the benzylic carbon of no-deuterated product), 30.2, 24.3. **HRMS (ESI+)**: calcd. for $C_{11}H_{17}DNO^+$ ($[M+H]^+$) 181.1146, found 181.1143.

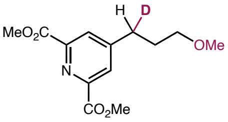

Dimethyl 4-(3-methoxypropyl-1-*d*)pyridine-2,6-dicarboxylate (34): According to the general procedure, dimethyl 4-cyclopropylpyridine-2,6-dicarboxylate (47.0 mg, 0.2 mmol, 1.0 equiv.), BPP (18.4 mg, 20 μmol, 0.1 equiv.), $(TRIPS)_2$ (9.4 mg, 0.02 mmol, 0.1 equiv.), and 1 mL MeCN/MeOD (v/v = 2/1) was used. Under the irradiation of LEDs (λ_{max} = 400 nm) for 48 h, white solid was obtained by flash chromatography (50% ethyl acetate in petroleum). 33% yield determined by isolation. D-inc.: 99% (determined by HR-MS). **^{1}H NMR (500 MHz, $CDCl_3$):** δ 8.16k (s, 2H), 4.02 (s, 6H), 3.40–3.37 (m, 2H), 3.34 (s, 3H), 2.88–2.84 (**m, 1H**), 1.97–1.94 (m, 2H); **^{13}C NMR (100 MHz, $CDCl_3$):** δ 165.4, 154.4, 148.3, 128.4, 71.2, 58.8, 53.3, 31.5 (t, $J_{C\text{-}D}$ = 19 Hz, benzylic carbon. Peak at 31.9 ppm is the benzylic carbon of no-deuterated product), 30.0. **HRMS (FI+)**: calcd. for $C_{13}H_{16}DNO_5^+$ ($[M]^+$) 268.1164, found 268.1161.

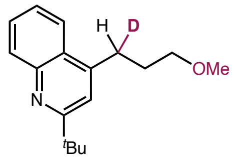

2-(*tert*-Butyl)-4-(3-methoxypropyl-1-*d*)quinoline (35): According to the general procedure, 2-(*tert*-butyl)-4-cyclopropylquinoline (45.1 mg, 0.2 mmol, 1.0 equiv.), BPP (18.4 mg, 20 μmol, 0.1 equiv.), $(TRIPS)_2$ (9.4 mg, 0.02 mmol, 0.1 equiv.), and 1 mL MeCN/MeOD (v/v = 2/1) was used. Under the irradiation of LEDs (λ_{max} = 400 nm) for 148 h, pale yellow oil was obtained by flash chromatography (17% ethyl acetate in petroleum). 45% yield determined by isolation. D-inc.: 84% (determined by HR-MS). **^{1}H NMR (500 MHz, $CDCl_3$):** δ 8.10–8.08 (m, 1H), 7.99 (dd, J = 8.4, 1.3 Hz, 1H), 7.67–7.63 (m, 1H), 7.49–7.46 (m, 1H), 7.38 (s, 1H), 3.44 (t, J = 6.0 Hz, 2H), 3.38 (s, 3H), 3.15–3.12 (**m, 1H**), 2.05–2.00 (m, 2H), 1.47 (s, 9H); **^{13}C NMR (126 MHz, $CDCl_3$):** δ 169.0, 147.8, 130.2, 128.8, 125.9, 125.6, 123.3, 118.2, 100.1, 71.8, 58.8, 38.1, 30.3, 28.7 (t, $J_{C\text{-}D}$ = 19 Hz, benzylic carbon. Peak at 29.1 ppm is the benzylic carbon of no-deuterated product). **HRMS (ESI+)**: calcd. for $C_{17}H_{23}DNO^+$ ($[M+H]^+$) 259.1912, found 259.1915.

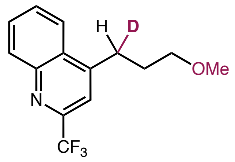

4-(3-Methoxypropyl-1-*d*)-2-(trifluoromethyl)quinoline (36): According to the general procedure, 4-cyclopropyl-2-(trifluoromethyl)quinoline (47.4 mg, 0.2 mmol, 1.0 equiv.), BPP (3.7 mg, 4 μmol, 0.02 equiv.), $(TRIPS)_2$ (9.4 mg, 0.02 mmol, 0.1 equiv.), and 1 mL MeCN/MeOD (v/v = 2/1) was used. Under the irradiation of LEDs (λ_{max} = 400 nm) for 72 h, yellow oil was obtained by flash chromatography (17% ethyl acetate in petroleum). 95% yield determined by isolation. D-inc.: 97% (determined by HR-MS). **^{1}H NMR (400 MHz, $CDCl_3$):** δ 8.24–8.21 (m, 1H), 8.14–8.12 (m, 1H), 7.81–7.77 (m, 1H), 7.69–7.65 (m, 1H), 7.59 (s, 1H), 3.44 (t, J = 6.0 Hz, 2H), 3.38 (s, 3H),

3.27–3.20 (**m, 1H**), 2.10–1.97 (m, 2H); **^{13}C NMR (126 MHz, $CDCl_3$):** δ 151.0, 147.7 (q, $J_{C\text{-}CF3}$ = 34 Hz), 147.4, 131.0, 130.5, 128.4, 128.3, 123.7, 121.8 (q, $J_{C\text{-}F}$ = 276 Hz), 116.6, 71.5, 58.8, 30.1, 28.8 (t, $J_{C\text{-}D}$ = 19 Hz, benzylic carbon); **^{19}F NMR (376 MHz, $CDCl_3$):** δ -67.57 (s); **HRMS (ESI+)**: calcd. for $C_{14}H_{13}DF_3NO^+$ ($[M+H]^+$) 271.1163, found 271.1161.

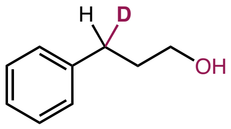

3-Phenylpropan-3-*d*-1-ol(-*d*) (37): According to the general procedure, cyclopropylbenzene (23.6 mg, 0.2 mmol, 1.0 equiv.), BPP (3.7 mg, 4 μmol, 0.02 equiv.), $(TRIPS)_2$ (9.4 mg, 0.02 mmol, 0.1 equiv.), and 1 mL MeCN/D_2O (v/v = 2/1) was used. Under the irradiation of LEDs (λ_{max} = 400 nm) for 16 h, pale yellow oil was obtained by flash chromatography (17% ethyl acetate in petroleum. Hydroxyl deuterium atom undergoes proton exchange during column chromatography.). 60% yield determined by isolation. D-inc.: 97% (determined by HR-MS). **^{1}H NMR (500 MHz, $CDCl_3$):** δ 7.30–7.27 (m, 2H), 7.20–7.17 (m, 3H), 3.68 (t, *J* = 6.5 Hz, 2H), 2.70–2.67 (**m, 1H**), 1.88 (q, *J* = 7.0 Hz, 1H), 1.69 (s, 1H); **^{13}C NMR (126 MHz, $CDCl_3$):** δ 141.89, 128.52, 128.49, 125.95, 62.28, 34.21 31.75 (t, $J_{C\text{-}D}$ = 19 Hz, benzylic carbon). **HRMS (FI+)**: calcd. for $C_9H_{11}DO^+$ ($[M]^+$) 137.0945, found 137.0943.

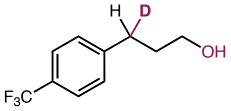

3-(4-(Trifluoromethyl)phenyl)propan-3-*d*-1-ol-*d* (38): According to the general procedure, cyclopropylbenzene (23.6 mg, 0.2 mmol, 1.0 equiv.), BPP (3.7 mg, 4 μmol, 0.02 equiv.), $(TRIPS)_2$ (9.4 mg, 0.02 mmol, 0.1 equiv.), and 1 mL MeCN/D_2O (v/v = 2/1) was used. Under the irradiation of LEDs (λ_{max} = 400 nm) for 16 h, pale yellow oil was obtained by flash chromatography (17% ethyl acetate in petroleum. Hydroxyl

deuterium atom undergoes proton exchange during column chromatography.). 62% yield determined by isolation. D-inc.: 92% (determined by HR-MS). **^{1}H NMR (500 MHz, $CDCl_3$):** δ 7.54–7.53 (m, 2H), 7.31–7.26 (m, 2H), 3.67 (t, J = 6.5 Hz, 2H), 2.77–2.74 (**m, 1H**), 1.90 (q, J = 7.0 Hz, 1H), 1.75 (s, 1H); **^{13}C NMR (126 MHz, $CDCl_3$):** δ 146.1, 128.9, 128.6 (q, $J_{C\text{-}CF3}$ = 33 Hz), 125.4 (q, J = 3.8 Hz), 124.5 (q, $J_{C\text{-}F}$ = 272 Hz), 62.0, 33.9, 31.6 (t, $J_{C\text{-}D}$ = 20 Hz, benzylic carbon), 31.2. **^{19}F NMR (376 MHz, $CDCl_3$):** δ -32.26 (s); **HRMS (FI+)**: calcd. for $C_{10}H_{10}DF_3O^+$ ($[M]^+$) 205.0819, found 205.0818.

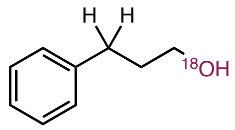

3-Phenylpropan-1-ol-^{18}*O* (39): According to the general procedure, cyclopropylbenzene (23.6 mg, 0.2 mmol, 1.0 equiv.), BPP (3.7 mg, 4 μmol, 0.02 equiv.), $(TRIPS)_2$ (9.4 mg, 0.02 mmol, 0.1 equiv.), and 1 mL MeCN/$H_2{}^{18}O$ (v/v = 2/1) was used. Under the irradiation of LEDs (λ_{max} = 400 nm) for 16 h, pale yellow oil was obtained by flash chromatography (17% ethyl acetate in petroleum). 64% yield determined by GC-FID analysis with PhOMe as internal standard. **^{1}H NMR (400 MHz, $CDCl_3$):** δ 7.30–7.26 (m, 2H), 7.20–7.16 (m, 3H), 3.66 (t, J = 6.4 Hz, 2H), 2.70 (t, J = 7.6 Hz, 2H), 1.92-1.85 (m, 2H); **^{13}C NMR (100 MHz, $CDCl_3$):** δ 142.0, 128.6, 128.5, 126.0, 62.4, 34.3, 32.2. **HRMS (FI+)**: calcd. for $C_9H_{12}{}^{18}O^+$ ($[M]^+$) 138.0925, found 138.0922.

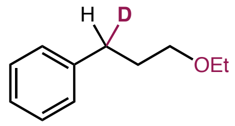

(3-Ethoxypropyl-1-*d*)benzene (40): According to the general procedure, cyclopropylbenzene (23.6 mg, 0.2 mmol, 1.0 equiv.), BPP (3.7 mg, 4 μmol, 0.02 equiv.), $(TRIPS)_2$ (9.4 mg, 0.02 mmol, 0.1 equiv.), and 1 mL MeCN/EtOD (v/v = 2/1) was used. Under the irradiation of LEDs (λ_{max} = 400 nm) for 16 h, volatile colorless oil was

obtained by flash chromatography (2% ethyl acetate in petroleum). 77% yield determined by GC-FID analysis with PhOMe as internal standard. D-inc.: 87% (determined by HR-MS). **^{1}H NMR (400 MHz, CDCl$_3$):** δ 7.30–7.26 (m, 2H), 7.20–7.16 (m, 3H), 3.47 (q, *J* = 7.2 Hz, 2H), 3.42 (t, *J* = 6.8 Hz, 2H), 2.69–2.67 (m, 1H), 1.89 (q, *J* = 8.0 Hz, 1H), 1.21 (t, *J* = 7.2 Hz, 3H), 2.67–2.64 (**m, 1H**), 1.91–1.86 (m, 1H); **^{13}C NMR (100 MHz, CDCl$_3$):** δ 142.2, 128.6, 128.4, 125.9, 69.9, 66.3, 32.2 (t, $J_{C\text{-}D}$ = 20 Hz, benzylic carbon), 31.4, 15.4. **HRMS (FI+)**: calcd. for $C_{11}H_{15}DO^+$ ([M]$^+$) 165.1258, found 165.1260.

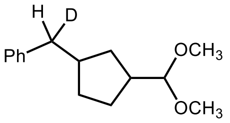

((2-(Dimethoxymethyl)cyclopentyl)methyl-*d*)benzene (41): According to the general procedure, 2-methoxy-3-phenylbicyclo[2.2.1]heptane (40.4 mg, 0.2 mmol, 1.0 equiv.), BPP (3.7 mg, 4 μmol, 0.02 equiv.), (TRIPS)$_2$ (9.4 mg, 0.02 mmol, 0.1 equiv.), and 1 mL MeCN/MeOD (v/v = 2/1) was used. Under the irradiation of LEDs (λ_{max} = 400 nm) for 16 h, colorless oil was obtained by flash chromatography (5% ethyl acetate in petroleum), 50% yield determined by ^{1}H NMR analysis with PhOMe as internal standard, D-inc.: 95% (determined by HR-MS). **^{1}H NMR (400 MHz, CDCl$_3$):** δ 7.29–7.25 (m, 2H), 7.19–7.15 (m, 3H), 4.12 (d, *J* = 8.0 Hz, 1H), 3.32 (s, 3H), 3.30 (s, 3H), 2.62–2.58 (**m, 1H**), 2.29–2.19 (m, 1H), 2.15–2.05 (m, 1H), 1.88–1.81 (m, 1H), 1.76–1.64 (m, 2H), 1.55–1.46 (m, 1H), 1.30–1.19 (m, 1H), 1.07–0.98 (m, 1H); **^{13}C NMR (100 MHz, CDCl$_3$):** δ 142.1, 128.9, 128.3, 125.8, 108.5, 53.2, 52.9, 42.4, 42.0–41.6 (m, benzylic carbon), 41.5, 35.6, 31.8, 27.0. **HRMS (EI+):** calcd. for $C_{15}H_{17}DO^+$ ([M-CH$_3$OH]$^+$) 203.1430, found 203.1426.

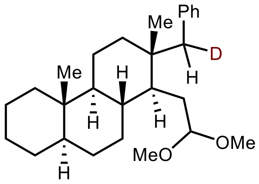

(*1S,2R,4aS,4bS,8aR,10aS*)-1-(2,2-dimethoxyethyl)-2,4b-dimethyl-2-(phenylmethyl-*d*)tetradecahydrophenanthrene (42): According to the general procedure, (*5R,8R,9S,10S,13S,14S*)-16-methoxy-10,13-dimethyl-17-phenylhexadecahydro-1*H*-cyclopentaphenanthrene (73.2 mg, 0.2 mmol, 1.0 equiv.), BPP (3.7 mg, 4 μmol, 0.02 equiv.), $(TRIPS)_2$ (9.4 mg, 0.02 mmol, 0.1 equiv.), and 1 mL MeCN/MeOD (v/v = 2/1) was used. Under the irradiation of LEDs (λ_{max} = 400 nm) for 16 h, white powder was obtained by flash chromatography (5% ethyl acetate in petroleum), 62% yield determined by ^{1}H NMR analysis with PhOMe as internal standard, D-inc.: 95% (determined by HR-MS). **^{1}H NMR (400 MHz, $CDCl_3$):** δ 7.25–7.21 (m, 2H), 7.19–7.16 (m, 1H), 7.15–7.12 (m, 2H), 4.42 (dd, *J* = 8.0, 2.8 Hz, 1H), 3.39 (s, 3H), 3.37 (s, 3H), 2.31–2.20 **(m, 1H)**, 1.97–1.88 (m, 2H), 1.68–1.63 (m, 2H), 1.50–1.45 (m, 2H), 1.44–1.41 (m, 1H), 1.39–1.37 (m, 1H), 1.36–1.33 (m, 1H), 1.32–1.28 (m, 2H), 1.25–1.10 (m, 6H), 1.06–1.01 (m, 1H), 0.99–0.94 (m, 2H), 0.92–0.85 (m, 1H), 0.83–0.78 (m, 1H), 0.71 (s, 6H); **^{13}C NMR (100 MHz, $CDCl_3$):** δ 139.39, 131.15, 131.13, 127.54, 125.66, 107.10, 54.88, 54.17, 52.98, 48.40, 48.38, 46.76, 39.70, 38.60, 38.25, 37.56–37.40 (m, benzylic carbon), 36.54, 34.53, 32.85, 29.49, 29.01, 26.90, 22.23, 20.01, 17.40, 17.38, 12.37. **HRMS (ESI+):** calcd. for $C_{27}H_{41}DO_2Na^+$ ($[M+Na]^+$) 422.3140, found 422.3135.

6.2 Failed trials

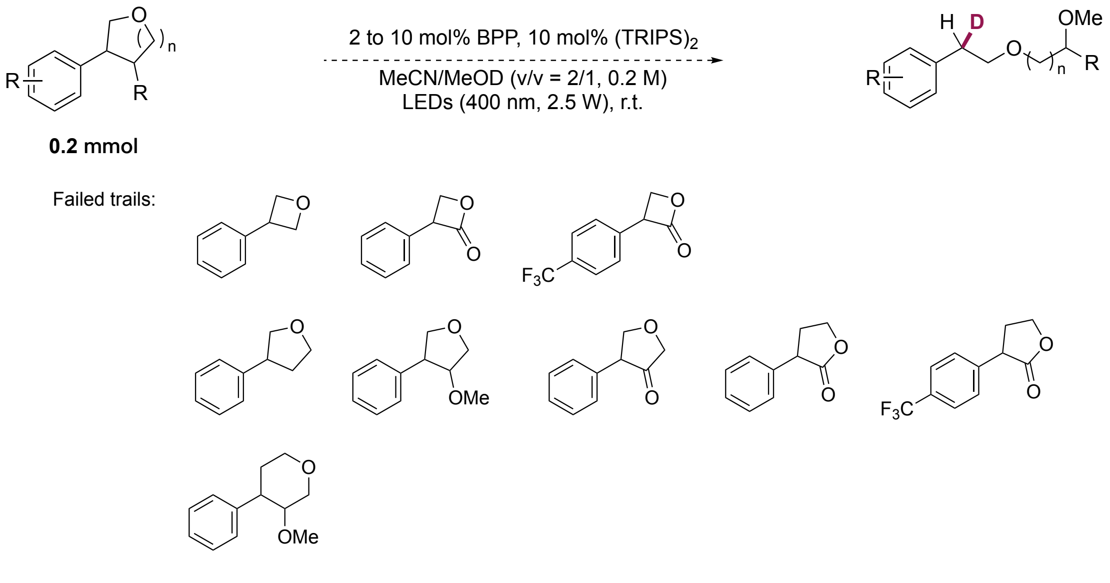

Figure S24. Failed trials. Conditions: starting material 0.2 mmol, BPP 0.02 equiv., $(TRIPS)_2$ 0.1

equiv. in 1.0 mL MeCN/MeOD (v/v = 2/1) under N_2 atmosphere, LEDs (λ_{max} = 400 nm, 2.5 W).

7. Experimental data for derivatives

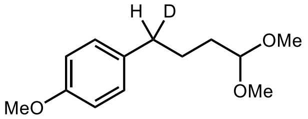

1-(4,4-Dimethoxybutyl-1-*d*)-4-methoxybenzene: According to the general procedure, 1-methoxy-4-(2-methoxycyclobutyl)benzene (38.4 mg, 0.2 mmol, 1.0 equiv.), BPP (3.7 mg, 4 μmol, 0.02 equiv.), $(TRIPS)_2$ (9.4 mg, 0.02 mmol, 0.1 equiv.), and 1 mL MeCN/MeOD (v/v = 2/1) was used. Under the irradiation of LEDs (λ_{max} = 400 nm) for 16 h, colorless oil was obtained by flash chromatography (5% ethyl acetate in petroleum), 82% yield determined by ^{1}H NMR analysis with PhOMe as internal standard, D-inc.: 95% (determined by HR-MS). **^{1}H NMR (400 MHz, $CDCl_3$):** δ 7.12–7.09 (m, 2H), 6.85–6.81 (m, 2H), 4.39–4.36 (m, 1H), 3.78 (s, 3H), 3.30 (s, 6H), 2.60–2.54 (**m, 1H**), 1.68–1.59 (m, 4H). **^{13}C NMR (100 MHz, $CDCl_3$):** δ 157.8, 134.3, 129.4, 113.8, 104.5, 55.3, 52.7, 34.4 (t, $J_{C\text{-}D}$ = 20 Hz, benzylic carbon), 32.1, 26.6. **HRMS (EI+):** calcd. for $C_{12}H_{15}DO_2^+$ ([M-MeOH]$^+$) 193.1208, found 193.1205.

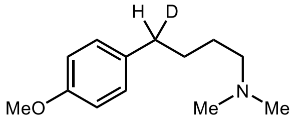

4-(4-Dethoxyphenyl)-*N*,*N*-dimethylbutan-1-amine-4-*d*[11]: A 8 mL vial was charged with 1-(4,4-dimethoxybutyl-1-*d*)-4-methoxybenzene (43.0 mg, 0.2 mmol, 1.0 equiv.) in 1 mL acetone, then HCl (0.2 mL, 2 M in water, 2.0 equiv.) was added. The reaction was stirred at 50 °C for 1 h. The reaction mixture was dried over Na_2SO_4, filtered and concentrated in *vacuo*. Then titanium (IV) isopropoxide (108 μL, 0.4 mmol, 2.0 equiv.) and a solution of dimethylamine in THF (0.2 mL, 2 M, 2.0 equiv.), 2 mL MeOH was added. The reaction mixture was stirred at ambient temperature for 5 h, and sodium borohydride (7.6 mg, 0.2 mmol, 1.0 equiv.) was added and the resulting mixture was further stirred for another period of 1.5 h. The reaction was then quenched by the addition of water, the resulting inorganic precipitate was filtered, washed with ethyl acetate and the aqueous filtrate was extracted with ethyl acetate. The combined extracts

were dried (Na_2SO_4) and concentrated *in vacuo* to give a white powder (38.3 mg, 92% yield), D-inc.: 95% (determined by HR-MS). **^{1}H NMR (400 MHz, $CDCl_3$):** δ 7.07–7.05 (m, 2H), 6.82–6.80 (m, 2H), 3.77 (s, 3H), 2.85–2.81 (m, 2H), 2.65 (s, 6H), 2.59–2.55 (**m, 1H**), 1.83–1.75 (m, 2H), 1.66–1.61 (m, 2H). **^{13}C NMR (100 MHz, $CDCl_3$):** δ 157.8, 134.7, 129.4, 113.8, 59.9, 55.3, 45.6, 34.7 (t, $J_{C\text{-}D}$ = 19 Hz, benzylic carbon), 29.6, 27.4. **HRMS (EI+):** calcd. for $C_{13}H_{20}DNO^+$ ($[M]^+$) 208.1680, found 208.1678.

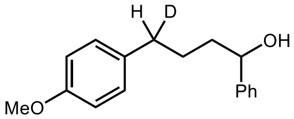

4-(4-Dethoxyphenyl)-1-phenylbutan-4-*d*-1-ol[12]: A 8 mL vial was charged with 1-(4,4-dimethoxybutyl-1-*d*)-4-methoxybenzene (43.0 mg, 0.2 mmol, 1.0 equiv.) in 1 mL acetone, HCl (0.2 mL, 2 M in water, 2.0 equiv.) was added. The reaction was stirred at 50 °C for 1 h. The reaction mixture was dried over Na_2SO_4, filtered and concentrated in *vacuo*, then a solution of 1.0 M PhMgBr (0.3 mL, 0.3 mmol, 1.5 equiv.) in 2 mL THF was added. The reaction mixture was stirred at ambient temperature for 5 h. The reaction was quenched with saturated NH_4Cl water solution and separated. The aqueous phase was extracted with diethyl ether three times, washed with brine and dried over Na_2SO_4. The organic phase was concentrated and subjected to flash chromatography (10% ethyl acetate in petroleum), a colorless oil was obtained (39.8 mg, 80% yield), D-inc.: 95% (determined by HR-MS). **^{1}H NMR (400 MHz, $CDCl_3$):** δ 7.35–7.25 (m, 5H), 7.08–7.04 (m, 2H), 6.82–6.78 (m, 2H), 4.68–4.66 (m, 1H), 3.77 (s, 3H), 2.59–2.53 (**m, 1H**), 1.86–1.79 (m, 2H), 1.76–1.71 (m, 2H). **^{13}C NMR (100 MHz, $CDCl_3$):** δ 157.8, 144.9, 134.4, 129.4, 128.6, 127.7, 126.0, 113.9, 74.7, 55.4, 38.6, 34.6 (t, $J_{C\text{-}D}$ = 19 Hz, benzylic carbon), 27.8. **HRMS (EI+):** calcd. for $C_{17}H_{17}DO^+$ ($[M\text{-}H_2O]^+$) 239.1415, found 239.1415.

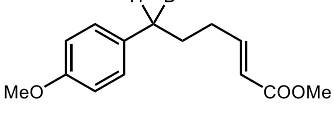

Methyl (*E*)-6-(4-methoxyphenyl)hex-2-enoate-6-*d*[13]: A 8 mL vial was charged with 1-(4,4-dimethoxybutyl-1-*d*)-4-methoxybenzene (43.0 mg, 0.2 mmol, 1.0 equiv.) in 1 mL acetone, HCl (0.2 mL, 2 M in water, 2.0 equiv.) was added. The reaction was stirred at 50 °C for 1 h. The reaction mixture was dried over Na_2SO_4, filtered and concentrated in *vacuo*, after which (carbomethoxymethylene) triphenyl phosphorane (133.6 mg, 0.4 mmol, 2.0 equiv.) and 2 mL toluene were added. The reaction mixture was stirred under an argon atmosphere at 80 °C for 4 h. The reaction mixture was concentrated under reduced pressure. The residue was purified by flash chromatography (10% ethyl acetate in petroleum), a colorless oil was obtained (47.0 mg, 81% yield), D-inc.: 95% (determined by HR-MS). **^{1}H NMR (400 MHz, $CDCl_3$):** δ 7.10–7.06 (m, 2H), 7.02–6.95 (m, 1H), 6.85–6.81 (m, 2H), 5.86–5.81 (m, 1H), 3.79 (s, 3H), 3.73 (s, 3H), 2.60–2.54 (**m, 1H**), 2.25–2.19 (m, 2H), 1.75 (q, J = 7.6 Hz, 2H). **^{13}C NMR (100 MHz, $CDCl_3$):** δ 167.2, 158.0, 149.3, 133.8, 129.4, 121.3, 113.9, 55.4, 51.5, 34.0 (t, $J_{C\text{-}D}$ = 19 Hz, benzylic carbon), 31.6, 29.9. **HRMS (EI+):** calcd. for $C_{14}H_{17}DO_3^+$ ($[M]^+$) 235.1313, found 235.1311.

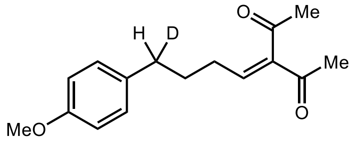

3-(4-(4-Dethoxyphenyl)butylidene-4-*d*)pentane-2,4-dione[14]: A 8 mL vial was charged with 1-(4,4-dimethoxybutyl-1-*d*)-4-methoxybenzene (43.0 mg, 0.2 mmol, 1.0 equiv.), acetylacetone (20.0 mg, 0.2 mmol, 1.0 equiv.), $Mg(ClO_4)_2$ (4.5 mg, 0.02 mmol, 0.1 equiv.), $MgSO_4$ (4.8 mg, 0.04 mmol, 0.2 equiv.) in 0.2 mL THF. The reaction mixture was stirred under an argon atmosphere at 50 °C for 12 h. The reaction mixture was concentrated under reduced pressure. The residue was purified by flash chromatography (10% ethyl acetate in petroleum), a colorless oil was obtained (23.5 mg, 45% yield), D-inc.: 95% (determined by HR-MS). **^{1}H NMR (400 MHz, $CDCl_3$):** δ 7.07 (d, J = 8.8 Hz, 2H), 6.82 (d, J = 8.8 Hz, 2H), 6.62 (t, J = 7.6 Hz, 1H), 3.77 (s, 3H), 2.61–2.56 (**m, 1H**), 2.28–2.22 (m, 8H), 1.78 (q, J = 7.6 Hz, 2H). **^{13}C NMR (100 MHz, $CDCl_3$):** δ 203.6, 197.2, 158.0, 146.6, 145.2, 133.3, 129.4, 113.9, 55.3, 34.2 (t,

$J_{C\text{-}D}$ = 20 Hz, benzylic carbon), 31.7, 30.5, 29.2, 26.1. **HRMS (ESI+):** calcd. for $C_{16}H_{19}DO_3Na^+$ ($[M+Na]^+$) 284.1367, found 285.1370.

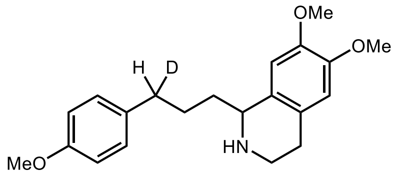

6,7-Dimethoxy-1-(3-(4-methoxyphenyl)propyl-3-*d*)-1,2,3,4-tetrahydroisoquinoline[15]: A 8 mL vial was charged with 1-(4,4-dimethoxybutyl-1-*d*)-4-methoxybenzene (43.0 mg, 0.2 mmol, 1.0 equiv.), 3,4-dimethoxyphenethylamine (43.4 mg, 0.24 mmol, 1.2 equiv.) in 1 mL formic acid. The reaction mixture was stirred at 70 °C for 10 h. The reaction mixture was concentrated under reduced pressure. The residue was purified by flash chromatography (10% methanol in dichloromethane), a white solid was obtained (34.0 mg, 47% yield), D-inc.: 95% (determined by HR-MS). **^{1}H NMR (400 MHz, CDCl$_3$):** δ 8.02 (br, 1H), 7.11 (d, J = 8.4 Hz, 2H), 6.80 (d, J = 8.4 Hz, 2H), 6.55 (s, 1H), 6.39 (s, 1H), 4.33 (t, J = 5.2 Hz, 1H), 3.83 (s, 3H), 3.75 (s, 6H), 3.47-3.41 (m, 1H), 3.21-3.15 (m, 1H), 3.10–3.02 (m, 1H), 2.93–2.86 (m, 1H), 2.69–2.54 (m, 1H), 2.05–1.84 (m, 4H). **^{13}C NMR (100 MHz, CDCl$_3$):** δ 158.0, 148.5, 148.2, 133.5, 129.6, 125.0, 124.7, 113.9, 111.5, 109.0, 56.1, 56.0, 55.3, 54.8, 40.0, 34.3–33.9 (m, deuterated benzylic carbon, overlaps with C1'), 27.0, 26.0. **HRMS (ESI+):** calcd. for $C_{21}H_{27}DNO_3^+$($[M+H]^+$)343.2127, found 343.2125.

8. Experimental data for starting materials

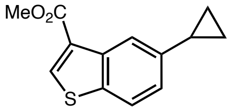

Methyl 5-cyclopropylbenzo[b]thiophene-3-carboxylate: According to the general procedure for the preparation of arylcyclopropanes, the residue was purified by flash chromatography (17% ethyl acetate in petroleum), a waxy yellow solid was obtained (1.7 g, 99% yield). **^{1}H NMR (500 MHz, $CDCl_3$):** δ 7.98 (s, 1H), 7.72 (d, *J* = 8.5 Hz, 1H), 7.57(s, 1H), 7.19–7.17 (m, 1H), 3.94 (s, 3H), 2.03–2.00 (m, 1H), 1.04–1.00 (m, 2H), 0.76–0.73 (m, 2H); **^{13}C NMR (126 MHz, $CDCl_3$):** δ 163.5, 141.2, 139.6, 139.1, 133.6, 130.5, 126.0, 122.6, 122.2, 52.6, 15.5, 9.4 .

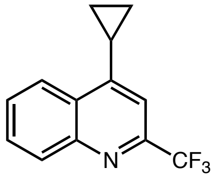

4-Cyclopropyl-2-(trifluoromethyl)quinoline: According to the general procedure for the preparation of arylcyclopropanes, the residue was purified by flash chromatography (17% ethyl acetate in petroleum), a waxy yellow solid was obtained (1.1 g, 59% yield). **^{1}H NMR (400 MHz, $CDCl_3$):** 8.40–8.37 (m, 1H), 8.24–8.21 (m, 1H), 7.83–7.78 (m, 1H), 7.72–7.68 (m, 1H), 7.34 (s, 1H), 2.53–2.46 (m, 1H), 1.28–1.23 (m, 2H), 0.96–0.91 (m, 2H); **^{13}C NMR (126 MHz, $CDCl_3$):** δ 152.6, 148.0 (q, *J* = 34 Hz), 147.1, 130.8, 130.5, 129.5, 128.3, 124.1, 121.8 (q, *J* = 276 Hz), 112.8 (q, *J* = 2.5 Hz), 12.6, 8.5. **^{19}F NMR (376 MHz, $CDCl_3$):** δ -37.5(s);

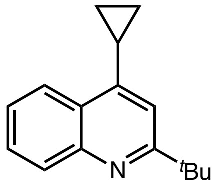

2-(tert-butyl)-4-cyclopropylquinoline: According to the general procedure for the

preparation of arylcyclopropanes, the residue was purified by flash chromatography (17% ethyl acetate in petroleum), a waxy yellow solid was obtained (1.0 g, 40% yield). **^{1}H NMR (400 MHz, CDCl$_3$):** δ 8.31–8.24 (m, 1H), 8.13–8.11 (m, 1H), 7.71–7.67 (m, 1H), 7.56–7.52 (m, 1H), 7.24 (s, 1H), 2.43–2.37 (m, 1H), 1.49 (s, 9 H),1.18–1.14 (m, 2H), 0.89–0.85 (m, 2H); **^{13}C NMR (126 MHz, CDCl$_3$):** δ 169.1, 148.7, 147.5, 130.0, 128.8, 127.2, 125.4, 123.7, 114.5, 38.2, 30.3, 12.6, 7.4.

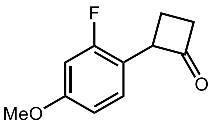

2-(2-Fluoro-4-methoxyphenyl)cyclobutan-1-one: according to the general procedure for the preparation of arylcyclobutanone, the residue was purified by flash chromatography (10% ethyl acetate in petroleum), a colorless oil was obtained (2.0 g, 40% yield). **^{1}H NMR (400 MHz, CDCl$_3$):** δ 7.11 (t, J = 8.8 Hz, 1H), 6.65–6.59 (m, 2H), 4.55–4.50 (m, 1H), 3.77 (s, 3H), 3.26–3.16 (m, 1H), 3.11–3.04 (m, 1H), 2.56–2.46 (m, 1H), 2.20–2.10 (m, 1H). **^{13}C NMR (100 MHz, CDCl$_3$):** δ 207.8, 161.4 (d, $J_{C\text{-}F}$ = 229 Hz), 160.2 (d, $J_{C\text{-}F}$ = 5 Hz), 129.6 (d, $J_{C\text{-}F}$ = 7 Hz), 116.4 (d, J = 16 Hz), 109.9, 102.0 (d, $J_{C\text{-}F}$ = 26 Hz), 59.1, 55.7 (d, J = 3 Hz), 45.2, 18.7. **^{19}F NMR (376 MHz, CDCl$_3$)** δ -113.83–(-113.89).

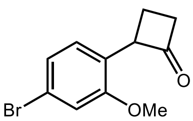

2-(4-Bromo-2-methoxyphenyl)cyclobutan-1-one: according to the general procedure for the preparation of arylcyclobutanone, the residue was purified by flash chromatography (10% ethyl acetate in petroleum), a colorless oil was obtained (3.2 g, 66% yield). **^{1}H NMR (400 MHz, CDCl$_3$):** δ 7.02–6.96 (m, 3H), 4.47–4.41 (m, 1H), 3.77 (s, 3H), 3.18–3.03 (m, 2H), 2.44–2.35 (m, 1H), 2.19–2.09 (m, 1H). **^{13}C NMR (100 MHz, CDCl$_3$):** δ 208.3, 157.9, 130.2, 125.1, 123.6, 121.6, 114.5, 61.0, 55.8, 45.0, 18.1.

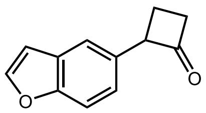

2-(Benzofuran-5-yl)cyclobutan-1-one: according to the general procedure for the preparation of arylcyclobutanone**,** the residue was purified by flash chromatography (10% ethyl acetate in petroleum), a white solid was obtained (1.56 g, 51% yield). **^{1}H NMR (400 MHz, CDCl$_3$):** δ 7.61 (m, 1H), 7.50–7.46 (m, 2H), 7.18–7.16 (m, 1H), 6.74–6.73 (m, 1H), 4.65–4.59 (m, 1H), 3.30–3.20 (m, 1H), 3.10–3.00 (m, 1H), 2.64–2.53 (m, 1H), 2.31–2.21 (m, 1H). **^{13}C NMR (100 MHz, CDCl$_3$):** δ 208.4, 154.1, 145.5, 131.2, 127.8, 123.5, 119.3, 111.5, 106.6, 64.5, 44.8, 18.4.

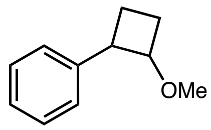

(2-methoxycyclobutyl)benzene: According to the general procedure for the preparation of arylcycloether**,** the residue was purified by flash chromatography (8% ethyl acetate in petroleum), a colorless oil was obtained (0.30 g, 94% yield). **^{1}H NMR (500 MHz, CDCl$_3$):** δ 7.32–7.25 (m, 4H), 7.22–7.18 (m, 1H), 3.87 (q, *J* = 8.0 Hz, 1H), 3.34 (q, *J* = 9.0 Hz, 1H), 3.24 (s, 3H), 2.26–2.20 (m, 1H), 2.18–2.11 (m, 1H), 1.19–1.81 (m, 1H), 1.67–1.59 (m, 1H); **^{13}C NMR (126 MHz, CDCl$_3$):** δ 143.5, 128.5, 126.8, 126.4, 80.9, 55.8, 49.0, 26.4, 19.7.

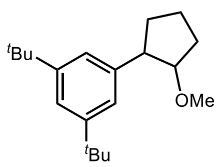

1,3-*di-tert*-Butyl-5-(2-methoxycyclopentyl)benzene: according to the general procedure for the preparation of arylcycloether**,** the residue was purified by flash chromatography (8% ethyl acetate in petroleum), a colorless oil was obtained (0.29 g, 90% yield). **^{1}H NMR (400 MHz, CDCl$_3$):** δ 7.28–7.27 (m, 1H), 7.10 (d, *J* = 1.6 Hz, 2H), 3.84–3.80 (m, 1H), 3.28 (s, 3H), 3.06–3.00 (m, 1H), 2.21–2.13 (m, 1H), 2.04–1.96

(m, 1H), 1.89–1.75 (m, 3H), 1.72–1.64 (m, 1H), 1.33 (s, 18H). **^{13}C NMR (100 MHz, $CDCl_3$):** δ 150.6, 143.8, 121.6, 120.2, 89.4, 57.3, 52.7, 35.0, 33.4, 31.8, 31.7, 23.4.

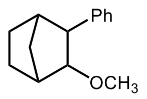

2-Methoxy-3-phenylbicyclo[2.2.1]heptane: according to the preparation of 2-methoxy-3-phenylbicyclo[2.2.1]heptane**,** the residue was purified by flash chromatography (8% ethyl acetate in petroleum), a colorless oil was obtained (0.13 g, 45% yield). **^{1}H NMR (400 MHz, $CDCl_3$):** δ 7.34–7.30 (m, 2H), 7.23–7.20 (m, 3H), 3.49 (s, 1H), 3.30 (s, 3H), 2.99–2.97 (m, 1H), 2.46–2.41 (m, 2H), 1.86–1.83 (m, 1H), 1.68–1.58 (m, 1H), 1.40–1.36 (m, 1H), 1.30–1.18 (m, 2H), 1.15–1.11 (m, 1H). **^{13}C NMR (100 MHz, $CDCl_3$):** δ 141.8, 128.3, 128.1, 126.0, 88.2, 56.5, 56.1, 41.8, 41.3, 37.4, 25.3, 22.2.

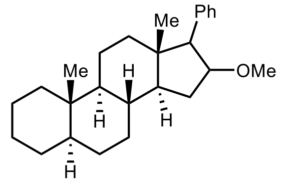

(*5R,8R,9S,10S,13S,14S*)-16-methoxy-10,13-dimethyl-17-phenylhexadecahydro-1*H*-cyclopenta[*a*]phenanthrene: according to the general procedure for the preparation of arylcycloether**,** the residue was purified by flash chromatography (8% ethyl acetate in petroleum), a white solid was obtained (0.30 g, 78% yield). **^{1}H NMR (400 MHz, $CDCl_3$):** δ 7.31–7.27 (m, 2H), 7.23–7.21 (m, 3H), 4.36–4.31 (m, 1H), 3.19 (s, 3H), 2.67 (d, J = 7.2 Hz, 1H), 1.84–1.78 (m, 1H), 1.75–1.62 (m, 1H), 1.57–1.47 (m, 4H), 1.42–1.34 (m, 2H), 1.29–1.17 (m, 7H), 1.12–1.03 (m, 2H), 1.01–0.93 (m, 1H), 0.91–0.85 (m, 1H),0.77 (s, 3H), 0.49 (s, 3H). **^{13}C NMR (100 MHz, $CDCl_3$):** δ 139.9, 128.7, 128.0, 126.3, 84.6, 65.6, 57.7, 55.1, 54.1, 47.2, 45.1, 38.7, 37.9, 36.5, 35.7, 32.5, 32.2, 29.2, 29.1, 26.9, 22.3, 20.2, 14.4, 12.4.

9. Continuous-flow synthesis

9.1 The continuous-flow reaction set-up

A homemade flow-photoreactor was used for each single photoredox reactions. The reaction mixture was fed into a FEP coil (1/32' – 1/16', 36 m) by a peristaltic pump. The LED chips were cooled with continuous water flow, the heating effect of the high-power LEDs can be offset and the reaction temperature can be maintained at ambient temperature. Through this design, identical irradiation intensity and ambient temperature can be guaranteed for each reaction to ensure data consistency.

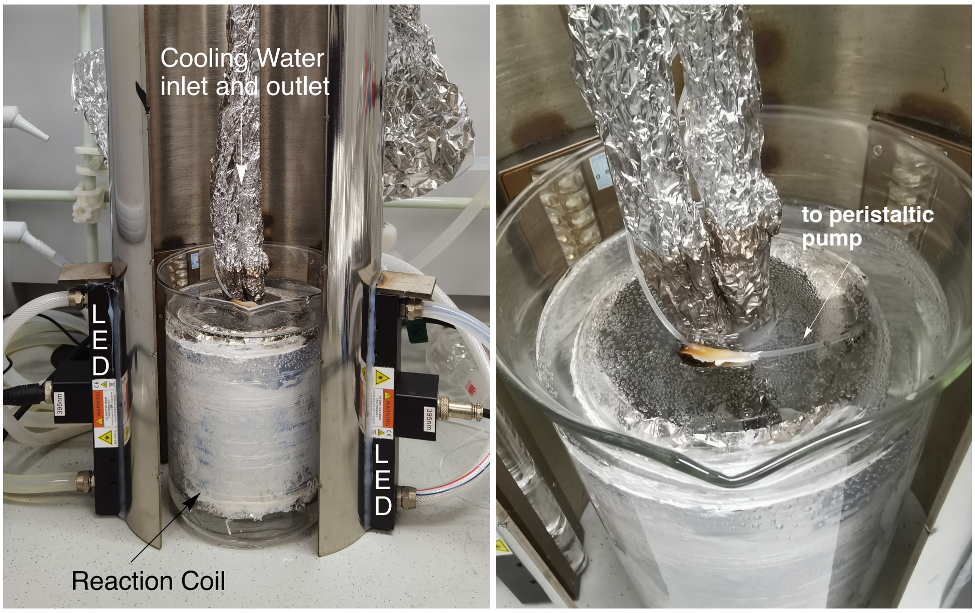

Figure S25. The continuous-flow reaction set-up. Left: main view of the photo reactor; right: close-up of the coil.

9.2 The continuous-flow synthesis

Table S3. Evaluation of different flow conditions

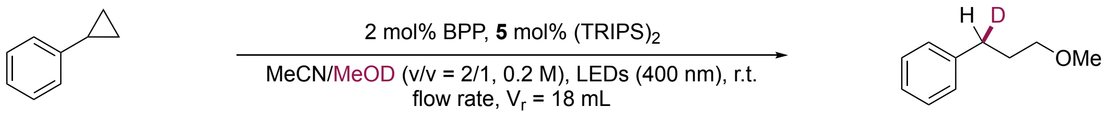

**Entry**	**BPP** (mol%)	**$(TRIPS)_2$** (mol%)	**MeOD amount** (equiv.)	**Flow rate** (mL/min)	**Yield**[a] (%)	**STY** (g/L•h)
1	2	5	40 (v/v = 2/1)	1.0	43	43.3
2	2	5	40 (v/v = 2/1)	0.5	51	25.7
3	2	5	40 (v/v = 2/1)	0.3	76	23.0
**4**	**2**	**5**	**40 (v/v = 2/1)**	**0.2**	**87**	**17.4**
5	2	5	40 (v/v = 2/1)	0.1	86	8.7
6	2	5	40 (v/v = 2/1)	batch	38	0.7
7	2	2	40 (v/v = 2/1)	0.2	10	2.0
8	1	5	40 (v/v = 2/1)	0.2	trace	-
9	2	5	20	0.2	68	13.6

[a] Determined by GC-FID with anisole as aninternal standard.

A representative procedure for the continuous-flow synthesis is shown as follows.

A 100 mL two-neck round-bottom flask was loaded with cyclopropylbenzene (10 mmol, 1.0 equivalent), BPP (0.02 equivalent), $(TRIPS)_2$ (0.05 equivalent), and a solvent mixture of 50 mL MeCN/MeOD (volume ratio 2:1). The reaction mixture underwent degasification by sparging with argon for a duration of 10 minutes to remove dissolved gases. Subsequently, the mixture was introduced into a reaction coil and exposed to irradiation from LEDs (λ_{max} = 400 nm), with a photon flux of 12 W/cm², at a flow rate of 0.2 mL/min. After the complete consumption of the solution, an additional 50 mL of acetonitrile was circulated through the coil at the established flow rate to ensure the collection of all reaction products. To the collected mixture, 500 μL of anisole was introduced as an internal standard. The conversion and yield of the reaction were then determined using gas chromatography with a GC-FID analysis.

10. Experimental data using various deuterated nucleophiles

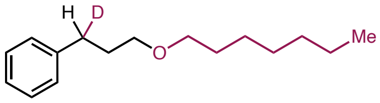

(3-(heptyloxy)propyl-1-*d*)benzene (R1): According to the general procedure, cyclopropylbenzene (23.6 mg, 0.2 mmol, 1.0 equiv.), BPP (3.7 mg, 4 μmol, 0.02 equiv.), 1,2-bis(2,4,6-trichlorophenyl)disulfane (8.5 mg, 0.02 mmol, 0.1 equiv.), and 1 mL MeCN/heptan-1-ol-*d* (v/v = 2/1) was used. Under the irradiation of LEDs (λ_{max} = 400 nm) for 16 h, yellow oil was obtained by flash chromatography (5% ethyl acetate in petroleum). 73% yield determined by isolation. D-inc.: 57% (determined by HR-MS). **^{1}H NMR (500 MHz, $CDCl_3$):** δ 7.31–7.28 (m, 2H), 7.22–7.18 (m, 3H), 3.43 (q, *J* = 6.5 Hz, 4H), 2.73–2.69 (**m, 1H**, no-deuterated product contaminated), 1.94–1.89 (m, 2H), 1.63–1.57 (m, 2H), 1.38–1.27 (m, 9H), 0.92–0.89 (m, 3H); **^{13}C NMR (126 MHz, $CDCl_3$):** δ 142.2, 128.6, 128.4, 125.8, 71.1, 70.0, 32.5, 32.0, 31.5 (benzylic carbon, peak at 31.4 ppm belongs to the no-deuterated product), 29.9, 29.3, 26.3, 22.8, 14.2. **HRMS (FI+)**: calcd. for $C_{16}H_{25}DO^{+}$ ($[M]^{+}$) 235.2041, found 235.2038.

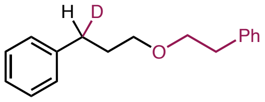

dimethyl 4-(3-methoxypropyl-1-*d*)pyridine-2,6-dicarboxylate (R2): According to the general procedure, dimethyl 4-cyclopropylpyridine-2,6-dicarboxylate (47.0 mg, 0.2 mmol, 1.0 equiv.), BPP (18.4 mg, 20 μmol, 0.1 equiv.), 1,2-bis(2,4,6-trichlorophenyl)disulfane (8.5 mg, 0.02 mmol, 0.1 equiv.), and 1 mL MeCN/2-phenylethan-1-ol-*d* (v/v = 2/1) was used. Under the irradiation of LEDs (λ_{max} = 400 nm) for 72 h, yellow oil was obtained by flash chromatography (2% ethyl acetate in petroleum). 70% yield determined by isolation. D-inc.: 71% (determined by HR-MS). **^{1}H NMR (500 MHz, $CDCl_3$):** δ 7.30–7.26 (m, 4H), 7.25–7.20 (m, 3H), 7.19–7.16 (m, 1H), 7.16–7.13 (m, 2H), 3.62 (t, *J* = 7.0 Hz, 2H), 3.43 (t, *J* = 6.0 Hz, 2H), 2.89 (t, *J* = 7.0 Hz, 2H), 2.67–2.64 (**m, 1H**), 1.91–1.86 (m, 2H); **^{13}C NMR (100 MHz, $CDCl_3$):** δ

142.2, 139.3, 129.1, 128.6, 128.5, 128.4, 126.3, 125.9, 71.9, 70.1, 36.6, 32.1 (t, $J_{C\text{-}D}$ = 20 Hz, benzylic carbon, peak at 32.4 ppm belongs to the no-deuterated product), 31.4 (peak at 31.3 ppm belongs to the no-deuterated product). **HRMS (FI+)**: calcd. for $C_{17}H_{19}DO^{+}$ ($[M]^{+}$) 241.1571, found 241.1566.

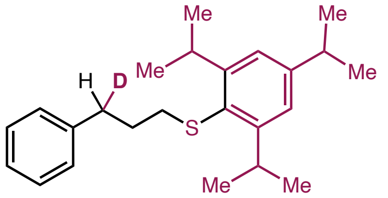

(3-phenylpropyl-3-*d*)(2,4,6-triisopropylphenyl)sulfane (R3): According to the general procedure, 2,4,6-triisopropylbenzenethiol-*d* (47.4 mg, 0.2 mmol, 1.0 equiv.), cyclopropylbenzene (118.2 mg, 1.0 mmol, 5.0 equiv.), BPP (3.7 mg, 4 μmol, 0.02 equiv.), and 1 mL MeCN was used. Under the irradiation of LEDs (λ_{max} = 400 nm) for 16 h, pale yellow solid was obtained by flash chromatography (100% petroleum). 33% yield determined by isolation. D-inc.: 52% (determined by HR-MS). **^{1}H NMR (400 MHz, CDCl$_3$):** δ 7.28–7.24 (m, 2H), 7.20–7.14 (m, 3H), 6.99 (s, 2H), 3.92 (hept, *J* = 6.8 Hz, 2H), 2.87 (hept, *J* = 6.8 Hz, 1H), 2.74–2.71 (**m, 1H**, no-deuterated product contaminated), 2.61 (t, *J* = 6.8 Hz, 2H), 1.91 (p, *J* = 7.2 Hz, 2H), 1.24 (d, *J* = 7.2 Hz, 6H), 1.21 (d, *J* = 7.2 Hz, 12H); **^{13}C NMR (126 MHz, CDCl$_3$):** δ 153.1, 149.4, 141.6, 128.9, 128.6, 128.5, 126.0, 121.8, 37.4, 35.1, 34.4, 31.6, 31.1, 24.6, 24.1. **HRMS (FI+)**: calcd. for $C_{24}H_{33}DS^{+}$ ($[M]^{+}$) 355.2439, found 355.2443.

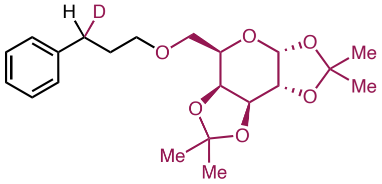

(3a*R*,5*R*,5a*S*,8a*S*,8b*R*)-2,2,7,7-tetramethyl-5-((3-phenylpropoxy-3-*d*)methyl)tetrahydro-5*H*-bis([1,3]dioxolo)[4,5-*b*:4',5'-*d*]pyran (R4): According to the general procedure, cyclopropylbenzene (11.8 mg, 0.1 mmol, 1.0 equiv.), ((3a*R*,5*R*,5a*S*,8a*S*,8b*R*)-2,2,7,7-tetramethyltetrahydro-5*H*-bis([1,3]dioxolo)[4,5-*b*:4',5'-

d]pyran-5-yl)methanol-*d* (130.5 mg, 0.5 mmol, 5.0 equiv.), BPP (1.8 mg, 2 μmol, 0.02 equiv.), 1,2-bis(2,4,6-trichlorophenyl)disulfane (4.3 mg, 0.01 mmol, 0.1 equiv.), and 1 mL MeCN was used. Under the irradiation of LEDs (λ_{max} = 400 nm) for 60 h, colorless oil was obtained by flash chromatography (17% ethyl acetate in petroleum). 33% yield determined by isolation. D-inc.: 38% (determined by HR-MS). **^{1}H NMR (500 MHz, $CDCl_3$):** δ 7.27–7.26 (m, 2H, peak of $CDCl_3$ contaminated), 7.21–7.16 (m, 3H), 5.54 (d, *J* = 5.0 Hz, 1H), 4.61 (dd, *J* = 8.0, 2.5 Hz, 1H), 4.31 (dd, *J* = 5.0, 2.0 Hz, 1H), 4.28 (dd, *J* = 8.0, 2.0 Hz, 1H), 3.98 (td, *J* = 6.0, 2.0 Hz, 1H), 3.67–3.58 (m, 2H), 3.55–3.45 (m, 2H), 2.70–2.67 (**m, 1H**, no-deuterated product contaminated), 1.93–1.87 (m, 2H), 1.62 (s, 2H), 1.54 (s, 3H), 1.46 (s, 3H), 1.35–1.34 (m, 6H); **^{13}C NMR (126 MHz, $CDCl_3$):** δ 142.21, 128.67, 128.42, 125.85, 109.35, 108.68, 100.13, 96.52, 71.32, 70.79, 70.77, 70.62, 69.46, 66.82, 32.36, 31.35 (benzylic carbon, peak at 31.28 ppm belongs to the no-deuterated product), 26.23, 26.14, 25.10, 24.59. **HRMS (FI+)**: calcd. for $C_{21}H_{29}DO_6^+$ ([M]$^+$) 379.2100, found 379.21058.

11. Spectral data

^{1}H NMR 400 MHz, $CDCl_3$

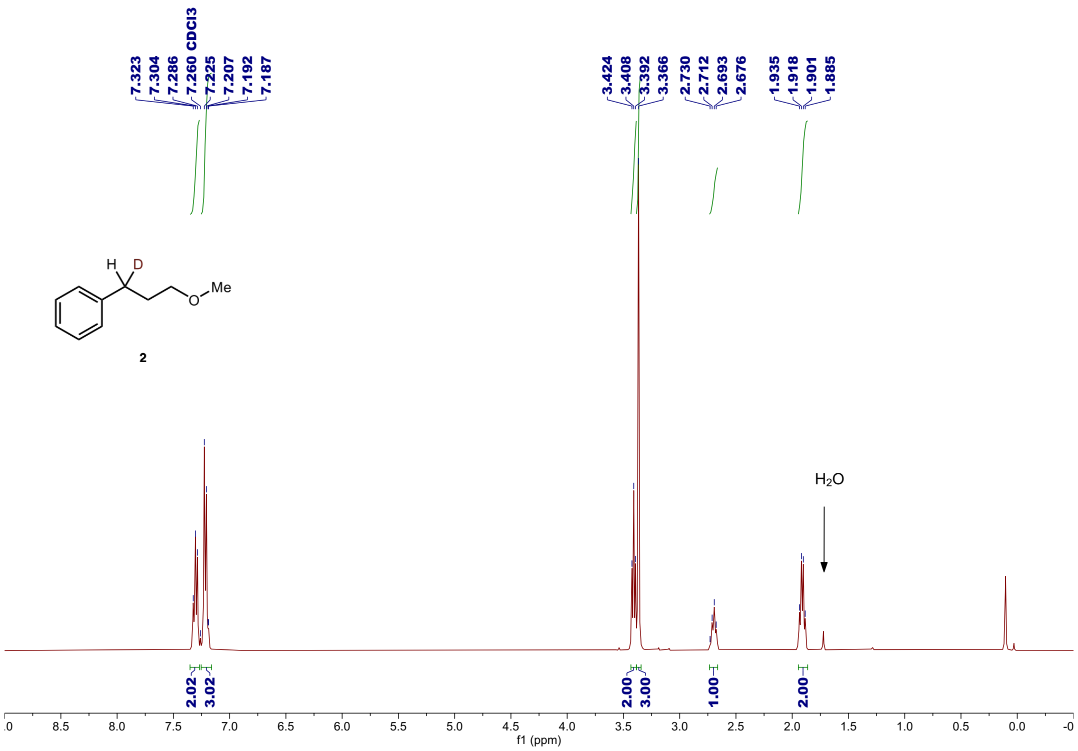

^{13}C NMR 100 MHz, $CDCl_3$

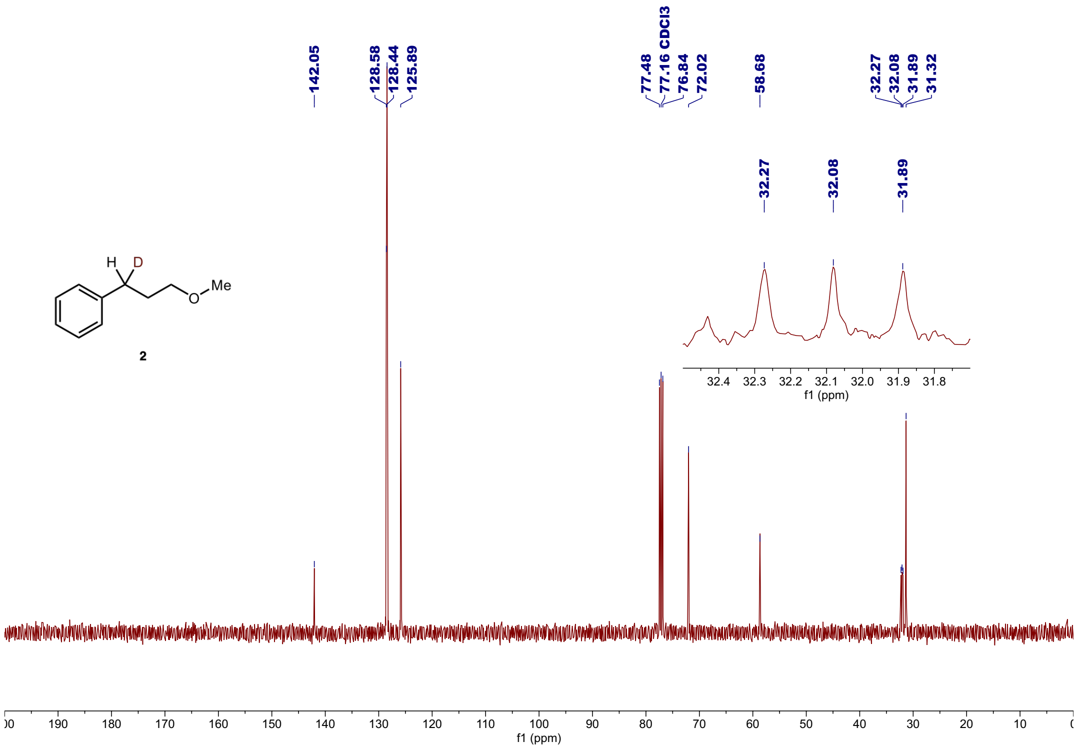

^{1}H NMR 400 MHz, $CDCl_3$

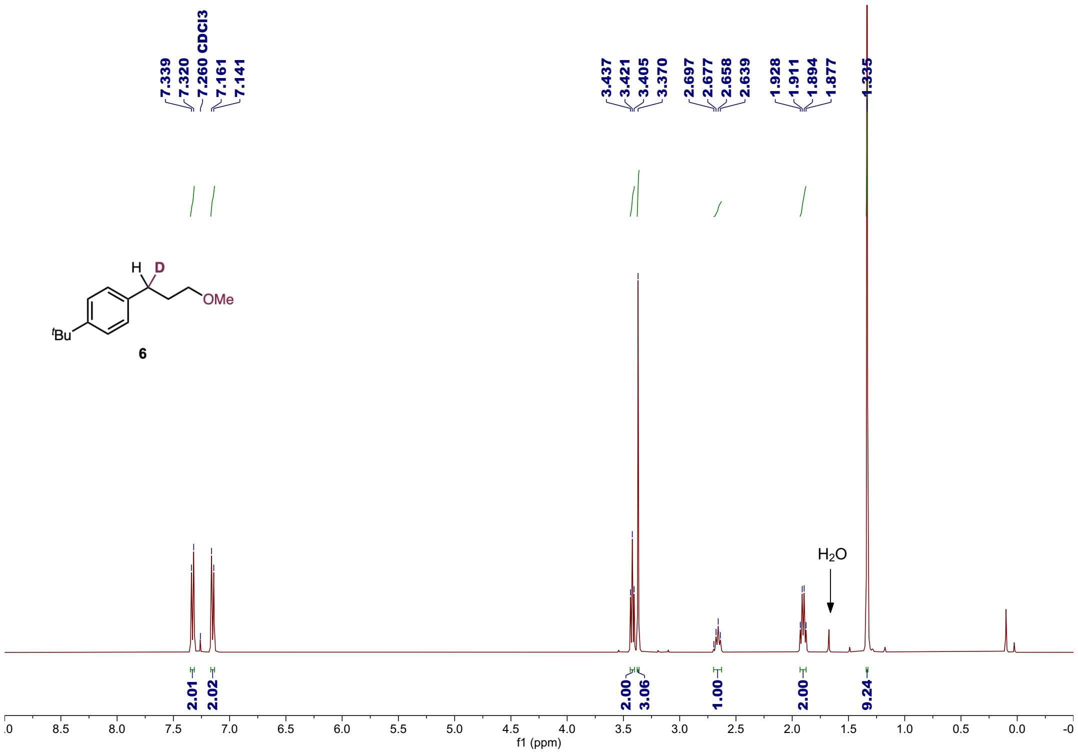

^{13}C NMR 100 MHz, $CDCl_3$

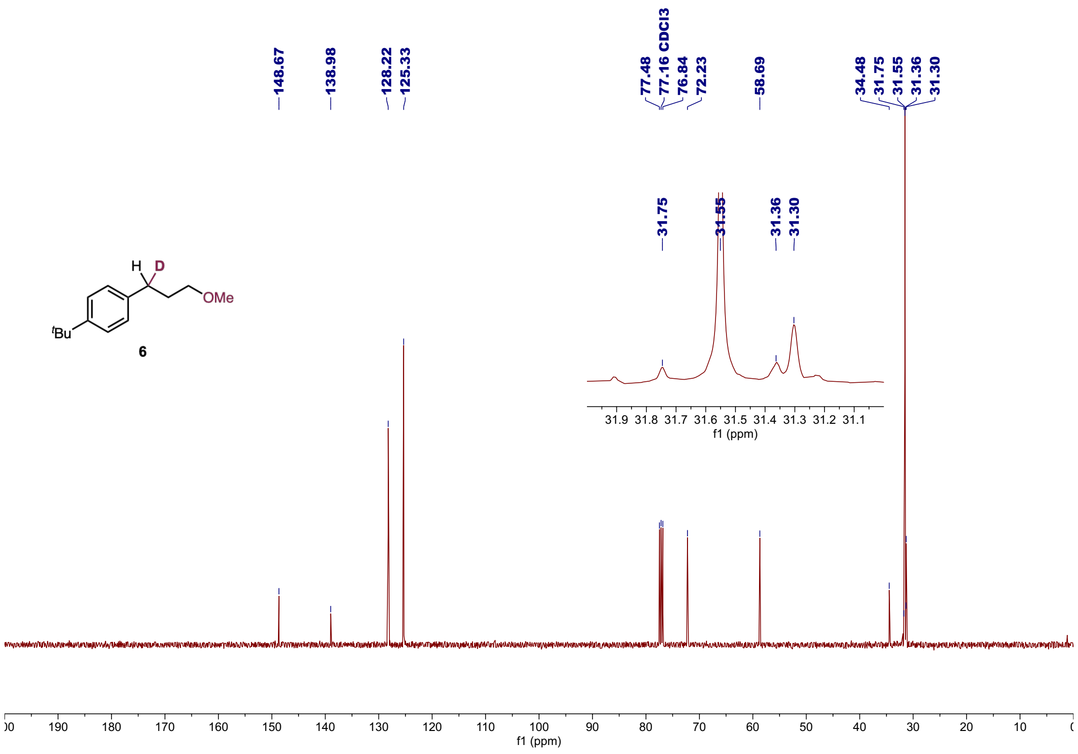

^{1}H NMR 400 MHz, $CDCl_3$

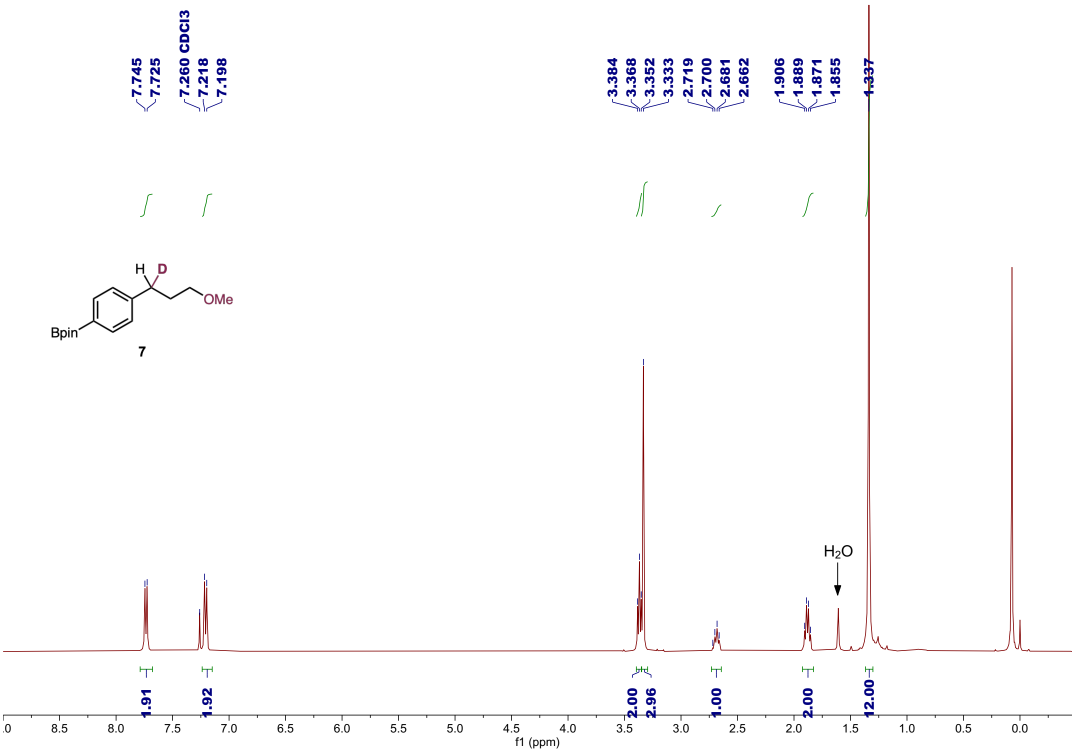

^{13}C NMR 100 MHz, $CDCl_3$

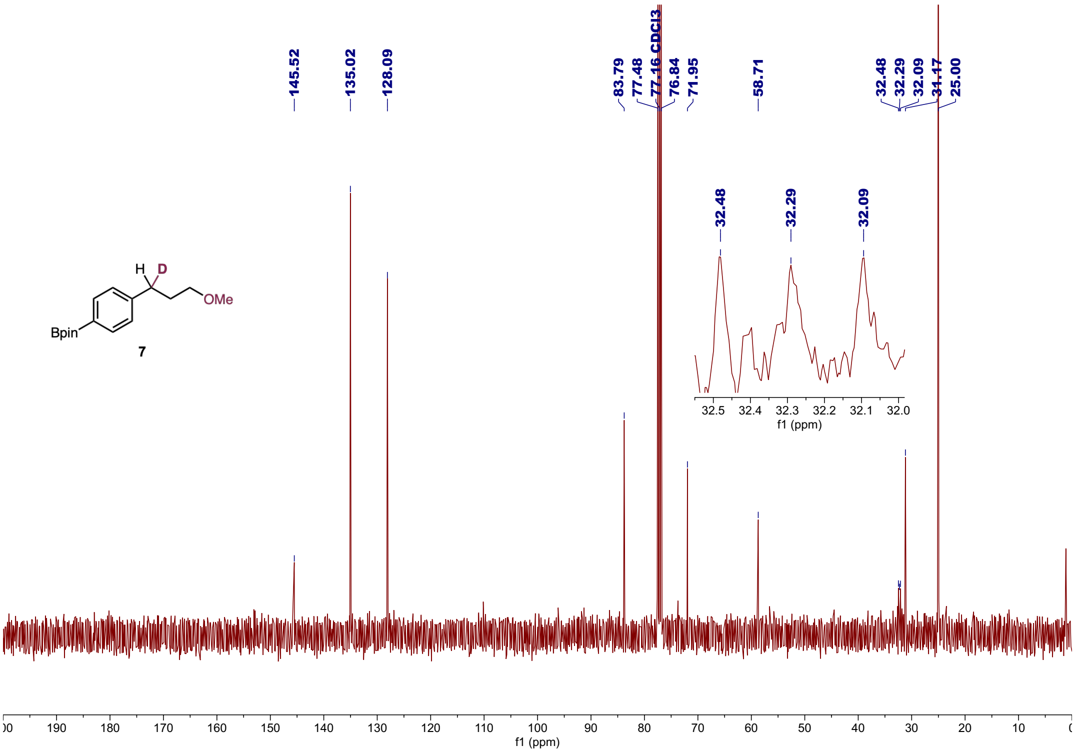

^{1}H NMR 400 MHz, $CDCl_3$

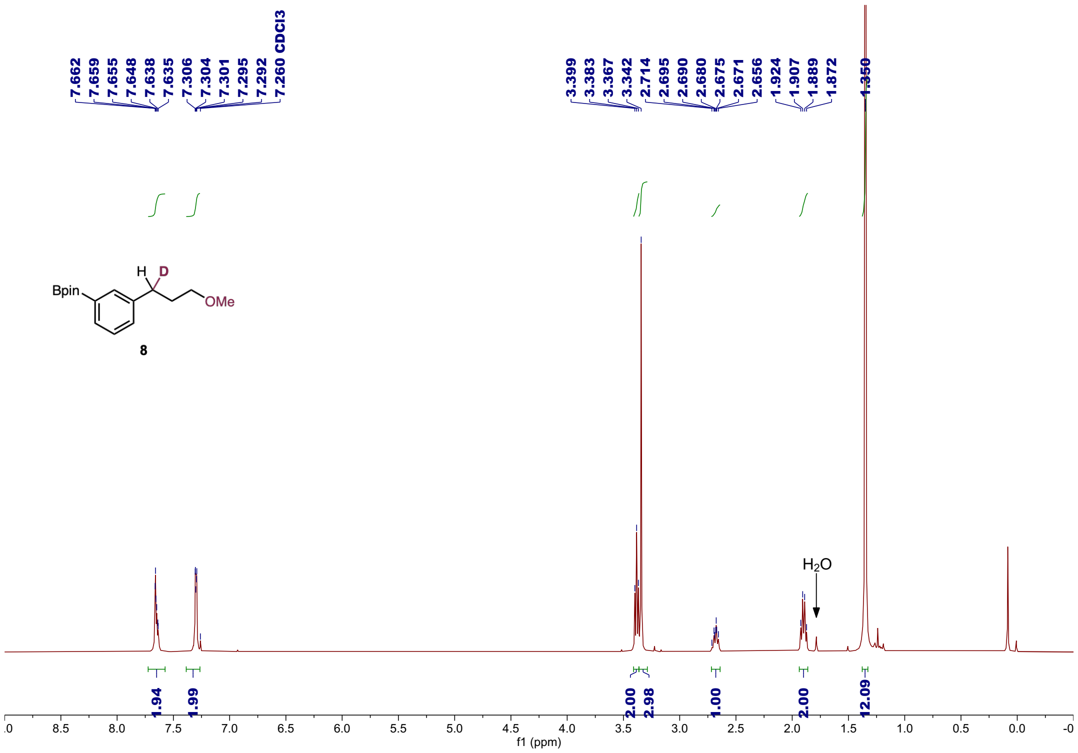

^{13}C NMR 100 MHz, $CDCl_3$

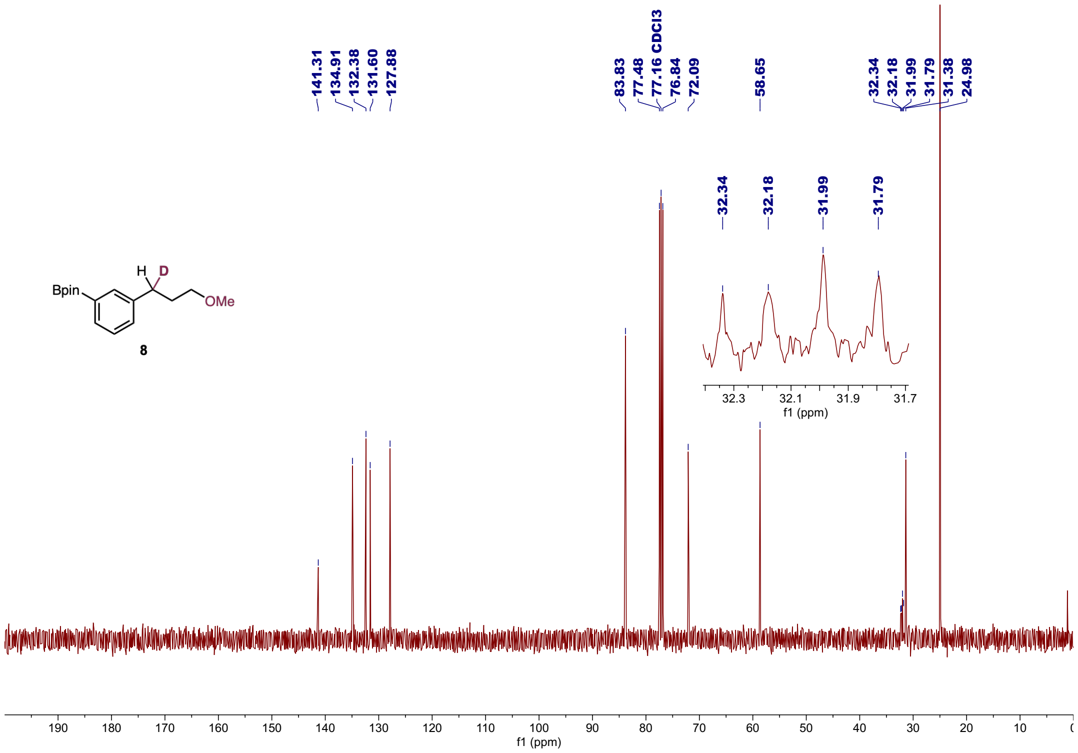

^{1}H NMR 400 MHz, $CDCl_3$

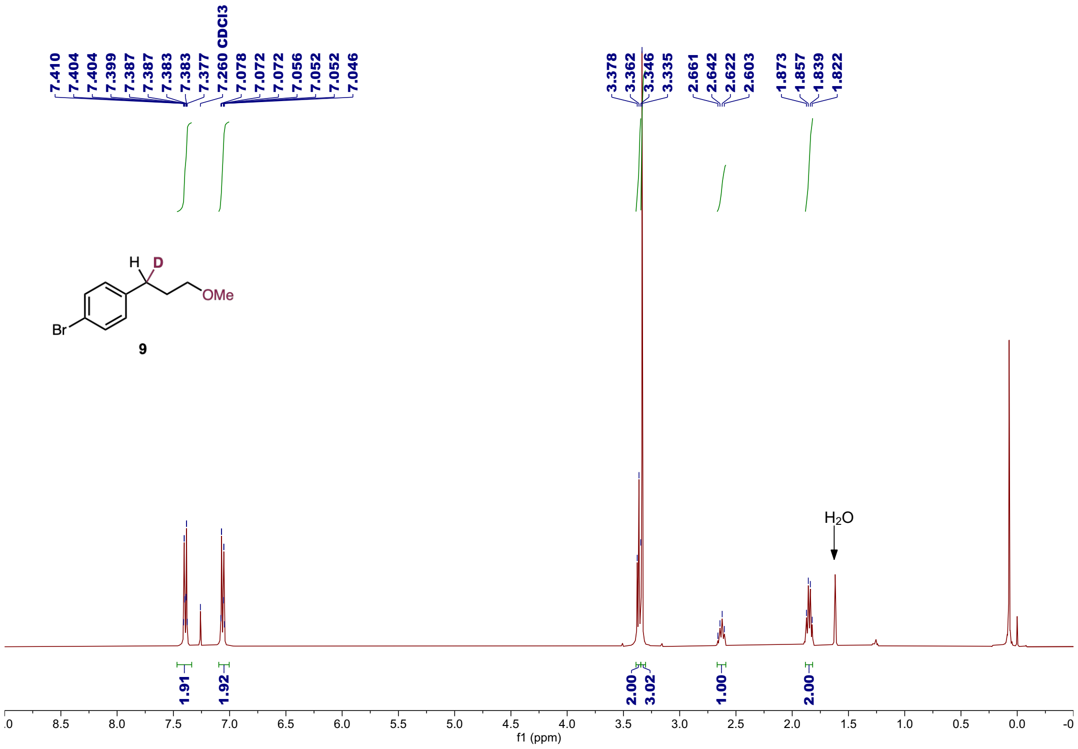

^{13}C NMR 100 MHz, $CDCl_3$

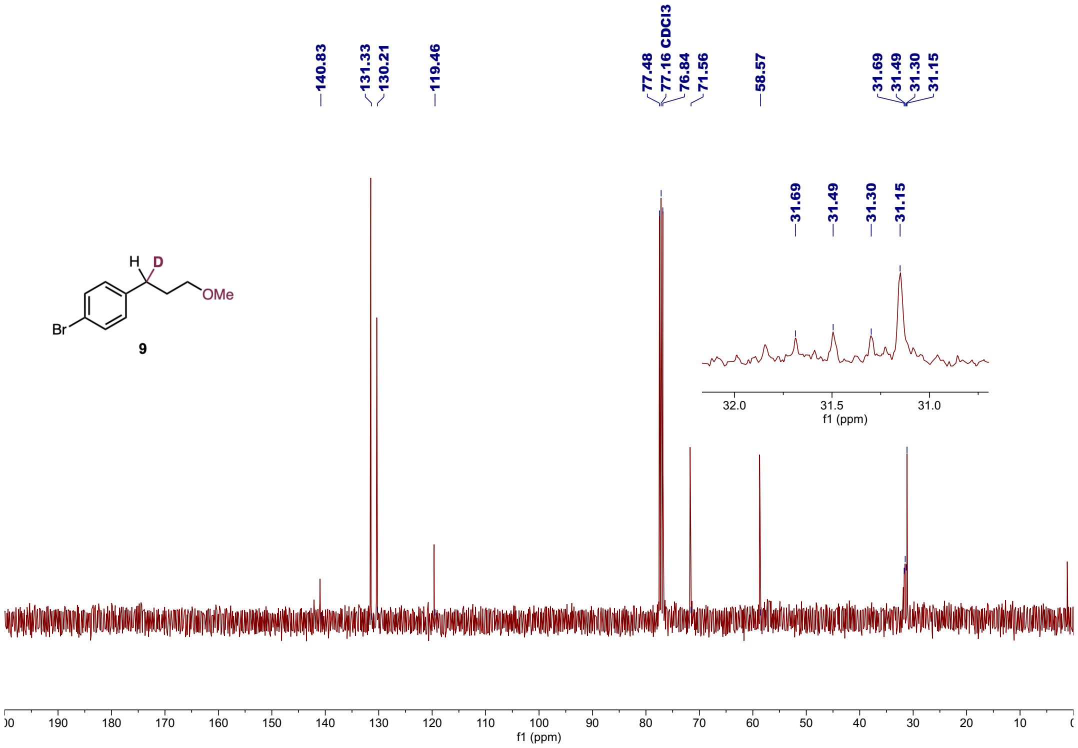

^{1}H NMR 400 MHz, $CDCl_3$

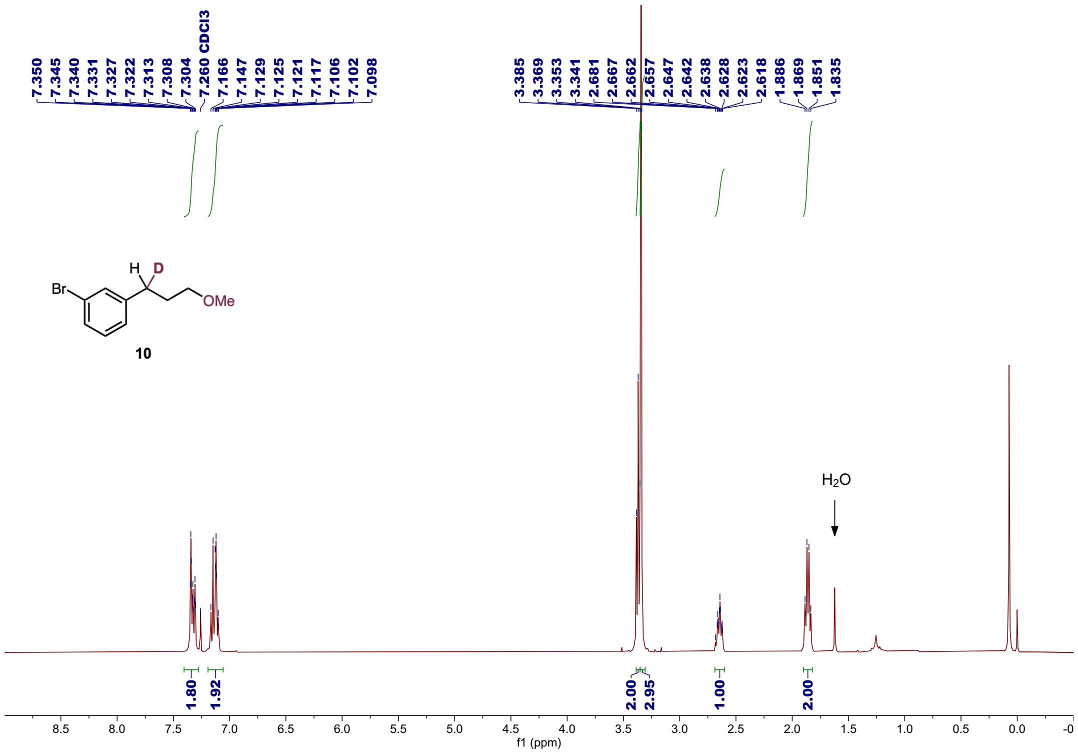

^{13}C NMR 100 MHz, $CDCl_3$

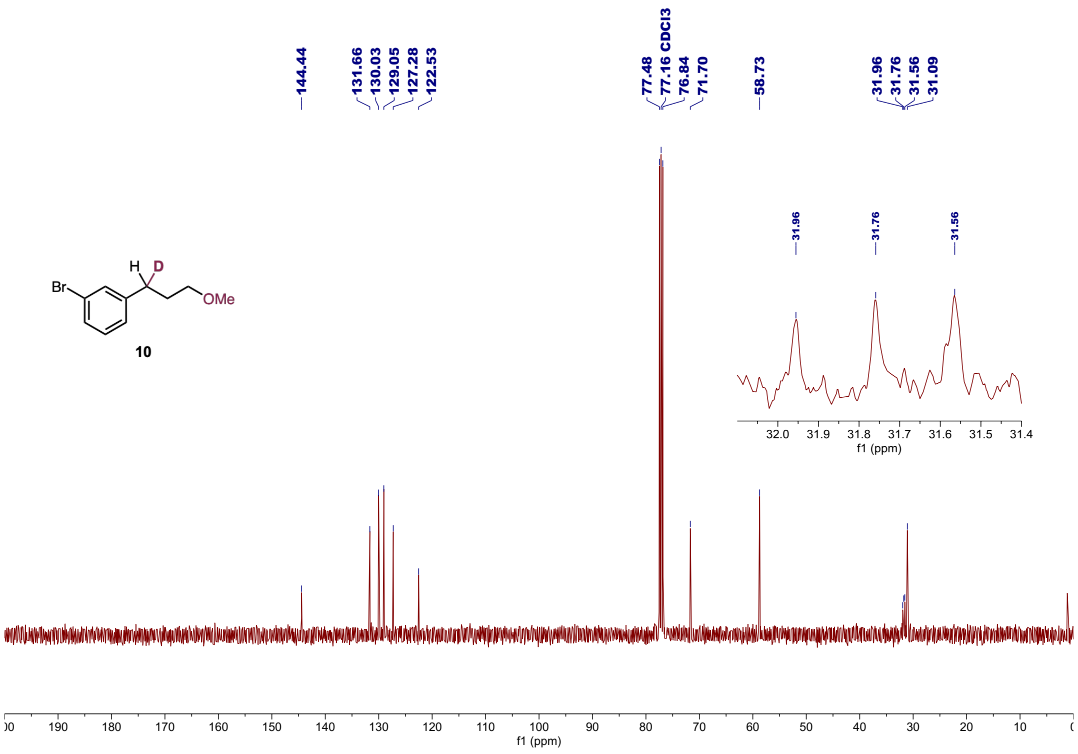

^{1}H NMR 400 MHz, $CDCl_3$

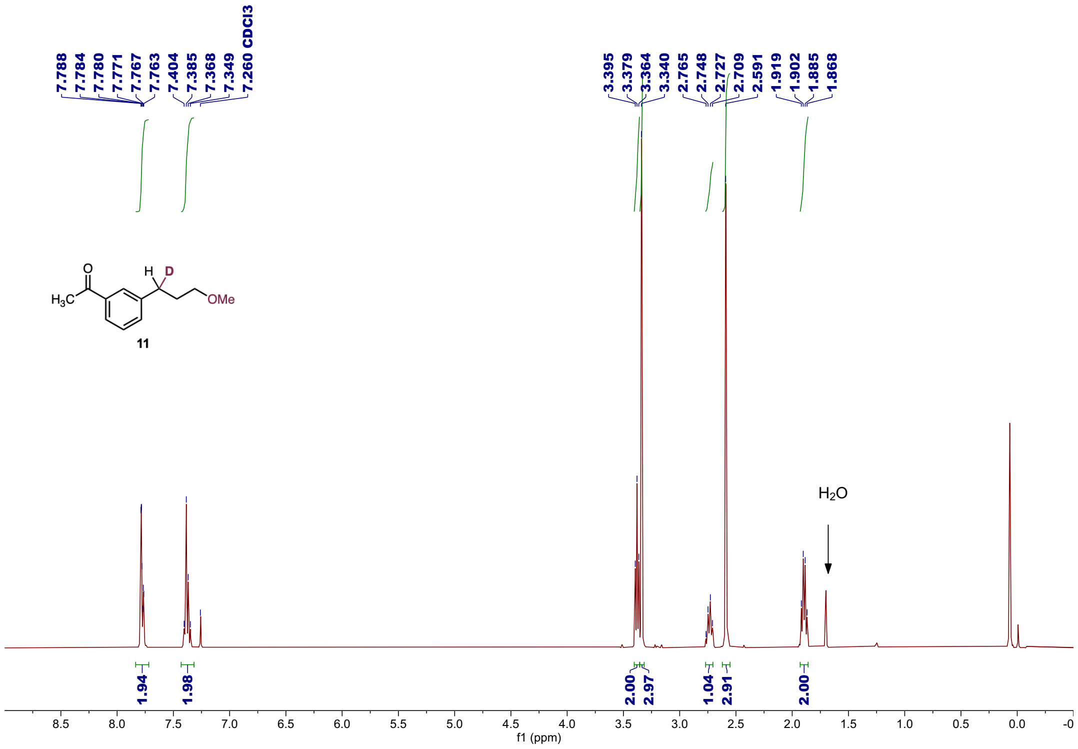

^{13}C NMR 100 MHz, $CDCl_3$

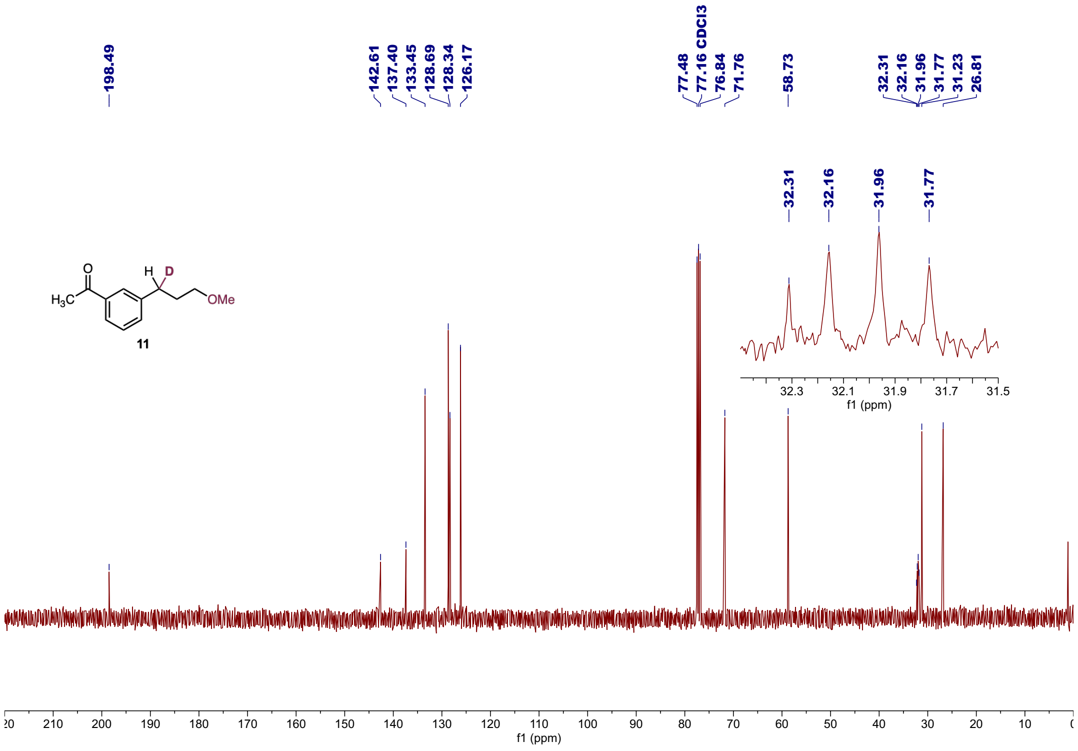

^{1}H NMR 400 MHz, $CDCl_3$

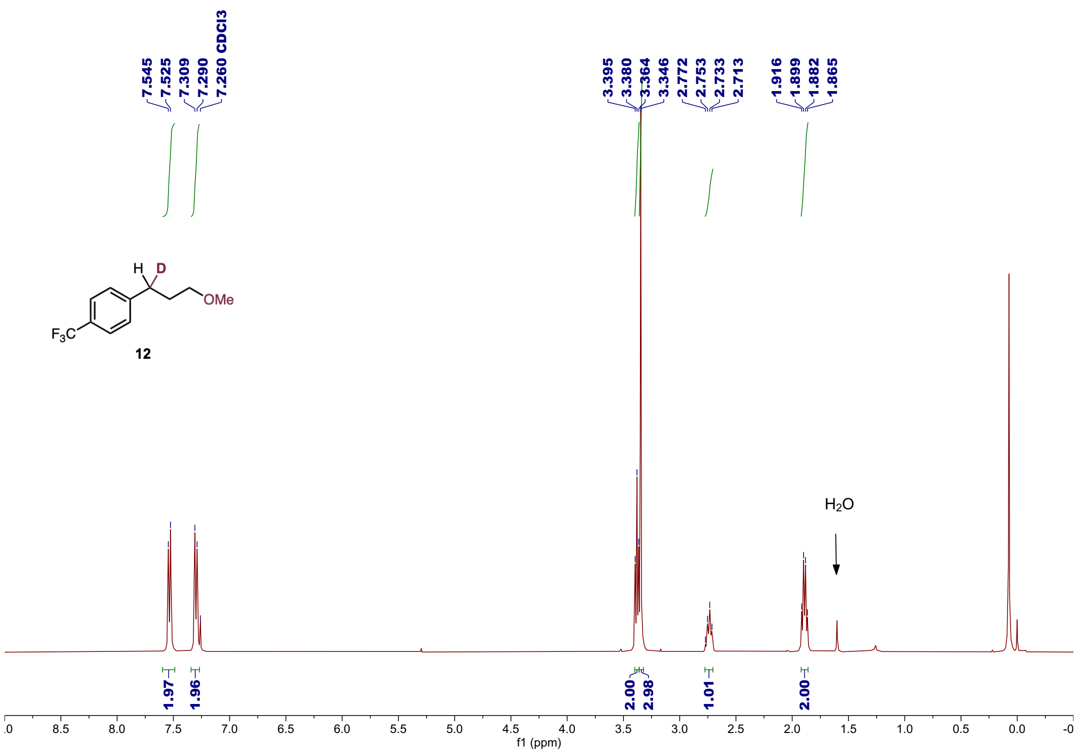

^{13}C NMR 100 MHz, $CDCl_3$

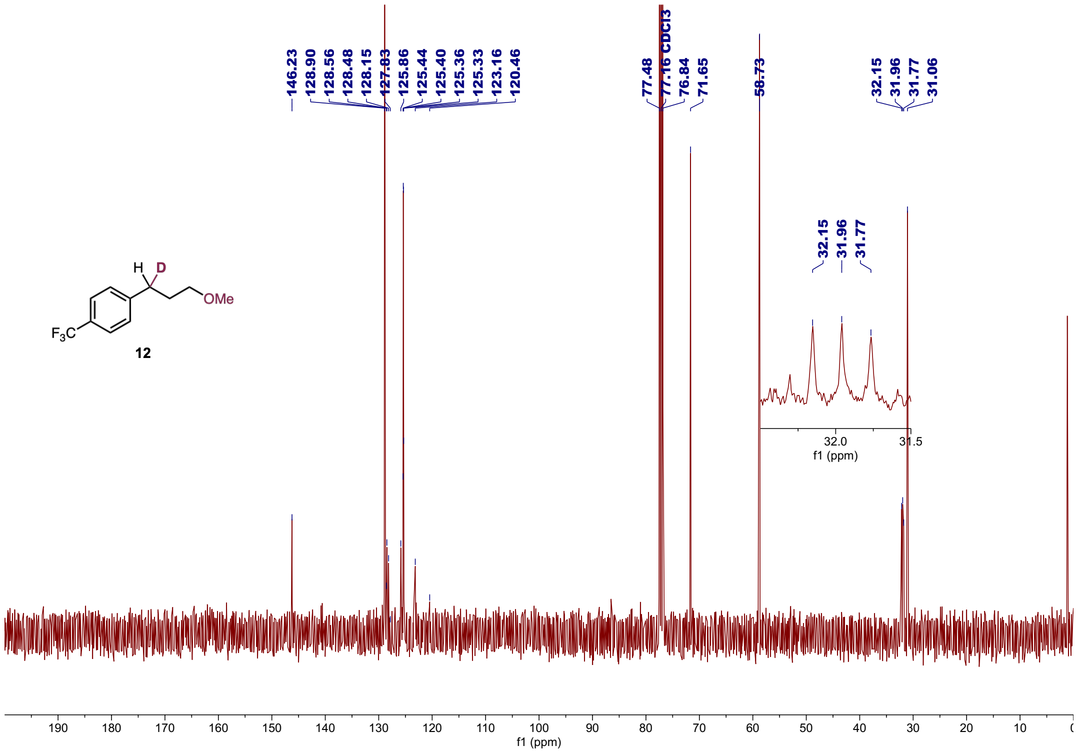

^{19}F NMR 376 MHz, $CDCl_3$

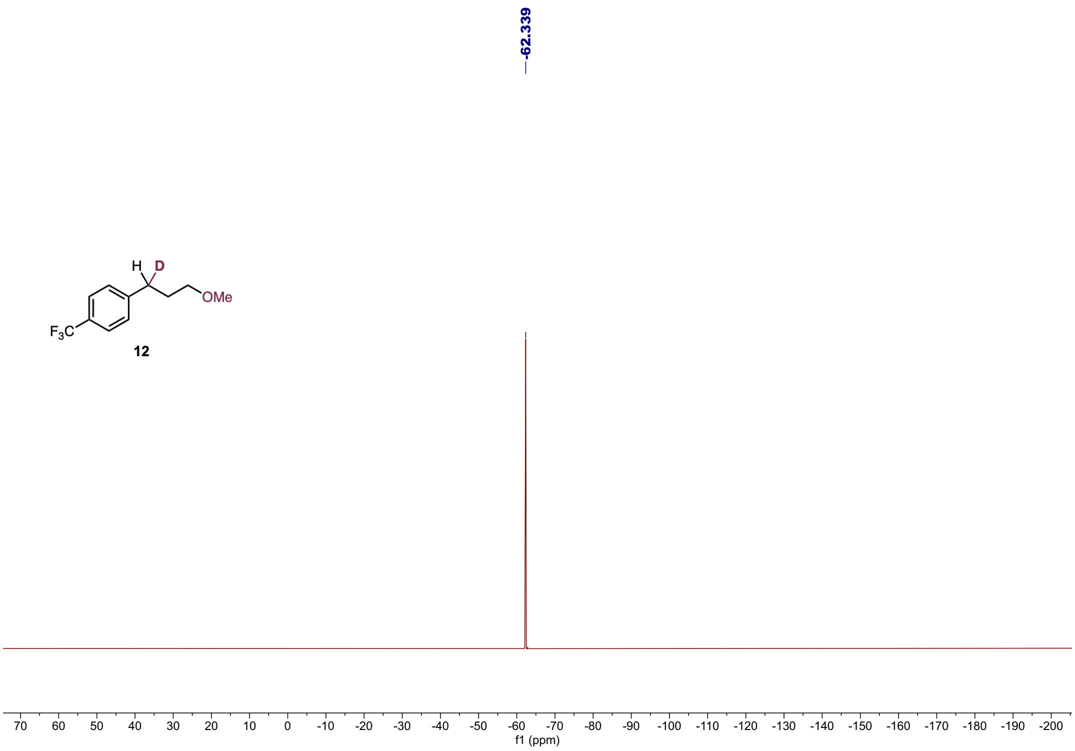

^{1}H NMR 400 MHz, $CDCl_3$

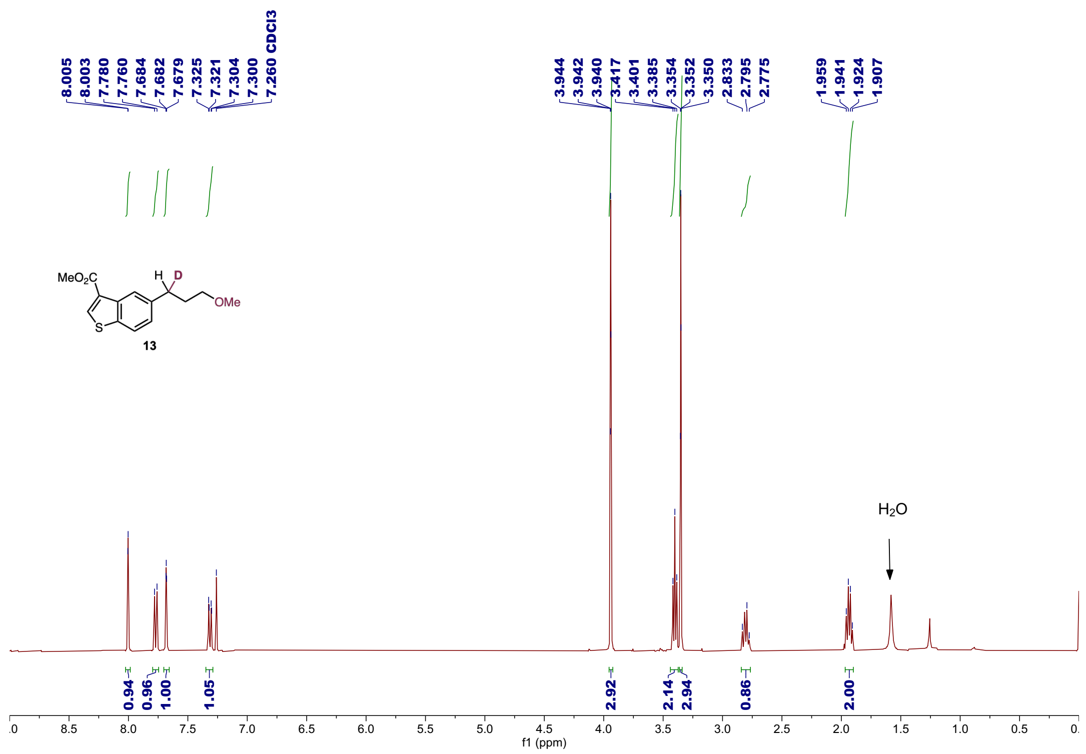

^{13}C NMR 100 MHz, $CDCl_3$

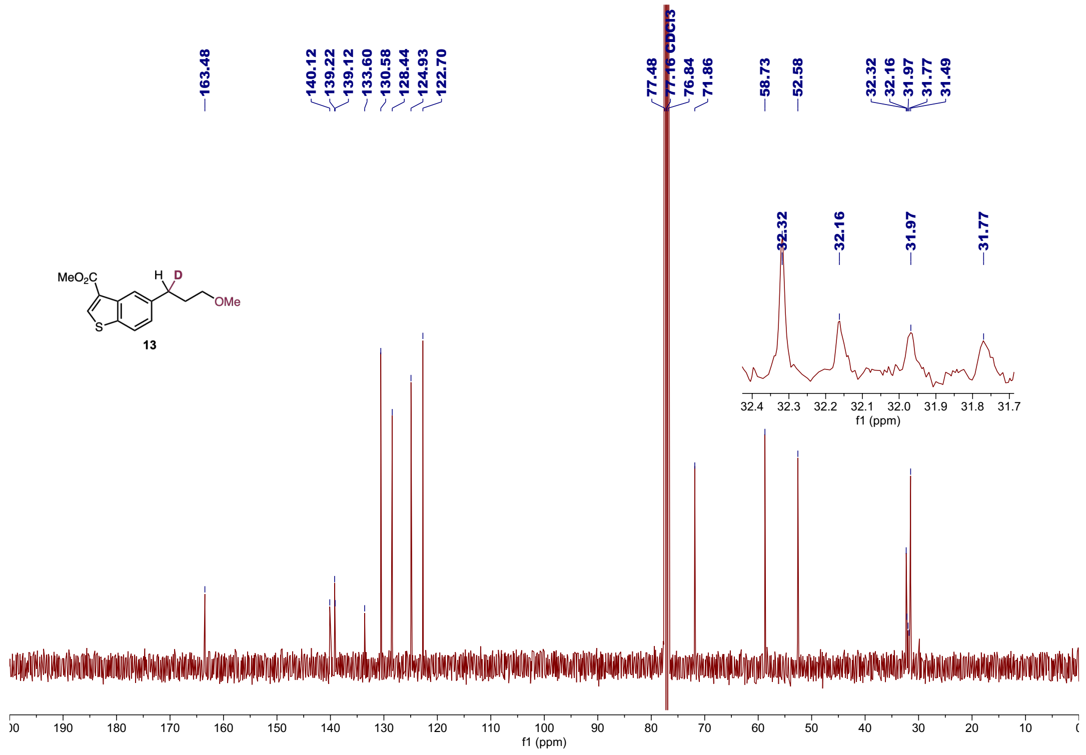

^{1}H NMR 400 MHz, $CDCl_3$

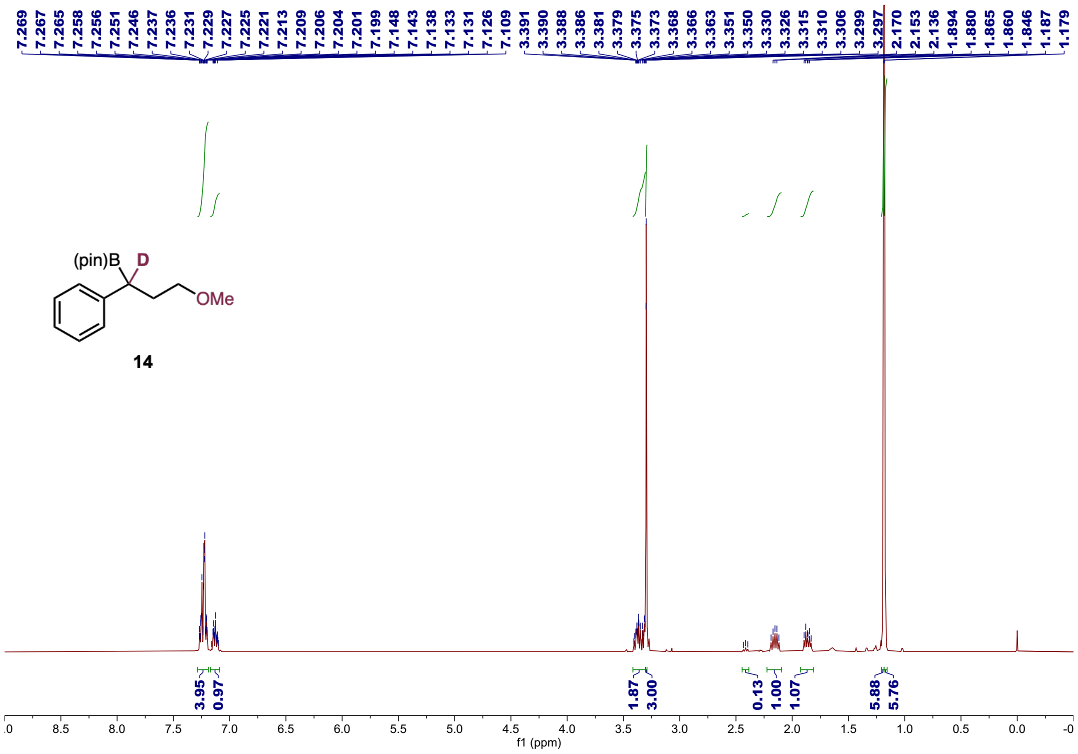

^{13}C NMR 100 MHz, $CDCl_3$

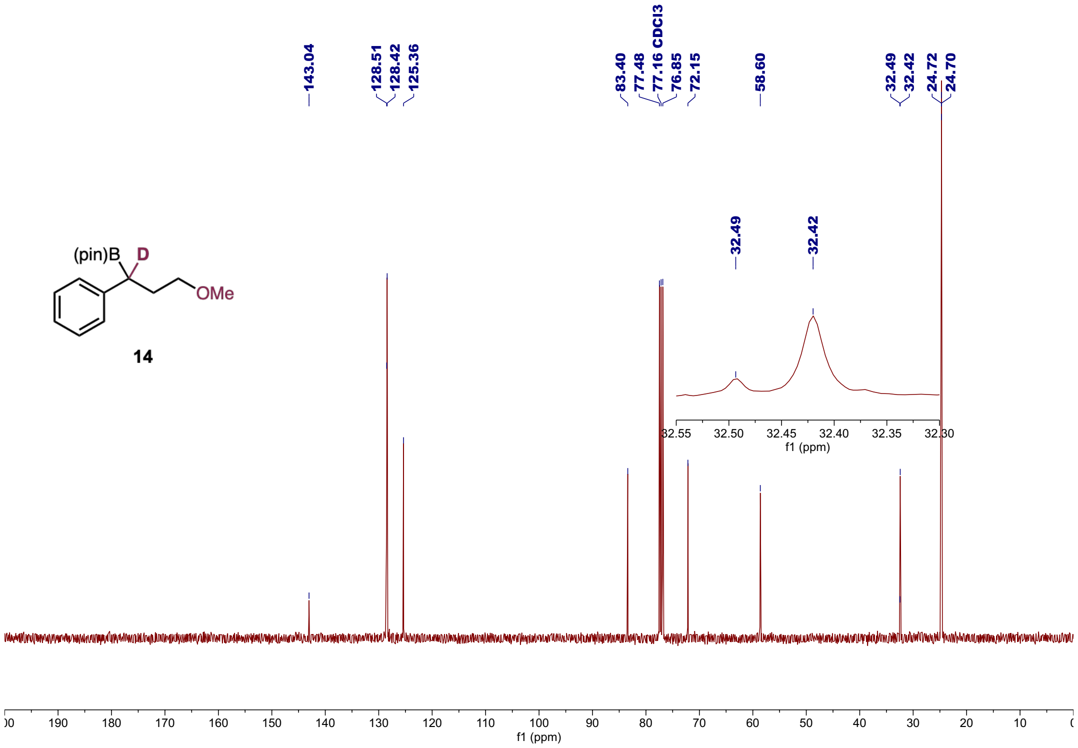

^{1}H NMR 400 MHz, $CDCl_3$

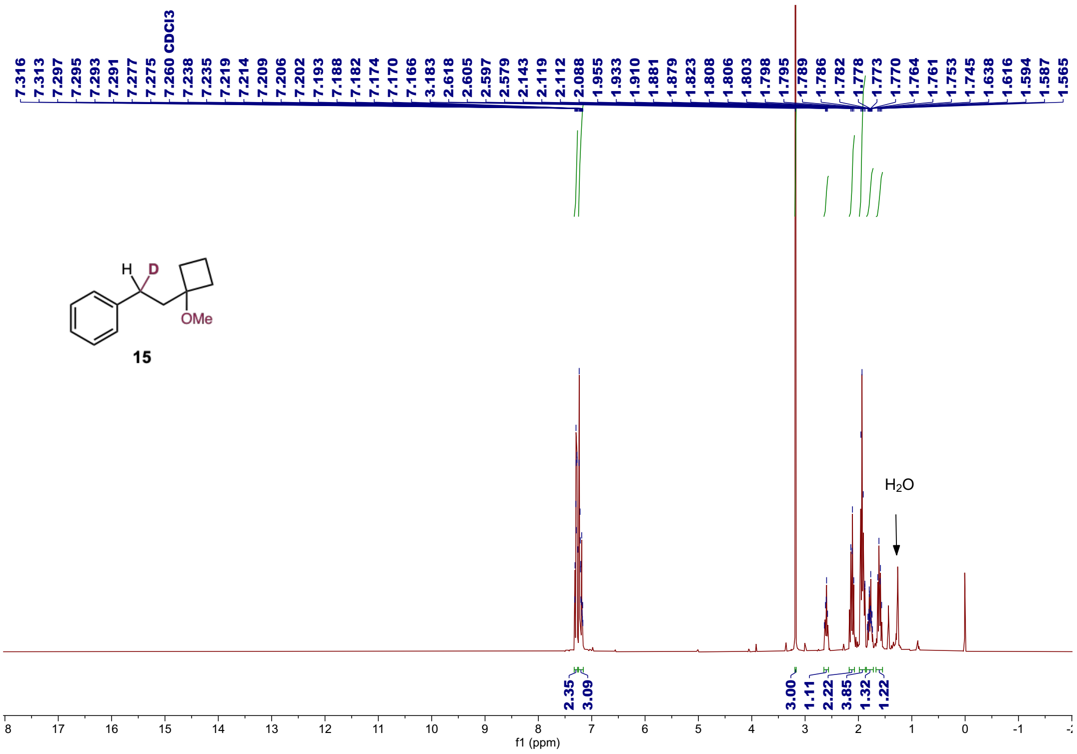

^{13}C NMR 100 MHz, $CDCl_3$

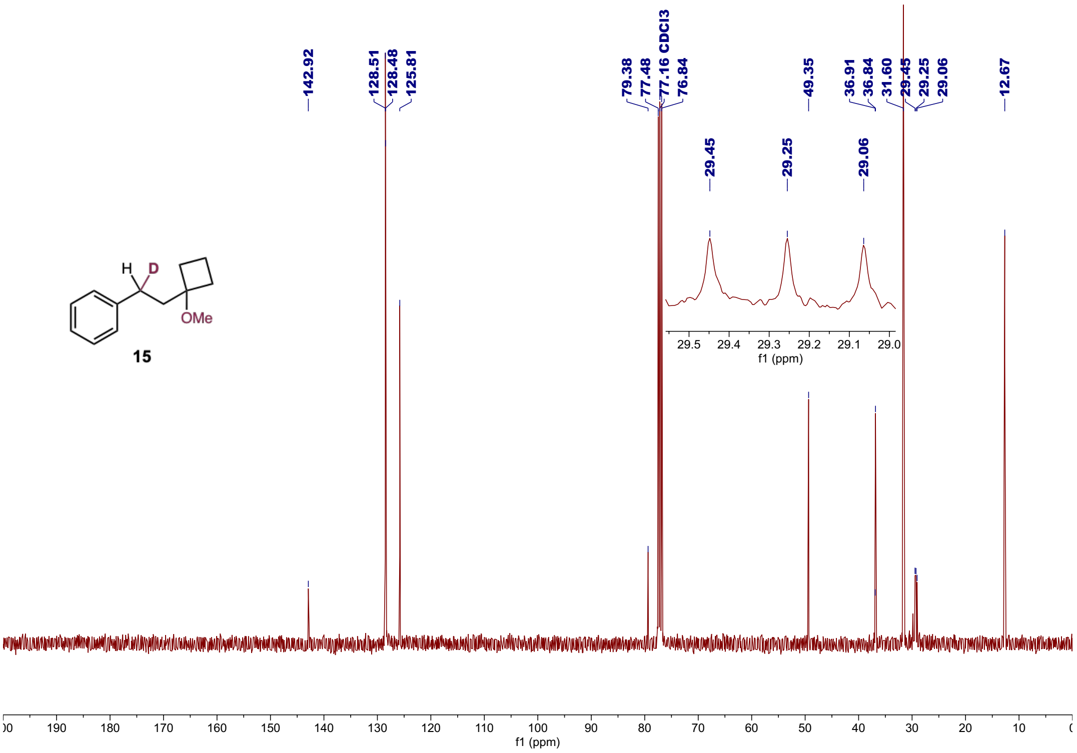

^{1}H NMR 400 MHz, $CDCl_3$

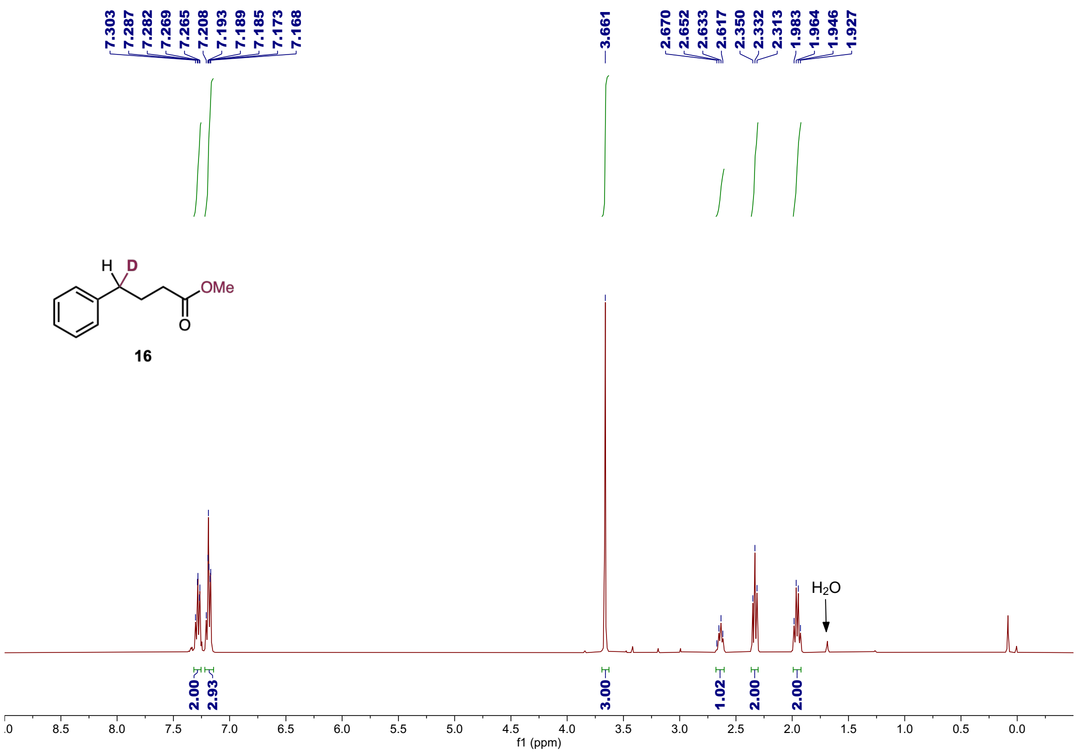

^{13}C NMR 100 MHz, $CDCl_3$

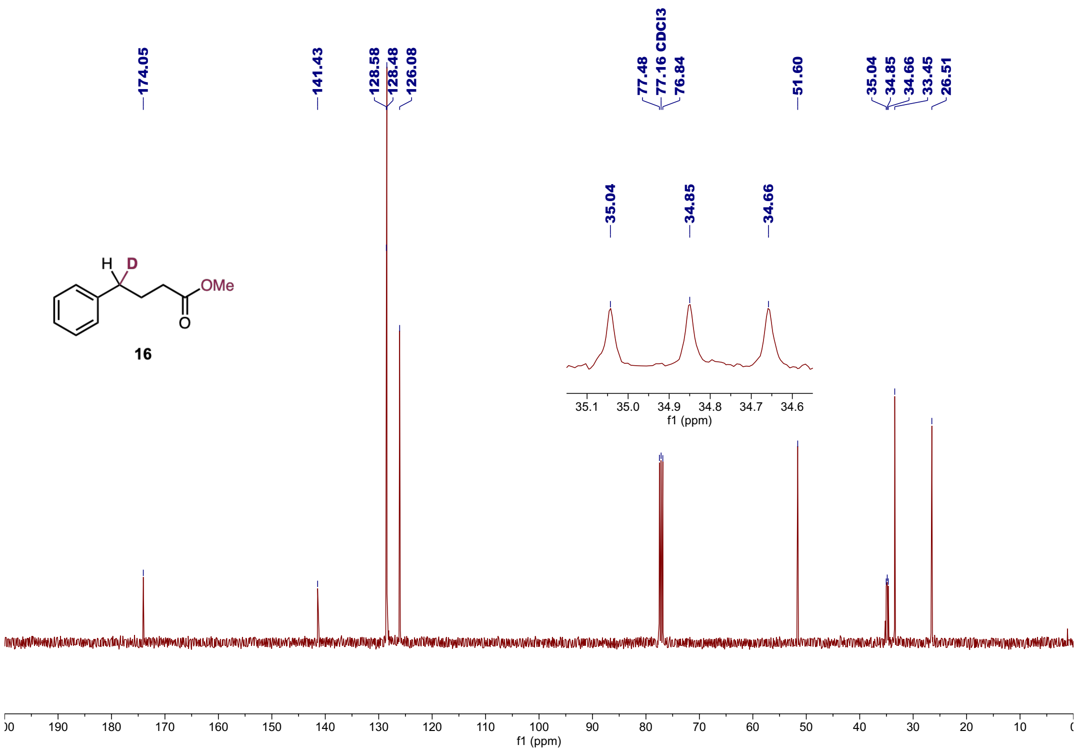

^{1}H NMR 400 MHz, $CDCl_3$

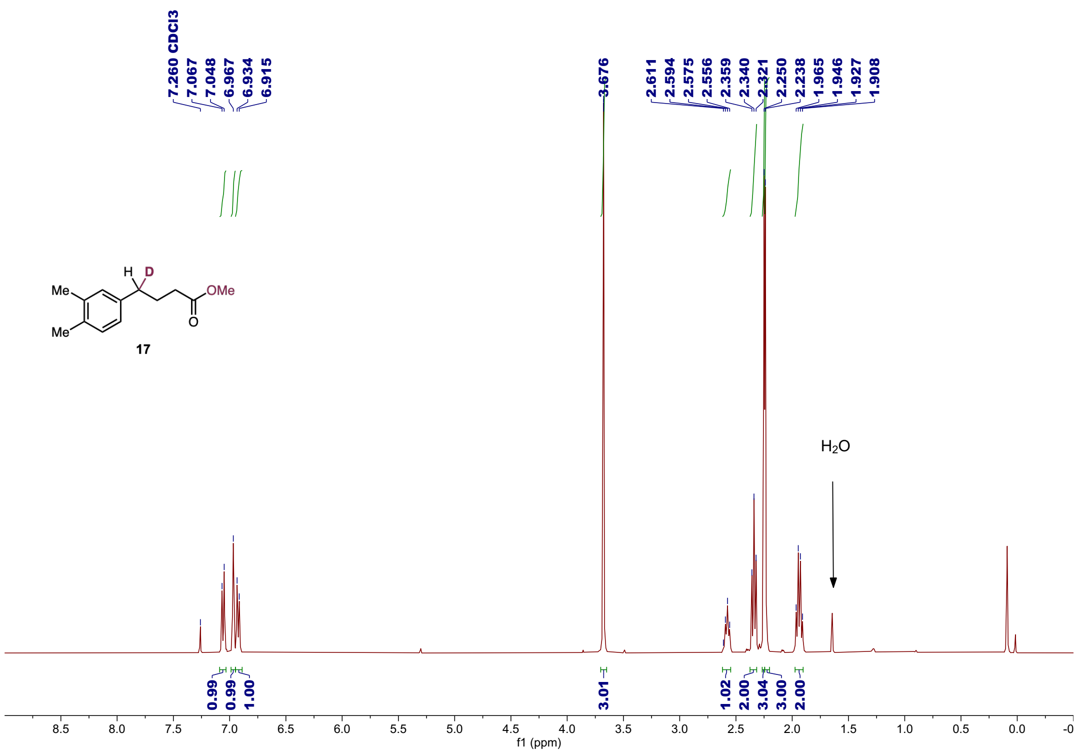

^{13}C NMR 100 MHz, $CDCl_3$

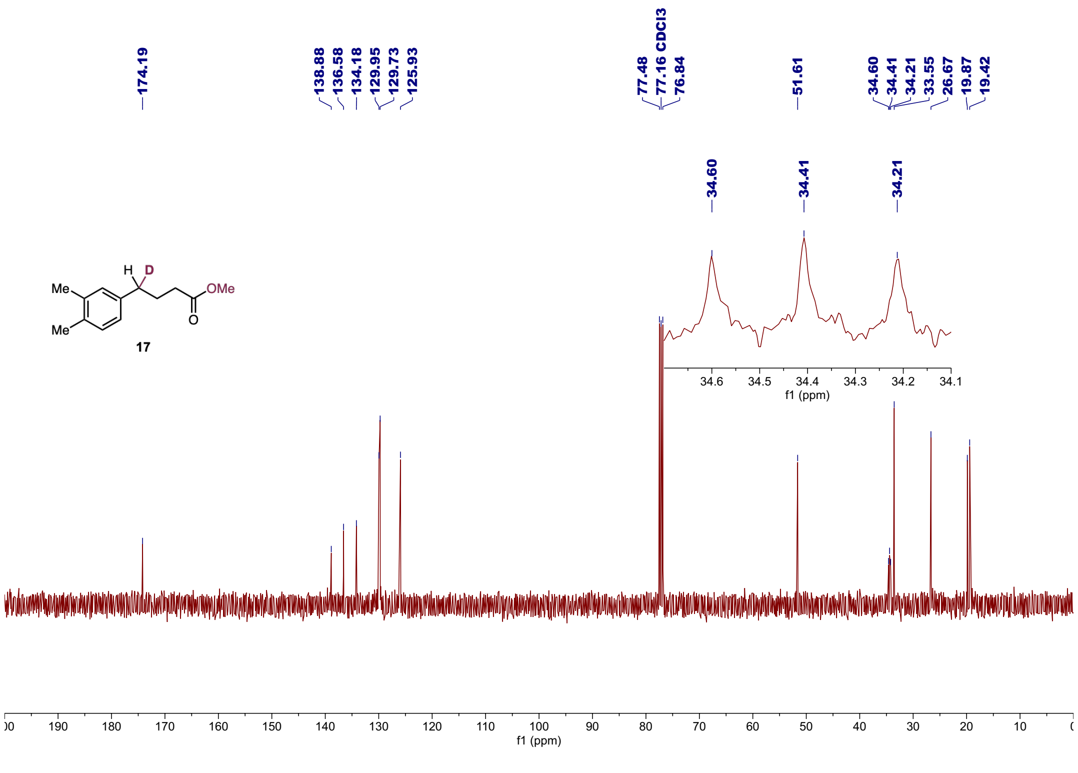

^{1}H NMR 400 MHz, CDCl$_3$

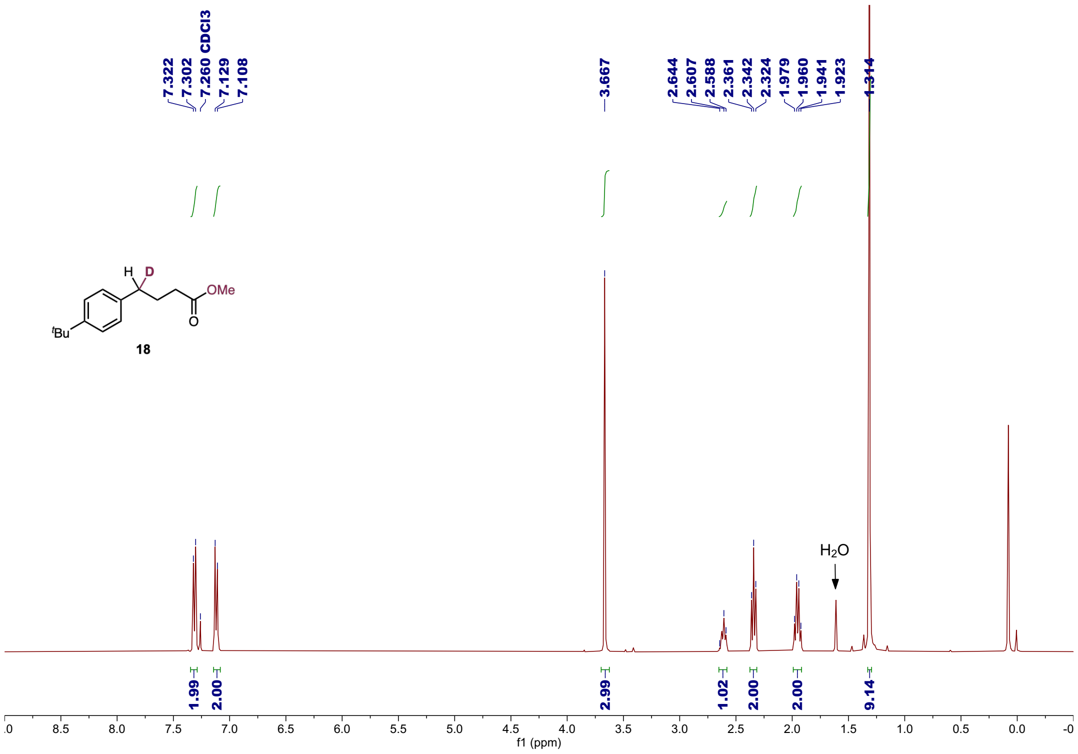

^{13}C NMR 100 MHz, CDCl$_3$

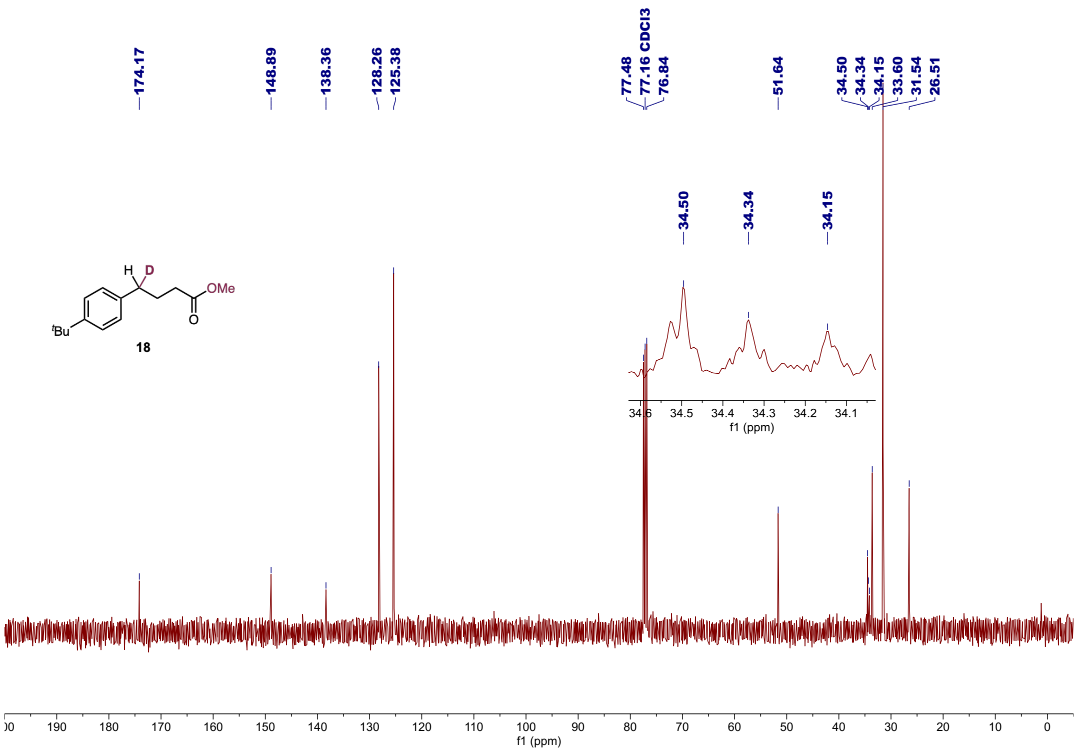

^{1}H NMR 400 MHz, CDCl$_3$

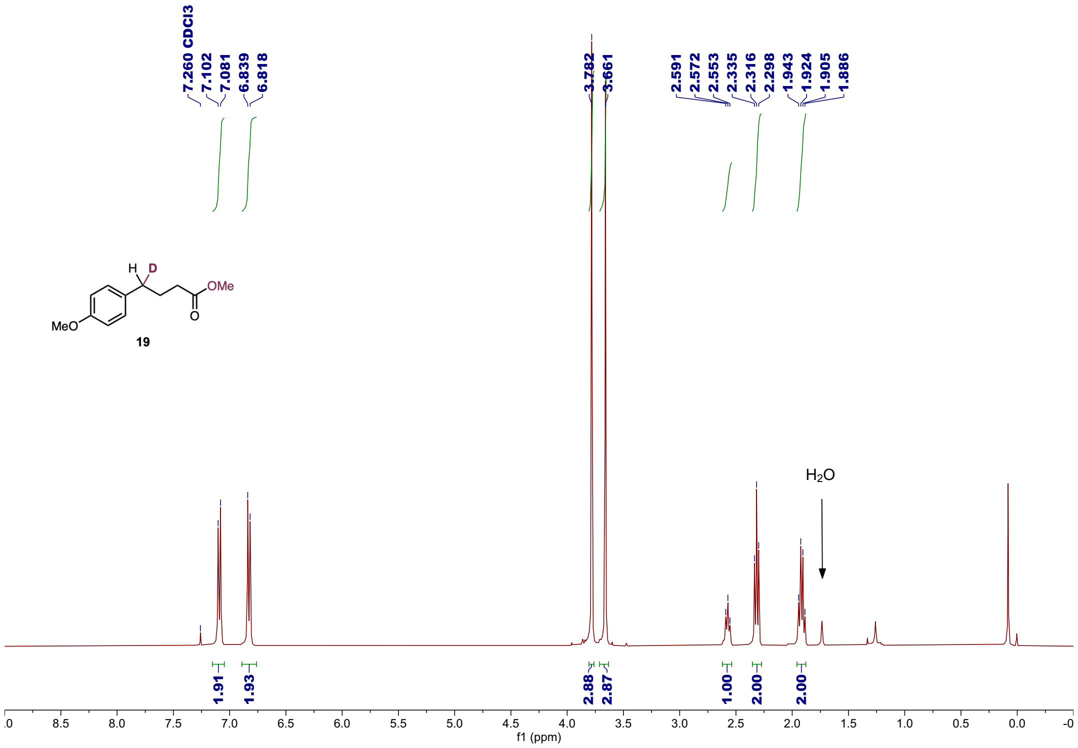

^{13}C NMR 100 MHz, CDCl$_3$

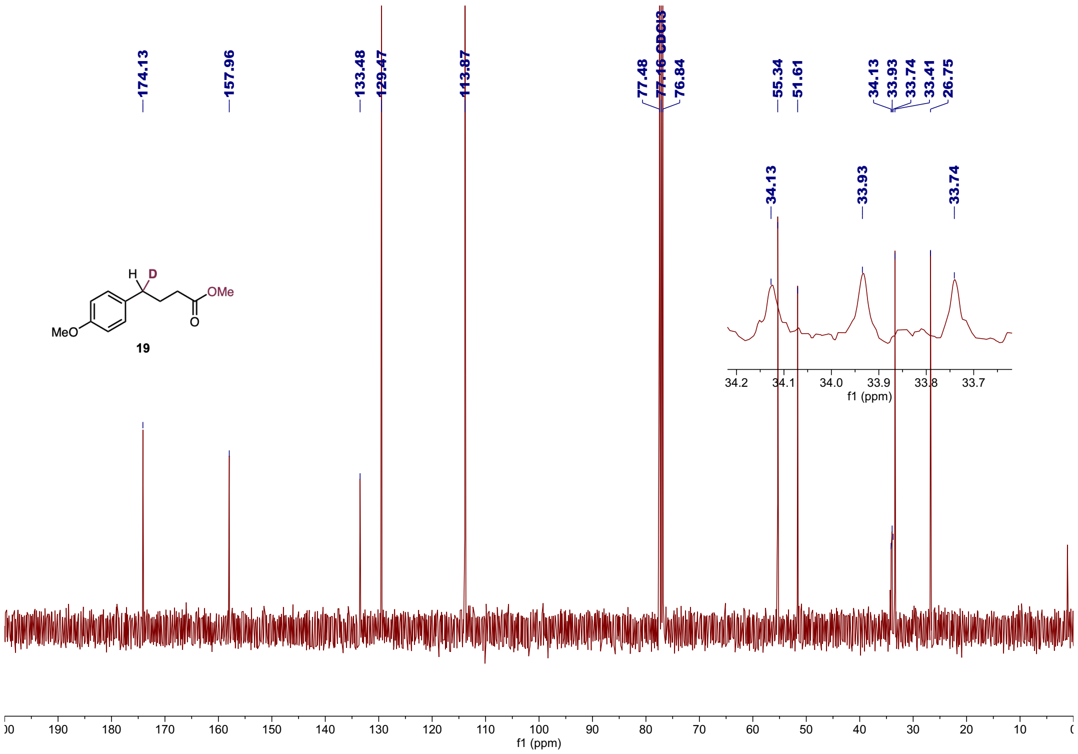

^1H NMR 400 MHz, $CDCl_3$

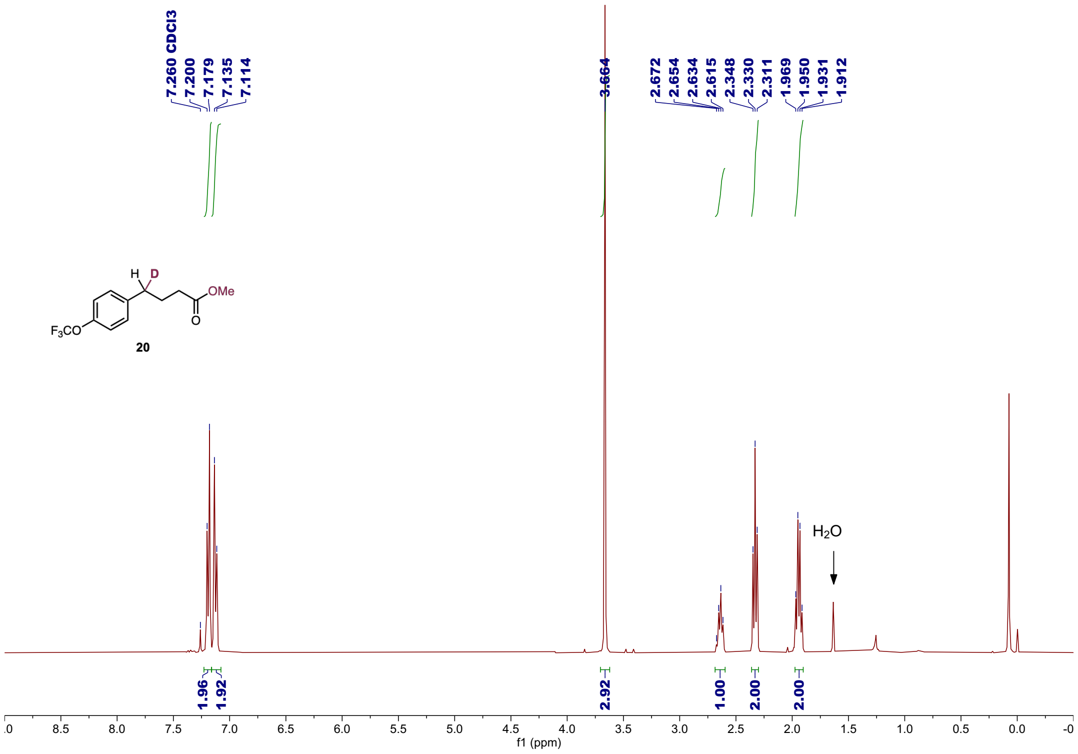

^{13}C NMR 100 MHz, $CDCl_3$

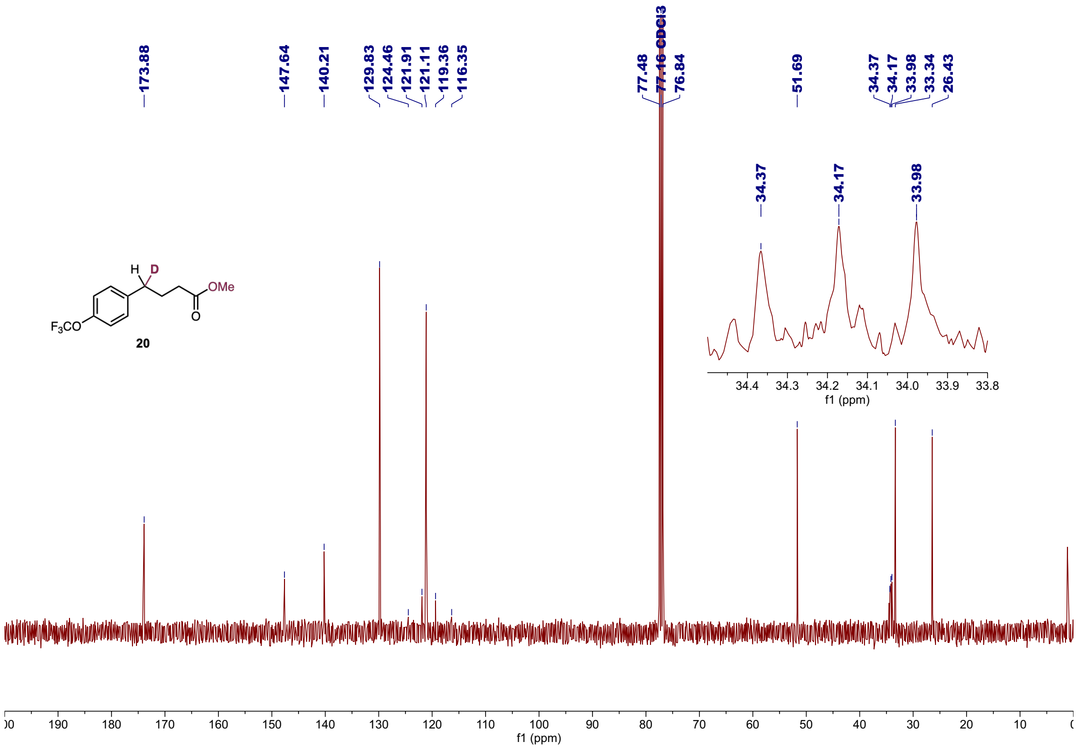

^{19}F NMR 376 MHz, $CDCl_3$

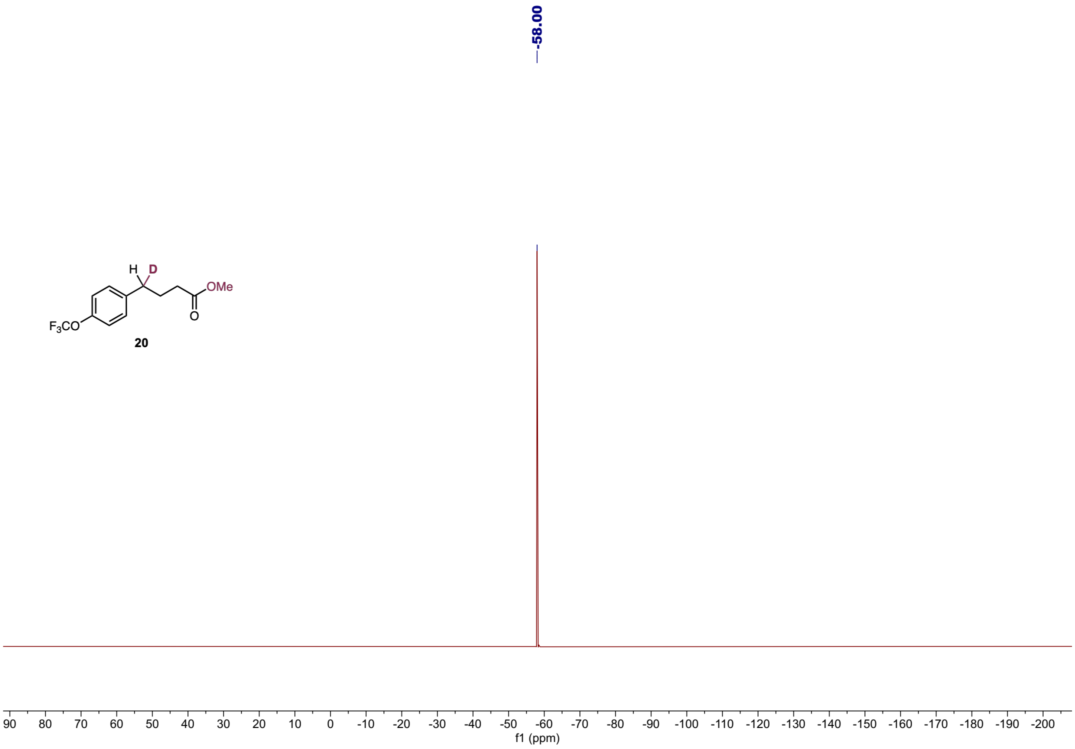

^{1}H NMR 400 MHz, $CDCl_3$

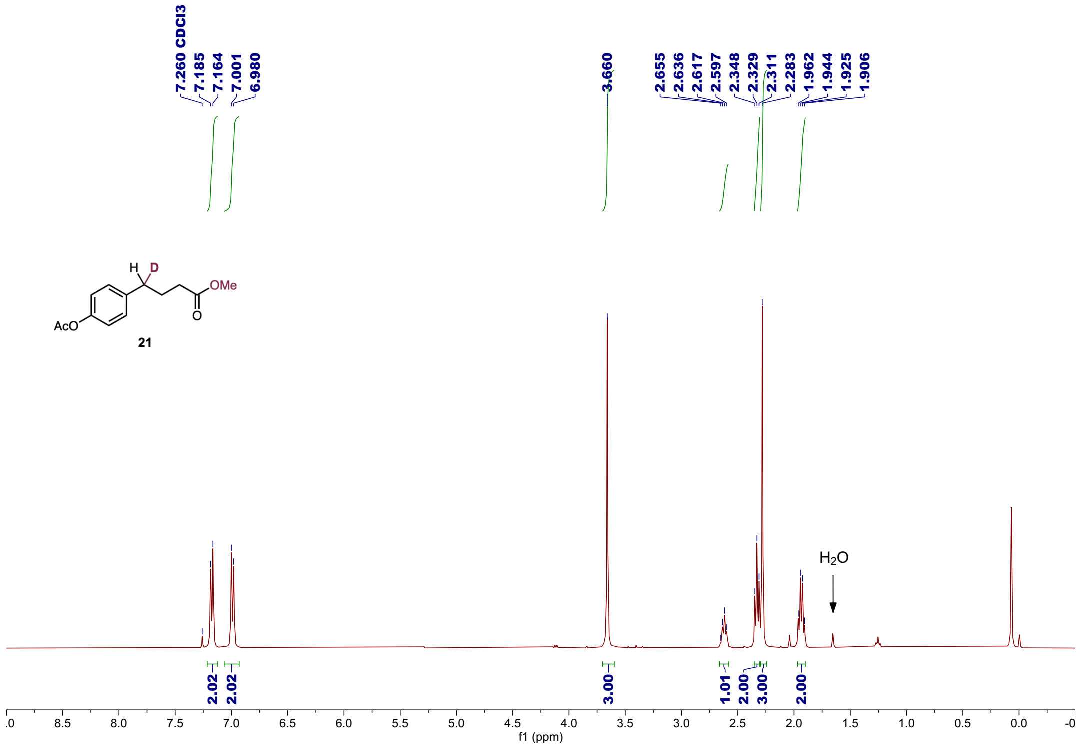

^{13}C NMR 100 MHz, $CDCl_3$

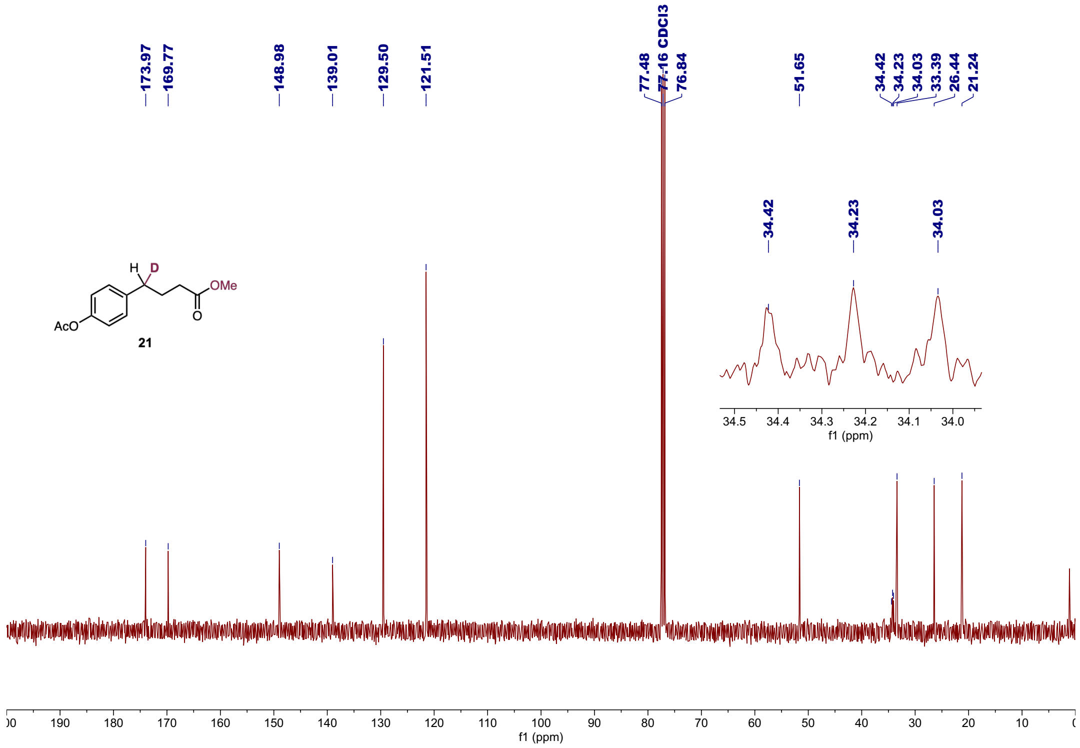

^{1}H NMR 400 MHz, $CDCl_3$

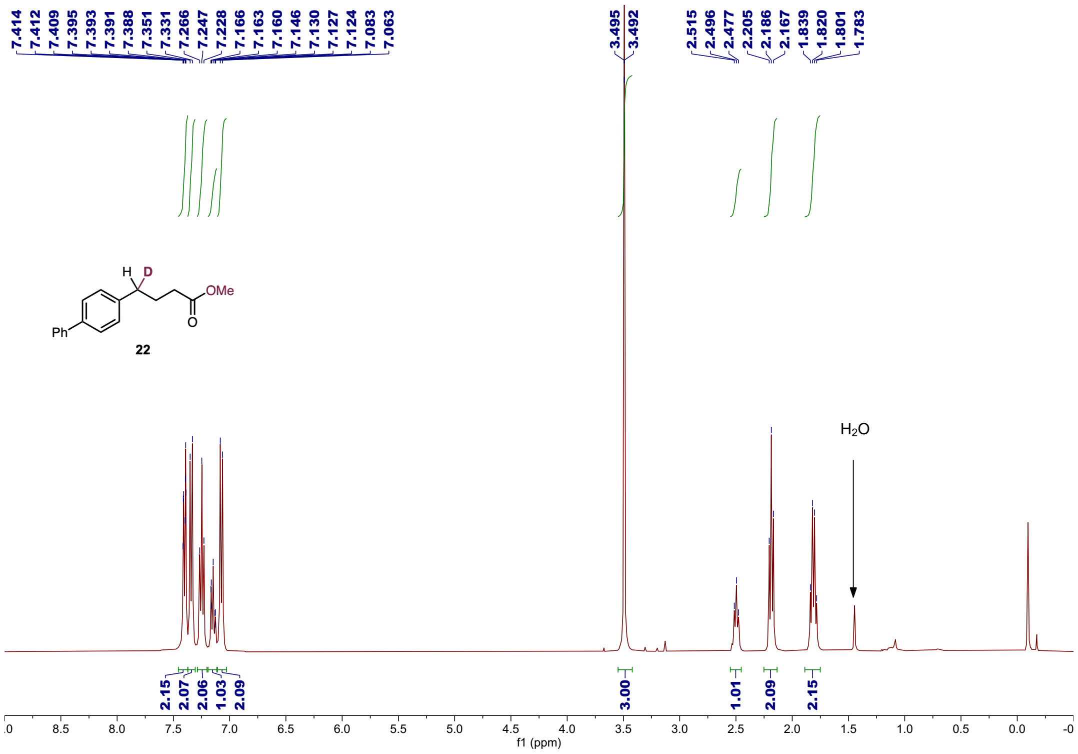

^{13}C NMR 100 MHz, $CDCl_3$

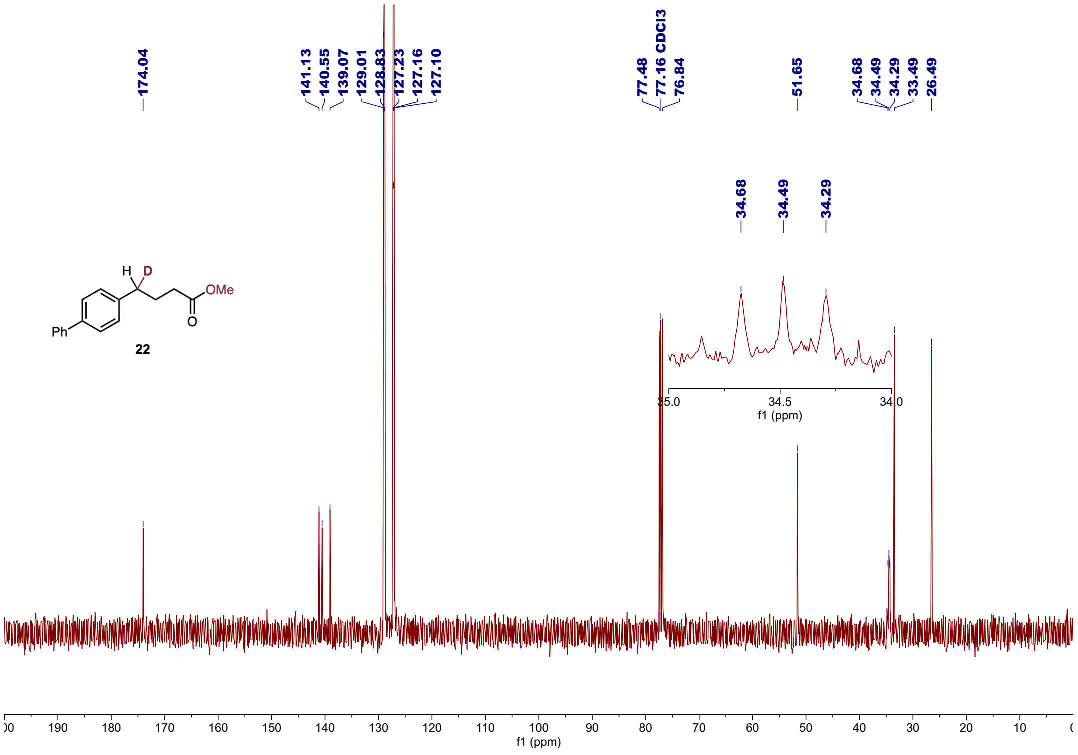

^{1}H NMR 400 MHz, $CDCl_3$

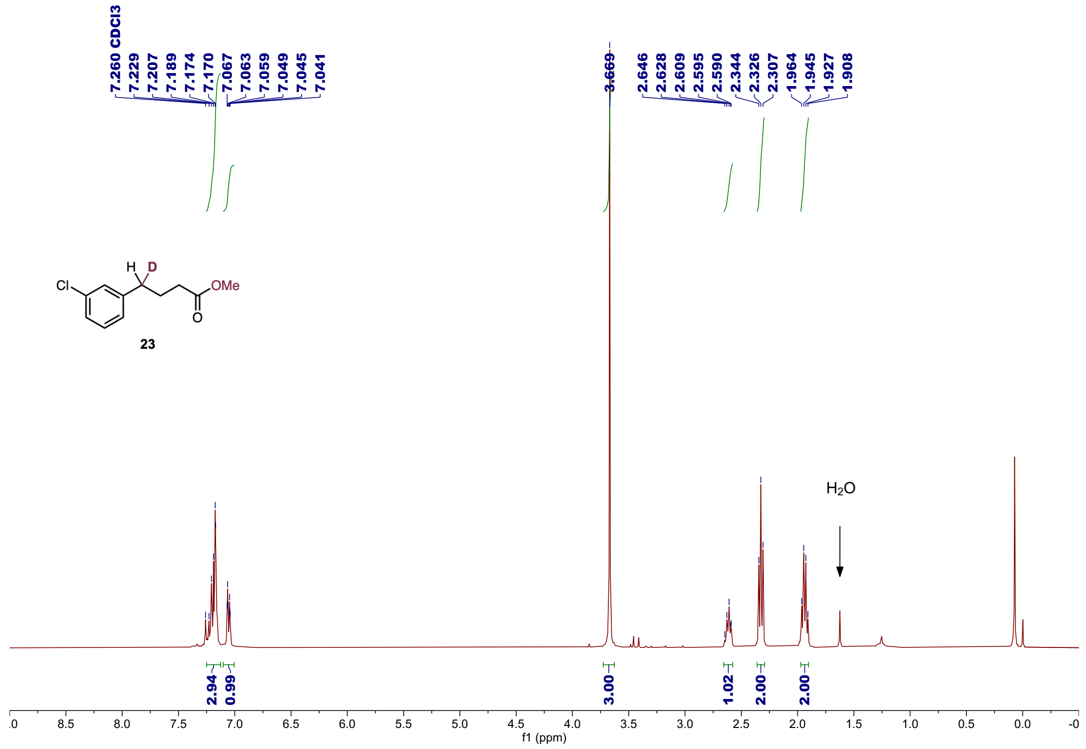

^{13}C NMR 100 MHz, $CDCl_3$

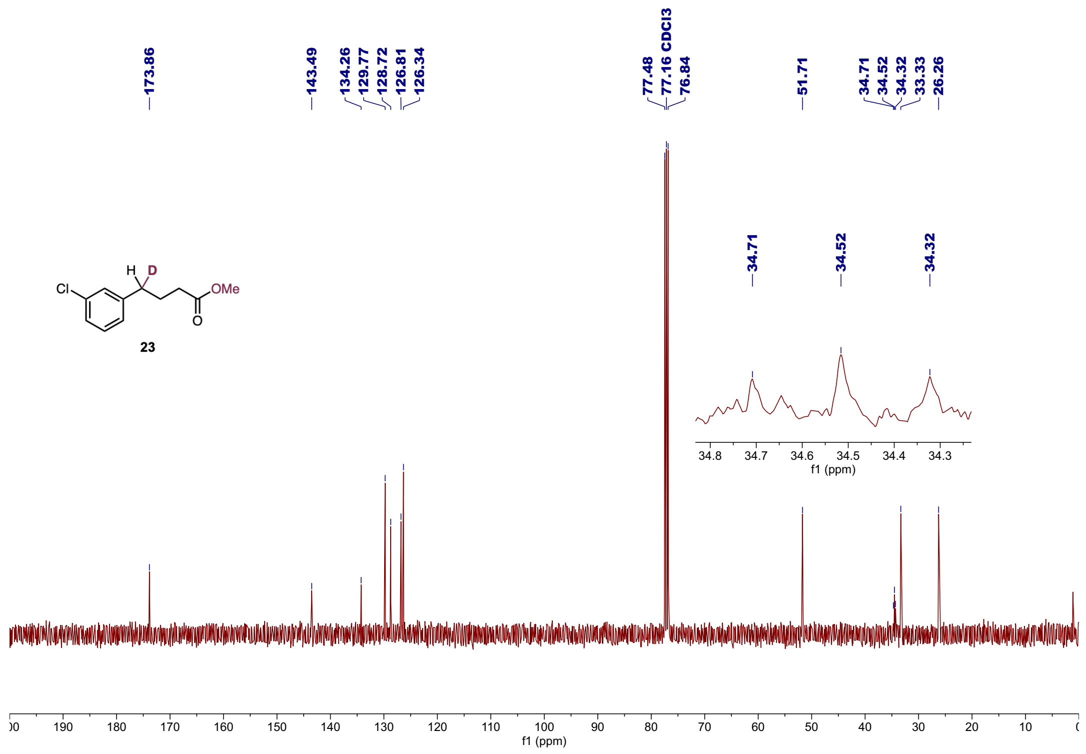

^{1}H NMR 400 MHz, $CDCl_3$

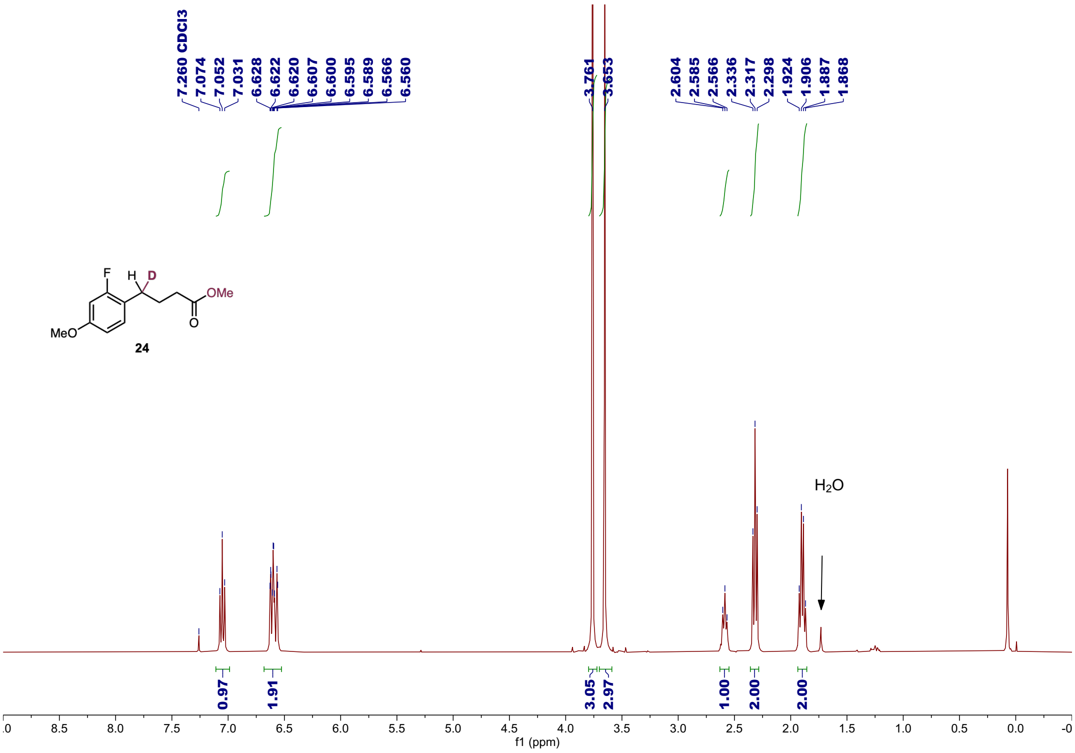

^{13}C NMR 100 MHz, $CDCl_3$

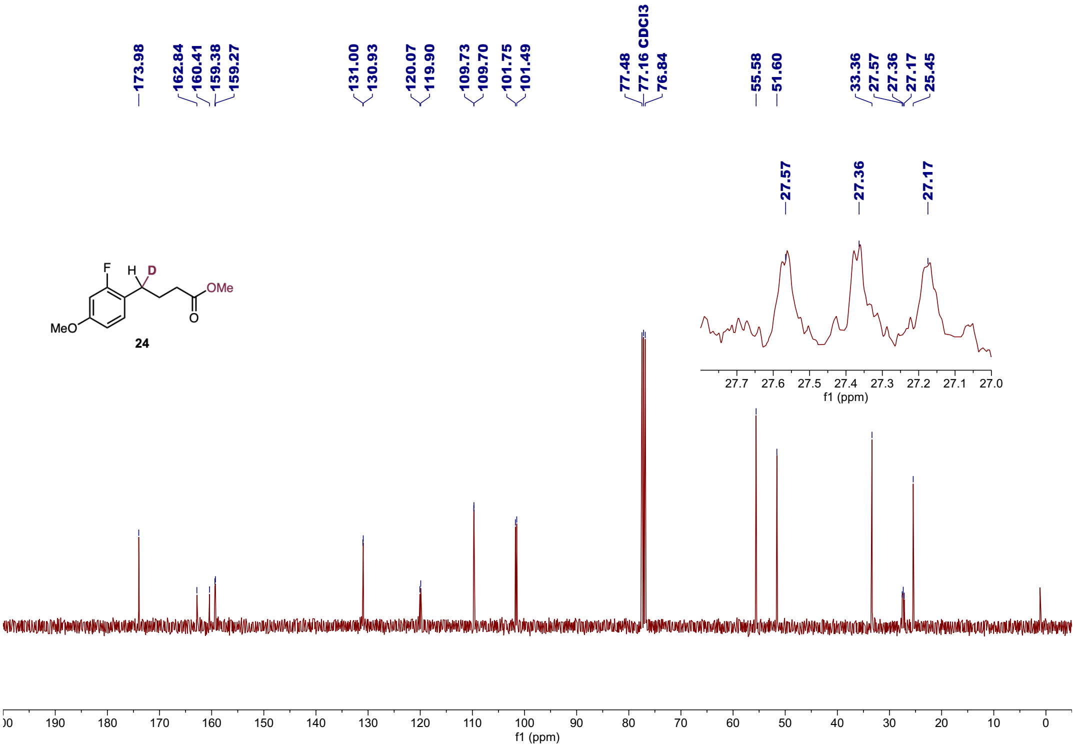

^{19}F NMR 376 MHz, $CDCl_3$

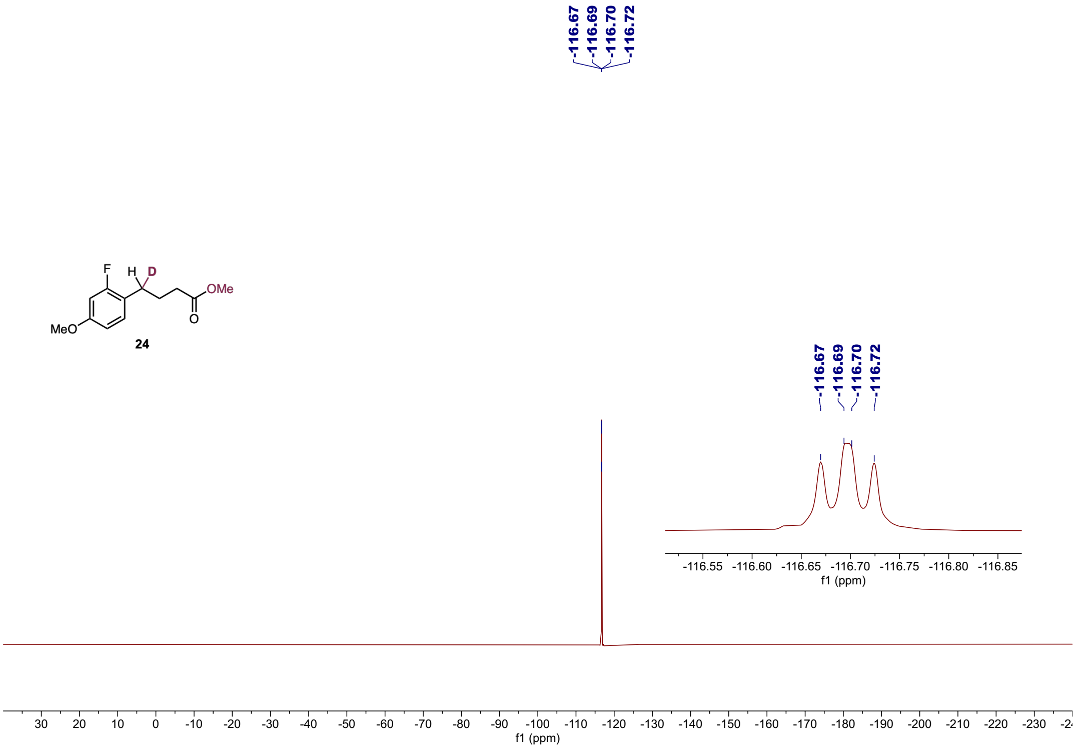

^{1}H NMR 400 MHz, $CDCl_3$

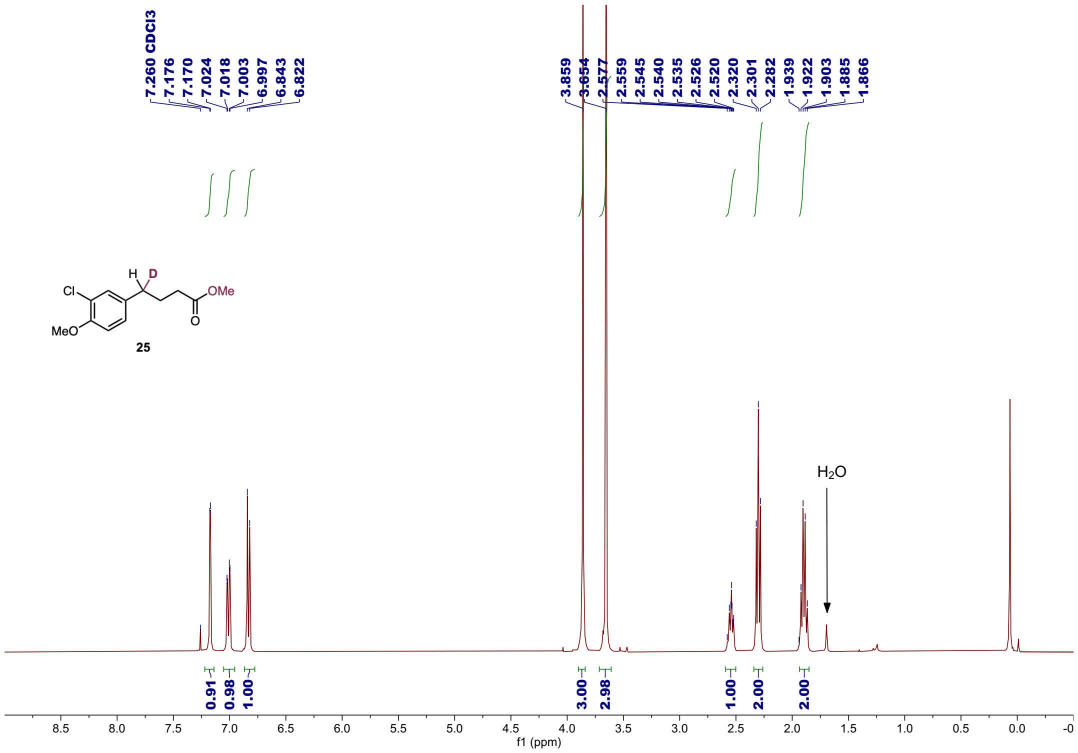

^{13}C NMR 100 MHz, $CDCl_3$

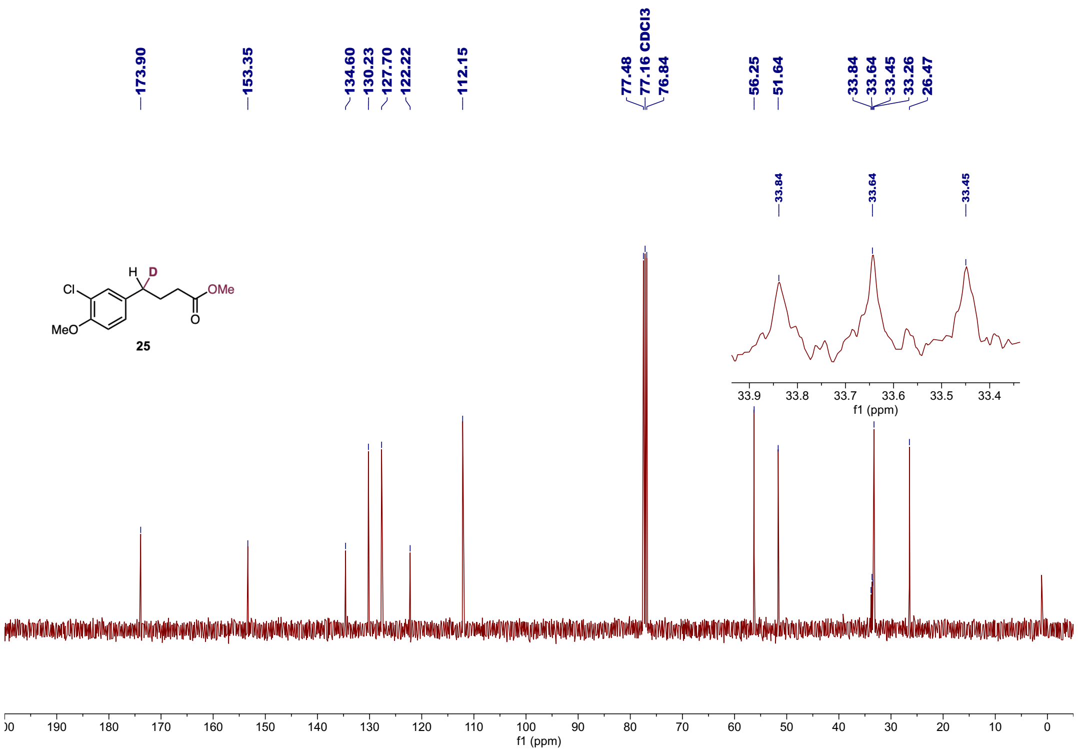

^{1}H NMR 400 MHz, $CDCl_3$

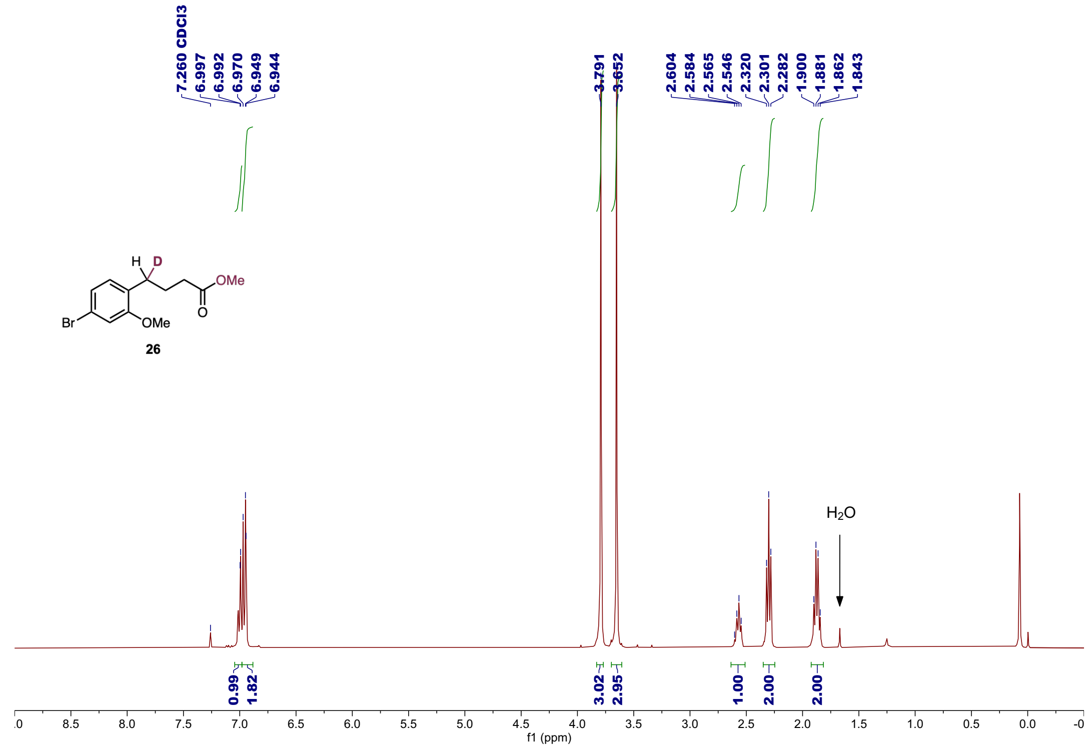

^{13}C NMR 100 MHz, $CDCl_3$

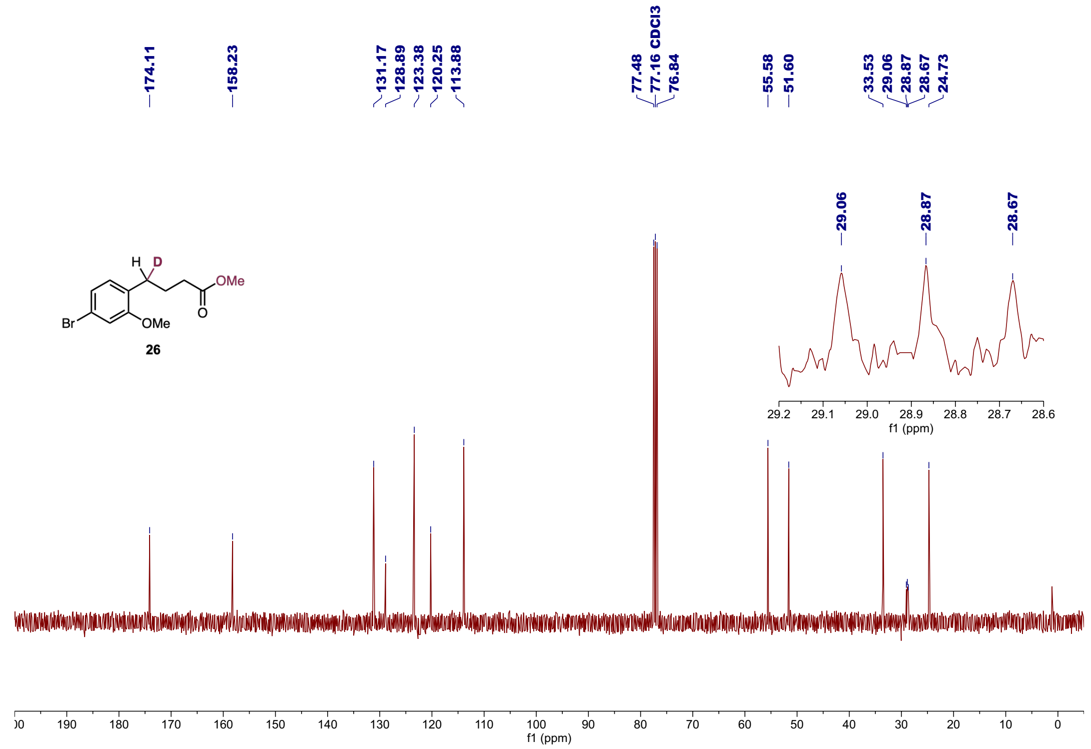

^{1}H NMR 400 MHz, $CDCl_3$

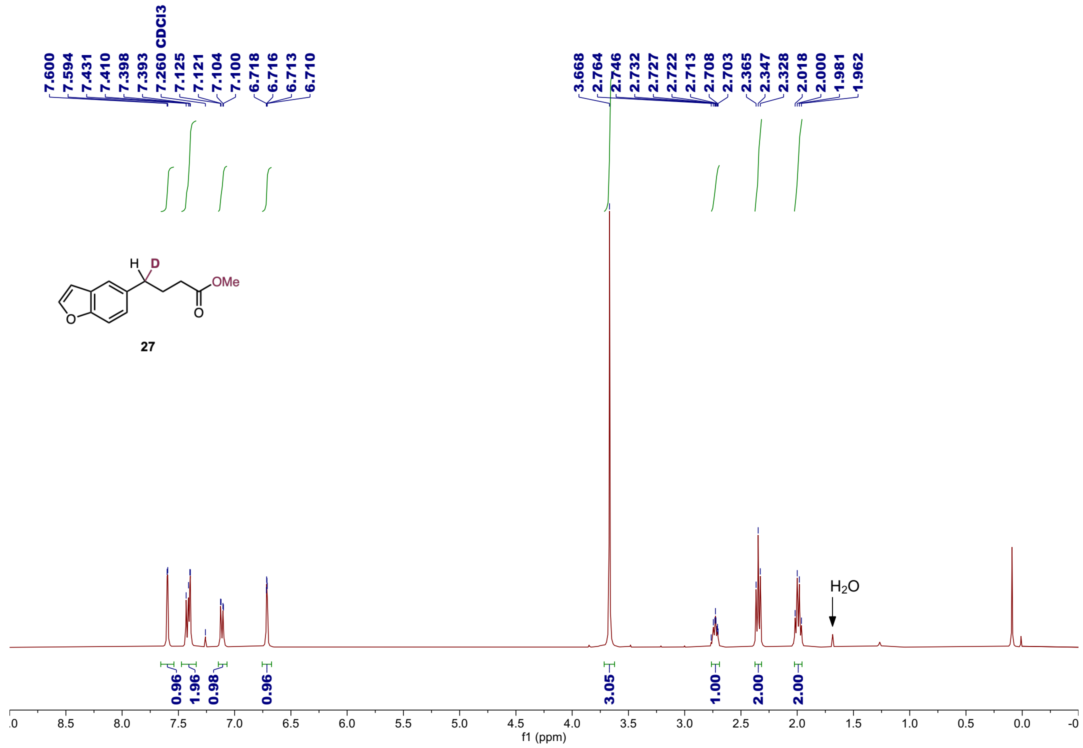

^{13}C NMR 100 MHz, $CDCl_3$

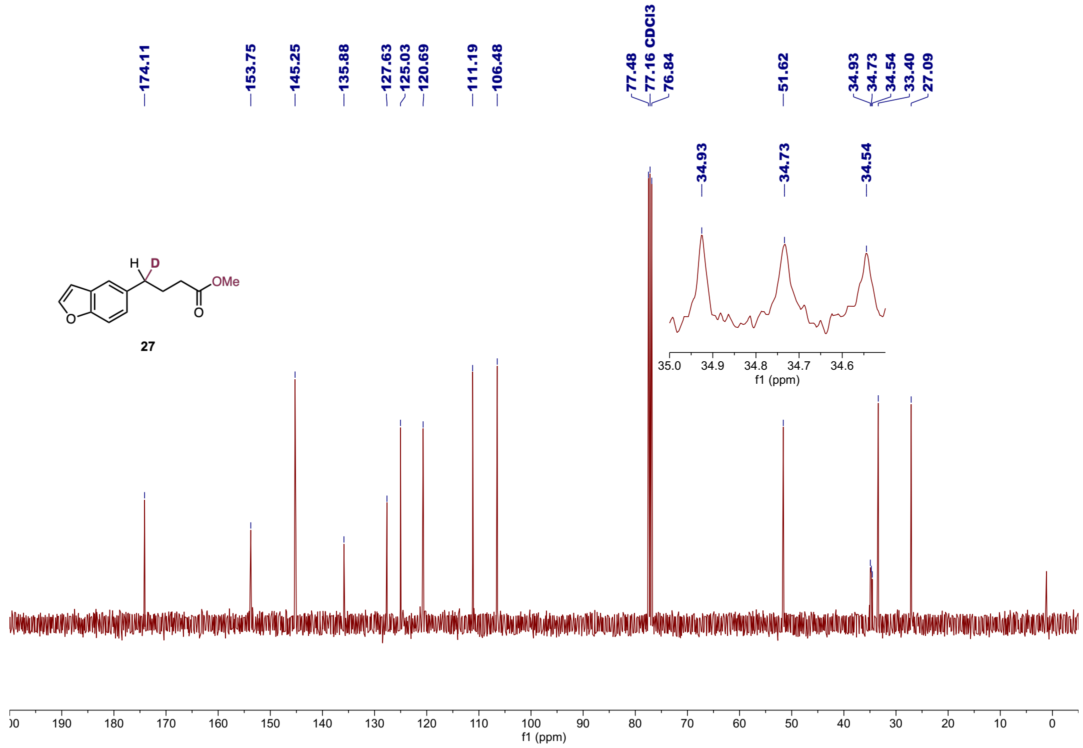

^{1}H NMR 400 MHz, $CDCl_3$

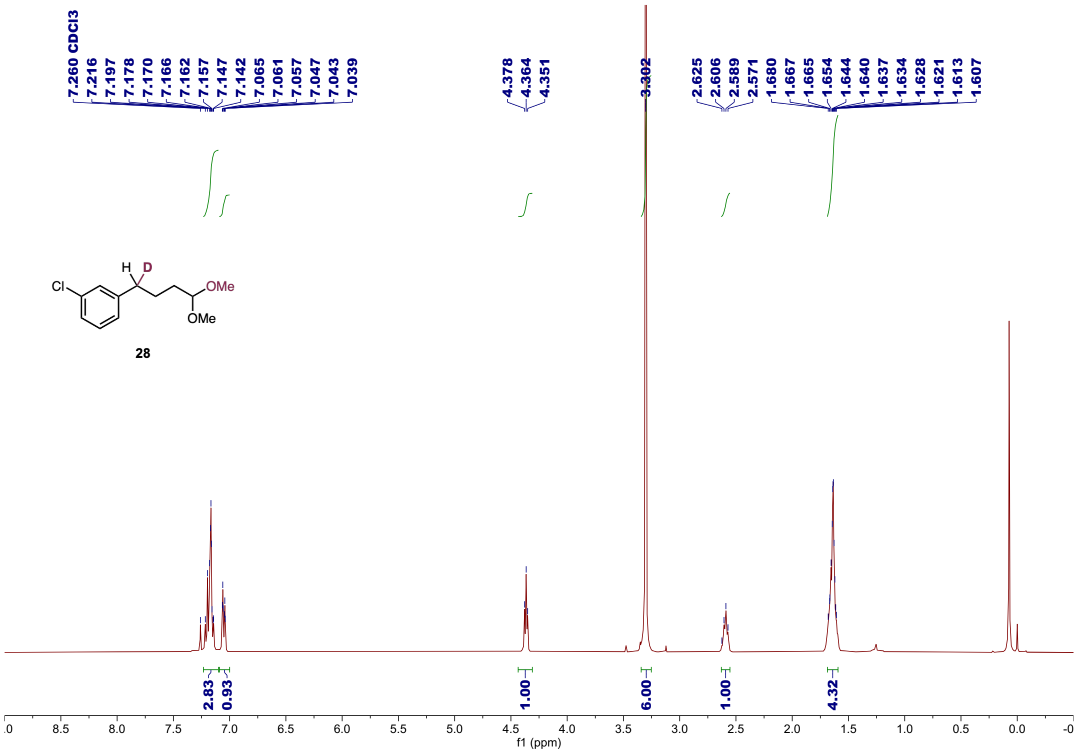

^{13}C NMR 100 MHz, $CDCl_3$

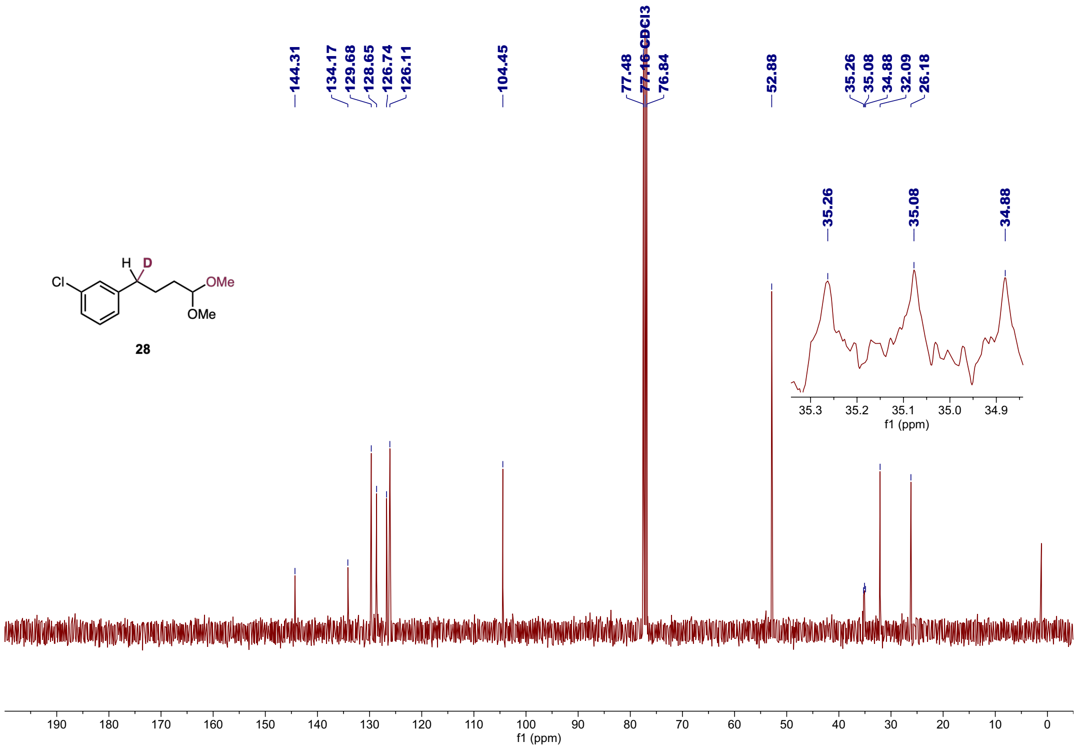

^{1}H NMR 400 MHz, CDCl$_3$

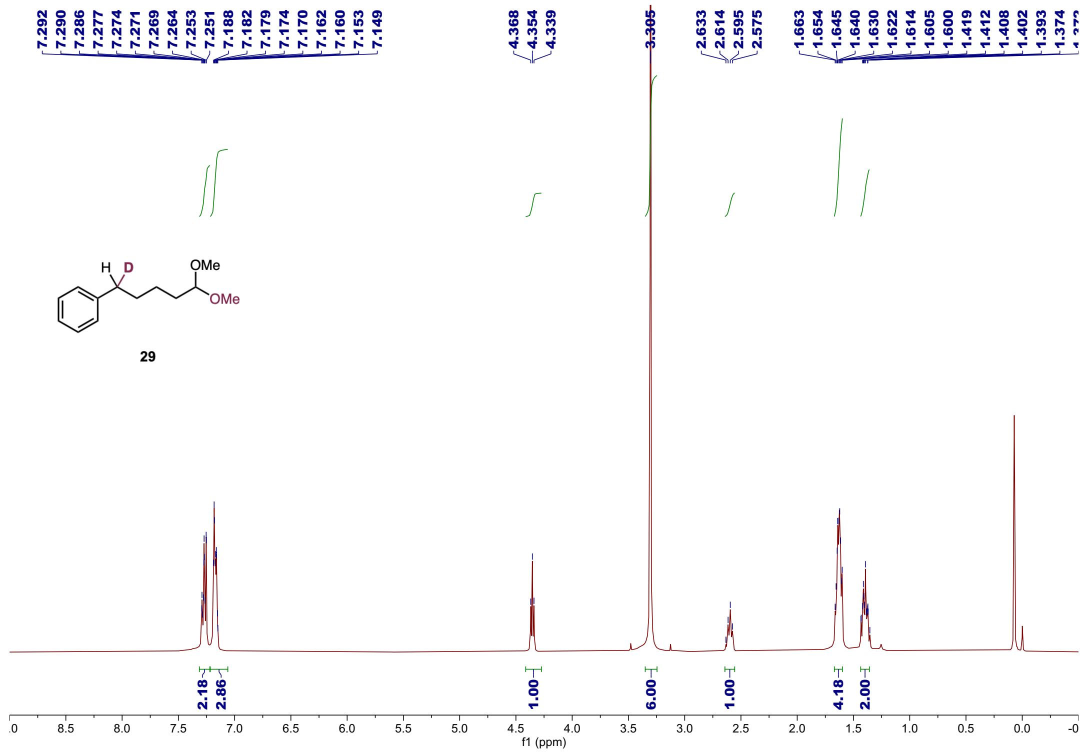

^{13}C NMR 100 MHz, CDCl$_3$

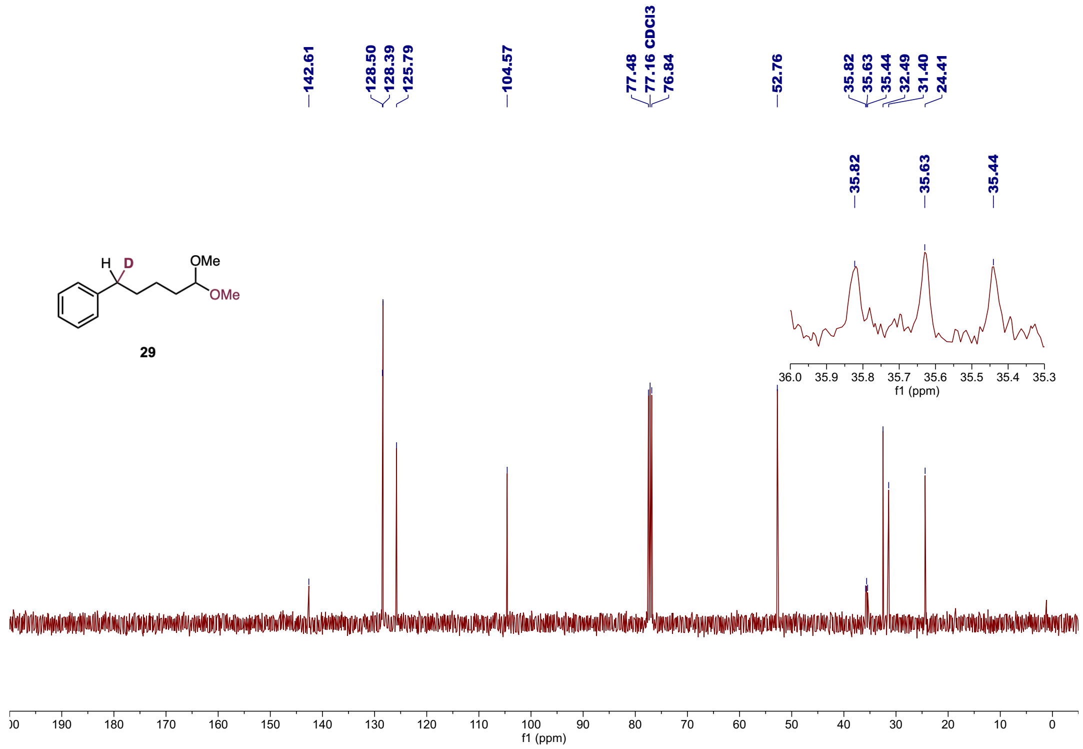

^{1}H NMR 400 MHz, CDCl$_3$

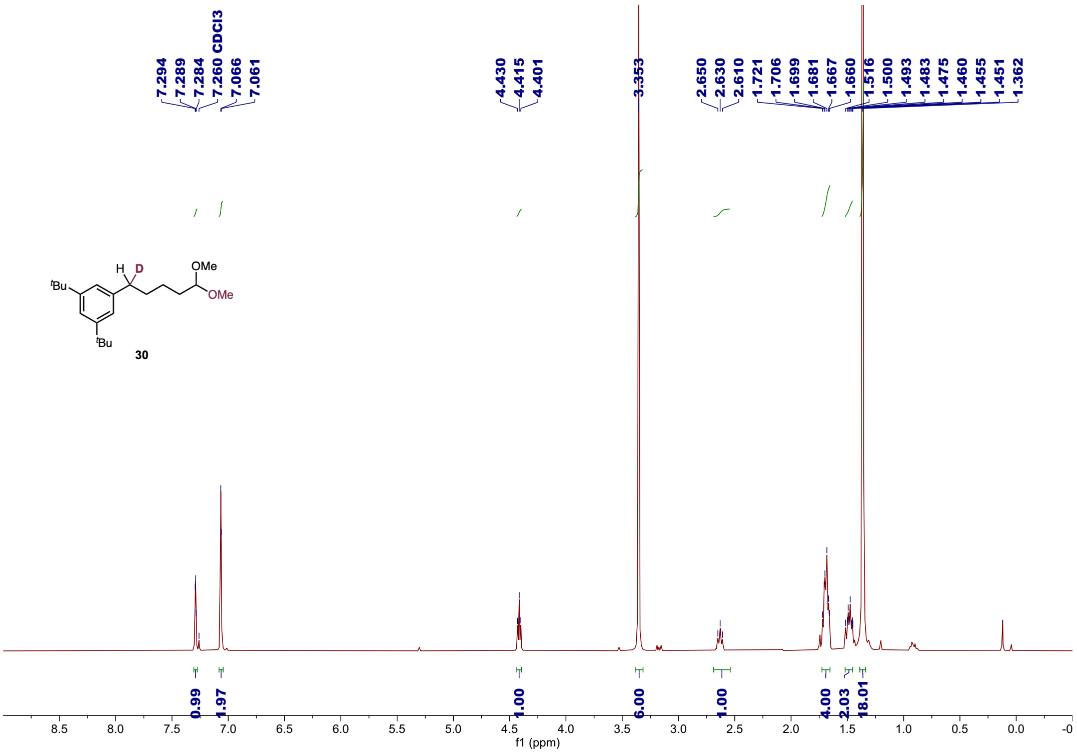

^{13}C NMR 100 MHz, CDCl$_3$

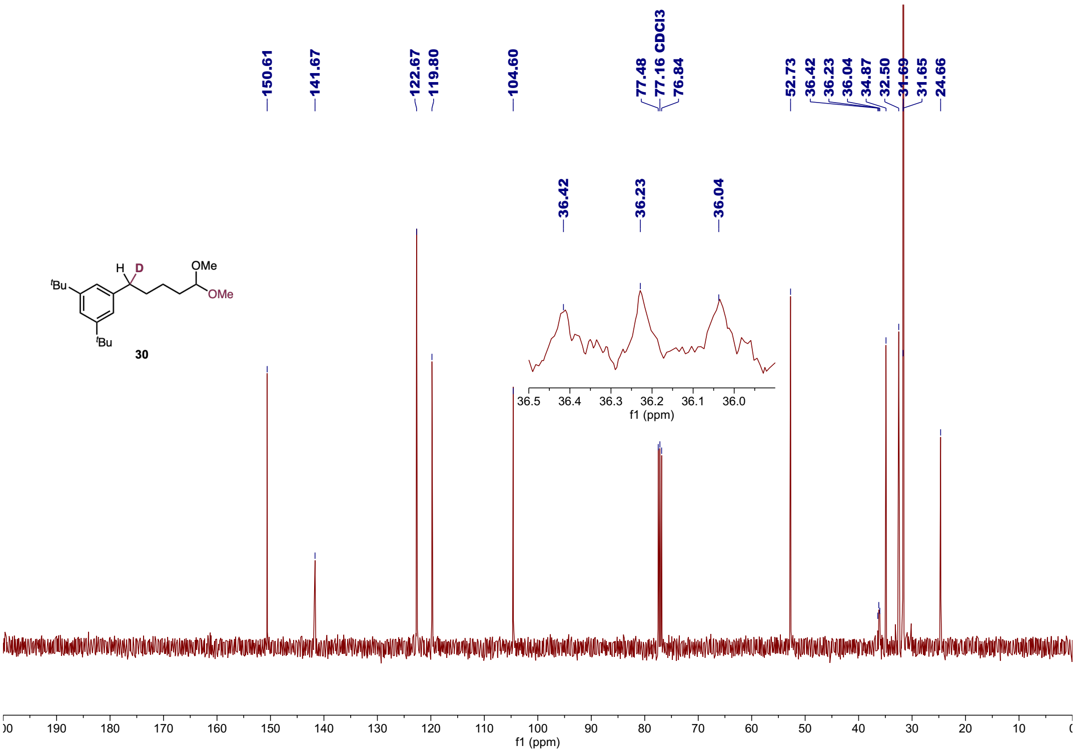

^{1}H NMR 400 MHz, $CDCl_3$

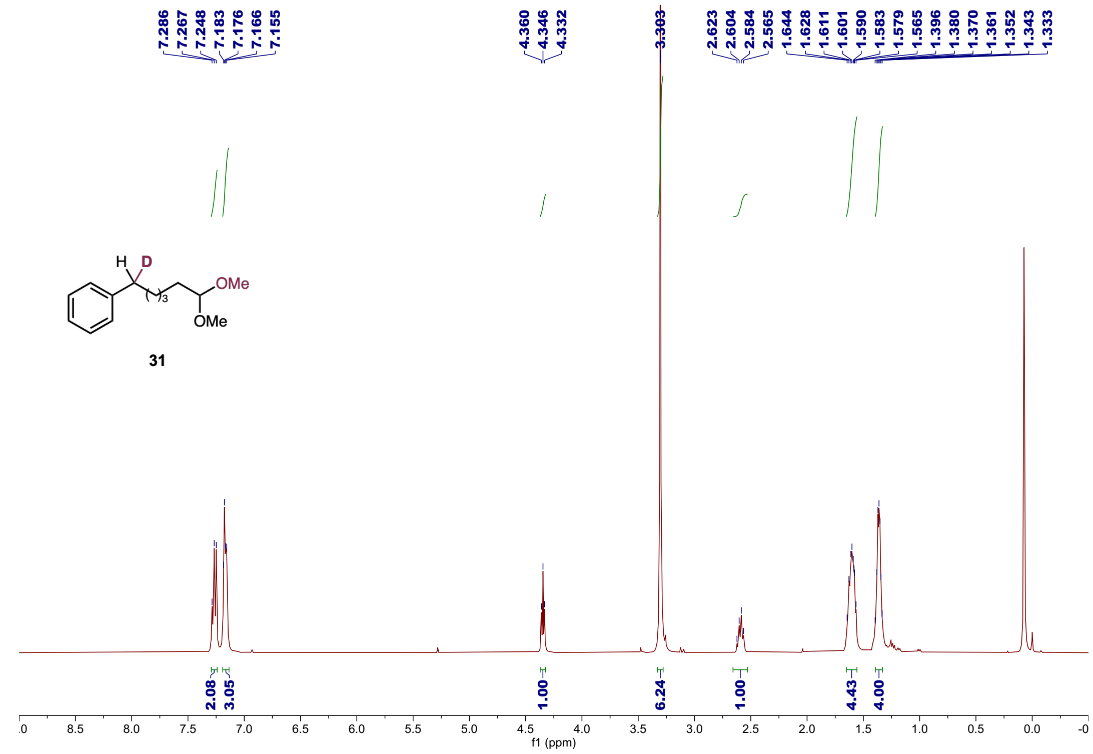

^{13}C NMR 100 MHz, $CDCl_3$

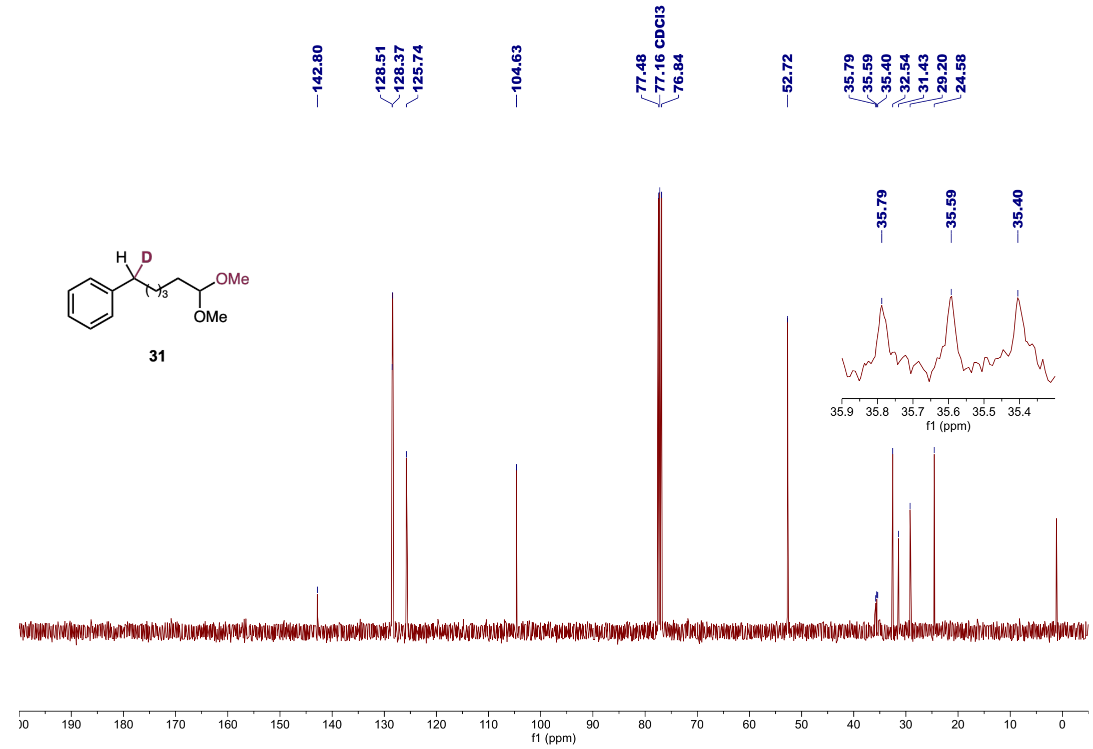

^{1}H NMR 400 MHz, $CDCl_3$

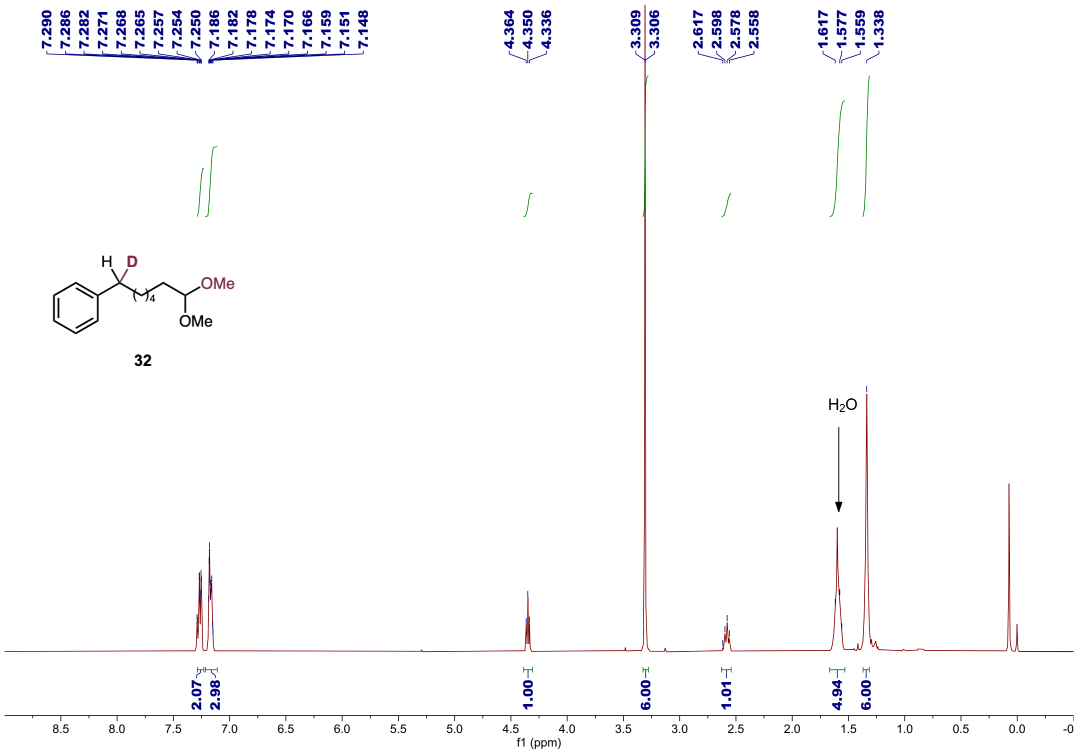

^{13}C NMR 100 MHz, $CDCl_3$

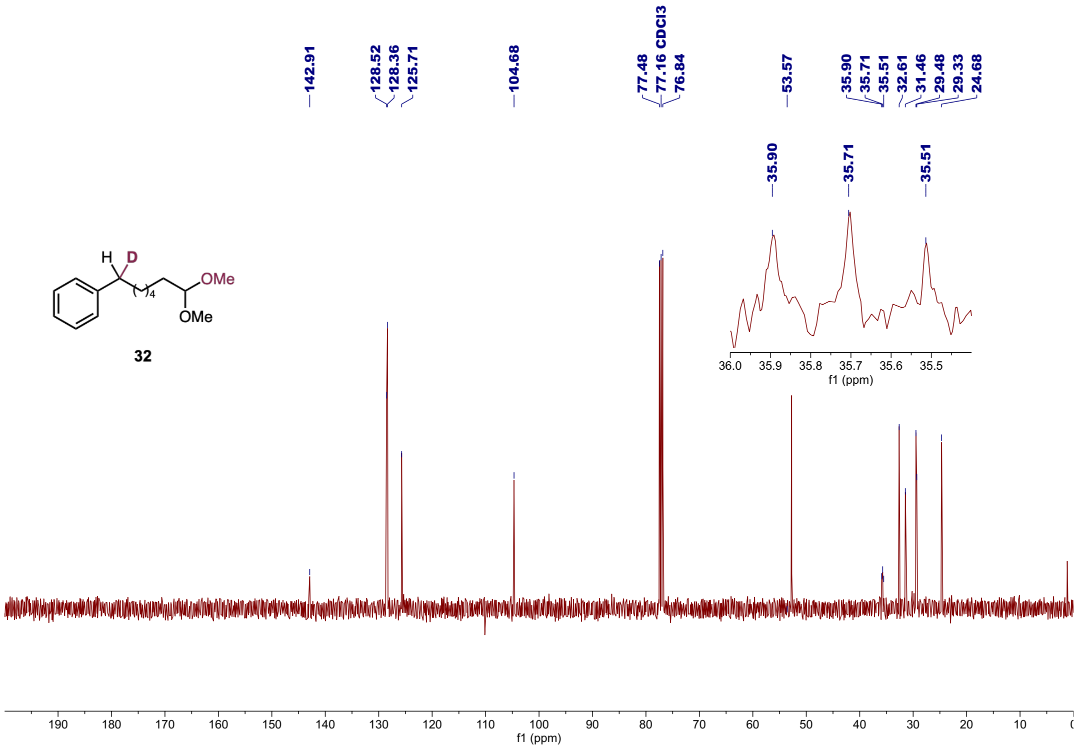

^{1}H NMR 500 MHz, $CDCl_3$

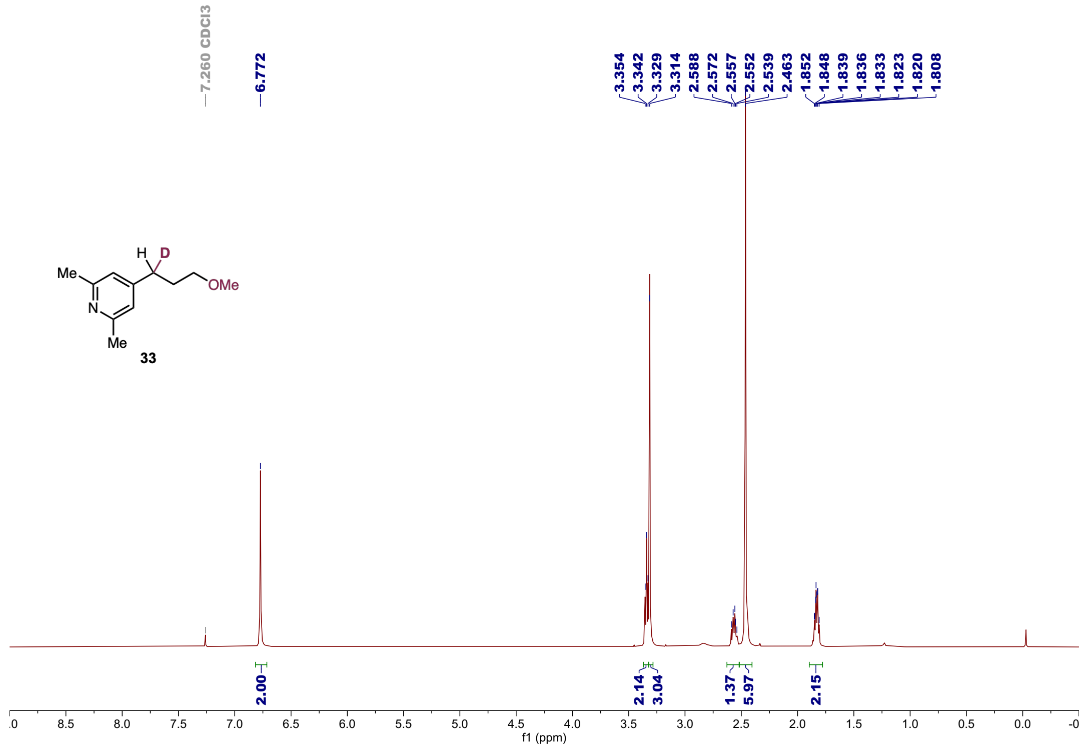

^{13}C NMR 126 MHz, $CDCl_3$

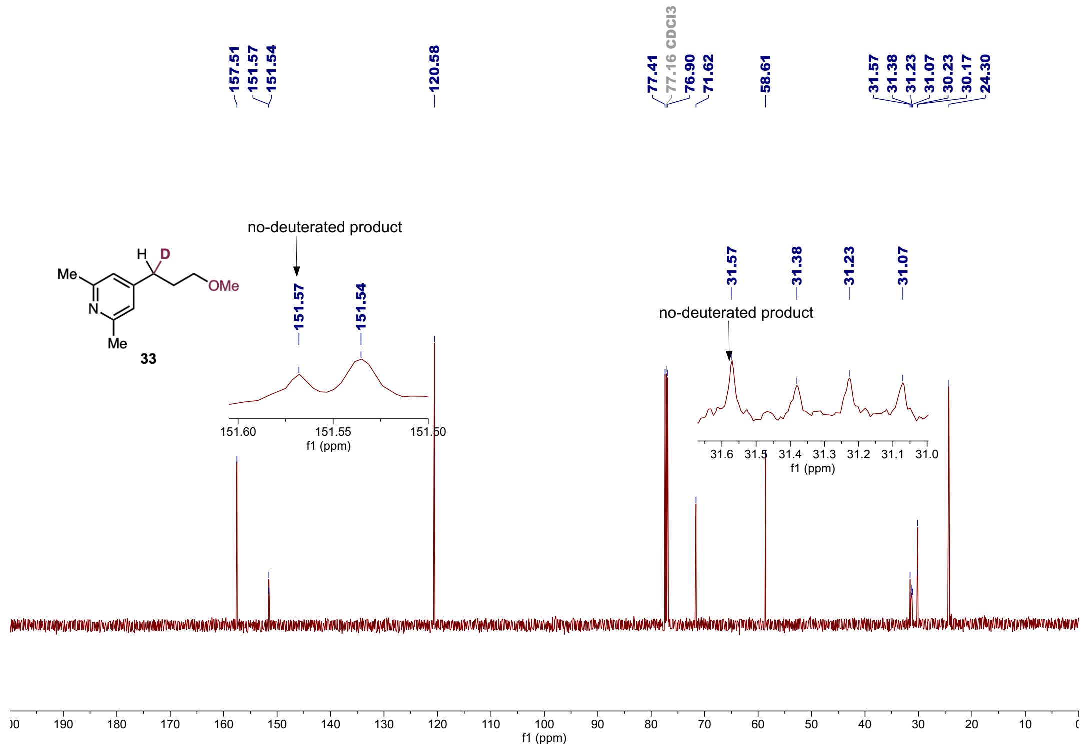

^{1}H NMR 500 MHz, $CDCl_3$

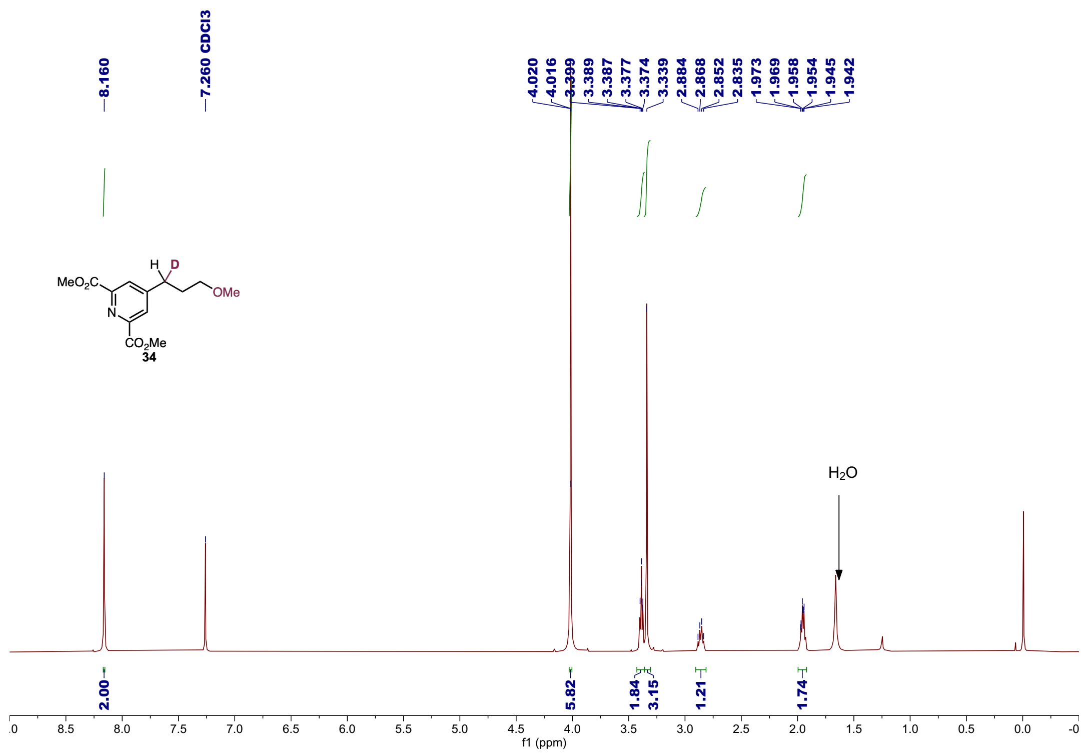

^{13}C NMR 100 MHz, $CDCl_3$

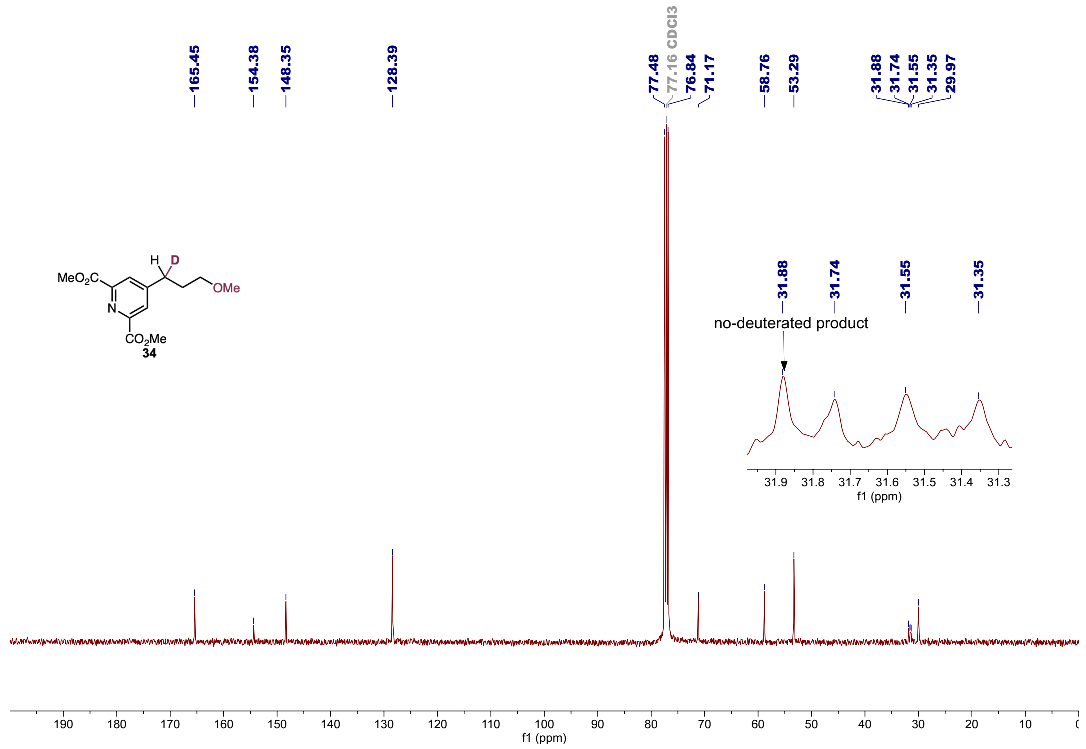

^{1}H NMR 500 MHz, CDCl$_3$

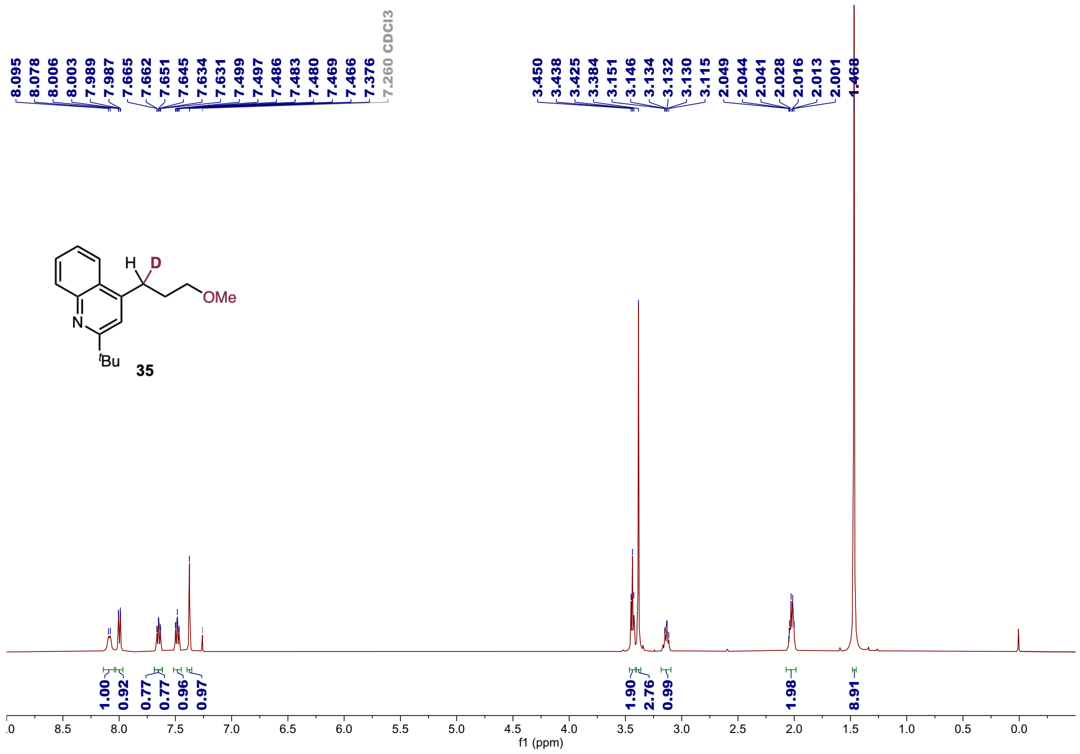

^{13}C NMR 126 MHz, CDCl$_3$

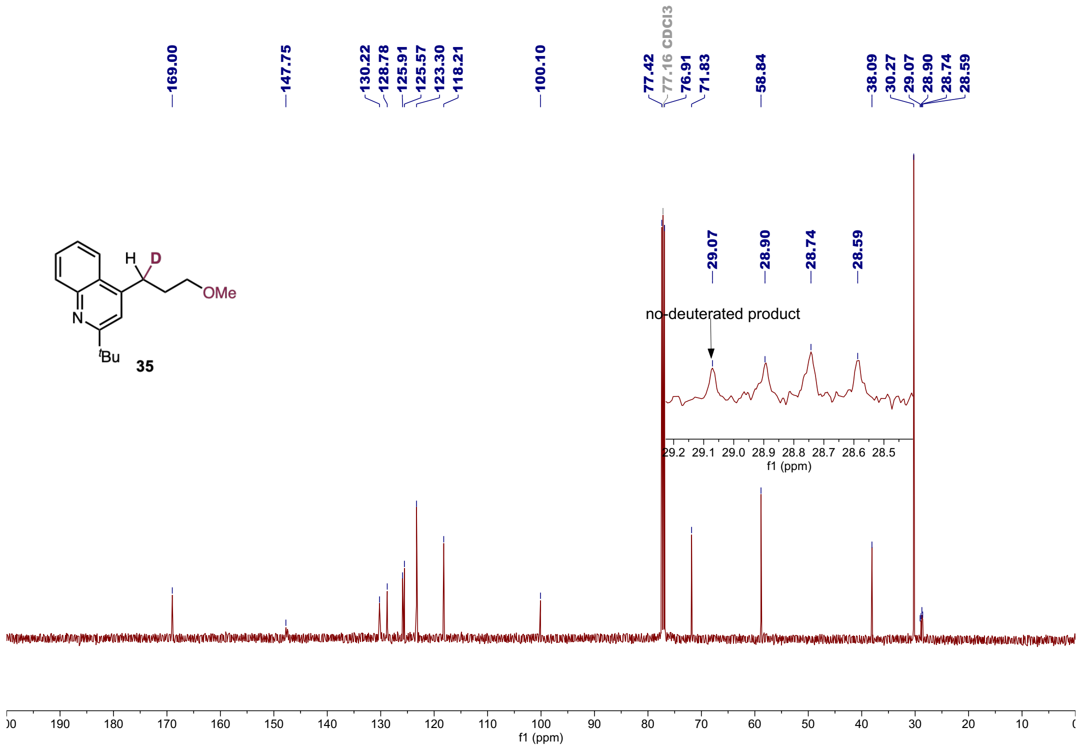

^{1}H NMR 400 MHz, $CDCl_3$

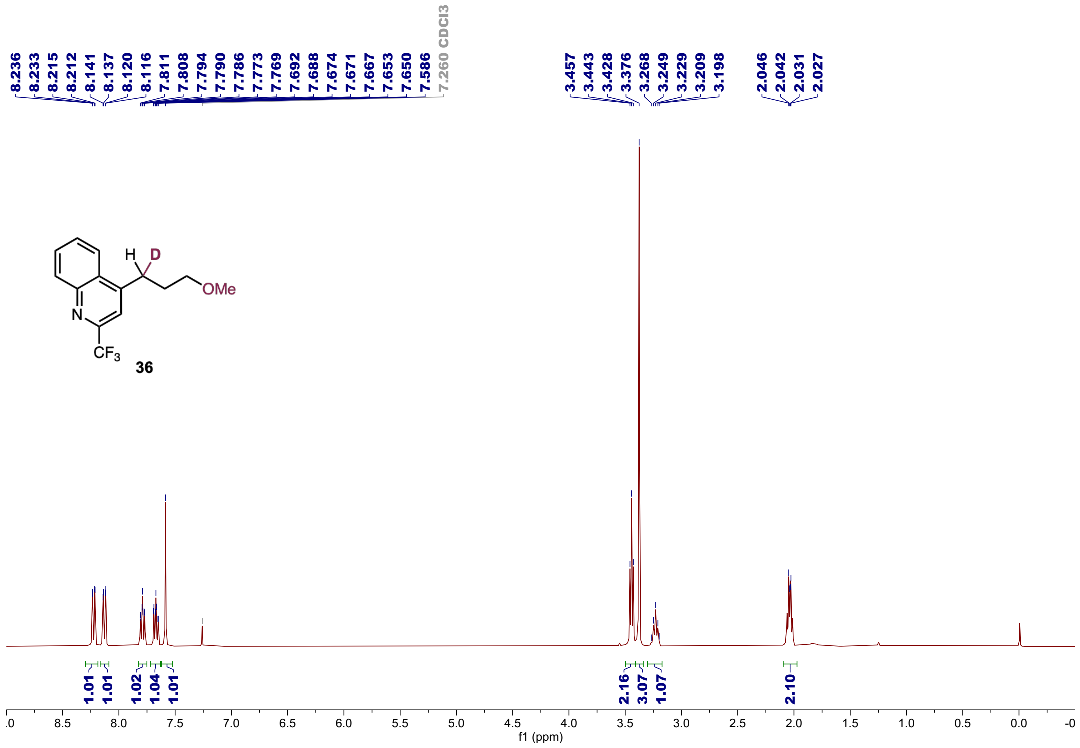

^{13}C NMR 126 MHz, $CDCl_3$

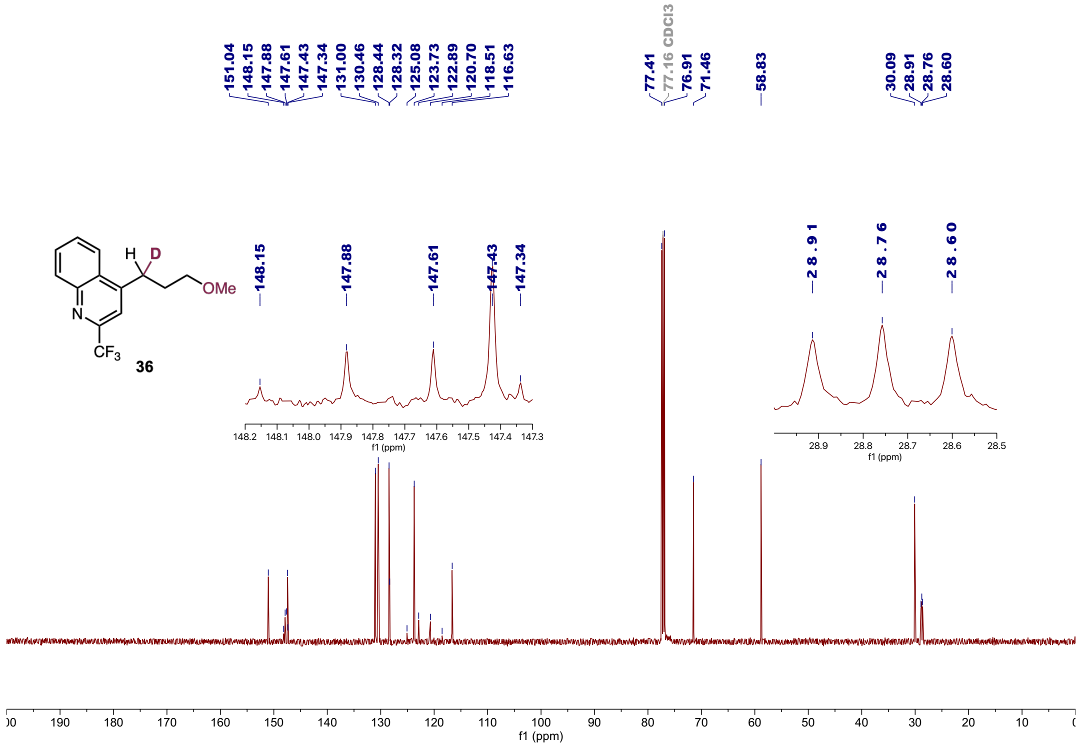

^{19}F NMR 376 MHz, $CDCl_3$

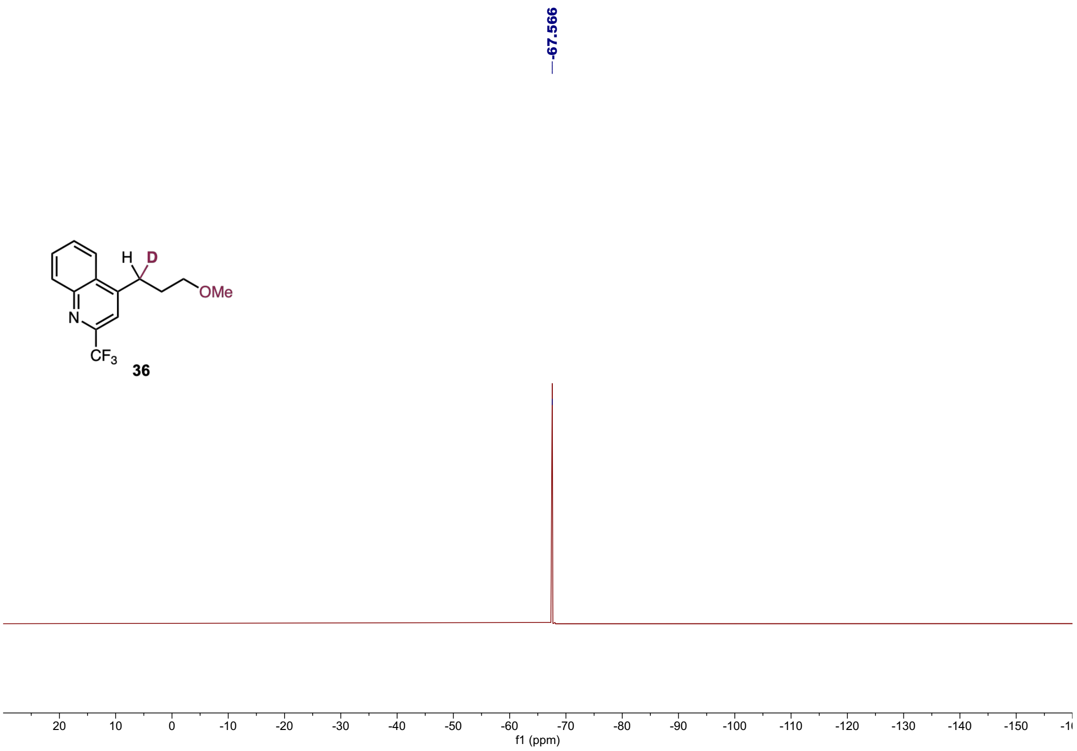

^{1}H NMR 500 MHz, $CDCl_3$

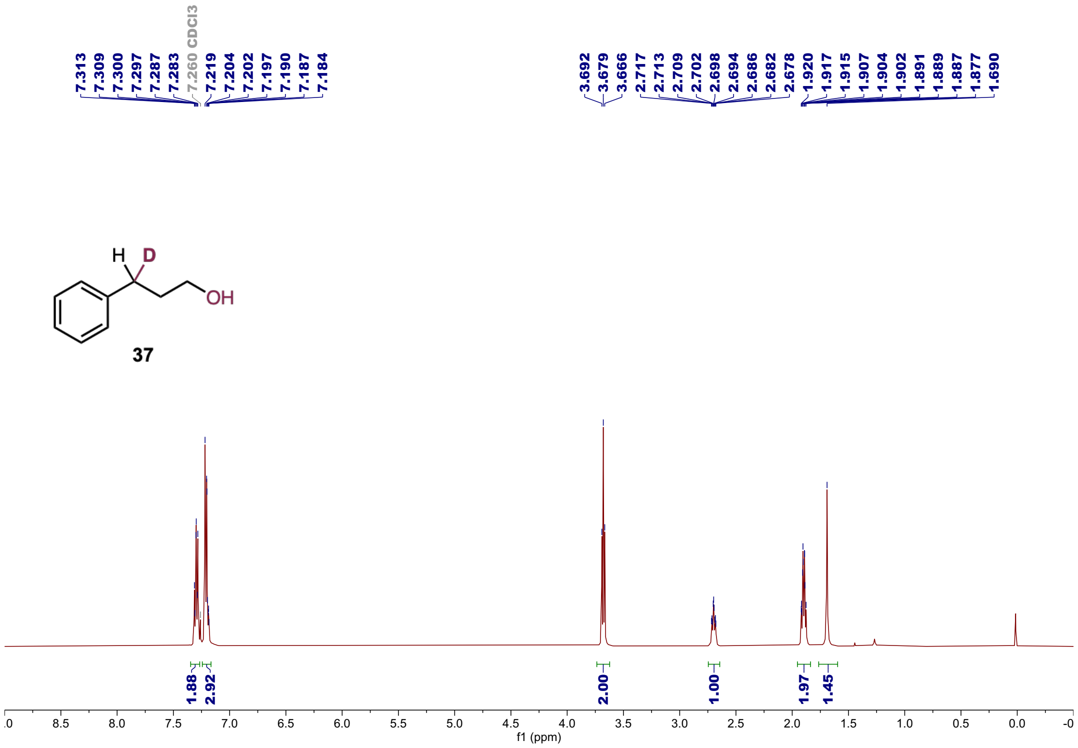

^{13}C NMR 126 MHz, $CDCl_3$

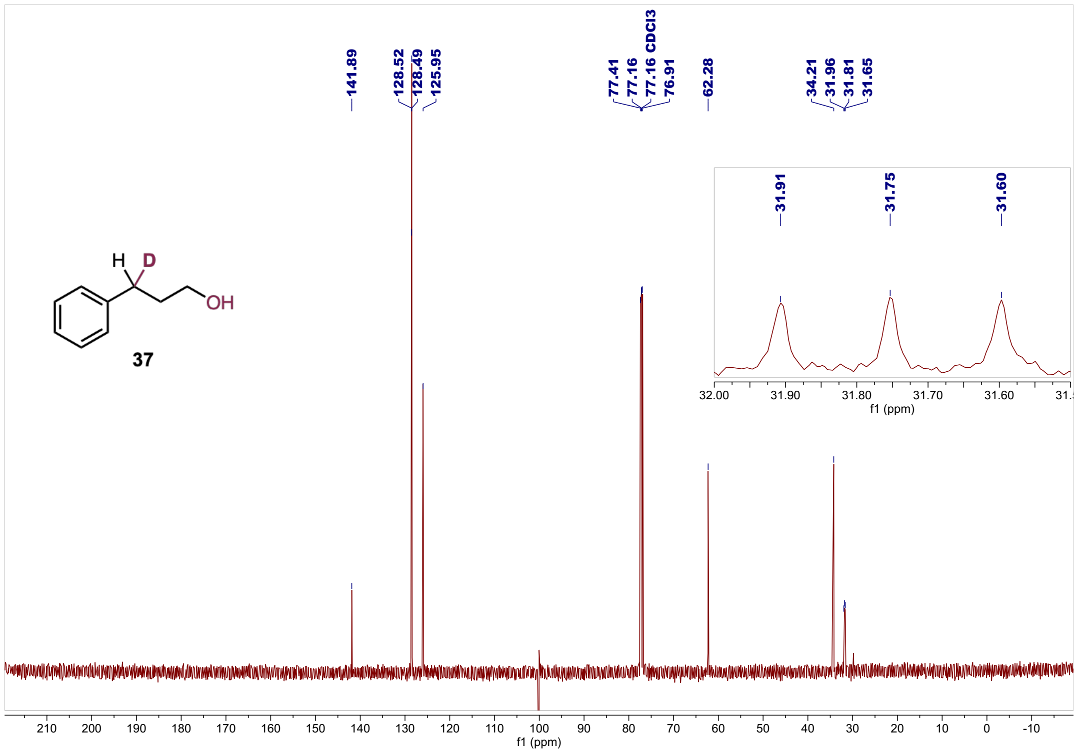

^{1}H NMR 500 MHz, $CDCl_3$

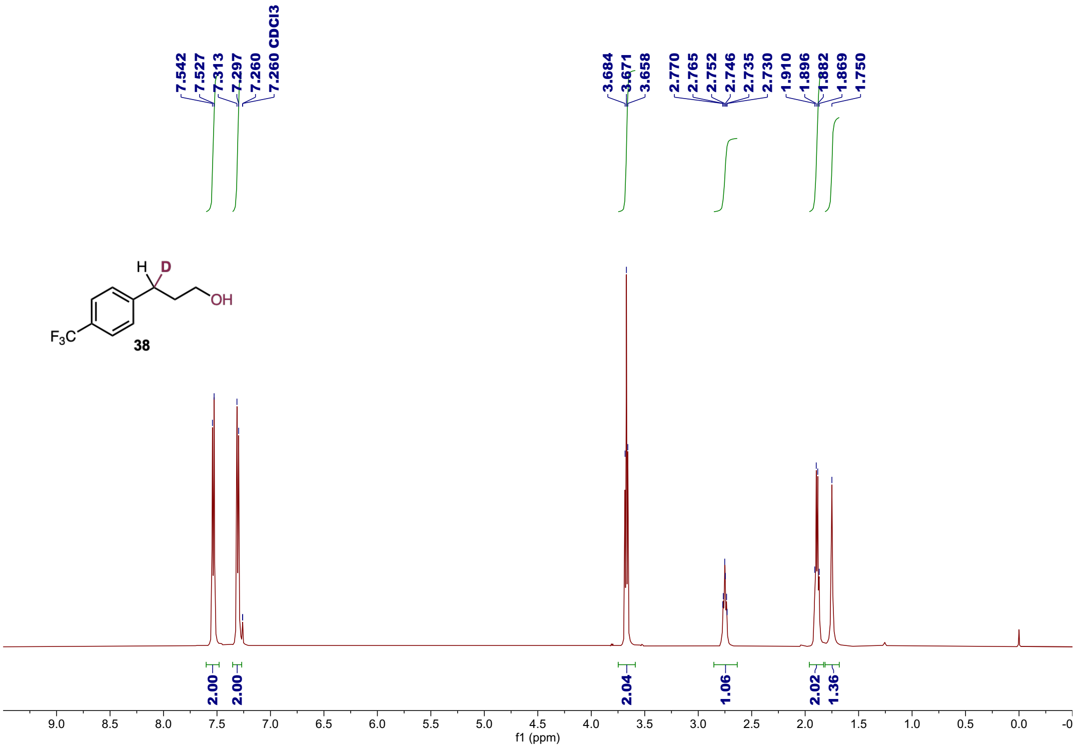

^{13}C NMR 126 MHz, $CDCl_3$

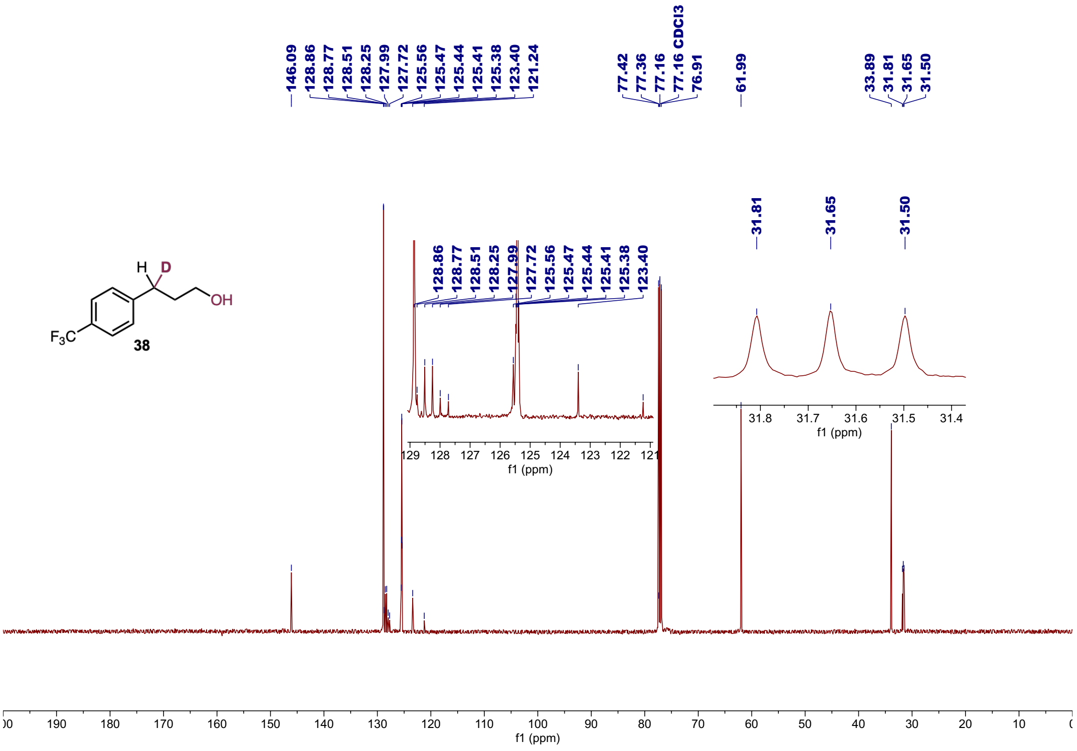

^{19}F NMR 376 MHz, $CDCl_3$

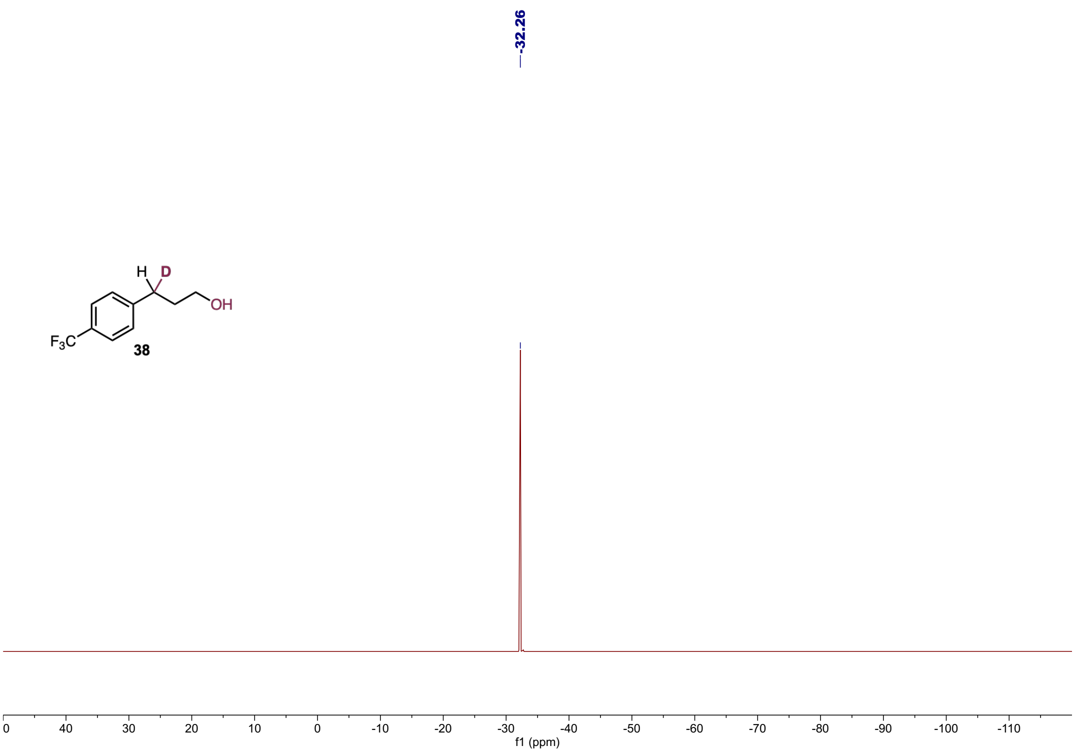

^{1}H NMR 400 MHz, $CDCl_3$

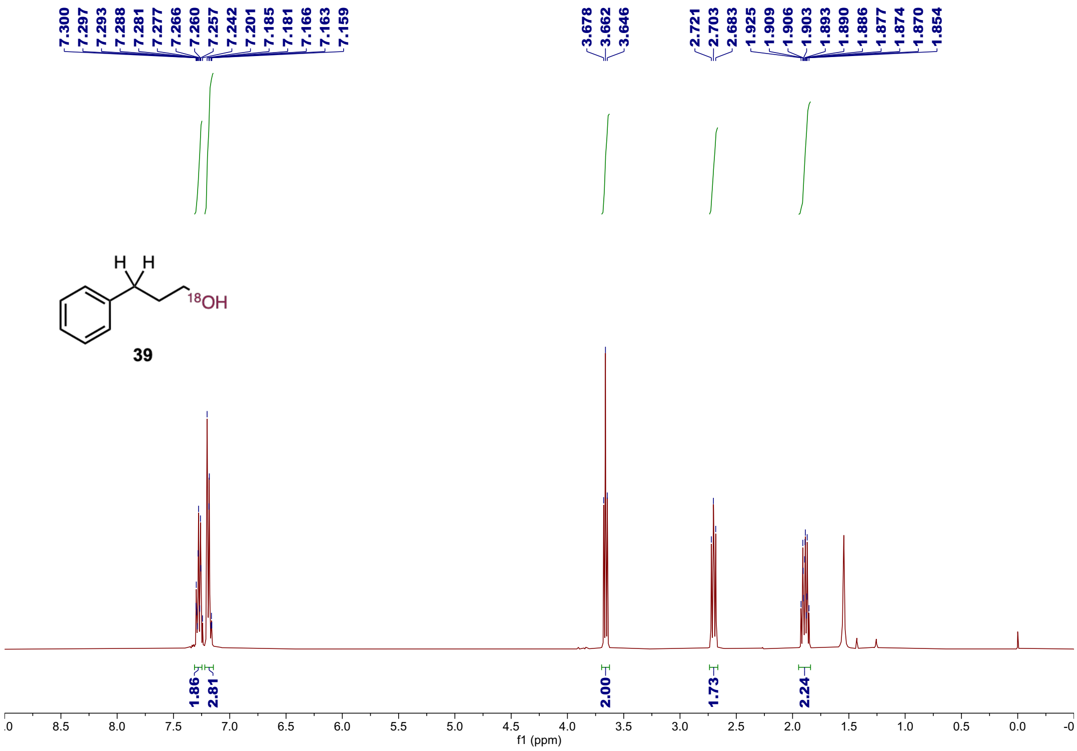

^{13}C NMR 100 MHz, $CDCl_3$

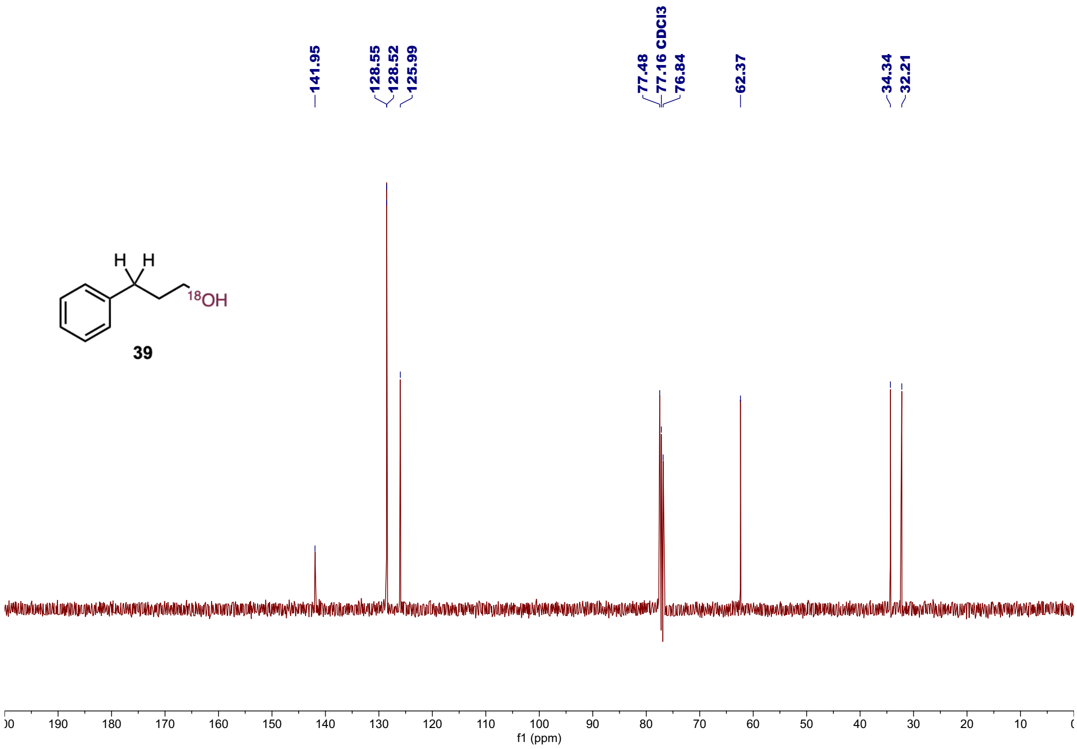

^{1}H NMR 400 MHz, $CDCl_3$

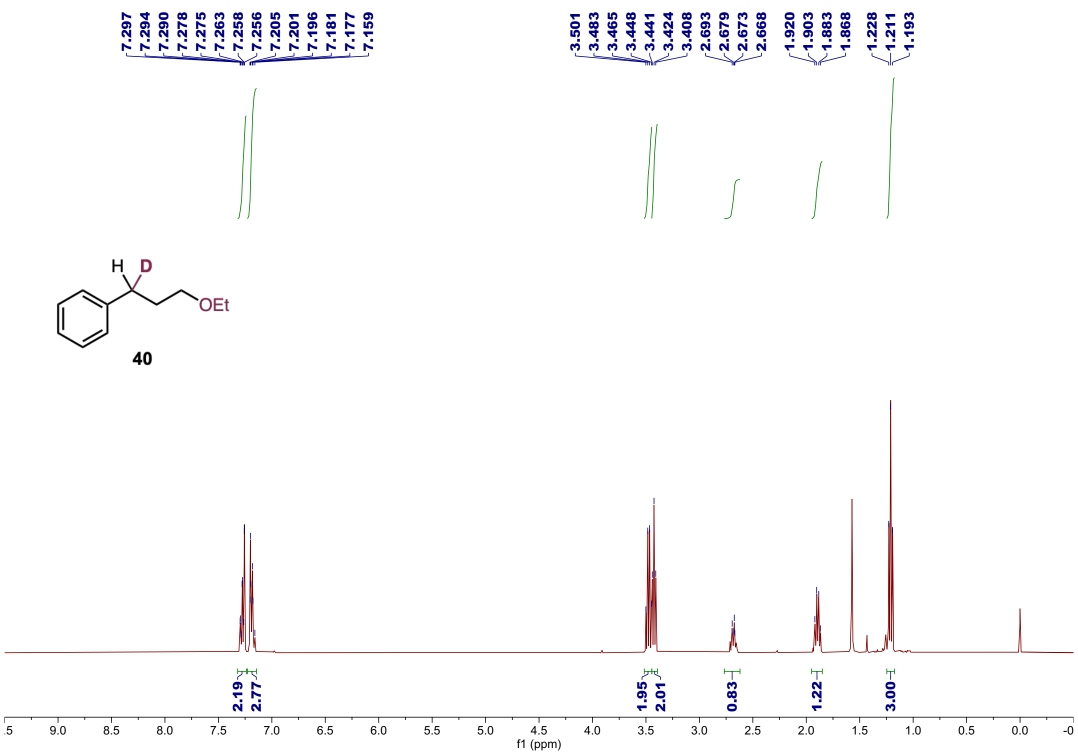

^{13}C NMR 100 MHz, $CDCl_3$

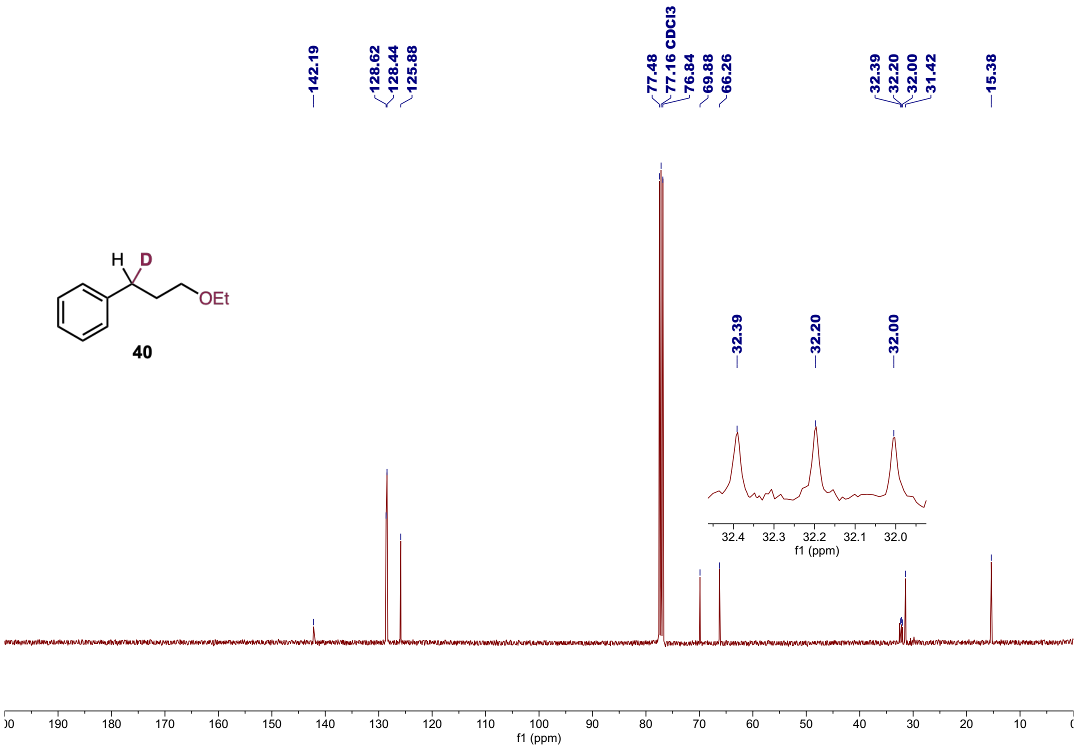

^{1}H NMR 400 MHz, $CDCl_3$

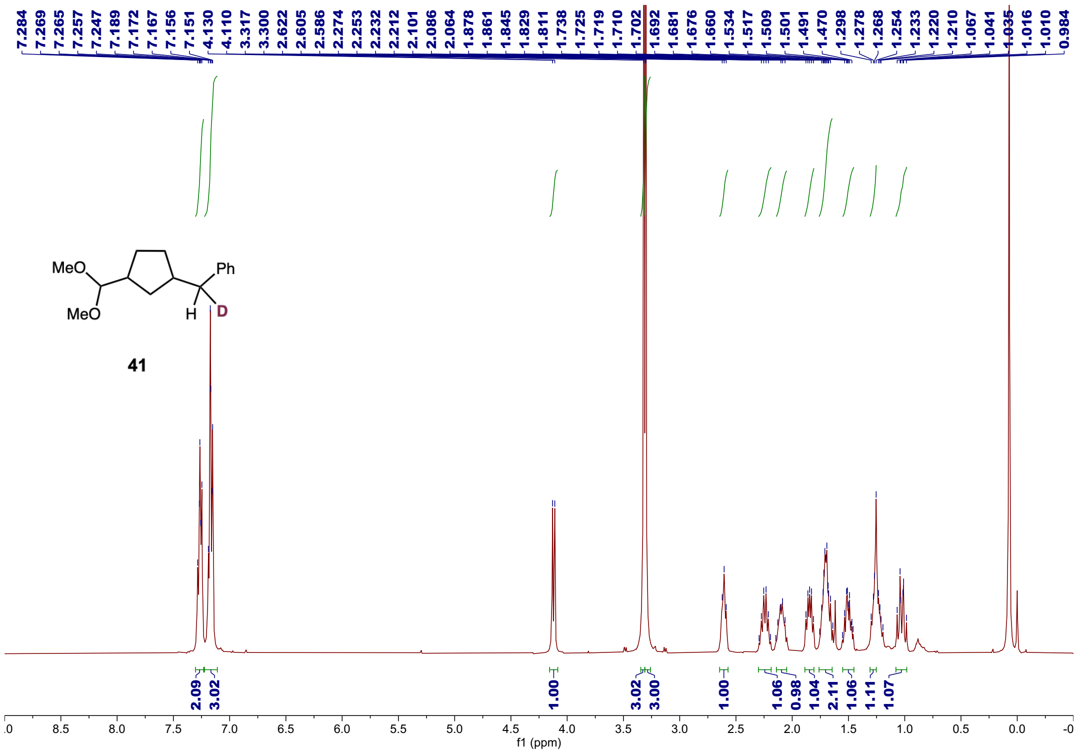

^{13}C NMR 100 MHz, $CDCl_3$

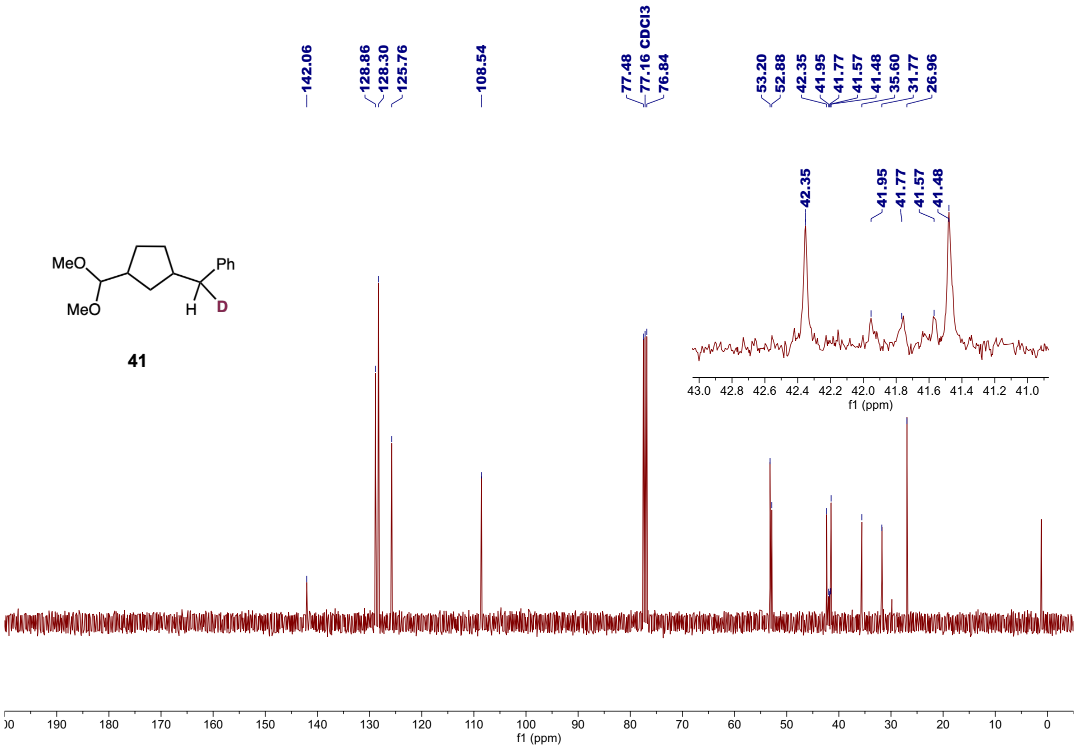

^{1}H NMR 400 MHz, $CDCl_3$

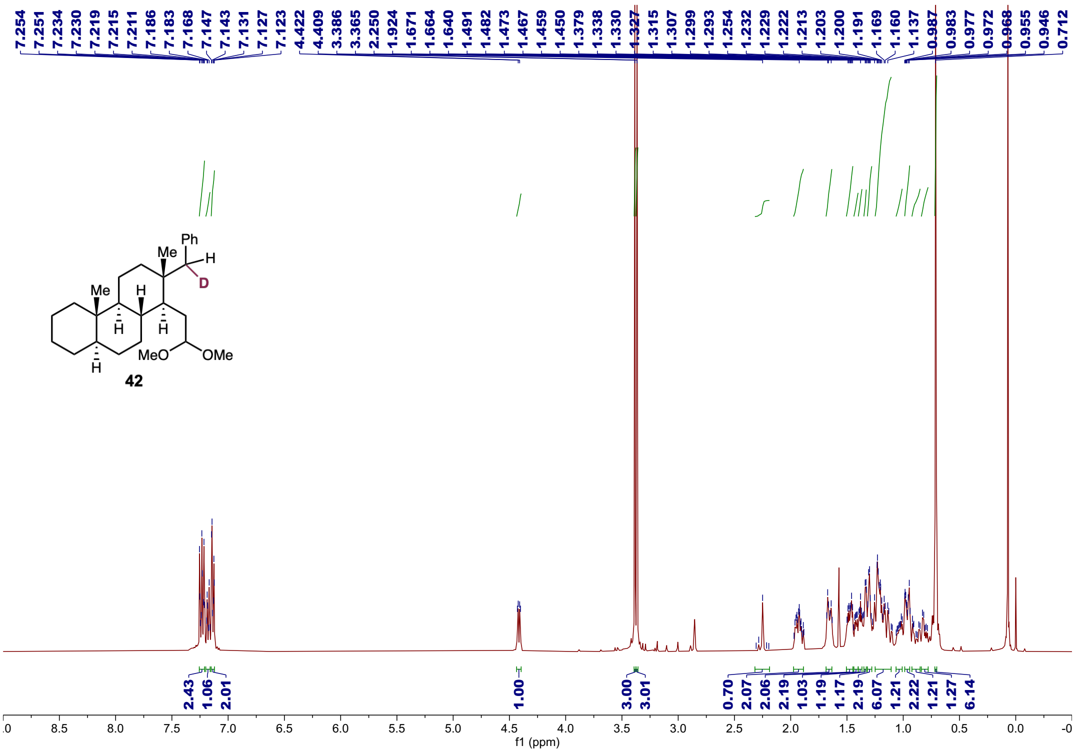

^{13}C NMR 100 MHz, $CDCl_3$

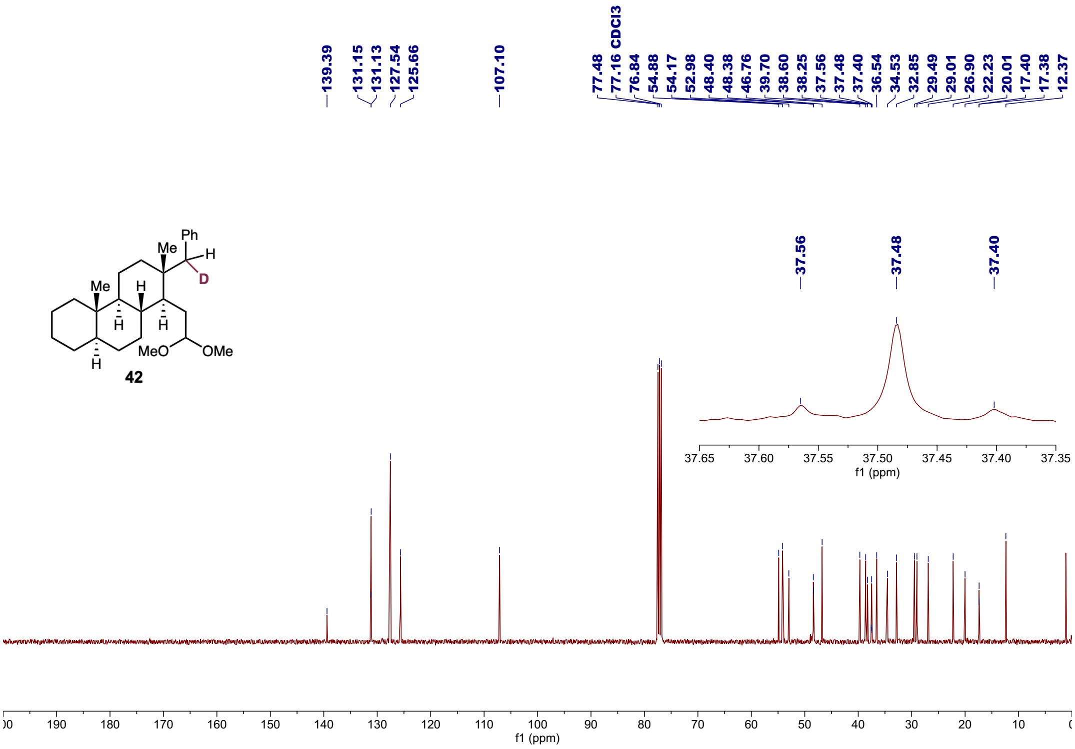

^{1}H NMR 400 MHz, $CDCl_3$

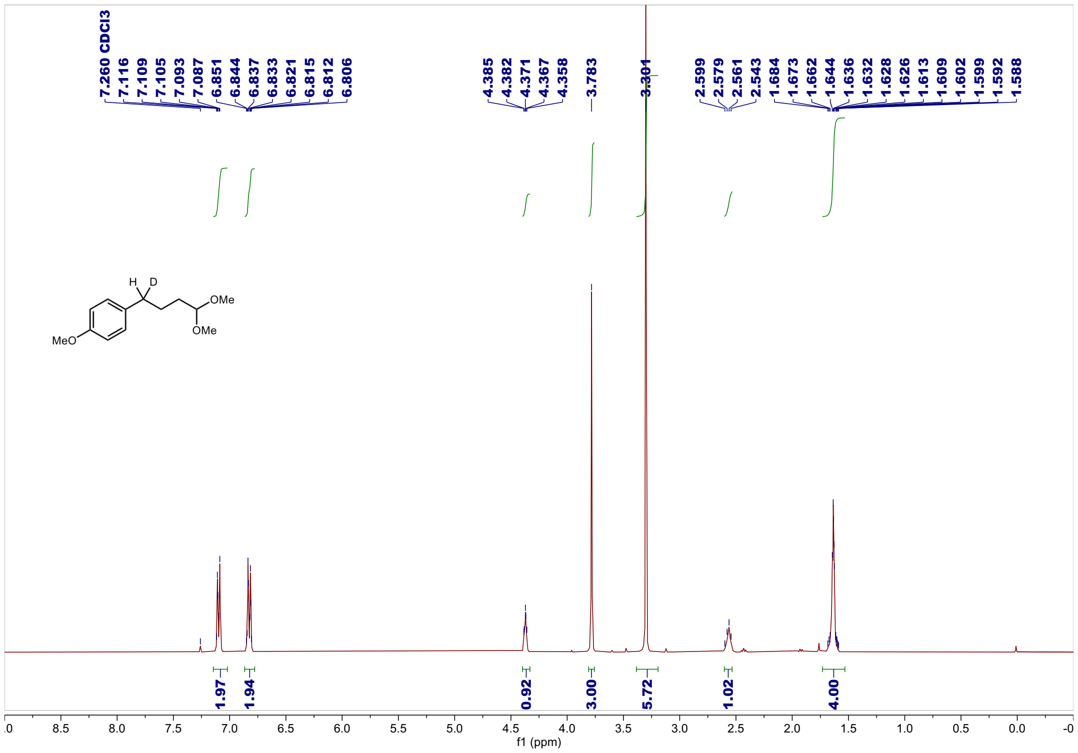

^{13}C NMR 100 MHz, $CDCl_3$

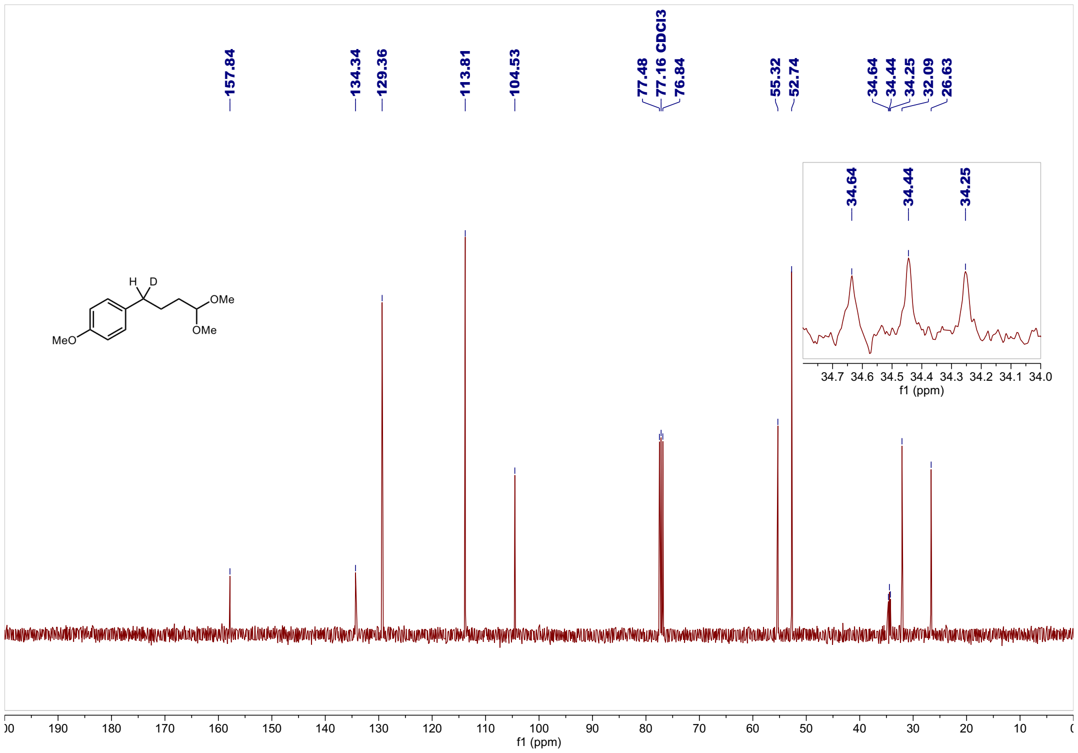

^{1}H NMR 400 MHz, $CDCl_3$

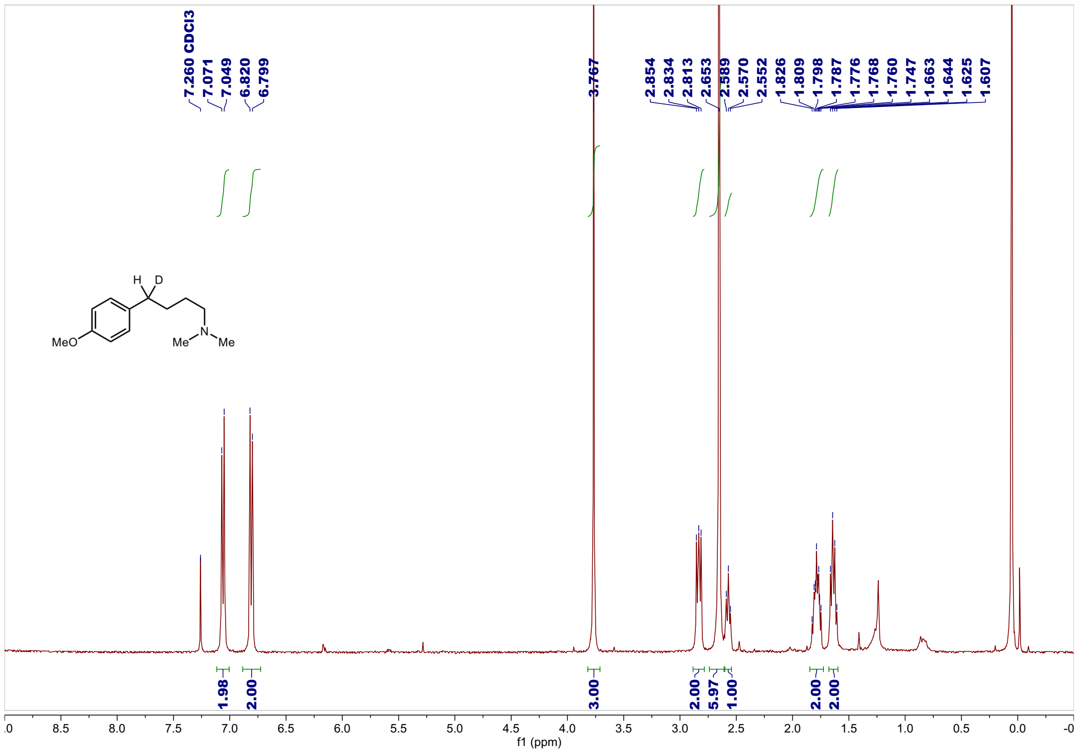

^{13}C NMR 100 MHz, $CDCl_3$

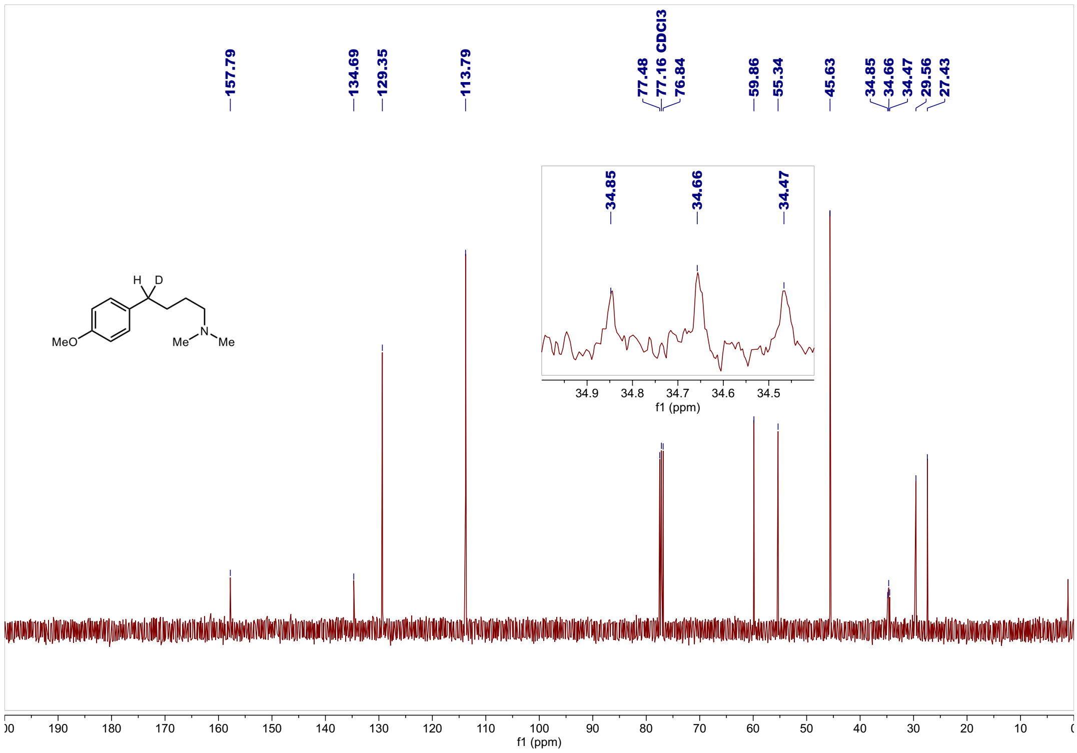

^{1}H NMR 400 MHz, $CDCl_3$

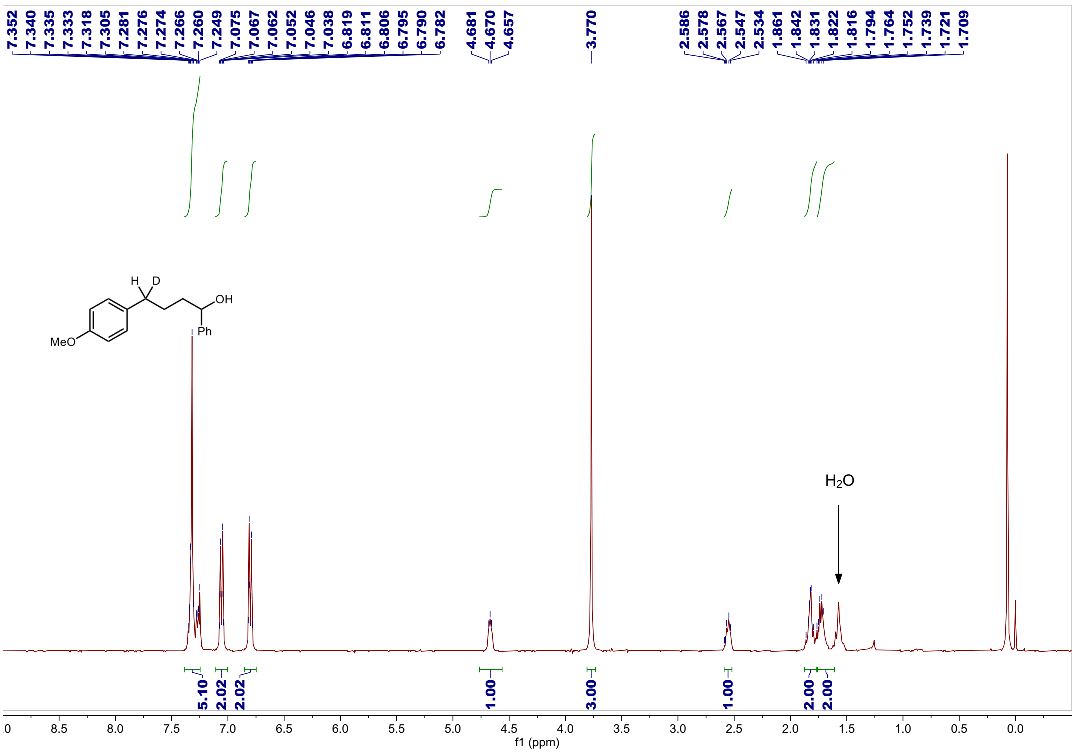

^{13}C NMR 100 MHz, $CDCl_3$

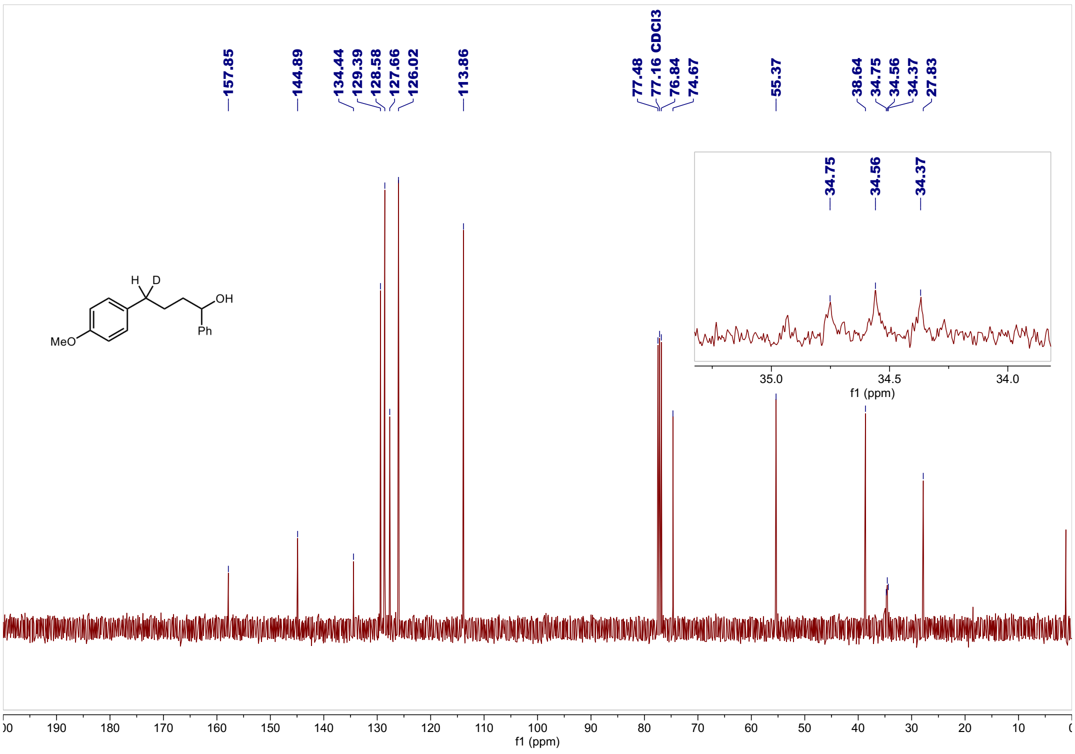

^{1}H NMR 400 MHz, $CDCl_3$

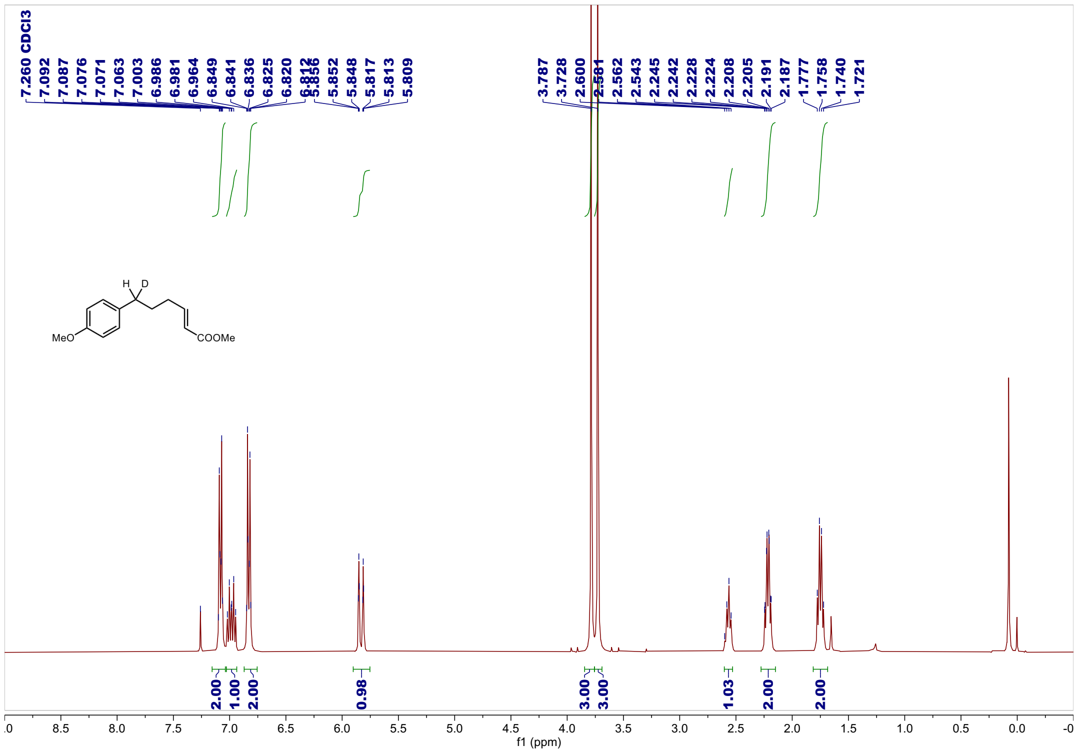

^{13}C NMR 100 MHz, $CDCl_3$

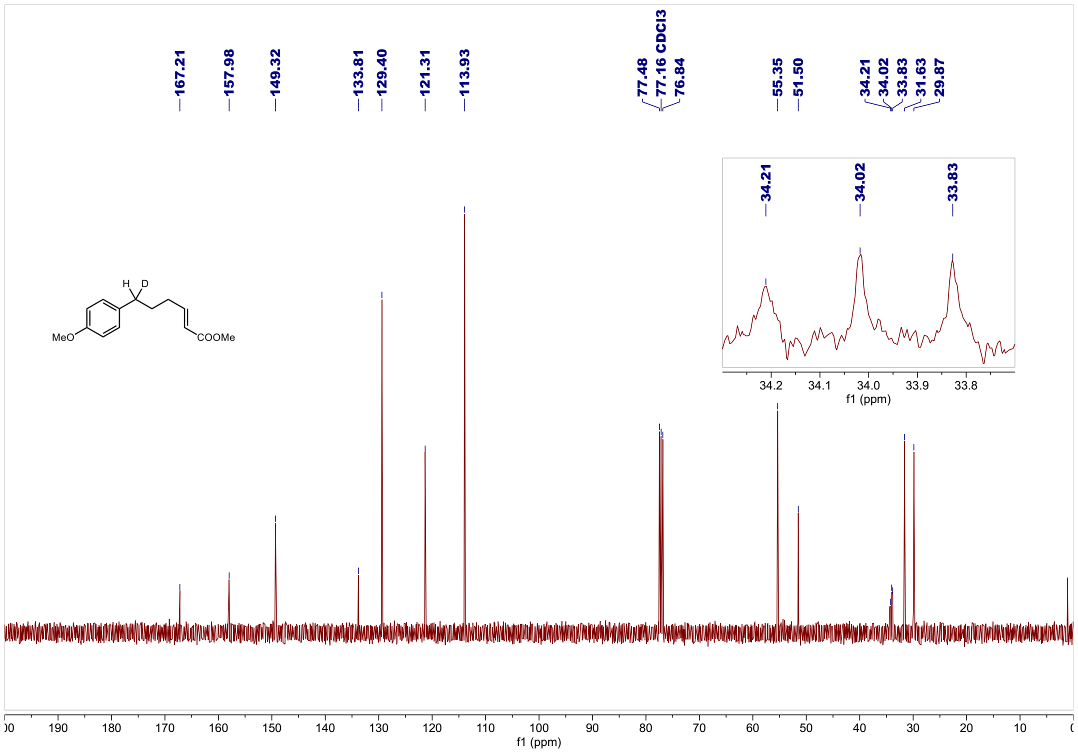

^{1}H NMR 400 MHz, $CDCl_3$

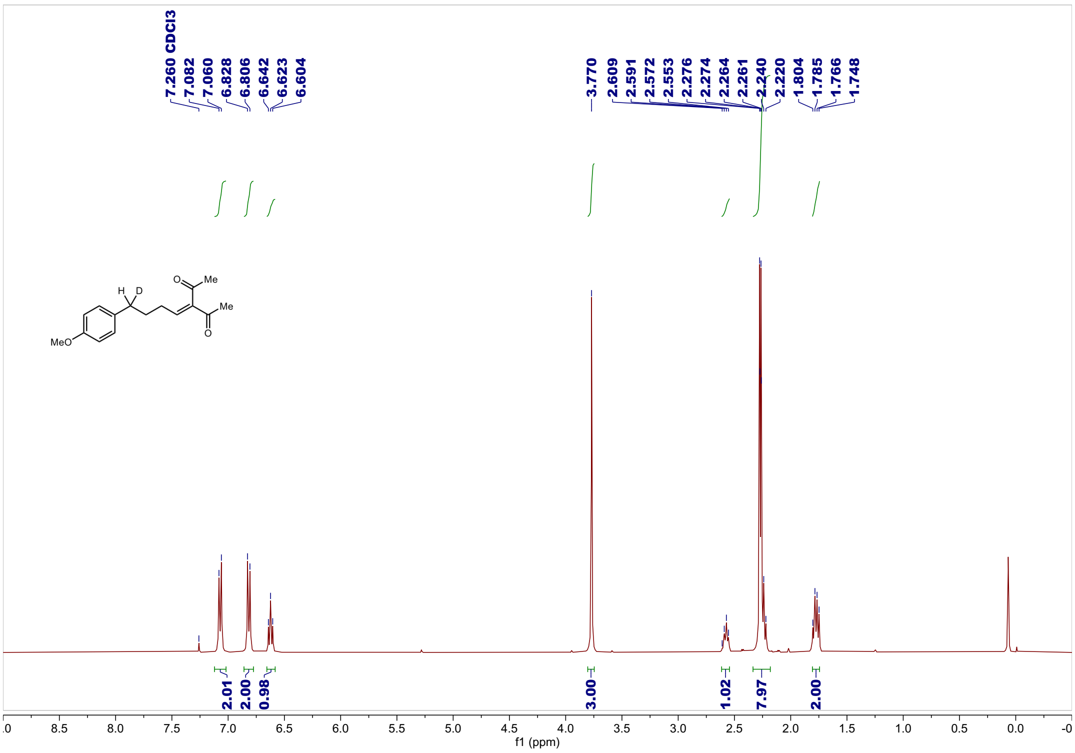

^{13}C NMR 100 MHz, $CDCl_3$

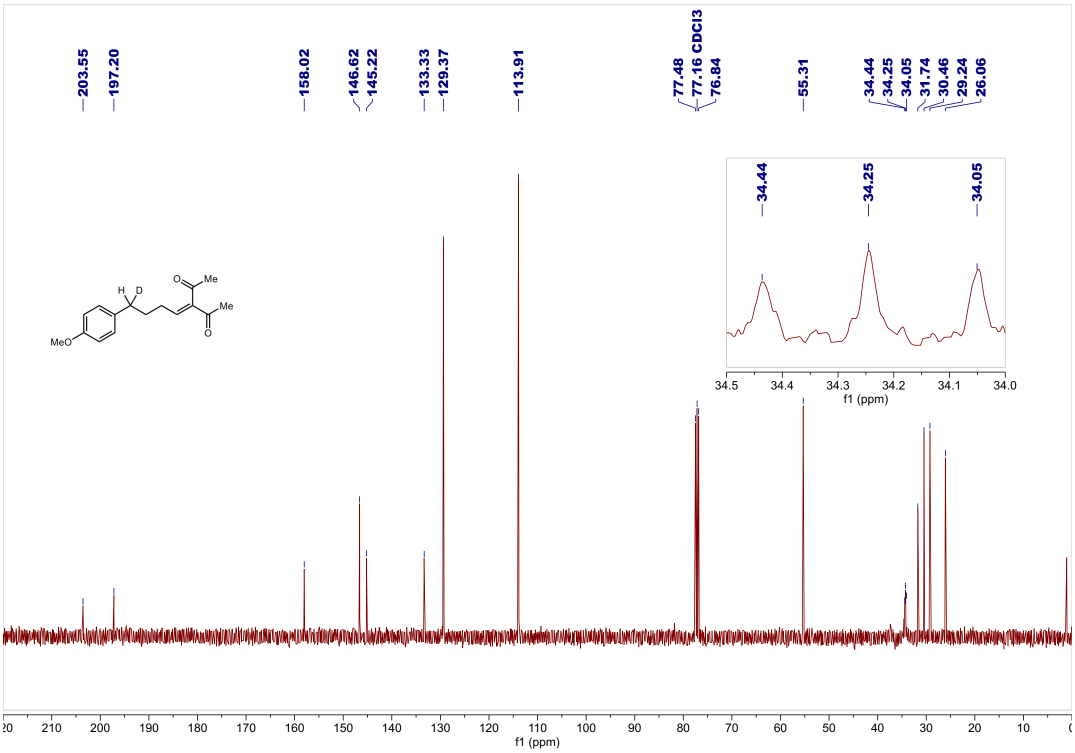

^{1}H NMR 400 MHz, $CDCl_3$

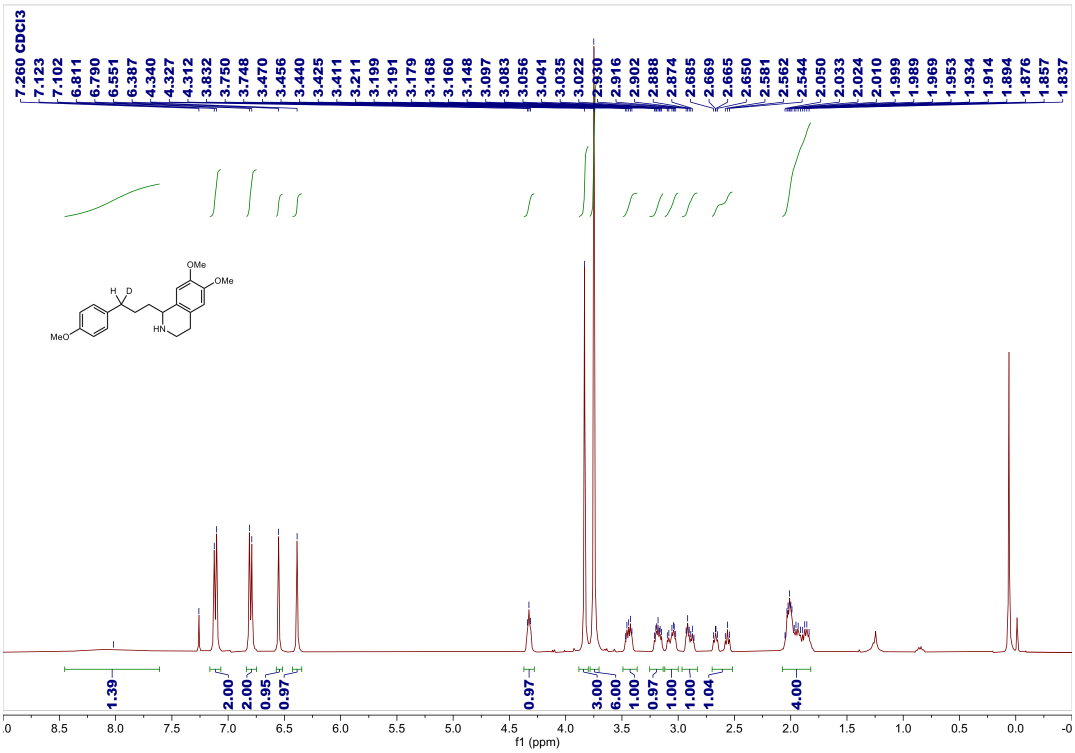

^{13}C NMR 100 MHz, $CDCl_3$

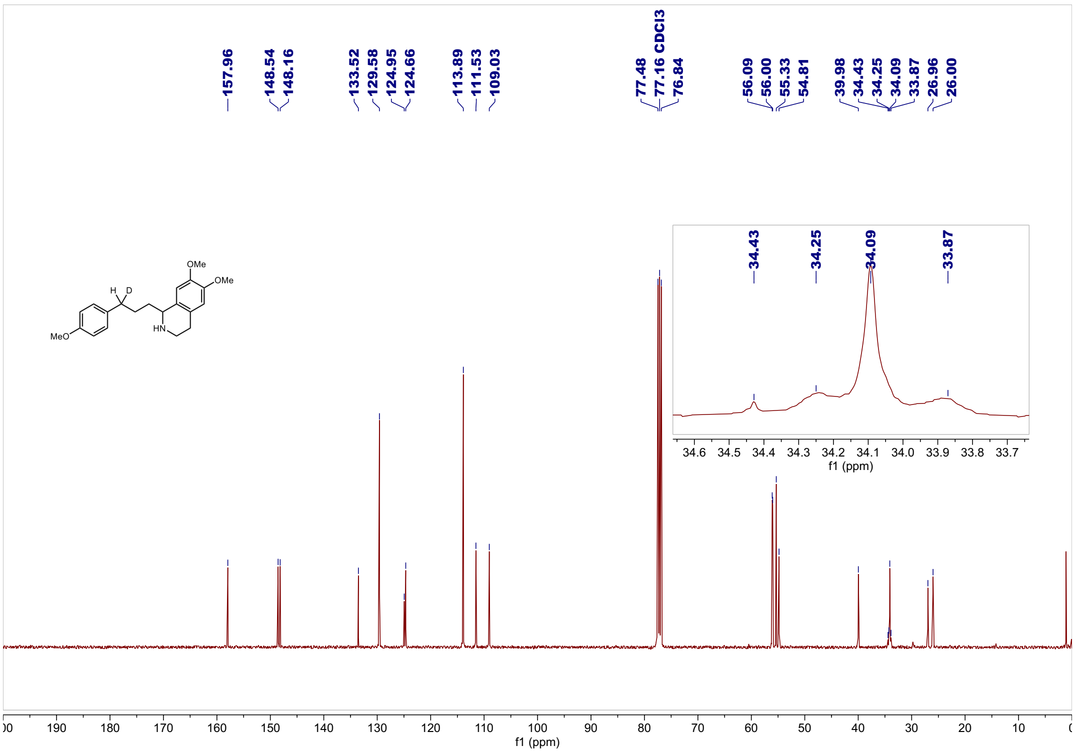

^{1}H NMR 500 MHz, $CDCl_3$

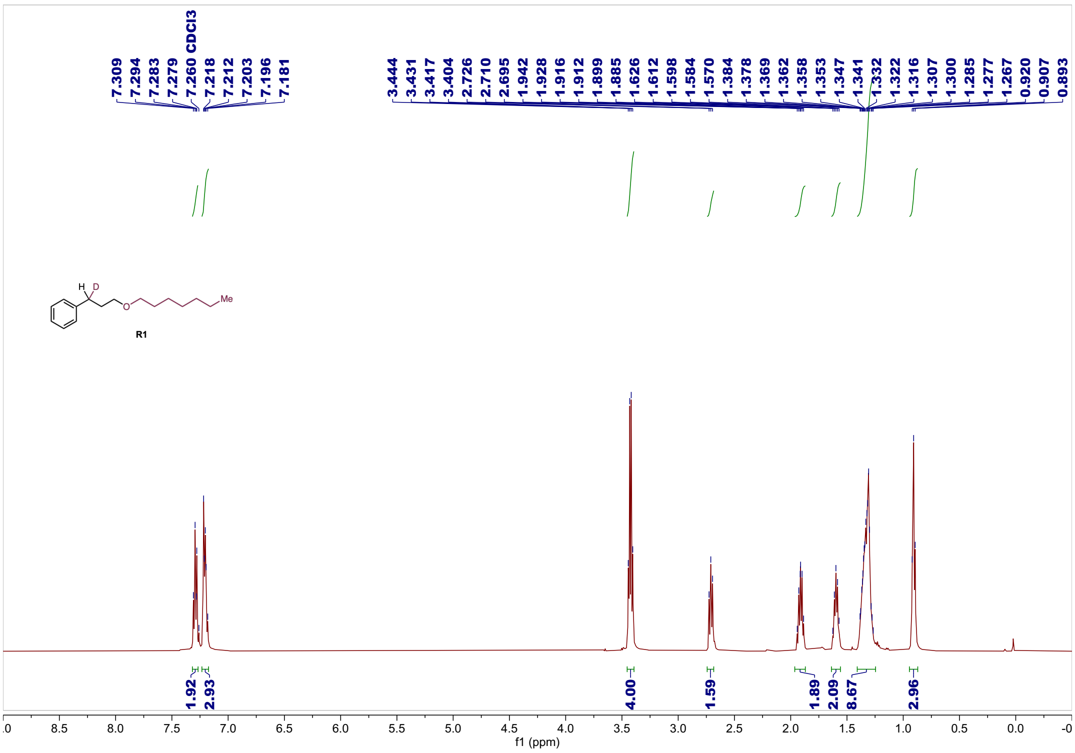

R1

^{13}C NMR 126 MHz, $CDCl_3$

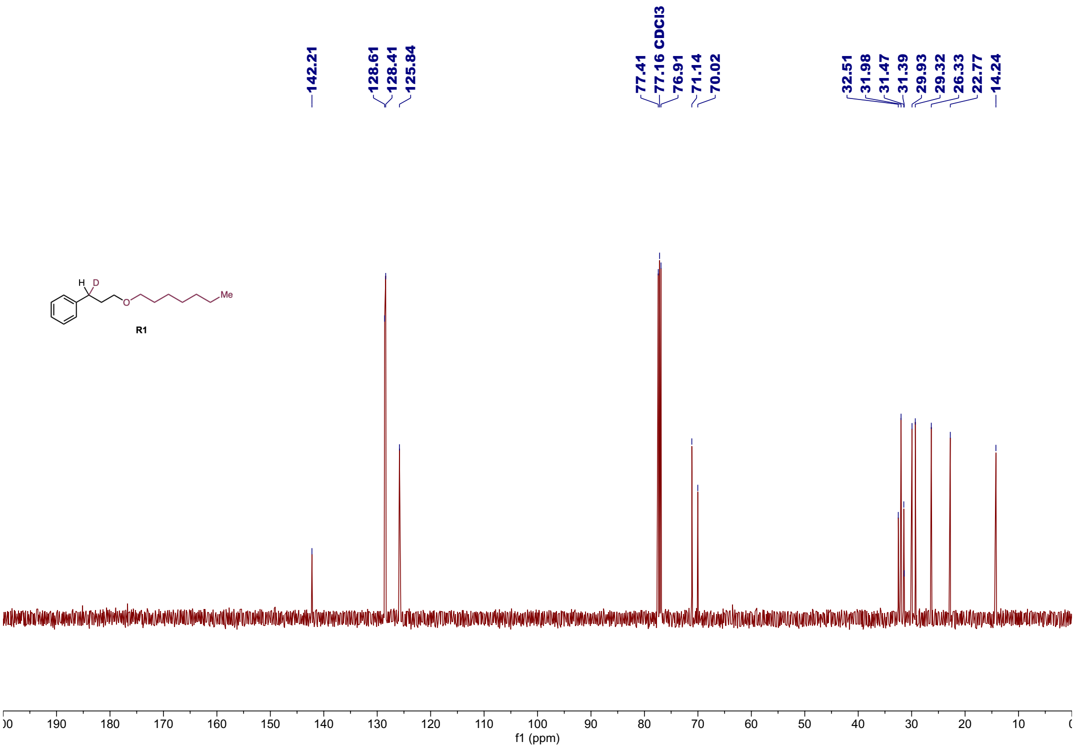

R1

^1H NMR 500 MHz, $CDCl_3$

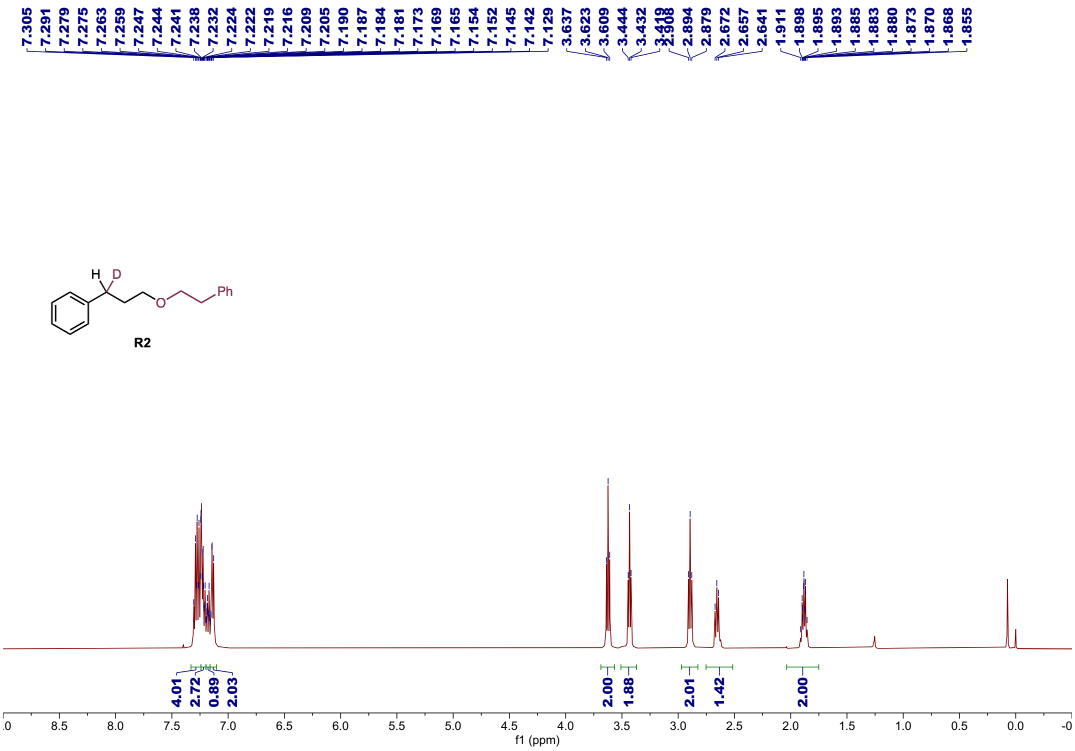

^{13}C NMR 100 MHz, $CDCl_3$

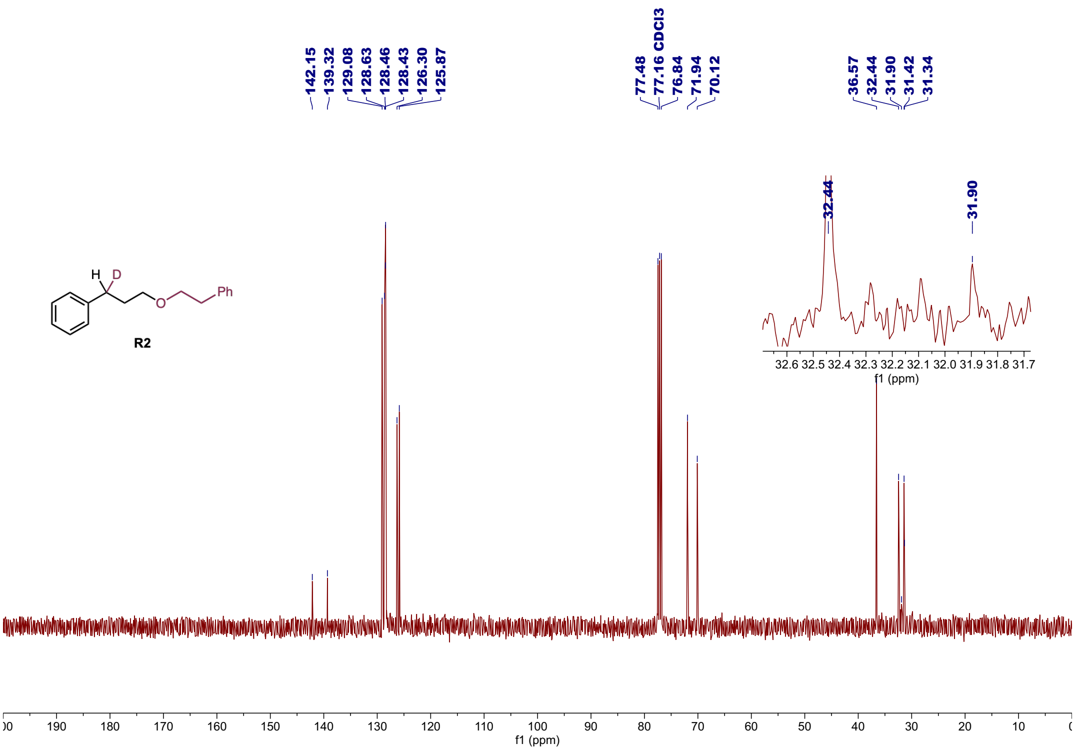

^{1}H NMR 400 MHz, $CDCl_3$

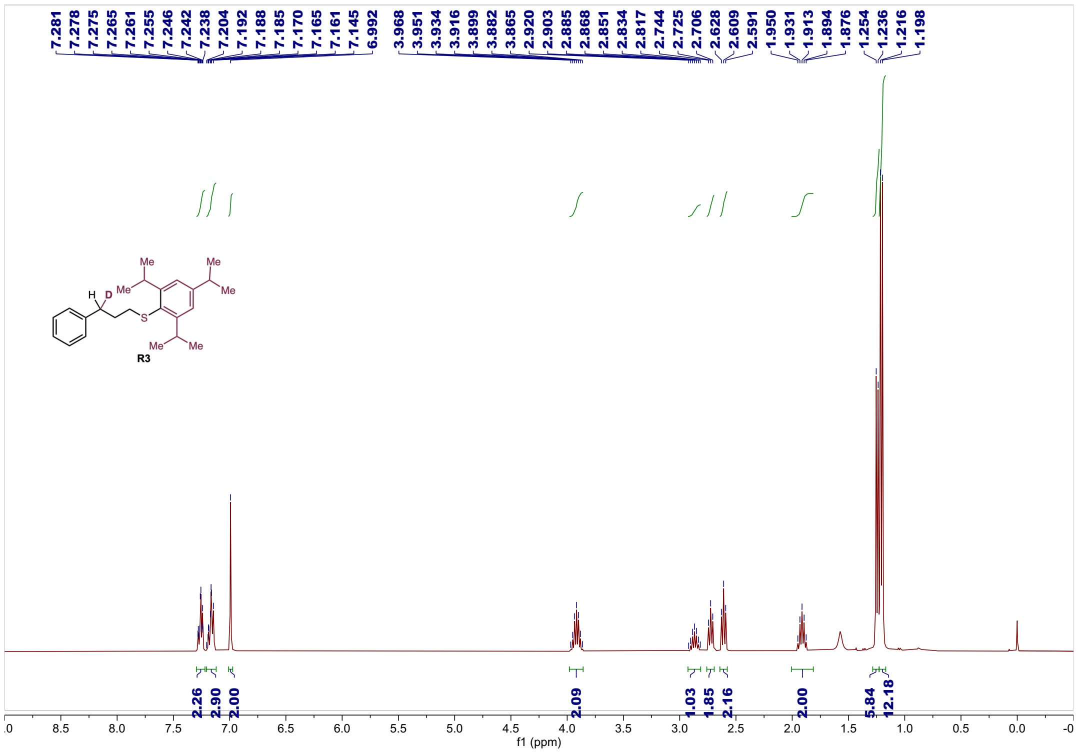

^{13}C NMR 126 MHz, $CDCl_3$

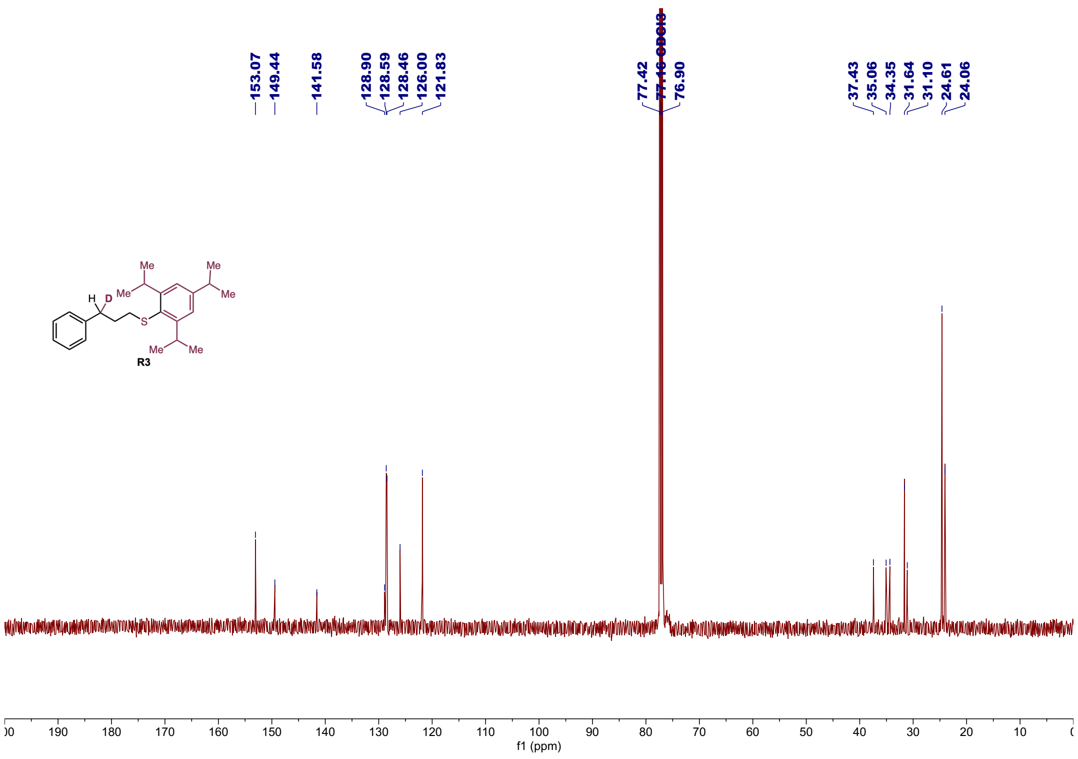

^{1}H NMR 400 MHz, $CDCl_3$

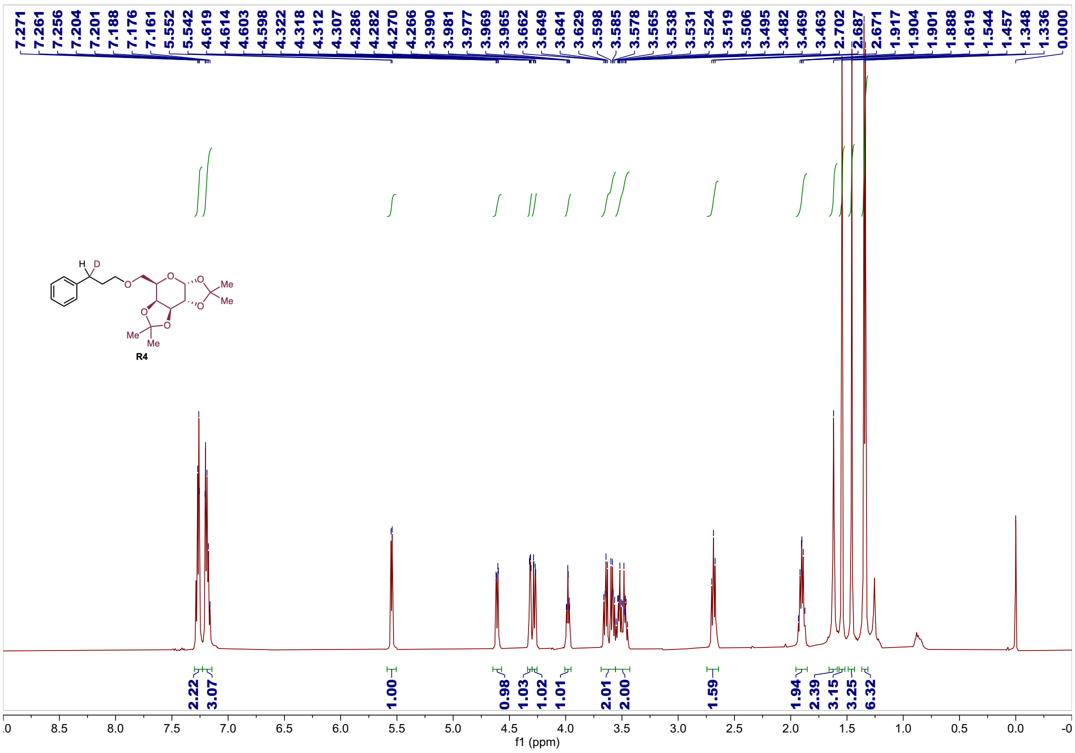

^{13}C NMR 126 MHz, $CDCl_3$

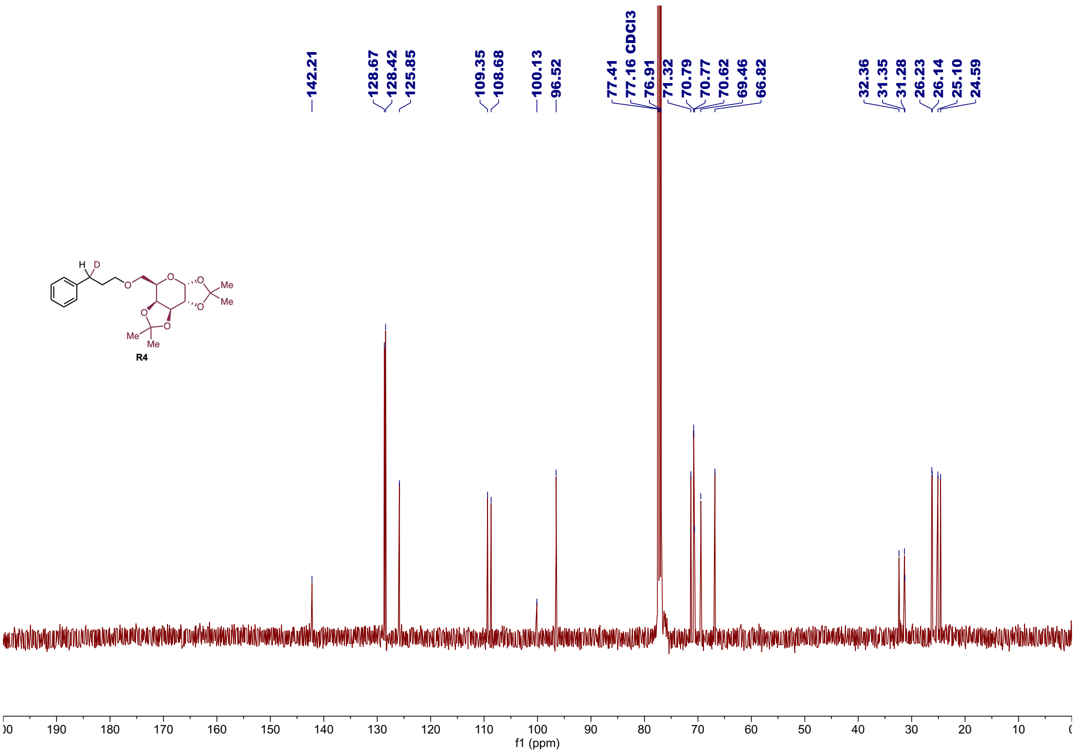

^{1}H NMR 500 MHz, $CDCl_3$

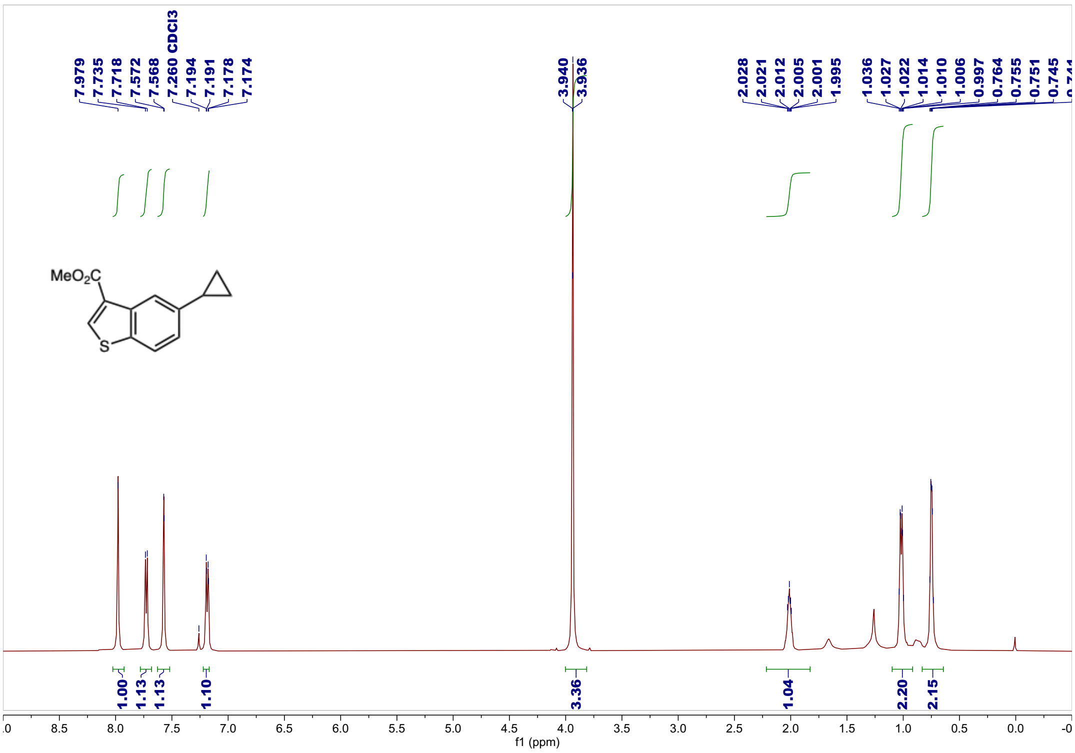

^{13}C NMR 126 MHz, $CDCl_3$

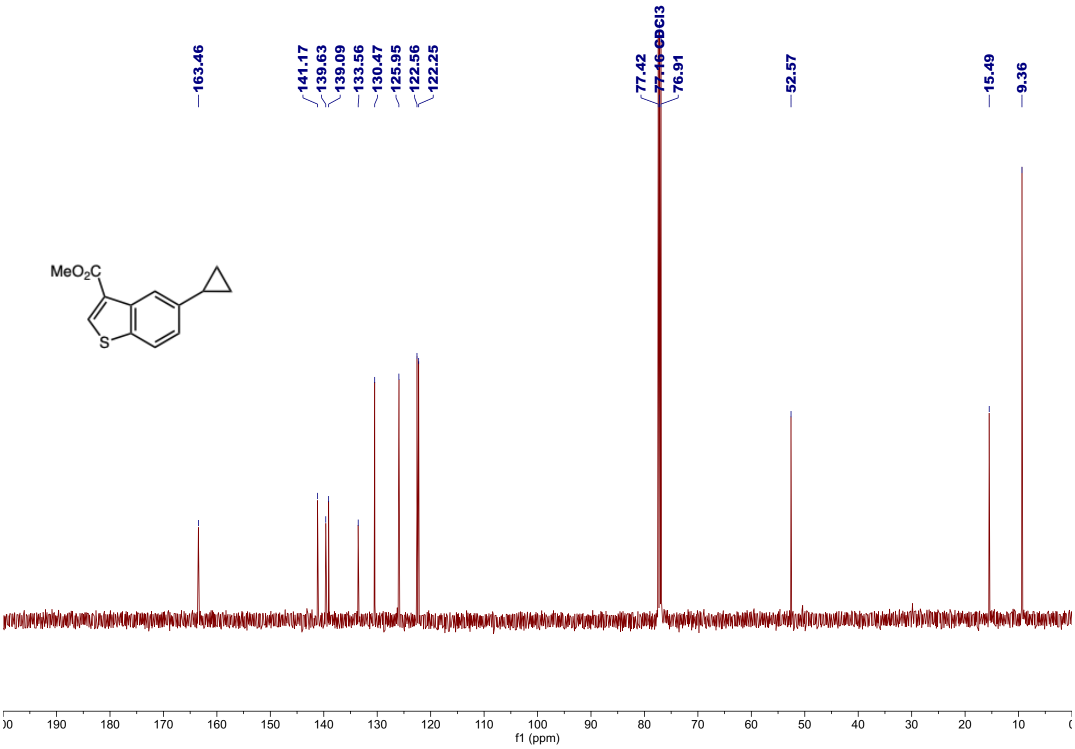

^{1}H NMR 400 MHz, $CDCl_3$

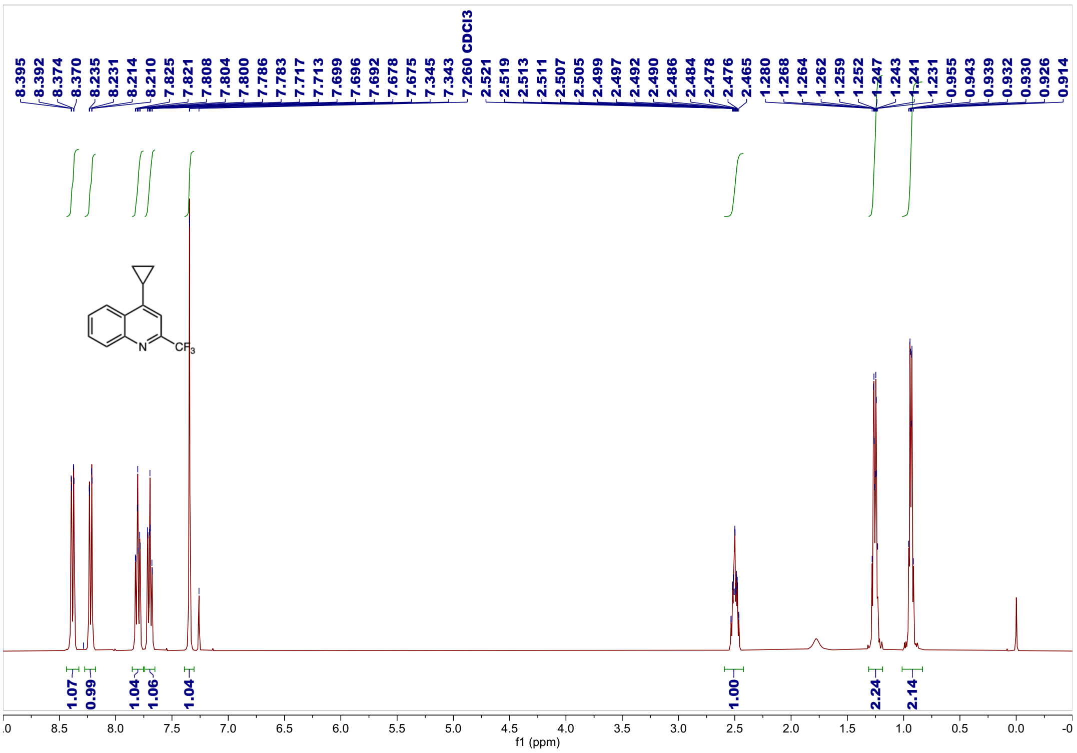

^{13}C NMR 126 MHz, $CDCl_3$

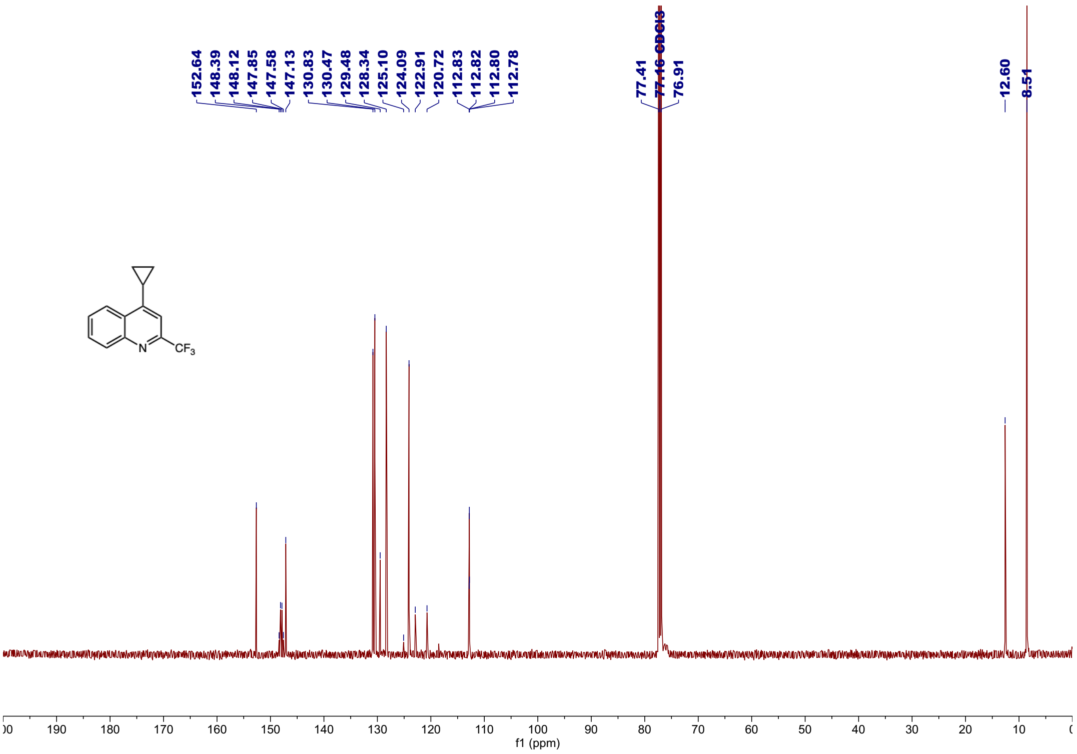

^{19}F NMR 376 MHz, $CDCl_3$

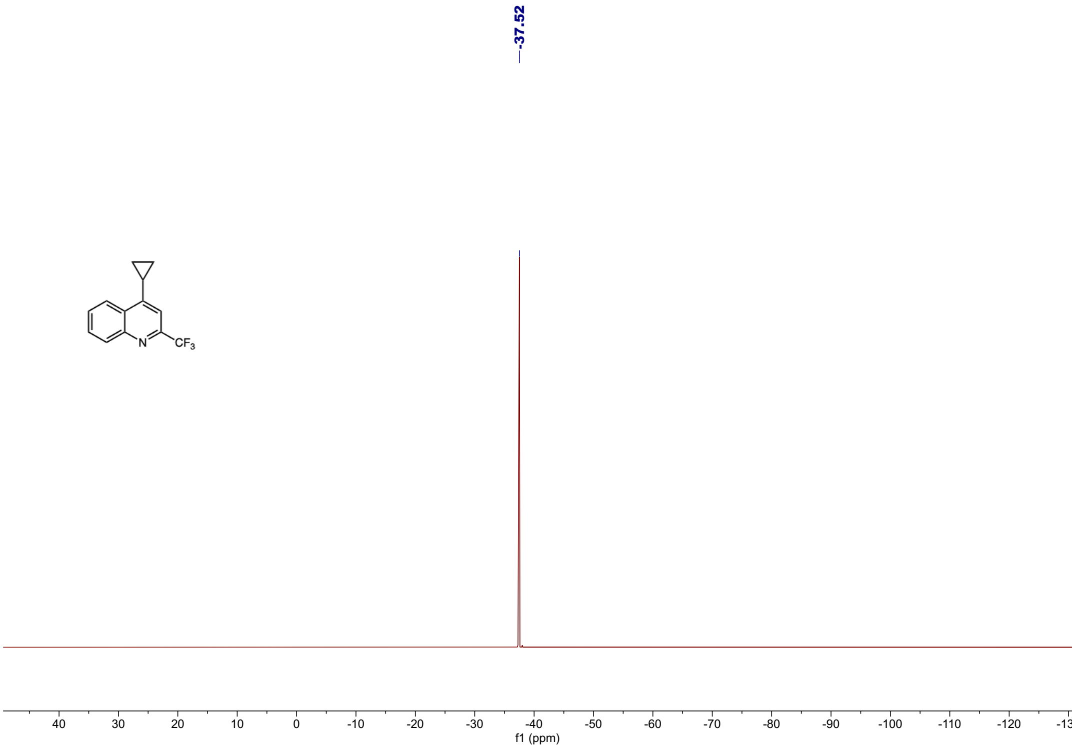

^{1}H NMR 400 MHz, $CDCl_3$

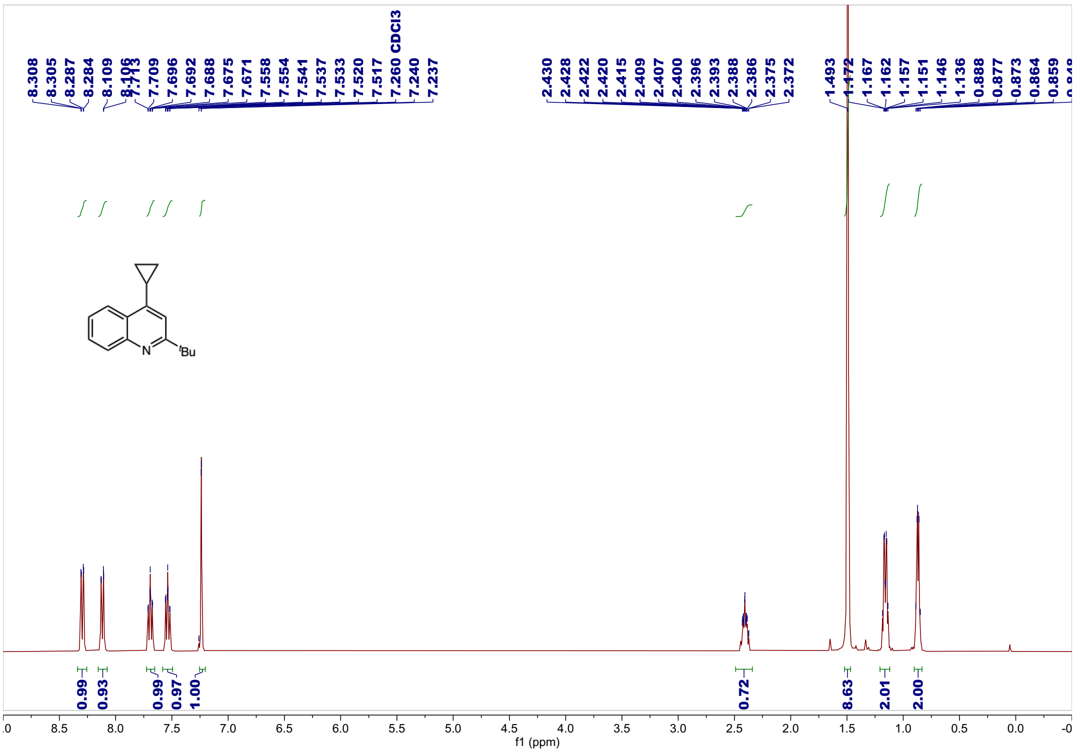

^{13}C NMR 126 MHz, $CDCl_3$

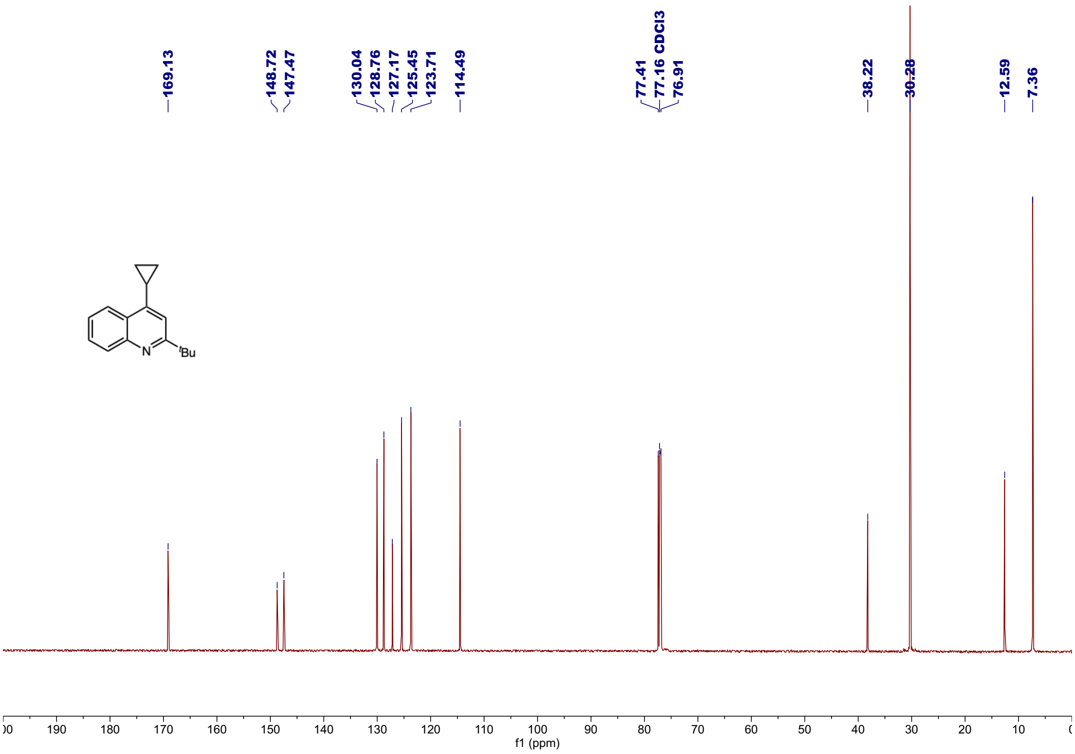

^{1}H NMR 400 MHz, $CDCl_3$

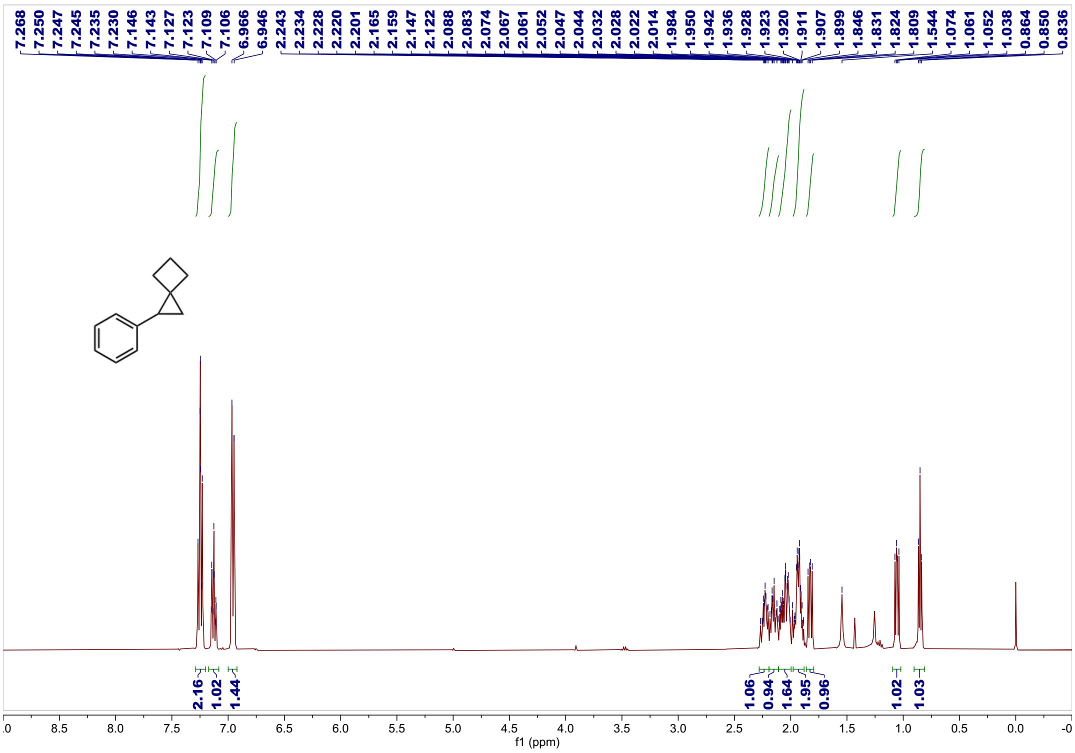

^{13}C NMR 100 MHz, $CDCl_3$

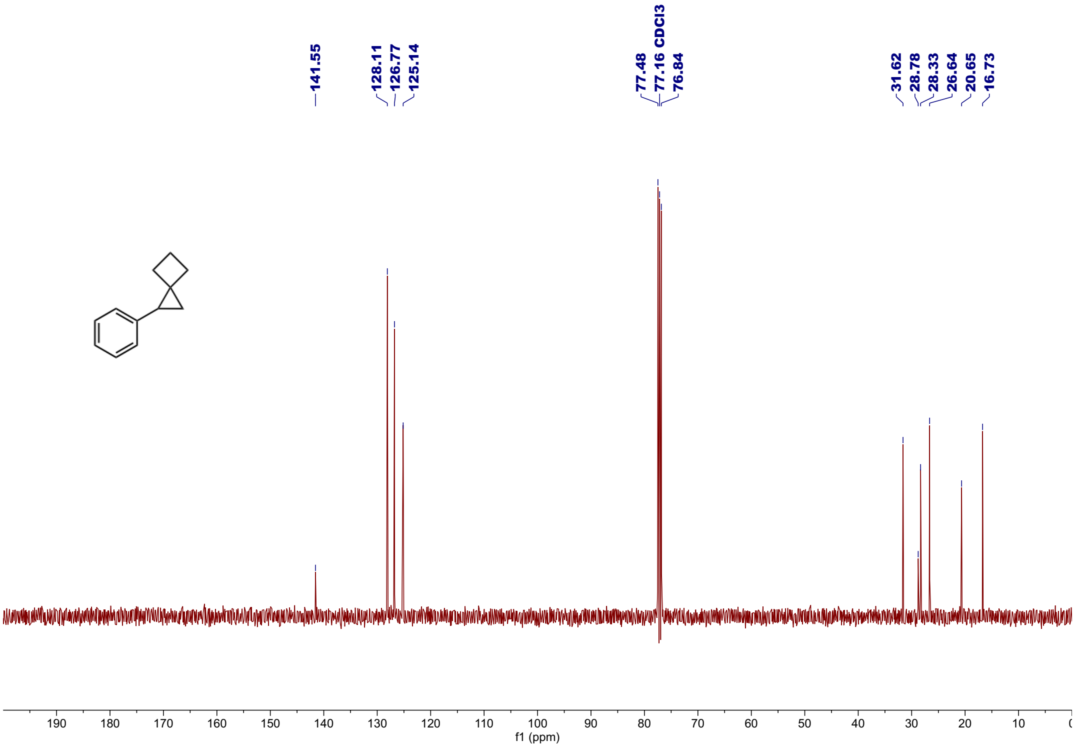

^{1}H NMR 400 MHz, $CDCl_3$

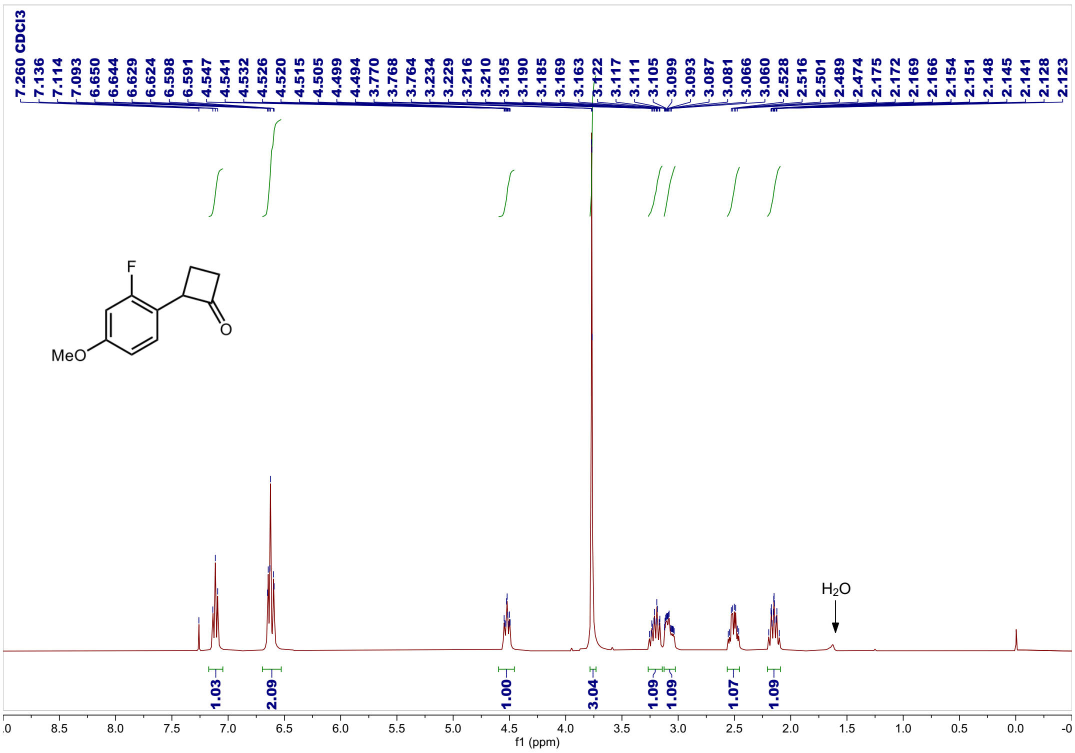

^{13}C NMR 100 MHz, $CDCl_3$

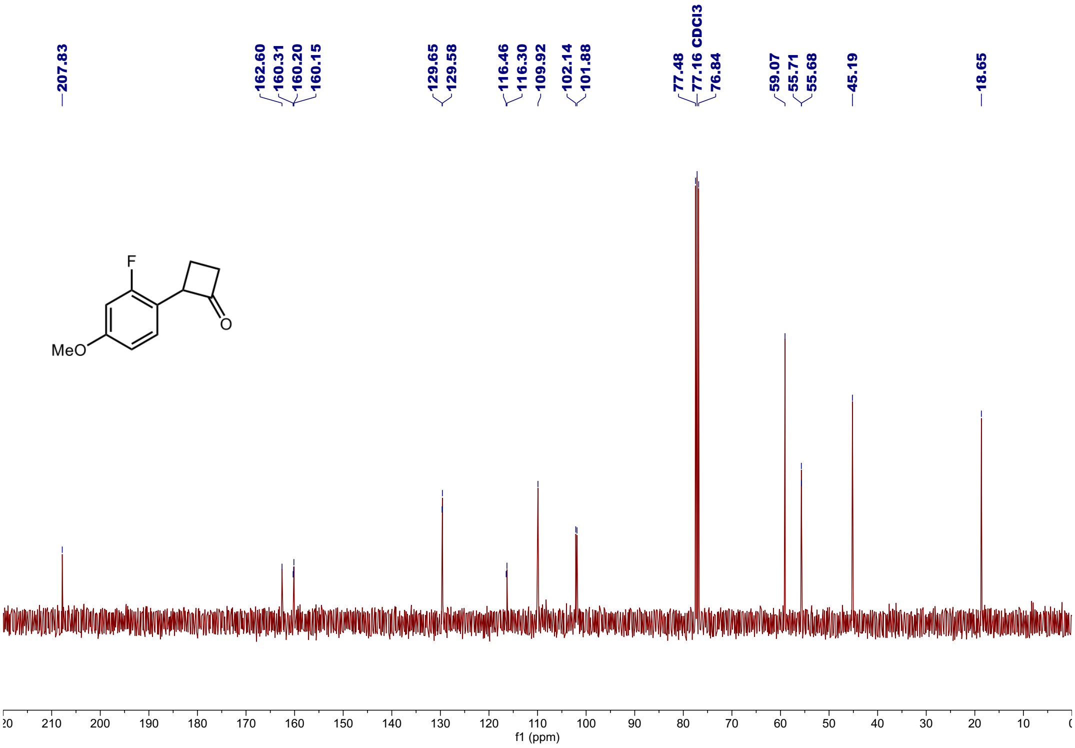

^{19}F NMR 376 MHz, $CDCl_3$

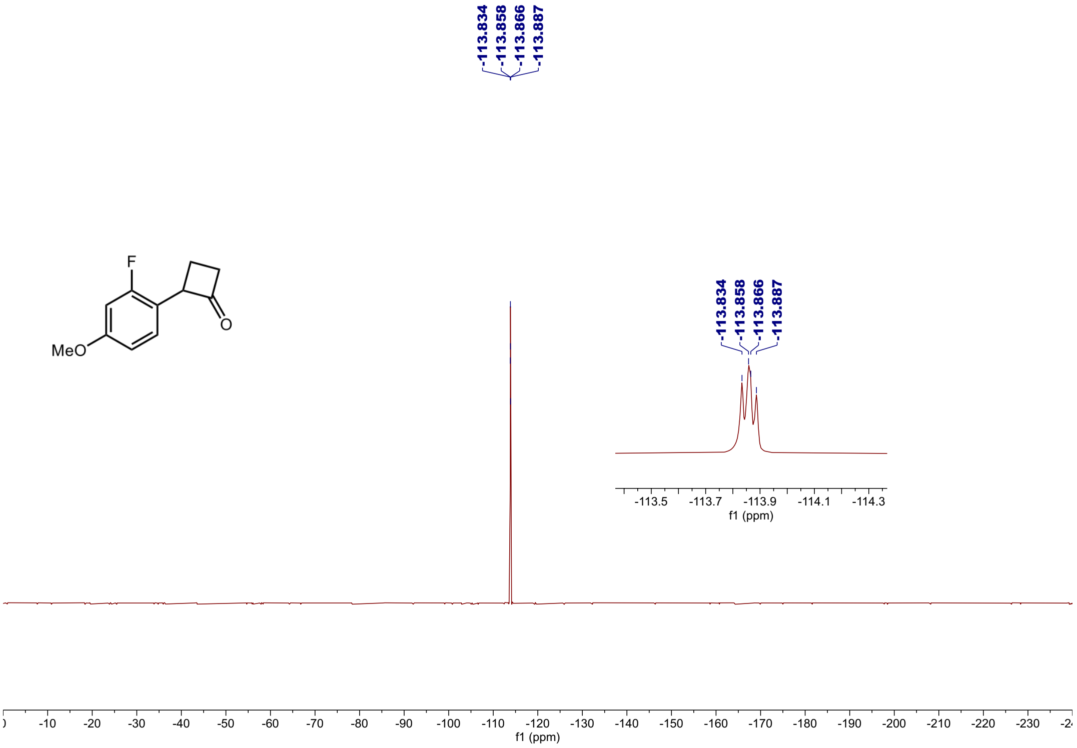

^{1}H NMR 400 MHz, $CDCl_3$

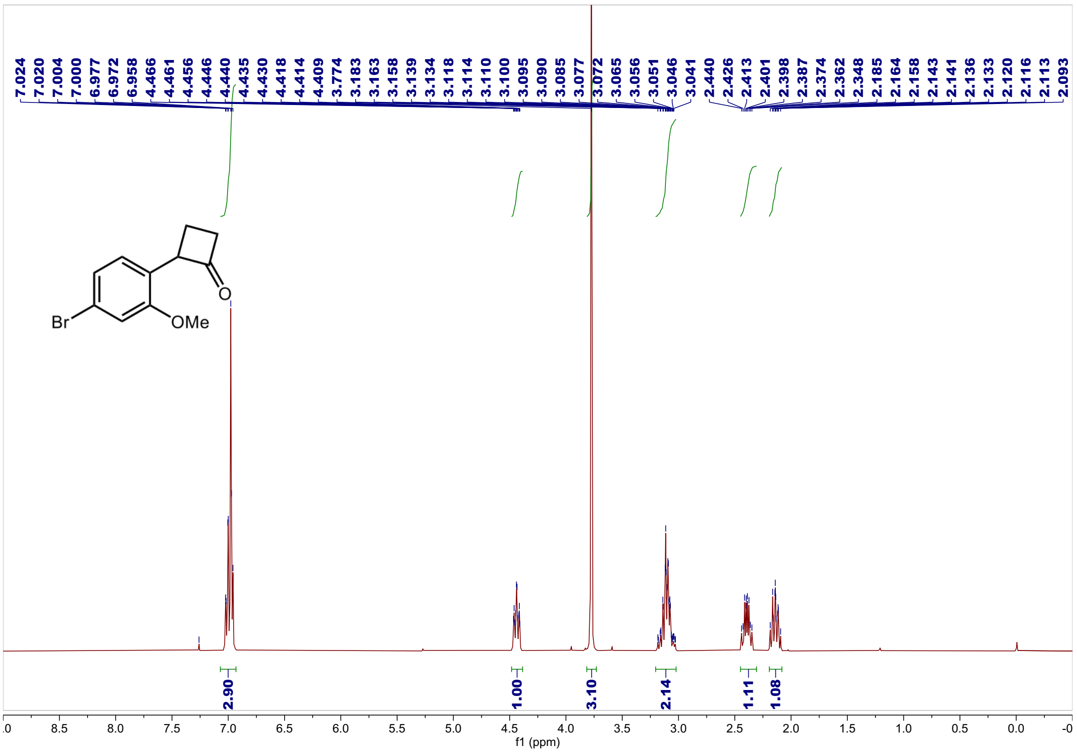

^{13}C NMR 100 MHz, $CDCl_3$

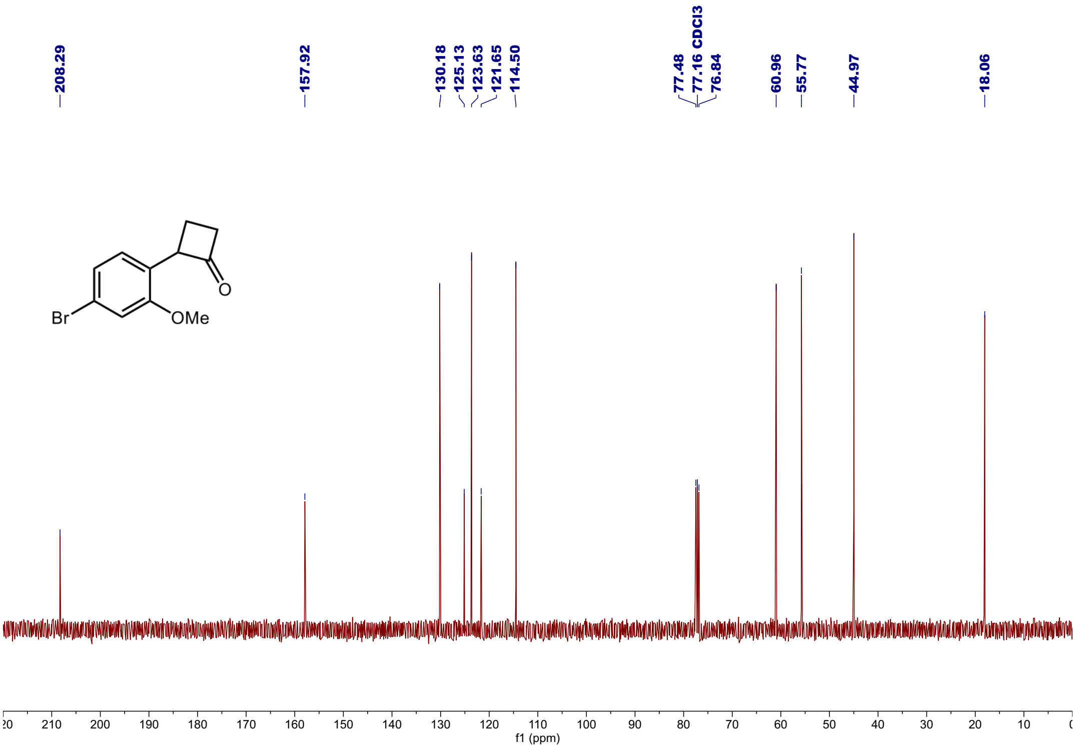

^{1}H NMR 400 MHz, $CDCl_3$

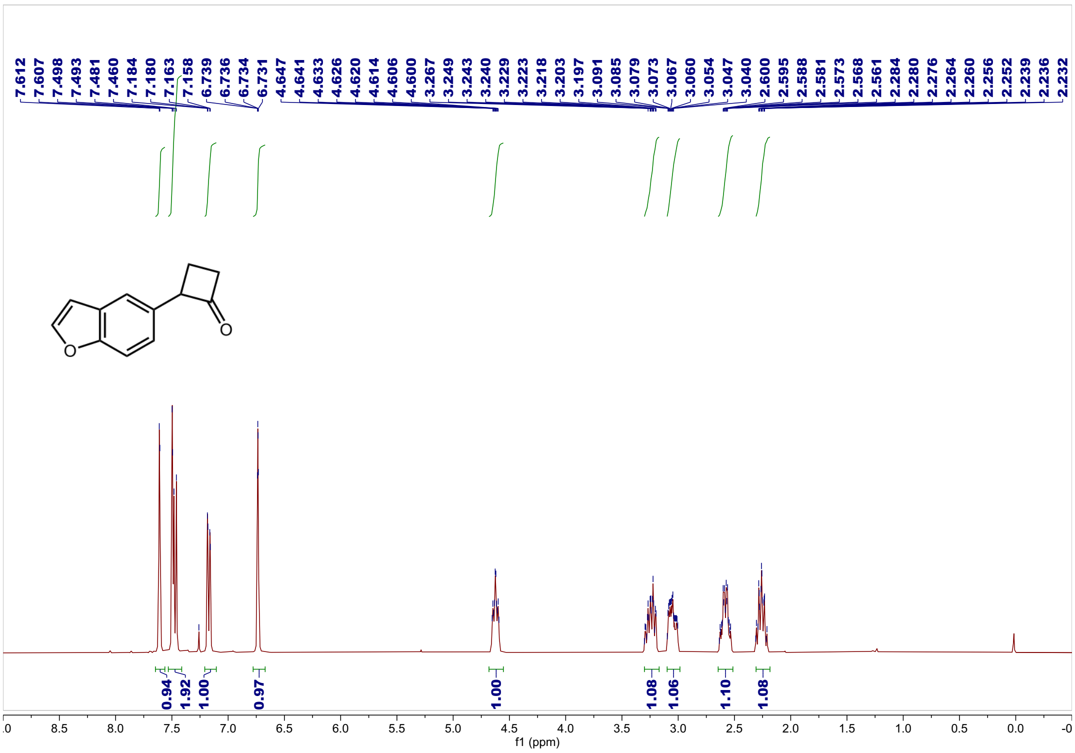

^{13}C NMR 100 MHz, $CDCl_3$

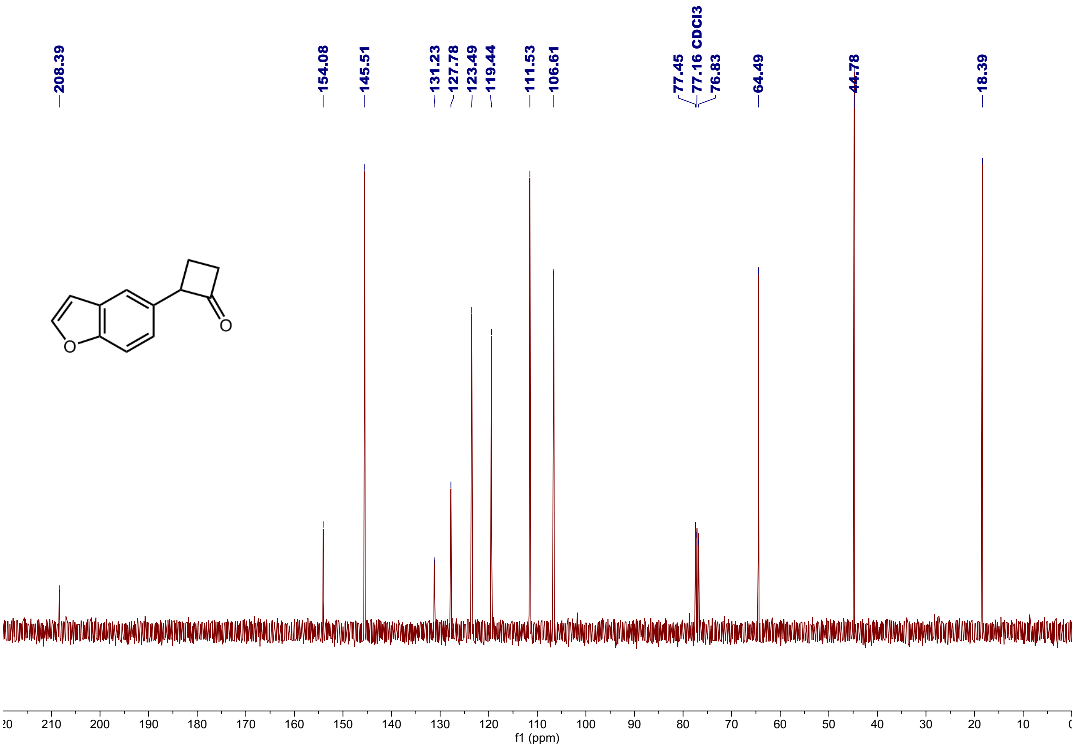

^{1}H NMR 500 MHz, $CDCl_3$

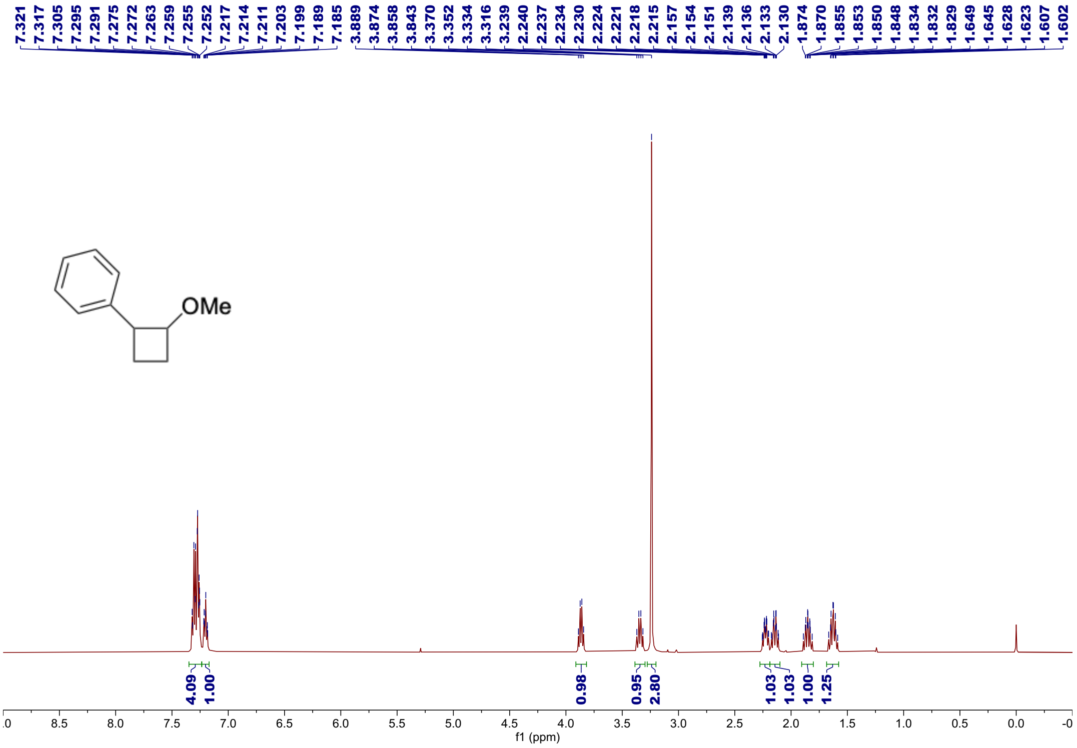

^{13}C NMR 126 MHz, $CDCl_3$

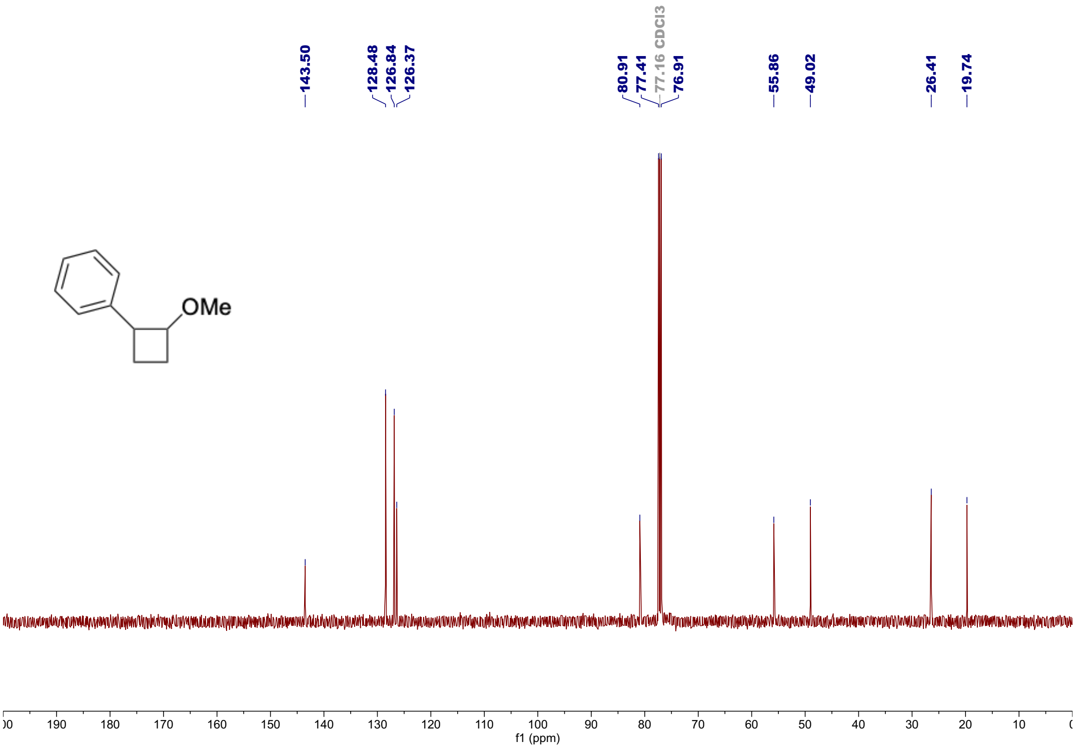

^{1}H NMR 400 MHz, $CDCl_3$

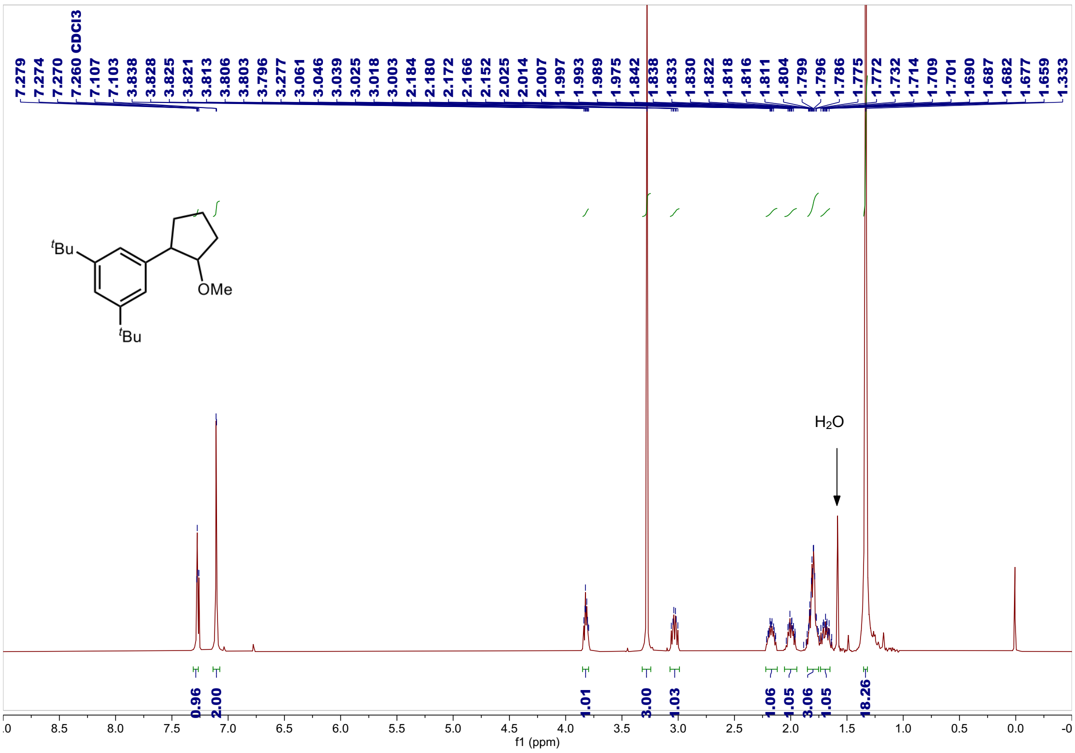

^{13}C NMR 100 MHz, $CDCl_3$

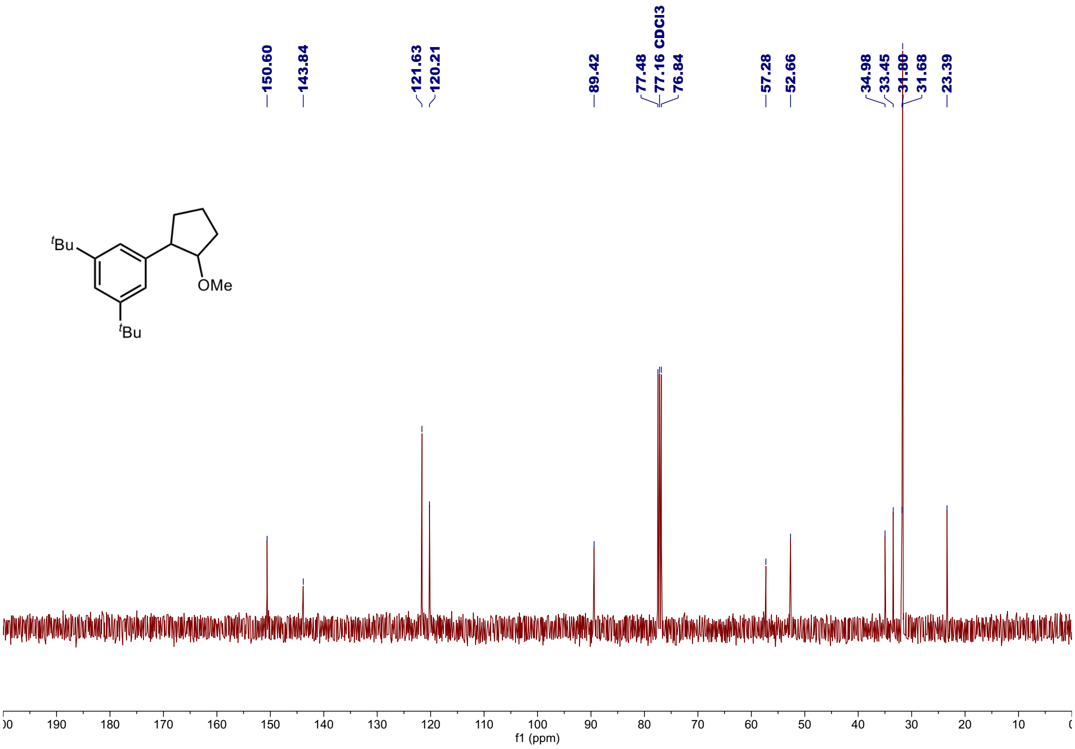

^{1}H NMR 400 MHz, $CDCl_3$

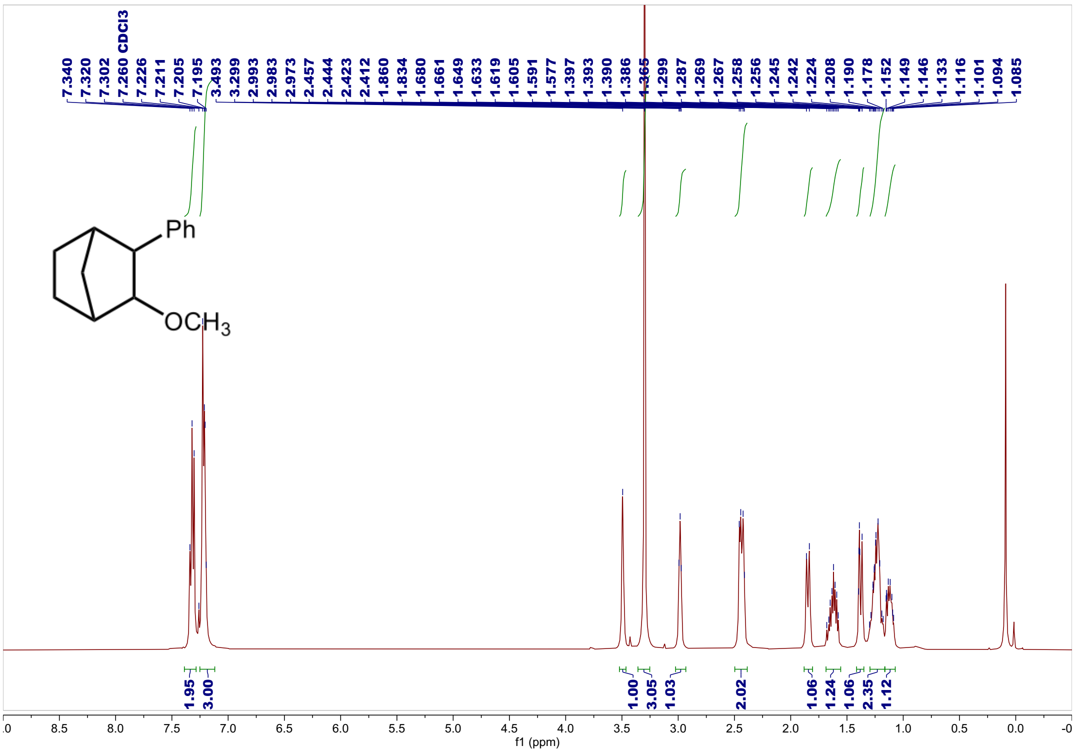

^{13}C NMR 100 MHz, $CDCl_3$

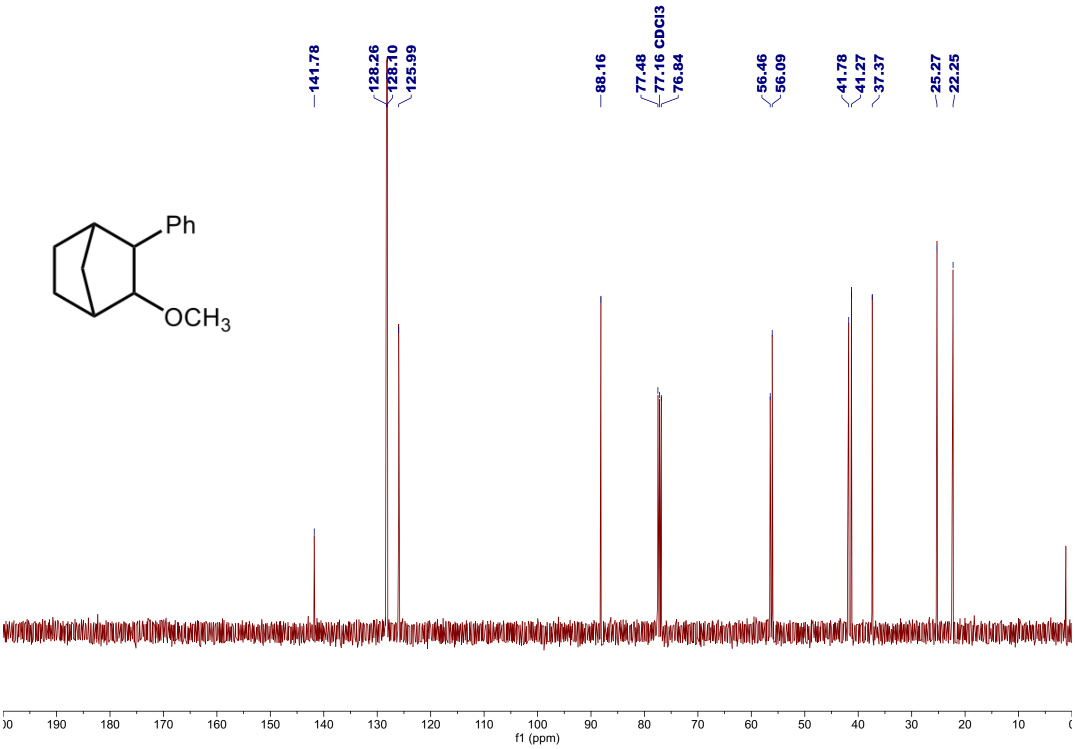

^{1}H NMR 400 MHz, $CDCl_3$

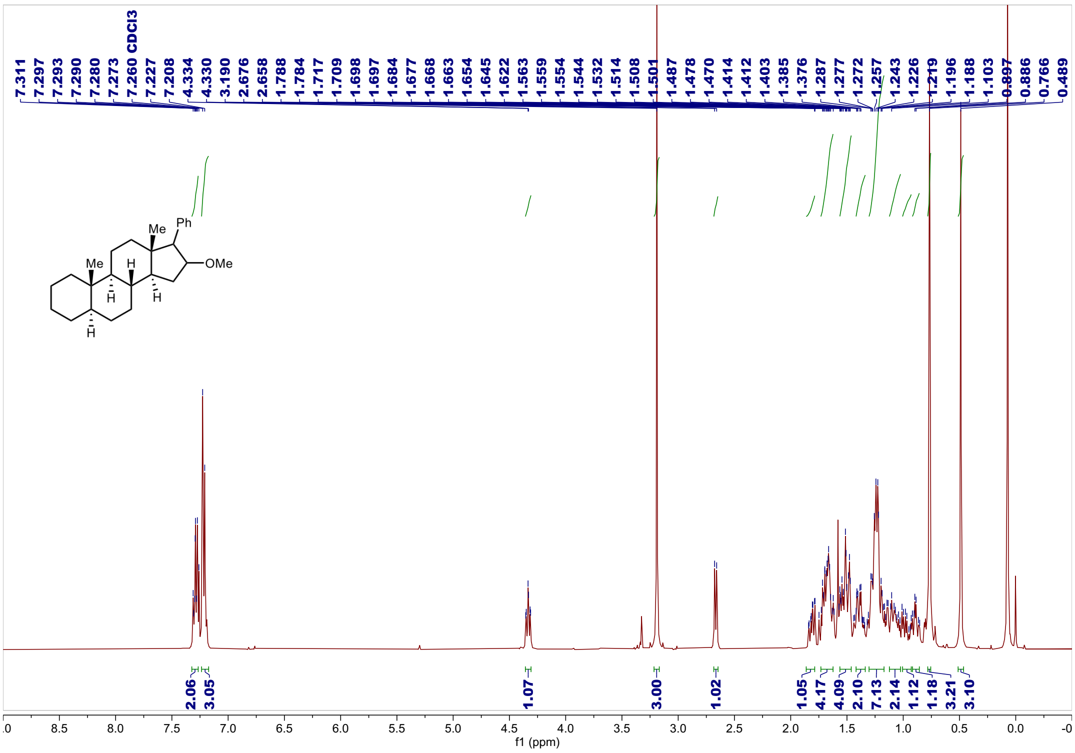

^{13}C NMR 100 MHz, $CDCl_3$

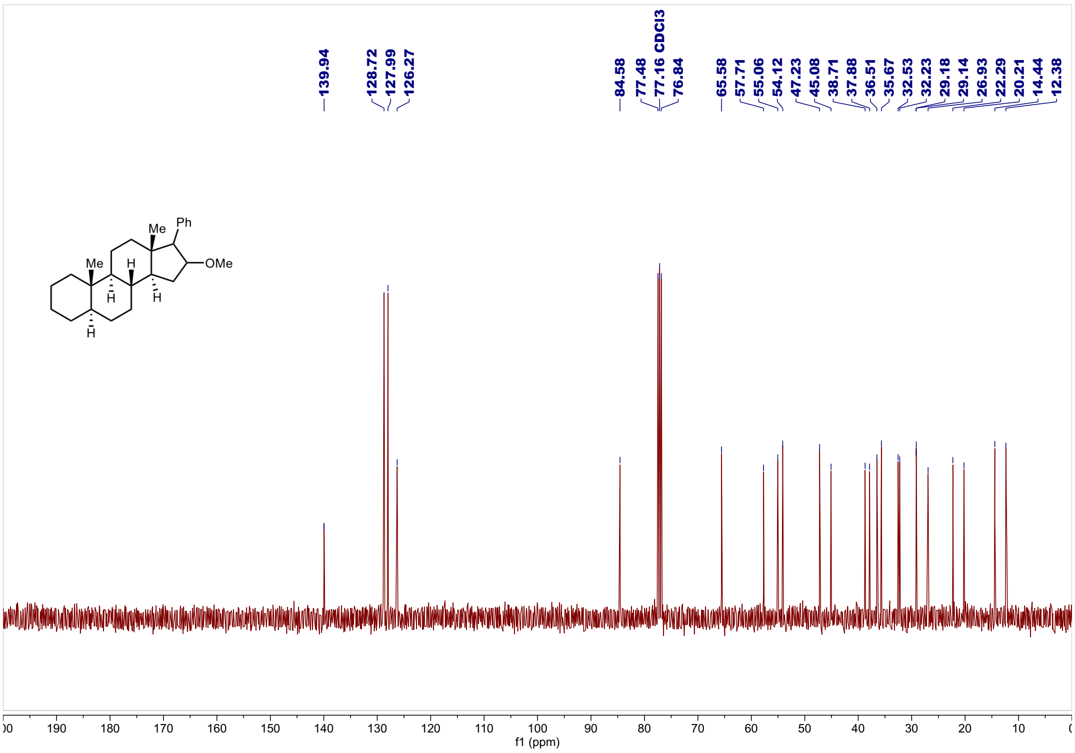

12. Reference

1. Z. Yang, J. Chen and S. Liao, *ACS Macro Lett.*, 2022, **11**, 1073-1078.
2. A. Belyaev, Y.-T. Chen, Z.-Y. Liu, P. Hindenberg, C.-H. Wu, P.-T. Chou, C. Romero-Nieto and I. O. Koshevoy, *Chem. Euro. J.*, 2019, **25**, 6332-6341.
3. T. Delouche, A. Vacher, E. Caytan, T. Roisnel, B. Le Guennic, D. Jacquemin, M. Hissler and P.-A. Bouit, *Chem. Euro. J.*, 2020, **26**, 8226-8229.
4. Z. Zuo, C. G. Daniliuc and A. Studer, *Angew. Chem. Int. Ed.*, 2021, **60**, 25252-25257.
5. M. M. López, N. Jamey, A. Pinet, B. Figadère and L. Ferrié, *Org. Lett.*, 2021, **23**, 1626-1631.
6. L. Zhou, Z. Wang, S. Wei and J. Sun, *Chem. Commun.*, 2007, 2977-2979.
7. T. Kano, Y. Hayashi and K. Maruoka, *J. Am. Chem. Soc.*, 2013, **135**, 7134-7137.
8. K. Nakayama, H. Kamiya and Y. Okada, *Beilstein J. Org.Chem.*, 2022, **18**, 1100-1106.
9. A. B. Charette and H. Lebel, *J. Org. Chem.*, 1995, **60**, 2966-2967.
10. B. Wrackmeyer, *Progress in Nuclear Magnetic Resonance Spectroscopy*, 1979, **12**, 227-259.
11. S. Bhattacharyya, *Syn. Commun.*, 2000, **30**, 2001-2008.
12. M. Winters, J. B. DuHadaway, K. N. Pham, A. Lewis-Ballester, S. Badir, J. Wai, E. Sheikh, S.-R. Yeh, G. C. Prendergast and A. J. Muller, *Eur. J. Med. Chem.*, 2019, **162**, 455-464.
13. K. Sakaguchi, M. Yamamoto, T. Kawamoto, T. Yamada, T. Shinada, K. Shimamoto and Y. Ohfune, *Tetrahedron Lett.*, 2004, **45**, 5869-5872.
14. G. Bartoli, M. Bosco, A. Carlone, R. Dalpozzo, P. Galzerano, P. Melchiorre and L. Sambri, *Tetrahedron Lett.*, 2008, **49**, 2555-2557.
15. G. N. Karageorge, S. Bertenshaw, L. Iben, C. Xu, N. Sarbin, A. Gentile and G. M. Dubowchik, *Bioorg. Med. Chem. Lett.*, 2004, **14**, 5881-5884.
